# An upper temporal limit of action-effect integration as reflected by motor adaptation

**DOI:** 10.1007/s00426-025-02121-4

**Published:** 2025-04-23

**Authors:** Márta Volosin, Olivér Nagybányai Nagy, Bence Neszmélyi, János Horváth

**Affiliations:** 1 Institute of Cognitive Neuroscience and Psychology, HUN-REN Research Centre for Natural Sciences, Magyar Tudósok körútja 2, Budapest, H-1117 Hungary; 2 Institute of Psychology, University of Szeged, Egyetem utca 2, Szeged, H-6722 Hungary; 3 Institute of Psychology, Károli Gáspár University of the Reformed Church in Hungary, Bécsi út 324, Budapest, H-1037 Hungary; 4 Department of Psychology, University of Würzburg, Röntgenring 11, Würzburg, D-97070 Germany

## Abstract

Motor parameters of simple, repetitive actions like tapping, pinching, or pushing a button differ as a function of their action effects – adding a consistent, immediate sound-effect to such actions leads to a decrease in applied force. This action-effect related motor adaptation occurs only, however, when the sound-effect follows actions within about 200 ms, which has been hypothesized to reflect a temporal limit of action-effect integration. Using a university student sample, the present study replicated the effect of action-sound effect delays on force application. Furthermore, given that the perception of action-effect contingencies, and that of temporal relations are deteriorated in schizophrenia, we explored the relationship between the schizotypy trait and the duration of the action-effect related motor optimization window. Participants pinched a force sensitive device every 3 s on their own volition, which elicited a tone with a delay increasing from block to block in 70 ms steps from 0 to 560 ms. The applied force gradually increased with action-effect delay, with an estimated force optimization window size of 290 ms, confirming the importance of temporal contiguity in action-effect related motor adaptation. A Bayes-factor based analysis provided evidence for no correlation between the motor optimization window size and schizotypy.

**Supplementary Information:**

The online version contains supplementary material available at 10.1007/s00426-025-02121-4.

## Introduction

Most of our everyday actions have multiple sensory consequences. For example, when we type, we experience the mechanical contact with the keys (tactile modality), the sound of the keystroke (auditory modality) and we see the resulting change on the screen (visual modality). Contiguity and contingency between the actions and their sensory effects results in the formation of representational links between the actions and their effects (Elsner & Hommel, 2001). Such connections lead to mutual influences between motor and sensory processes. On one hand, actions modulate the sensory processing of stimuli caused by them or coinciding with them (see e.g. Horváth, 2015; Hughes et al., 2013) but motor control processes are also affected by modulations of their action effects (Hommel, 2009; Kunde et al., 2004; Neszmélyi & Horváth, 2018). These action-effect connections also lead to the sense of agency (Haggard & Chambon, 2012; Haggard & Eitam, 2015). By characterizing changes in the perception and processing of a sensory event as a function of the eliciting action, or by characterizing changes in motor parameters of an action as a function of the elicited stimulus, it is possible to gain insight into the nature of such action-effect representations. To this end, in the current study, we investigated how constant delays between simple pinching actions and their auditory consequences influenced the force application patterns of these actions. We also explored whether the pattern of action effect-related motor adaptation was related to individual differences in sub-clinical schizotypy and Big Five personality factors.

The perception of self-generated stimulation is extensively studied in normal (e.g., Cullen et al., 2009; Horváth, 2015; Weiskrantz et al., 1971) as well as clinical (e.g., Ford et al., 2014) samples. In addition, recent studies also demonstrated alterations of various action parameters as a function of the elicited sensory effects (e.g., Pfister et al., 2014, 2023). Previous studies showed that during simple, brief interactions with tactile devices (pinching, tapping, or pressing), participants apply less force when these actions consistently elicit sounds, than in conditions without auditory action effects (Horváth et al., 2018; Neszmélyi & Horváth, 2017, 2018; Volosin & Horváth, 2022). In such cases the elicited sound might be regarded as feedback about the successful interaction with the device, thus participants can lower the applied force. In contrasts, when the auditory feedback is absent and only the tactile feedback is available, there is a higher uncertainty whether the interaction was successful, and participants tend to use more force to ensure the action’s success (Neszmélyi & Horváth, 2017). This suggests that action-induced auditory effects – even when they are not directly task-relevant – are used to optimize the motor parameters of subsequent actions.

The idea that sensory action effects play a central role in the planning and execution of actions fits well with the framework of ideomotor theories such as the Theory of Event Coding (TEC) (Hommel et al., 2001; Hommel, 2019). According to TEC, motor and sensory features are automatically integrated into episodic representations referred to as *event files*. Recent studies indicated that continuous features of actions, like duration or force can be bound into the event file (Varga et al., 2022, 2024), which raises the possibility that sensory features integrated into the event file can have an influence on these features: Ideomotor theories suggest that motor patterns are selected by activating the sensory effects associated with the action. Adding a sound or any other sensory consequence to an action results in a richer event file, which allows more opportunities to represent the action. The weighting of the features in an event file may depend on several factors (e.g., task relevance; other associated features). In the absence of an auditory action effect, it was hypothesized that the tactile feature will be dominant, whereas the presence of an auditory action effect may allow prioritization of the auditory features, and the weighting of the tactile dimension will be less pronounced. Although the ideomotor approach focuses primarily on the selection of motor plans based on already experienced action-effect conjunctions, the idea of optimization could also fit into this framework: Due to the differences between tactile and auditory effects in the information content of the stimuli (only auditory stimuli provide distinctive feedback on action success) and in their connection to motor features (continuous vs. “all-or-nothing”), the dominance of auditory or tactile features might be also reflected in different action control mechanisms.

Action-effect related motor adaptation may be brought about in several (not mutually exclusive) ways and may depend on different types of action representations. The sound elicited by the operation of the response device may well be regarded as feedback on the success of the action by the participants, which allows a “strategic” optimization: the participant may set a target force level that ensures successful interaction with the device while reducing work (i.e., conserving energy). For interactions longer than about 100 ms, there is also evidence for “on-the-fly” adjustments, that is, following force application onset, participants may respond to the onset of the tone, and stop force exertion (or start to release the device) earlier than initially planned (Cao et al., 2020; Varga et al., 2022).

Although theoretically action-effect contingency should provide the necessary conditions for action-effect related motor adaptation, temporal contiguity (proximity) of actions and action effects seems to have decisive influence on whether the type of action optimization described above actually occurs. In a series of experiments conducted by Neszmélyi and Horváth (2018), participants performed voluntary pinching actions with the only constraint that a uniform between-action interval (BAI) distribution between 2 and 6 s should be produced, that is, no strict adherence to a regular temporal pattern was required. Action-effect delays were manipulated blockwise: sinusoidal tones were presented with 0, 50, 100, 200, 400, 800 or 1600 ms delays or no auditory event followed the actions (motor condition). The results showed that force gradually increased as a function of action-effect delay until about 200 ms, however, forces for delays longer than 200 ms did not differ from forces measured in the motor condition. These results suggest that although the auditory feedback contributes to the optimization of motor performance, the causal action-effect relationship is not sufficient per se, as there is a short temporal window in which optimization can occur (Neszmélyi & Horváth, 2018), and this time interval may otherwise not be necessarily accessible for conscious cognition (Aschersleben & Prinz, 1997; Elijah et al., 2016). Neszmélyi and Horváth (2018) speculated that the short window may reflect an automatic action-effect integration (binding) process. Again, it is important to note that this hypothetical process could be one of several mechanisms that contribute to the formation of action-effect representations, as other types of experimental paradigms provided evidence for action-effect integration at longer (1 s) intervals (Elsner & Hommel, 2004).

The importance of temporal contiguity in action-effect integration is also highlighted in studies relying on more complex tasks such as playing instruments (e.g., in playing tone sequences on the piano), where short delays (100–400 ms: Couchman et al., 2012; 250 ms: Finney, 1997; 180 ms: Gates et al., 1974) led to less accurate timing, and higher number of errors in comparison to the immediate auditory feedback condition. Moreover, when longer delays were also included (up to 1050 ms), performance was disrupted strongest at 270 ms action-effect delay (Gates & Bradshaw, 1974). Action synchronization to metronomes also slowed and became more variable when auditory feedback was presented with a delay (Pfordrehser & Dalla Bella, 2011; Pfordrehser & Palmer, 2002, 2006). The deteriorated performance was also correlated with reduced sense of agency for delayed sensory feedback (for a review see Rohde & Ernst, 2016; Wen, 2019), that is, participants felt to a lesser degree that their action led to the auditory effects. When participants were explicitly asked to rate the perceived degree of agency after every trial, trials with action-effect delay decreased the sense of agency both for more complex (producing musical sequences: Couchman et al., 2012) and for simpler actions (actions resulting in visual change on the screen: Haering & Kiesel, 2015; Minohara et al., 2016).

Forming connections between actions and their sensory effects is impacted in certain psychiatric or neurological conditions. In schizophrenia, a subset of symptoms such as delusions or third-person hallucinations (American Psychiatric Association, 2013) reflect difficulties in distinguishing self-generated and externally generated events (Frith & Done, 1988). Several studies demonstrated that patients with schizophrenia showed lower levels of sensory suppression in the processing of self-generated tones (Ford et al., 2014) and their own speech (Ford et al., 2007; Whitford et al., 2011), which is generally interpreted as a reflection of the disruption of action-related sensory predictions (for reviews see: Abram et al., 2022; Bansal et al., 2018; Whitford, 2019). Although less conspicuous, psychosis-like experiences and motor dysfunctions may also occur in the non-clinical population, as well as in first-degree relatives of schizophrenic patients without impairing everyday functioning (Hirjak et al., 2018; Mason, 2015; Meehl, 1962, 1990; Oestreich et al., 2016). Thus it seems reasonable to hypothesize that individual differences in forming action-effect representations may correlate with non-clinical schizotypy or schizotypal personality (a cluster of personality traits characterized by possible presence of unusual perceptions, magical or odd thinking, inappropriate affect, cognitive disorganization, social withdrawal and anhedonia based on the feeling of paranoia, as well as discomfort with close relationships, American Psychiatric Association, 2013; Mason, 2015; Ross et al., 2002). Indeed, when contrasting sensory suppression of self-generated sounds in individuals with high and low levels of schizotypy, a pattern similar to that obtained by contrasting patients with schizophrenia and healthy controls was found (Oestreich et al., 2015, 2016). In contrast, while the amount of sensory suppression was not associated with schizotypal traits, the peri-motor representation of sense of agency (i.e., readiness potential) was decreased in individuals with high schizotypy, suggesting lower reliance on the efference copy of the motor commands (Luzi et al., 2024). Behavioral studies demonstrated that when the delay between actions and a resulting tone was varied from 100 to 900 ms, high schizotypal participants showed lower sense of agency in the 100 ms condition but not at intervals of 200 ms or longer (Pan et al., 2021). This is in line with results of Luzi et al. (2024) who found no association between schizotypy and sense of agency at action-effect delays of 200, 500 and 800 ms. Utilizing a shorter time scale (from 0 to 135 ms in 15 ms steps), the high schizotypal group started to experience decreased sense of agency significantly earlier than the low schizotypal group (Asai & Tanno, 2008). These results indicate that despite the presence of action-effect contingency, high schizotypal individuals’ ability to recognize the sensory consequences of their own actions is reduced for shorter action-effect delays. Furthermore, individuals with high level of schizotypy reported lower sense of agency when they had to judge whether a cursor was moved by them or by the experimenter (Asai & Tanno, 2007), and they showed abnormal response patterns to other-produced actions (Itaguchi et al., 2018).

Besides schizotypy, other personality traits might also influence how action-effect links are processed. One might speculate, for example, that people who have a reduced sense of control in a general sense perceive the relationship between their actions and the consequences of these actions as weaker than those characterized by a higher sense of control. Since several explanations of action optimization rely on recognizing the connection between action and effect, such differences in personality might be also reflected in the use of auditory action effects in action control. Schwarz et al. (2022) demonstrated that the Big Five personality trait framework (Digman, 1990) can capture differences in the perception of the action-effect connection: In an environment characterized by dynamically changing action-effect contingency (from 50 to 100%), participants with higher levels of the Big Five Neuroticism and those with lower level of Big Five Openness traits were experiencing lower level of sense of agency. It is an open question, however, whether explicit agency judgements, investigated by Schwarz et al. (2022), are also reflected in other phenomena related to the action-effect connection, such as action optimization: When participants had to judge the time of their actions and of the resulting effects, no relationship was found between intentional binding and any of Big Five personality factors (Galang et al., 2021). It is important to note that beside exploring connections between similar concepts on the levels of personality and motor control, the assessment of Big Five traits might also be relevant for the connection between schizotypy and action optimization, which is a central theme of our study. In addition to the notion that the continuum of schizotypy can be considered as a personality trait which might be present in the normal, non-clinical population (Mason, 2015; Ross et al., 2002), schizotypy was also associated with general personality dimensions. Specifically, it was demonstrated that the factors of the Big Five personality trait framework (see e.g., Digman, 1990) were not only correlated with schizotypal traits (Asai et al., 2011) and schizophrenia symptoms, but also shared a common taxonomy, suggesting that schizotypal and normal personality reflect the same underlying dimensions (Cicero et al., 2019).

Based on the literature presented above, we conducted a conceptual replication of the study by Neszmélyi and Horváth (2018) with a finer temporal resolution and larger sample. In the current study, participants were instructed to pinch a force sensitive device every 3 s while the time interval between actions and auditory feedback increased gradually, block-by-block from 0 to 560 ms in 70 ms steps. Due to this systematic progression of action-effect delays, the present paradigm provided participants an opportunity to learn the action-sound association and thus adapt to the task over time, thereby providing an upper estimate for the temporal limit of action-effect integration reflected by action-effect related motor adaptation.

We also attempted to individually characterize the duration of the window in which action-effect related motor adaptation occurred by fitting a sigmoid function on the force as a function of action-effect delay. In the study of Neszmélyi and Horváth (2018), the gradual increase of force with the action-effect delays until 200 ms was demonstrated on group-level, however, the temporal window of integration was not defined individually. In the present study, fitting a sigmoid to individual data allows the characterization of each participant with their own temporal window of integration, opening up the possibility to get a deeper insight into individual differences in motor optimization of actions. Based on this assumption, we also tested the hypothesis that this integration window will be shorter in participants with higher levels of schizotypy (as measured by the Oxford-Liverpool Inventory of Feelings and Experiences – O-LIFE-questionnaire, Mason et al., 2005, see below), suggesting that these participants rely on the sensory consequences of their actions for a shorter time. That is, we hypothesized that higher levels of schizotypy will be associated with a lower capacity to exploit delayed auditory feedback to optimize actions which might be linked to the altered perception of self-generated stimuli (Oestreich et al., 2015, 2016), changes in sense of agency (Asai & Tanno, 2007, 2008) or even with unusual perceptions (Mason, 2015; Ross et al., 2002). Because of the similar taxonomy of schizotypal and general personality dimensions, we also explored potential associations between Big Five personality traits (as measured by the Facet5 questionnaire, Buckley & Williams, 2002, see below) and the temporal patterns of action-effect related motor optimization.

## Methods

### Participants

To our knowledge no previous experiment attempted to individually characterize the size of the temporal window in which action-effect related pinch force optimization occurred, and no study investigated its potential relationship with schizotypy. Therefore, no meaningful sample size estimation could be conducted for this research question. Thus, for this research question, the present study should be regarded as explorative, and – based on the available resources – we aimed for a sample size of 120 participants completing the experiment. A sensitivity analysis based on the final sample size is presented below.

146 university students participated in the experiment for course credits. All participants reported normal or corrected-to-normal vision, normal hearing and the lack of neurological or psychiatric disorders. They gave electronic informed consent before filling in the online questionnaires, and also written informed consent before the beginning of the experimental session, after the experimental procedures were explained to them. The project was approved by the Research Ethics Committee of Károli Gáspár University of the Reformed Church in Hungary (BTK/815-1/2021). The study was conducted according to the Declaration of Helsinki.

Two participants filled out only the O-LIFE questionnaire, 23 filled both questionnaires, but did not participate in the experimental session, which left 121 participants completing the full experiment (101 women, 20 men, mean age: 21.43 years, range: 18–30 years) between February and May of 2021 (78 participants) and between September and November of 2021 (43 participants).

Five participants failed to follow the instructions, misunderstood the task, or experienced technical difficulties with the device during the experiment, which resulted in a loss of more than 50% of the actions in some of the blocks (see criteria for the rejection of actions below), so their data was not included in the analyses. For further 14 datasets, no fit of sufficient quality (*R*^*2*^ lower than 0.50, see below) – thus, no individual window size estimate – could be obtained. Thus, for exploring the potential relationships between the size of the temporal optimization window and personality factors, a sample of 102 participants was used, which comprised 85 women and 17 men, mean age: 21.36 years, range: 18–30 years. 91 participants were right-, 11 participants were left-handed. This sample size allowed the reliable detection of a correlation *r* =.27 (with a power of 1-β = 0.8 and at alpha = 0.05, using a two-tailed test; calculated through the “pwr” package in R, version 1.3-0, Champely, 2020).

Because no significant correlations were found, and because selecting by fitting quality may bias the general characterization of force application patterns, the 14 datasets mentioned above were included in the general characterization of force application patterns. Thus, datasets of 116 participants were used for a general characterization of force application patterns (95 women and 21 men, mean age: 21.34 years, range: 18–30 years. 103 participants were right-, 13 participants were left-handed). Based on data yielding the comparison with the smallest effect size in Experiment 2 of the study by Neszmélyi and Horváth (2018; i.e., the comparison between the 50-ms-delay and 100-ms-delay conditions), bootstrap resampling (with 10000 random draws) was used to estimate the necessary sample size to detect a similar difference with a power of 1-β = 0.8 at alpha = 0.05, which yielded 73 participants. The 116 participants in the present dataset provided a power of 0.944 at alpha = 0.05 to detect such an effect.

### Stimuli and procedure

The experiment had two parts. First, all participants completed questionnaires consisting of the Oxford-Liverpool Inventory of Feelings and Experiences (O-LIFE, Mason et al., 2005; Mason & Claridge, 2006; in Hungarian: Kocsis-Bogár, 2015; Kocsis-Bogár et al., 2016) and the Big Five-based Facet5 test (Buckley & Williams, 2002; McDonald & Yarker, 2016; Nagybányai Nagy, 2013) online, which was followed by the experimental session (within weeks after the questionnaires were filled). Because the exploration of the potential associations between Big Five personality traits and characteristics of action-effect related motor optimization did not yield significant results, details of the questionnaire and the exploration are reported as Supplementary Information.

#### The Oxford-liverpool inventory of feelings and experiences (O-LIFE)

We used the shortened version of The Oxford-Liverpool Inventory of Feelings and Experiences (O-LIFE), which contains 43 dichotomous (true/false) items, and is partly used to measure non-clinical, i.e., trait-level schizotypy. The O-LIFE measured four dimensions of personality: Unusual Experiences; Cognitive Disorganisation; Introvertive Anhedonia; Impulsive Nonconformity. For our hypothesis testing, two of the four dimensions of O-LIFE questionnaire were used: Unusual Experiences and Cognitive Disorganization.

#### Experimental session

The experiment took place in a separate session at the Institute of Cognitive Neuroscience and Psychology, Research Centre for Natural Sciences, Budapest. During the experiment, participants were sitting at a table in a comfortable chair. They were holding a force-sensitive resistor (FSR; FSR Model 402, Interlink Electronics, Westlake Village, CA, USA; 0.3 mm thick, circular active area with a 13 mm diameter) mounted on a thin plastic sheet. The sheet was held between the thumb and index finger of the dominant hand, with the thumb holding the sheet from above with the hand and wrist rested on the table. Participants were instructed to produce brief pinch impulses every 3 s. The FSR was calibrated to detect impulses when its signal exceeded 0.84 N after being continuously below threshold for at least 60 ms. The FSR signal was registered at 14 bits resolution with a sampling rate of 972 Hz by a Teensy 3.2 development board with an Audio Shield (PJRC.COM, Sherwood, OR, USA) connected to a personal computer. Auditory stimuli were delivered by the Audio Shield through HD-25 (Sennheiser, Wedemark, Germany) headphones with an intensity of 69 dB (sound pressure level, measured by an HSUIII.2 artificial head, Head Acoustics, Germany).

As the experiment was administered during the COVID-19 pandemic, the data collection followed the institutional safety protocol. The experimenter only met the participant in person at the very beginning and at the end of the experiment. During the experimental session they were in separate rooms and communicated via microphone. At the beginning of the experiment, two one-minute-long videos were played explaining the task instructions. In the first video, the appropriate way to pinch the FSR device was presented with narration. In the second video, the requested time interval was demonstrated by discrete vertical lines appearing at 3 s intervals on the screen. After the videos ended, a practice block followed in which participants had to produce 25 pinch impulses, which resulted in 1000 Hz sine tones of 50 ms duration (including 5 ms linear rise and fall times). Due to hardware limitations, tones were played with a delay of 2 ms – and delay times mentioned in the following do not include this extra delay. The experimenter gave feedback to the participants about their pacing performance in the practice block.

The practice block was followed by ten further blocks in fixed order for each participant. As in the practice block, participants’ task was to produce successive pinch impulses 3 s apart (3 s BAI), which resulted in the same 1000 Hz tone. However, the action-sound interval gradually increased from block to block: in the first block (0 ms block), when the FSR signal exceeded the force threshold, pinches immediately resulted in a tone; in the 2nd, 3rd, 4th, 5th, 6th, 7th, 8th and 9th blocks the tones were played with 70, 140, 210, 280, 350, 420, 490 and 560 ms delay, respectively. In the 10th block, no auditory action-effect followed the pinch impulses, that is, no tones were played (Motor block). Each block consisted of 50 pinch actions, and action-effect delay was kept constant during the blocks, maximizing the action-effect contingency. At the end of each block, a histogram with the BAI distribution was shown, and the experimenter gave oral feedback to the participants on their performance on keeping the 3 s pace, therefore participants had the opportunity to adjust their timing behavior when needed. For one participant, the order of the final two blocks was accidentally exchanged; this dataset was nonetheless included in the analyses.

Note that the fixed order of the experimental blocks might lead to order effects, which may bias the results: Neszmélyi and Horváth (2018) demonstrated that participants who were adapted to immediate action effects at the start of the experiment showed increased force in the test condition (200 ms action-effect delay) in comparison to those who adapted to 400 ms action-effect delay, suggesting that force optimization depends on prior experience. We, however, chose not to randomize the order of the action-effect delays for two reasons. First, the gradual, systematic increase of delays provides the best opportunity for participants to keep up an initially (in the 0-ms-delay block) formed integrated action-effect representation, and thus this condition order captures potential individual differences in the ability to *maintain* such connections in the face of increasing temporal separation. Second, as we aimed to examine whether performance correlated with personality, between-subject variability in stimulus presentation might have introduced additional noise potentially impacting the detectability of such correlates. In sum, although the systematic increase of delay over the presentation may have inflated the estimated temporal limit for action-effect integration, it has also provided more power to detect correlations between performance and personality scores.

It is also important to note that the experiment was designed to capture a *spontaneous* aspect of behavior – a relationship between pinch force and action-effect delay demonstrated in experiments aggregating data across participants (Neszmélyi & Horváth, 2018). Because the paradigm is largely unconstrained regarding force application (i.e., the emphasis is on action pacing performance), participants are free to pinch the device (more-or-less) as they wished, and thus individual force application patterns may not well match the previously observed group-level pattern in this paradigm. Indeed, we assume that some of the low-quality sigmoid function fits resulted from behavior unrelated to the effects of action-effect delay.

#### Behavioral data processing

**Fig. 1:**
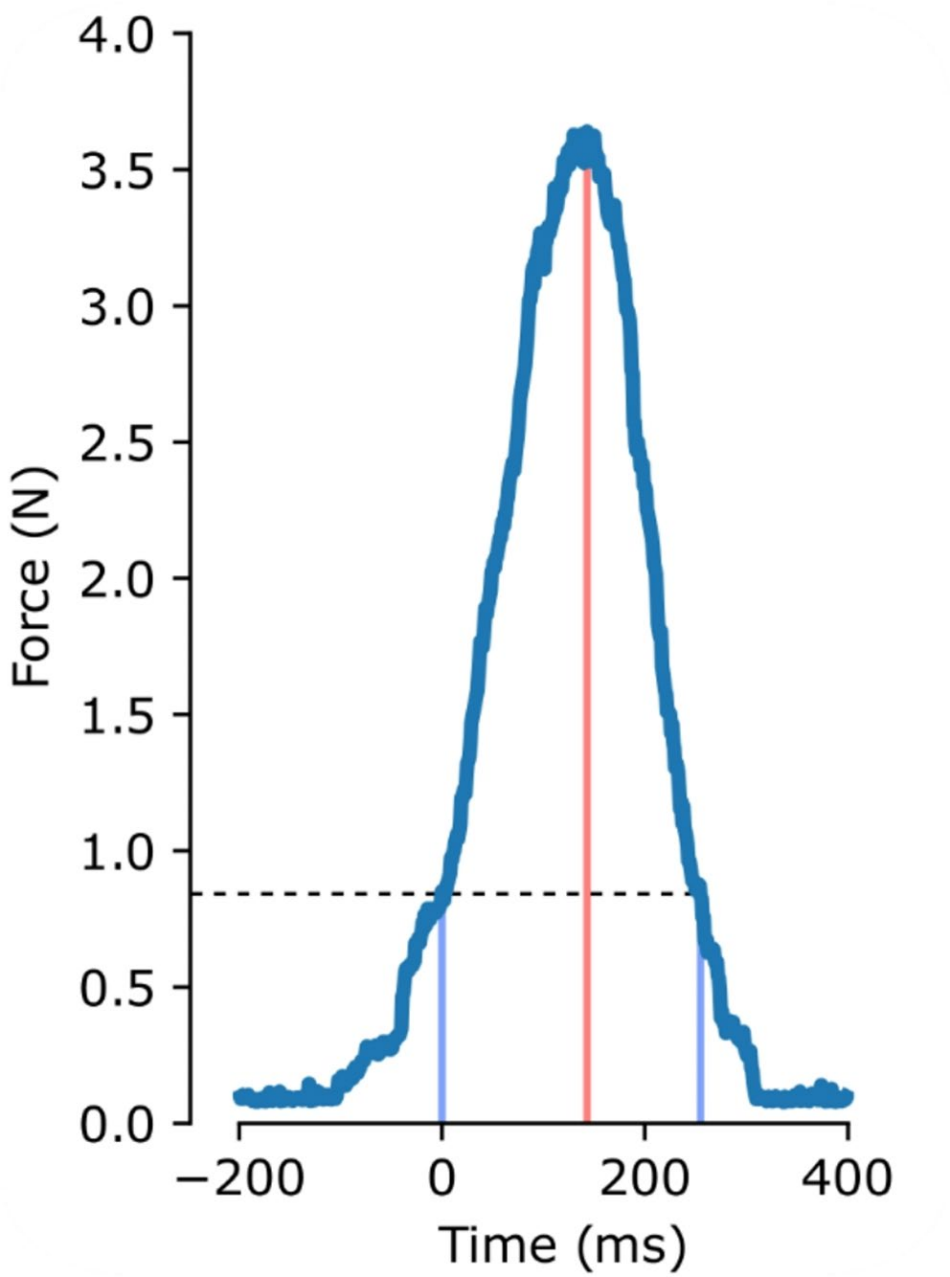
A representative force-time graph for a single action. Action onset (0 ms, indicated by a light blue vertical line) was detected when force application exceeded the pre-set force threshold of 0.84 N (shown by a dashed horizontal line), after being under the threshold for at least 60 ms. Action offset was defined as the timepoint when the force signal dropped below the threshold and stayed under it for at least 60 ms (indicated by another light blue vertical line). Peak latency is indicated by a red vertical line

The continuously recorded FSR signal was converted to force and segmented into epochs of 3 s, from − 1000 to 2000 ms relative to the timepoint when the FSR signal exceeded the threshold. As in previous studies (e.g., Neszmélyi & Horváth, 2017; Horváth et al., 2018), force application resulted in a single force peak in most cases. Each action was characterized by peak force, peak latency (by simple local peak search using the SciPy *signal.find_peaks* function) and duration (similarly to action onset, offset was defined as the timepoint when the force signal dropped below the threshold and stayed under it for at least 60 ms). Figure 1 shows the force-time graph for a representative single action. If multiple peaks were present, the highest peak was extracted. The first 10 actions of each block were not analyzed, because the first actions of the blocks may show a stepwise adaptation (Neszmélyi & Horváth, 2017). Actions were also rejected from analysis if they followed the previous action in less than 1 s or more than 6 s, or if they were followed by another action within 1 s. To characterize force application, median peak forces were calculated for the remaining actions in each block type (action-tone delay) and for each participant. Participants with blocks in which more than 50% of the actions were rejected, were excluded from further analysis. The mean, minimum and maximum number of remaining actions are presented in Table 1. To test for differences in applied force as a function of block type (action-tone delay), Friedman’s test was conducted, which was followed up by pairwise Wilcoxon signed rank tests with Holm-correction (Holm, 1979), calculated through the “coin” R-package (version 1.4.2, Hothorn et al., 2006, 2008). Effect size was characterized as the standardized test statistic (Z) divided by the square root of the number of participants (*r*; Fritz et al., 2012; Tomczak & Tomczak, 2014). To better characterize the force-delay relationship, the rate of change, that is, force differences between action-effect delays (approximating the derivative of the force-delay function) were also calculated and compared by Wilcoxon signed-rank tests with Holm-correction.

**Table 1:** Group (*N* = 116) mean, minimum and maximum number of pinches for each action-effect delay

Delay	0 ms	70 ms	140 ms	210 ms	280 ms	350 ms	420 ms	490 ms	560 ms	Motor
Mean	39.59	39.47	39.28	39.52	39.23	39.16	39.09	39.47	39.36	38.15
Min.	27	31	26	34	22	24	29	29	32	26
Max.	40	40	40	40	40	40	40	40	40	40

To investigate the relationship of personality traits and action characteristics, each participant was characterized by two parameters: (1) The size of the temporal window in which action optimization occurred, and (2) the ratio of forces measured in the 0-ms-delay and motor conditions – an index of reliance on the action-effect sound in motor adaptation.

To estimate the duration of the temporal window in which action optimization occurred, for each participant, a sigmoid curve was fitted to the median forces across the nine action-effect delays with the following formula:

$$\:f\left(t\right)=\:\frac{L}{1+\text{e}\text{x}\text{p}(-k*\left(t-t0\right))}+b$$,

where *L* denotes the range between lower and upper asymptotes, *t* the action-effect delay, *t0* the inflection point (i.e. the point where the rate of change as the function of action-effect delay – that is, the derivative of the function – is maximal), *k* the steepness of the transition, and *b* the lower asymptote of the sigmoid. The most appropriate fit was selected by the application of the curve_fit algorithm of SciPy 1.10.1 (Virtanen et al., 2020) in Python (version 3.8.1) using the Trust Region Reflective algorithm with the following bounds: *L* was bound between 0 N and the range of force of the nine action-effect delays for the given participant, *t0* was bound between 0 and 560 ms, *k* was bound to be non-negative, and the lower asymptote was bound between the minimum and maximum force among the nine action-effect delays for the given participant. To ensure proper coverage of the parameter space, the fitting algorithm was nested in a brute force grid search, with each initial parameter value selected from 10 evenly spaced values (for a total of 10000 initial value combinations) from the following ranges: For *L*, the range was between 0 N and the range of force measured for the given participant; for *t0*, the range was between 0 and 560 ms; for *k*, the range was between 0 and 0.2 ms^− 1^; and for *b* it was between the minimum and maximum force among the nine action-effect delays for the given participant. For each participant, the inflection point of the best fitting sigmoid (i.e., the one characterized by the highest *R*^*2*^) was used to characterize the size of the window in which action-effect related motor adaptation occurred. Data from participants with an *R*^*2*^ lower than 0.5 were rejected from these analyses.

In addition to the analysis of applied forces, we conducted two additional exploratory analyses on the behavioral data. First, based on the idea that sounds might serve as feedback about the successful interaction with the device (Cao et al., 2020; Varga et al., 2022), one may assume that longer action-effect delays result in longer actions because participants release their pinch on the FSR only after they hear the sound, that is, participants may tend to temporally align action offsets to tone onsets. A further possibility is that participants may tend to align the latency of force peaks to tone onsets, which fits the idea that the perceived occurrence of such actions corresponds to force peak latency (Kunde et al., 2004; Du et al., 2017). To explore these possibilities, peak latency and duration differences between adjacent delays were submitted to one-sample Wilcoxon tests with Holm-correction against the 70 ms delay differences between the corresponding conditions.

Although Neszmélyi and Horváth (2018) found that BAIs were significantly longer in the Motor condition than in any other condition, in the present study the fixed order of the conditions combined with the block-by-block feedback about pace-keeping performance (with encouragements to make adjustments in the following blocks when they deviated from the target pace) leaves potential differences open to interpretation. Nonetheless, for the sake of completeness, BAI analyses are presented in the Supplementary Materials.

#### Relationships between questionnaire and behavioral data

To test the main hypotheses, Kendall rank correlations (τ_b_) were calculated between the Unusual Experiences and Cognitive Disorganization sub-scales of the O-LIFE with inflection point latency, and force ratio of the 0-ms-delay and motor condition. Beside the correlation coefficient and the p-value, we report the Bayes factor supporting the alternative hypothesis (van Doorn et al., 2018). In contrast with the p-values provided by the more traditionally applied null hypothesis significance testing framework, Bayes Factors allow the comparison of the fit of the null- and alternative (effect) models to the data, and thus provide evidence not only for the alternative but also for the null-model (for a review see e.g., Lee & Wagenmakers, 2013). We conducted an exploratory analysis to explore the potential relationships of the two behavioral measures mentioned above with O-LIFE and Facet5 scales: in addition to the correlation analysis, separate common factor analysis was also applied. Detailed results of these analyses are presented as Supplementary Information.

Statistical analysis was run in R (R Core Team, 2023; version 4.3.2) and in IBM SPSS Statistics (version 29.0.0.0).

## Results

### Behavioral data

**Fig. 2:**
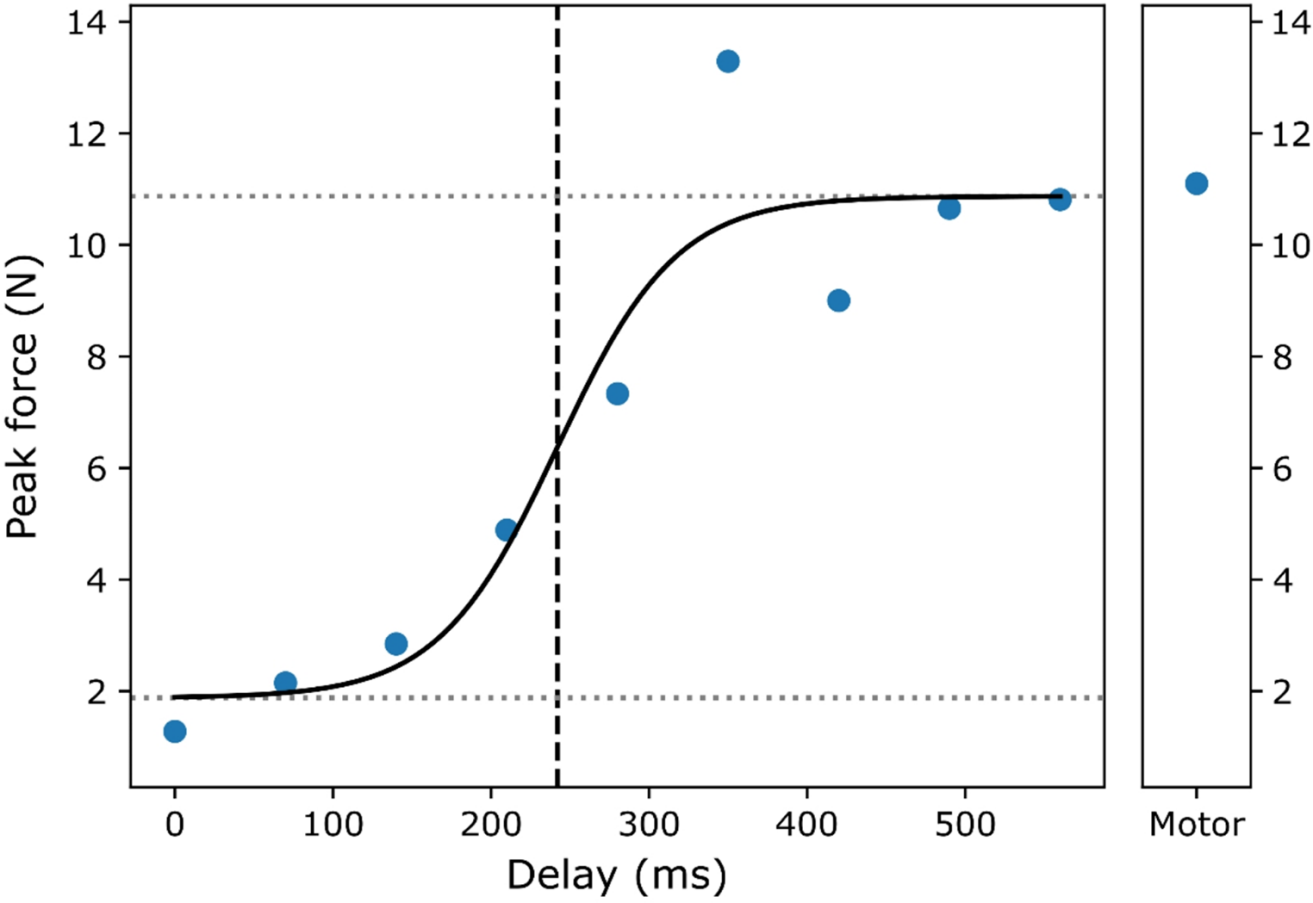
A representative individual dataset with the best sigmoid fit. Filled blue circles show the individual median peak force for the given (action-tone delay or motor) condition. Dotted horizontal lines indicate the force levels of the lower and upper asymptotes, a dashed vertical line shows the latency of the inflection point

**Fig. 3:**
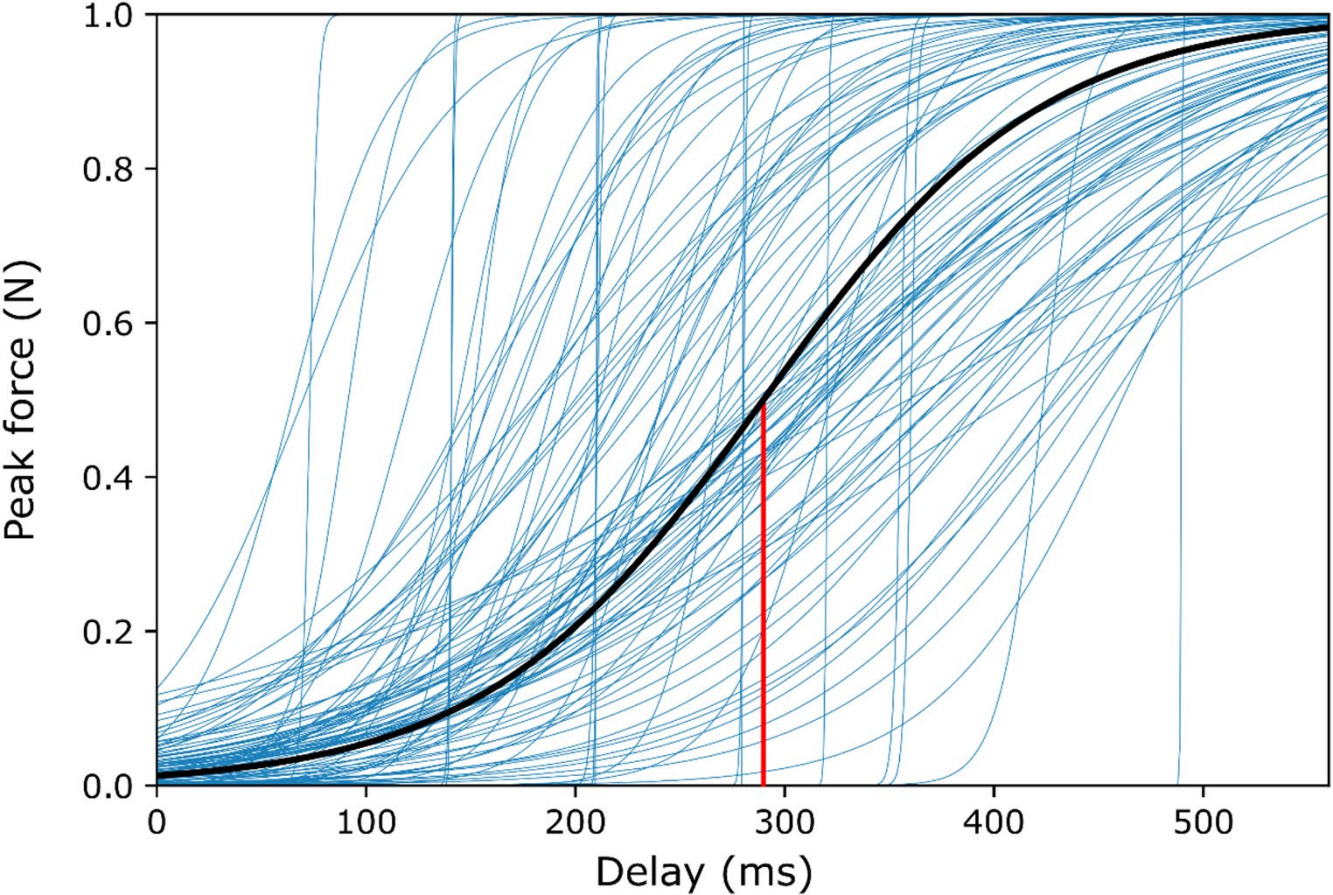
Individual sigmoid fits (thin blue lines, *N* = 102) and the sigmoid corresponding to the median fitting parameters (thick black line), with force ranges scaled to the 0–1 range. A red vertical line indicates the median latency of the inflection point (289.711 ms)

Participants complied with the instructions. Most pinches were brief force applications with a single peak (Fig. 1). BAIs were somewhat longer than the target 3 s (median: 3504 ms, range: 2918–4148 ms, *N* = 116), which might be due to the fact that the histogram showing the BAI distribution to the participants after each block had 0.5-s-wide bins around 3.0 s (i.e., columns summarizing BAI counts in the 2.5–3.0 s, and the 3.0–3.5 s range), and thus participants may have “aimed” for the upper bin. As described in the Methods section, sigmoids of sufficient quality could be fitted to 102 participants’ datasets. A representative dataset with the best fitting sigmoid function is presented in Fig. 2. Descriptive statistics of the fitted sigmoid parameters and that of the ratio of force applied in the 0-ms delay and motor conditions are presented in Table 2. Figure 3. shows the fitted individual sigmoids and the sigmoid corresponding to the mean fitting parameters, with force ranges scaled to the 0–1 range for each individual fit, as well as for the sigmoid corresponding to the mean fitting parameters. All individual datasets, fits and sigmoid parameters, as well as the histogram of R^2^ values are presented in Supplementary Material.

**Table 2:** Descriptive statistics (group median, inter-quartile range- IQR, minimum, and maximum) of the sigmoid parameters: inflection point, lower and upper asymptote, and steepness; as well as the force ratio between the 0-ms-delay and motor conditions (*N* = 102)

	Inflection point (ms)	Lower asymptote (*N*)	Upper asymptote (*N*)	Steepness (ms^− 1^)	0-ms-delay/motor force ratio
Median	289.711	1.969	10.402	0.015	0.141
IQR	126.696	1.719	11.143	0.019	0.179
Min	64.748	1.044	2.553	0.006	0.038
Max	489.498	19.896	56.069	62.056	1.137

### Questionnaires and correlation analyses

Descriptive statistics of the questionnaires are presented as Supplementary Information.

The O-LIFE Unusual Experiences sub-scale did not correlate either with the inflection point latency (*τ*_*b*_ = 0.090, *p* =.200, BF_10_ = 0.317) or the ratio of force in the 0-ms-delay and the motor condition (τ_b_ = − 0.034, *p* =.630, BF_10_ = 0.147). Similarly, the Cognitive Disorganization was also not associated with any of the force-related variables (inflection point: *τ*_*b*_ = 0.073, *p* =.294, BF_10_ = 0.233; 0-ms-delay/motor force ratio: *τ*_*b*_ = − 0.107, *p* =.126, BF_10_ = 0.452). The low (i.e., B_10_ < 1/3) Bayes Factors for correlations with the inflection point actually provide “moderate” evidence (Lee & Wagenmakers, 2013) for the null-effect. (Bayes Factors favoring the alternative model are in a reciprocal relationship with the corresponding Bayes Factor in favor of the null model – i.e. a BF_10_ of 3 corresponds to a BF_01_ of 1/3.) Scatterplots corresponding to these calculations are presented in Supplementary Material, in Figure S7.

The common factor analysis with the O-LIFE scales showed no interpretable links.

### Analyses of force exertion patterns as a function of action-effect delay

**Fig. 4:**
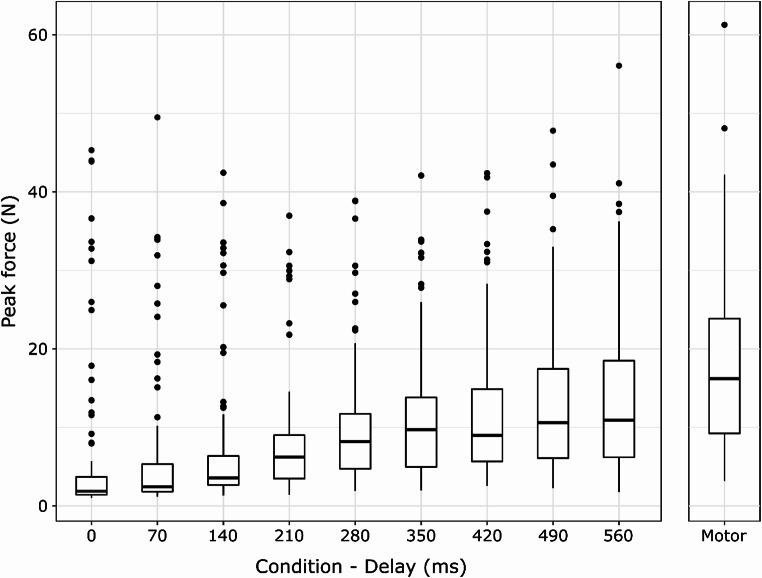
Tukey boxplots displaying the distribution of the individual median forces in different action-effect delays (*N* = 116). Note: Horizontal lines denote the median of the group, upper and lower strokes of the box indicate the 1st and 3rd quartiles of the data, and whiskers cover datapoints within the 1.5 interquartile range below and above the 1st and 3rd quartiles. Data points falling outside these bounds are shown individually

To determine whether median forces differed between conditions, Friedman’s test was applied. Results showed a significant effect of action-effect delay: *Χ*^*2*^(9) = 626.26, *p* <.001. Follow-up, Wilcoxon signed-rank tests with Holm-correction (see Table 3; Fig. 4) showed that longer action-effect delays and the absence of the auditory action effect (in the motor condition) resulted in systematically higher force in each comparison.

**Table 3:** Z standardized test statistics of pairwise Wilcoxon signed-rank comparisons (with Holm-correction) of action forces in the 9 action-effect delay and motor conditions for the whole sample (*N* = 116). Positive Z statistics mean that the force marked in the given row was higher than that in the respective column. The corresponding R effect sizes are presented in brackets

Condition	0 ms	70 ms	140 ms	210 ms	280 ms	350 ms	420 ms	490 ms	560 ms
70 ms	4.810*** (0.447)	-							
140 ms	5.595*** (0.520)	7.160*** (0.665)	-						
210 ms	6.212*** (0.577)	7.565*** (0.702)	7.135*** (0.662)	-					
280 ms	7.256*** (0.674)	7.634*** (0.709)	8.028*** (0.745)	7.477*** (0.694)	-				
350 ms	7.593*** (0.705)	7.868*** (0.731)	8.292*** (0.770)	8.110*** (0.753)	4.741*** (0.440)	-			
420 ms	7.785*** (0.723)	8.075*** (0.750)	8.207*** (0.762)	8.008*** (0.744)	6.295*** (0.584)	3.532*** (0.328)	-		
490 ms	7.703*** (0.715)	7.997*** (0.743)	8.281*** (0.769)	8.028*** (0.745)	6.887*** (0.639)	4.568*** (0.424)	3.890*** (0.361)	-	
560 ms	7.557*** (0.702)	7.915*** (0.735)	8.044*** (0.747)	7.606*** (0.706)	6.375*** (0.592)	4.747*** (0.441)	3.672*** (0.341)	2.022* (0.188)	-
Motor	8.496*** (0.789)	8.711*** (0.809)	8.774*** (0.815)	8.887*** (0.825)	8.515*** (0.791)	8.163*** (0.758)	8.072*** (0.749)	8.088*** (0.751)	7.907*** (0.734)

Note: Significance values: *p <.05, ***p <.001

**Fig. 5:**
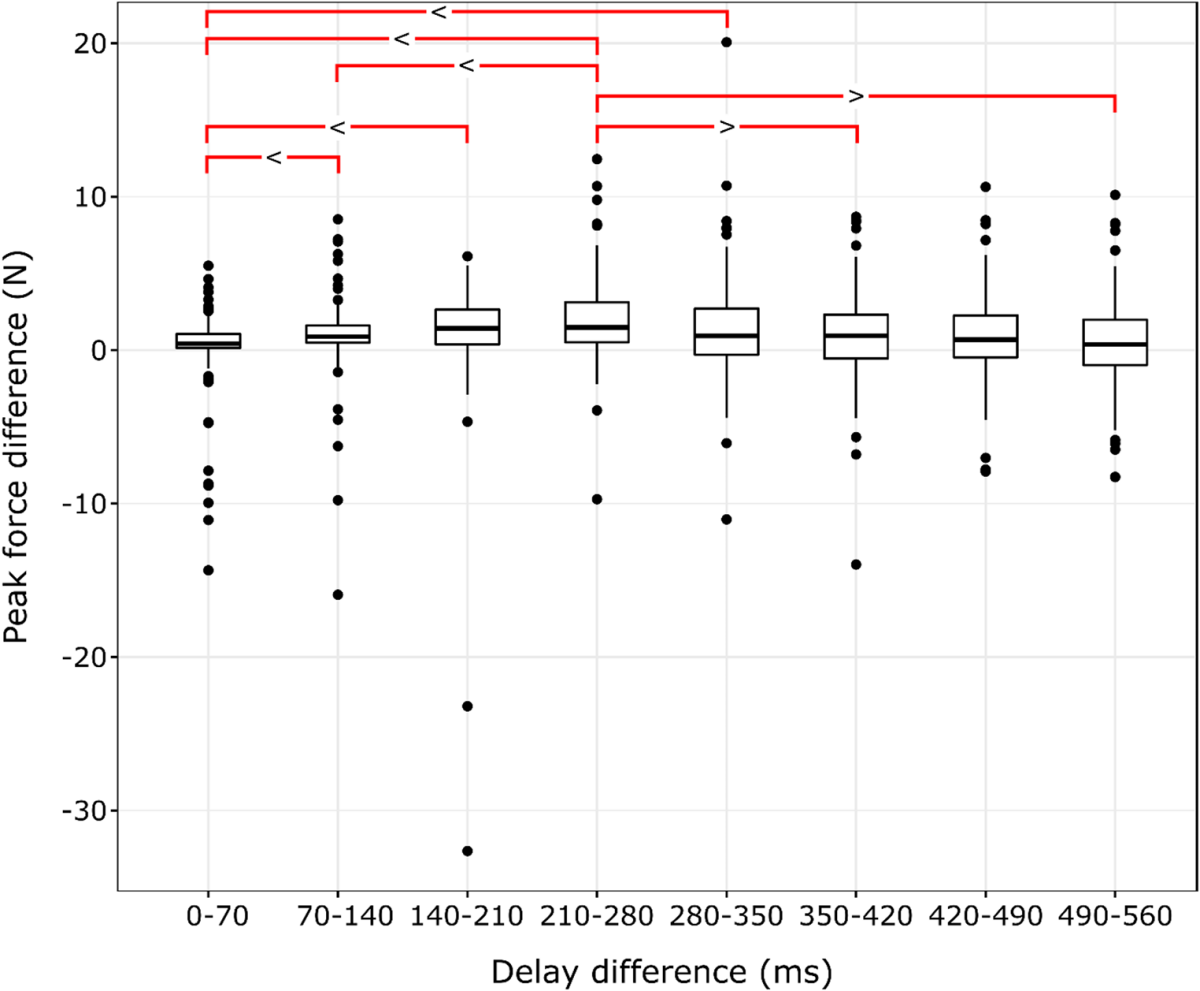
Tukey boxplots displaying the distribution of the individual median force differences between adjacent action-effect delays (*N* = 116). Significant differences described in Table 4 are shown with red lines with the direction of difference denoted by relation symbols. Note: Horizontal lines denote the median of the group, upper and lower strokes of the box indicate the 1st and 3rd quartiles of the data, and whiskers cover datapoints within the 1.5 interquartile range below and above the 1st and 3rd quartiles. Data points falling outside these bounds are shown individually

To get a better grasp on the dynamics of force increase over the range of action-effect delays, force differences between adjacent delays were submitted to pairwise Wilcoxon signed-rank tests with Holm-correction – the results are shown in Table 4, and Fig. 5. The results well match the notion that the rate of force increase as a function of action-effect is relatively low with smaller delays, reaches its maximum around 280 ms, and then gradually drops again to a lower rate – as one would expect for a sigmoid function.

**Table 4:** Z standardized test statistics of pairwise Wilcoxon signed-rank comparisons (with Holm-correction) of action forces differences between adjacent action-effect delays in the whole sample (*N* = 116). Negative Z statistics mean that the force increment marked in the given row was larger than that in the respective column. The corresponding R effect sizes are presented in brackets

Condition difference	0 vs.70 ms	70 vs. 140 ms	140 vs. 210 ms	210 vs. 280 ms	280 vs. 350 ms	350 vs. 420 ms	420 vs. 490 ms
70 vs. 140 ms	-4.298 *** **(-0.399)**	-					
140 vs. 210 ms	**-5.692 ** *** **(-0.528)**	-2.824 n.s. (-0.262)	-				
210 vs. 280 ms	**-6.480** *** **(-0.602)**	**-4.028 ** ** **(-0.374)**	-1.950 n.s. (-0.181)	-			
280 vs. 350 ms	**-3.777** ** **(-0.351)**	-0.452 n.s. (-0.042)	+ 0.749 n.s. (0.070)	+ 1.741 n.s. (0.162)	-		
350 vs. 420 ms	-2.174 n.s. (-0.202)	-0.264 n.s. (-0.025)	+ 0.912 n.s. (0.085)	**+ 3.499** * **(0.325)**	+ 0.223 n.s. (0.021)	-	
420 vs. 490 ms	-2.391 n.s. (-0.222)	-0.140 n.s. (-0.013)	+ 1.306 n.s. (0.121)	+ 2.551 n.s. (0.237)	+ 0.547 n.s. (0.051)	-0.289 n.s. (-0.027)	-
490 vs. 560 ms	-0.997 n.s. (-0.093)	+ 1.306 n.s. (0.121)	+ 2.986 n.s. (0.277)	**+ 3.906** ** **(0.363)**	+ 2.625 n.s. (0.244)	+ 1.838 n.s. (0.171)	+ 0.777 n.s. (0.072)

Note: Significance values: *p <.05,**p <.01, ***p <.001; n.s.: not significant

### Explorative analyses of peak latency and action duration as a function of action-effect delay

**Fig. 6:**
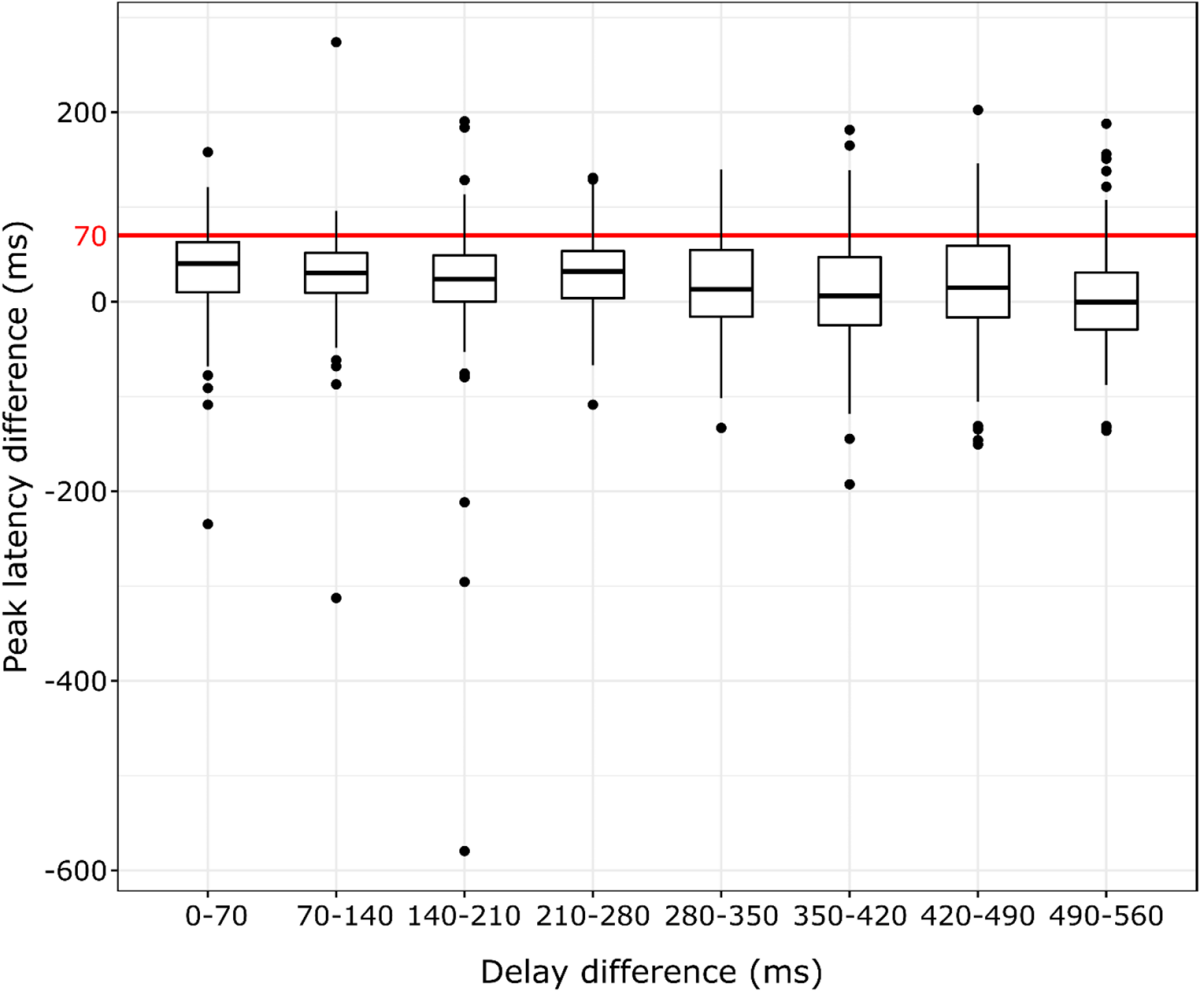
Tukey boxplots of the individual median force peak latency differences between adjacent action-effect delays (*N* = 116). A red horizontal line corresponding to the 70 ms action-effect delay difference is also shown. Note: Horizontal lines denote the median of the group, upper and lower strokes of the box indicate the 1st and 3rd quartiles of the data, and whiskers cover datapoints within the 1.5 interquartile range below and above the 1st and 3rd quartiles. Data points falling outside these bounds are shown individually

**Fig. 7:**
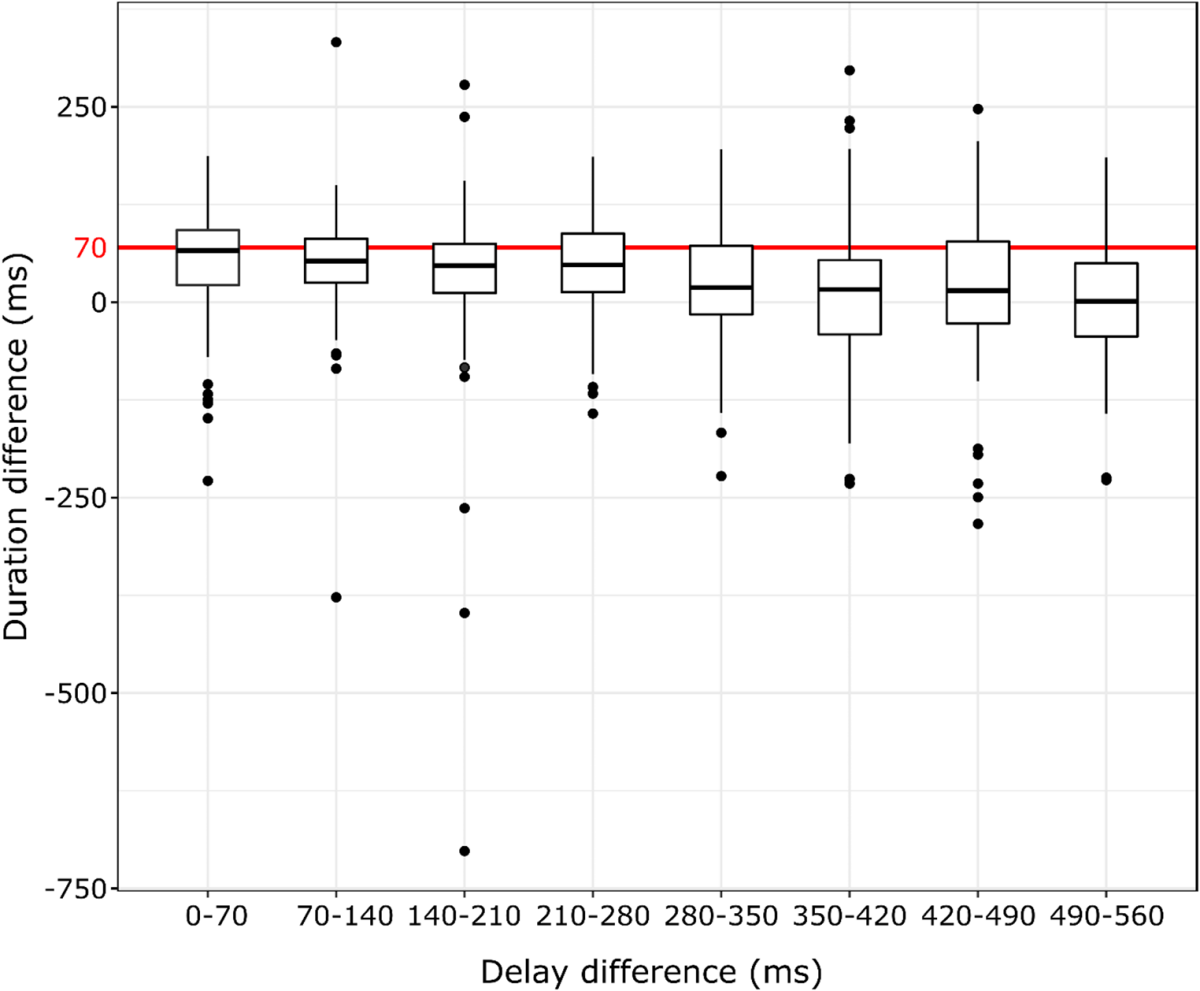
Tukey boxplots of the individual pinch duration differences between adjacent action-effect delays (*N* = 116). A red vertical line correponding to the 70 ms action-effect delay difference is also shown. Note: Horizontal lines denote the median of the group, upper and lower strokes of the box indicate the 1st and 3rd quartiles of the data, and whiskers cover datapoints within the 1.5 interquartile range below and above the 1st and 3rd quartiles. Data points falling outside these bounds are shown individually

Explorative analyses of whether peak latencies or action durations differed across conditions are presented in Supplementary Material. To investigate whether peak force latencies or durations (i.e. action offsets) followed action-effect delays – that is, whether participants systematically adjusted their force application to match force peaks or action offsets to the onsets of the elicited tones, one-sample Wilcoxon tests with Holm-correction were used: Peak latency and duration differences between adjacent delays were compared to 70 ms – the delay difference between the corresponding conditions. These showed that peak force differences, as well as duration differences between adjacent delays were significantly shorter than 70 ms in all cases (see Table 5, and 6; Figs. 6 and 7, respectively).

**Table 5:** Group median peak latency differences, the corresponding inter-quartile ranges (IQR), Z standardized test statistics, and R effect sizes for one-sample Wilcoxon tests of force peak latency differences between adjacent action-effect delays against 70 ms with Holm-correction in the whole sample (*N* = 116)

Conditions	Median peak latency difference (ms)	Peak latency difference IQR (ms)	Z	*r*
0 vs.70 ms	40.50	53.125	7.291 ***	-0.677
70 vs. 140 ms	30.50	42.000	8.609 ***	-0.799
140 vs. 210 ms	24.00	48.750	7.904 ***	-0.734
210 vs. 280 ms	32.00	49.625	7.792 ***	-0.724
280 vs. 350 ms	13.25	70.625	8.284 ***	-0.769
350 vs. 420 ms	6.25	71.625	7.763 ***	-0.721
420 vs. 490 ms	15.00	75.625	7.287 ***	-0.677
490 vs. 560 ms	-0.25	60.250	8.061 ***	-0.748

Note: Significance values: ***p <.001

**Table 6:** Group median pinch duration differences, the corresponding inter-quartile ranges (IQR), Z standardized test statistics, and R effect sizes for the one-sample Wilcoxon tests of pinch duration differences between adjacent action-effect delays against 70 ms with Holm-correction in the whole sample (*N* = 116)

Conditions	Median duration difference (ms)	Duration difference IQR (ms)	Z	*r*
0 vs.70 ms	66.00	70.250	1.970 *	0.183
70 vs. 140 ms	52.50	56.125	4.153 ***	0.386
140 vs. 210 ms	46.75	62.875	5.025 ***	0.467
210 vs. 280 ms	47.75	74.750	4.177 ***	0.388
280 vs. 350 ms	18.75	87.625	6.306 ***	0.585
350 vs. 420 ms	16.25	95.000	6.576 ***	0.611
420 vs. 490 ms	14.75	104.625	5.522 ***	0.513
490 vs. 560 ms	1.00	93.500	7.500 ***	0.696

Note: Significance values: *p <.05; ***p <.001

## Discussion

The aim of the present study was to investigate the effect of action-effect delay on motor optimization for a simple pinching action. We tested whether the size of the action-effect related motor optimization window was correlated with trait schizotypy, and explored its potential relationship with Big Five personality traits. Confirming previous studies (e.g., Horváth, 2024; Horváth et al., 2018; Neszmélyi & Horváth, 2017, 2018; Volosin & Horváth, 2022), in comparison to the motor condition, participants exerted less force when pinching the FSR elicited a sound. Our main finding is that even with 100% contingency between actions and tones, the level of force gradually increased as a function of action-effect delay, confirming results of previous studies (Cao et al., 2020; Neszmélyi & Horváth, 2018) in a larger sample, demonstrating that in similar tasks, action-effect contingency (causal action-effect relationship) on its own does not result in motor optimization – temporal contiguity is also needed (Neszmélyi & Horváth, 2018). The hypothesized correlation with schizotypy, however, was not found, in fact, Bayes Factors provided some support for the absence of correlation. Similarly, no significant correlations with Big Five personality traits were found.

Whereas Neszmélyi and Horváth (2018, Experiment 1), found no significant force differences for tone delays of 400, 800 or 1600 ms in comparison to actions with no auditory consequences, in the current study all delays (up to 560 ms) resulted in significantly lower forces than in the motor condition. This may be attributed to higher power (the sample size in Neszmélyi & Horváth, 2018 was *N* = 28), but also to the potential effect of the fixed presentation order with systematically increasing delays (whereas Neszmélyi & Horváth, 2018, used random block order) that might have allowed participants to utilize the action-contingent sounds as feedback to some degree. The force difference between the motor and the delay conditions may also indicate a strategy, or habit specifically related to the absence of stimulation in the motor condition. The motor condition differs from the delay conditions in that there is no unequivocal feedback from the device that signals that the action was registered. Because of this, participants’ strategies, habits, assumptions (or knowledge) about the operation of the device may become dominant among the processes that shape force application and may contribute more markedly than in the delay conditions (Horváth, 2024). In the present study, participants were exposed to a relatively long period of actions resulting in (delayed) tone elicitation (9 blocks: about 23 min not counting breaks), thus, they might have strongly compensated for the “sudden” lack of auditory feedback in their final experimental block.

In Experiment 2 in Neszmélyi and Horváth (2018), the authors attempted to map the delay-related force change in the 0 to 200 ms range in 50 ms steps, but failed to find a delay that could be characterized as an inflection point. In the current study the similarly fine-grained delay manipulation in the 0-560 ms range allowed the fitting of a sigmoid curve to the force data in 88% of our sample, which provided a group-mean inflection point latency of about 290 ms, with considerable variability (see Table 2). Comparisons between force differences between adjacent delay conditions (i.e. an approximation of the derivative of the force-delay function) in the whole sample (*N* = 116) provided further support for the inflection point being at around the 280 ms action-sound delay. Given the systematic, block-by-block increase of delay during the experiment, which allowed considerable time to adapt to the action-tone relationship, this estimate may well be inflated, but even so, the time interval in which action-effect related motor adaptation occurred was relatively short, which is compatible with the assumption that information on action-effect contingency has to be available within a short time to be usable for the optimization of subsequent movements. This result fits well with the idea of intentional weighting of different action effects within event-files (Memelink & Hommel, 2013): when a sensory action consequence occurs earlier, a stronger action-effect association can be formed, and the event file that includes the given consequence becomes available at an earlier timepoint during the sequence of repetitions – that is, participants can immediately rely on the sound to represent the action instead of enhancing tactile, or proprioceptive re-afference by applying more force. This temporal constraint on action optimization is also compatible with the notion that at longer intervals participants experience lower sense of agency (Rohde & Ernst, 2016; Wen, 2019), in other words, for sense of agency temporal contiguity is also required.

Given that previous studies (e.g., Horváth et al., 2018) demonstrated that action duration significantly correlated with applied force (i.e. reaching larger peak forces takes longer), one may also argue that the increase in force may reflect participants’ tendency to exert force till they hear a sound, or that they match the peak force latency to the onsets of the elicited tones. Because tactile re-afference is maximal when peak force is reached, and thus the perceptual moment of the tactile interaction may be at the peak latency (Du et al., 2017; Kunde et al., 2004), it could be argued that in the present paradigm, participants may pinch the device so that the point of maximum tactile re-afference – that is, the force peak – would be as close to the sound onset as possible. The same line of thought could be also valid for the moment when the device is actually released (action offset) as well. Such an effect could be partially driven (1) by continuous force adjustments, which have been reported in experiments (Varga et al., 2022) with randomly (50%) occurring sound action-effects. In these experiments force applications were shorter for actions with a sound effect than for actions without. The systematic presentation order of the delays in the present experiments may compel participants to pursue such a strategy by gradually increasing force. A somewhat different speculation on the mechanism driving the increase in force is related to multimodal integration. (2) Based on sensorimotor synchronization studies (Huntley et al., 2024) one may speculate that participants may extend their actions, and thus delay the perceptual moment of the tactile interaction in order to reduce the temporal separation of the tactile and the sound events, thereby furthering the formation of integrated tactile-sound events. Since these events are highly contingent, representing these as unitary events would provide a more accurately structured event representation. If participants indeed extended their actions until the appearance of the sound, the pinch duration and peak force difference between adjacent conditions would be equal to 70 ms, reflecting the experimental manipulation of the action-effect delay. Our exploratory analyses of pinch durations and peak force latencies showed however, that the between-condition difference in pinch duration or peak force latency were always smaller than the corresponding difference in delay (70 ms, see Fig. 7). That is, the observed force application pattern cannot be fully explained by participants matching peak force or offset latency to the onset of the sound.

The expected relationships between the questionnaire data and the experimental variables were not confirmed – no significant correlations with the respective scales of the O-LIFE questionnaire were found. Indeed, the Bayes Factors, which were lower than 0.33 (sometimes described as “moderate” evidence, see e.g. Lee & Wagenmakers, 2013, but see Rouder et al., 2009), provide some support for the lack of association between the level of schizotypy and the size of the time window of action optimization. This result is compatible with the notion that in contrast with action-effect contingency, the contiguity factor manipulated in the present study may not play a substantial role in schizotypy, and might also suggest that action optimization and sense of agency rely on – at least partly – different mechanisms. However, the lack of effect may also be due to the low signal-to-noise ratio provided by the procedure, as well as the uncertainty of the measuring device’s reliability reflected by poor Cronbach’s alpha values for Introvertive Anhedonia and Impulsive Nonconformity (between 0.5 and 0.6). A further reason might be that while most of the studies clearly distinguished a high and a low schizotypy group (e.g., Asai et al., 2008; Asai & Tanno, 2007; Oestreich et al., 2015, 2016; Pan et al., 2021), we utilized the O-LIFE scales’ information fully as continuous variables. Since studies demonstrating significant correlational relationship between schizotypy and cognitive domains utilize sample sizes of several hundred participants (e.g., Arzy et al., 2011; Chen et al., 1997; Lányi et al., 2024), the present study may be underpowered.

The lack of significant correlation (reported in detail in the Supplementary Material) between the size of the window for action optimization and the Big Five traits fits to the results of Galang et al. (2021) who did not find any association between any of the Big Five factors and sense of agency in a Libet clock task in a university sample similar size as in the present study (*N* = 80). In contrast, Schwarz et al. (2022) demonstrated that higher level of Neuroticism and lower level of Openness were associated with lower level of sense of agency in a much larger sample (*N* = 491). The exploratory analysis suggested that the size of the force optimization window and reliance on the sound action-effect were mostly associated with the Facet5 Affection (Agreeableness) and Control (Conscientiousness) factors, respectively. These associations suggest that force application patterns in the present paradigm could be well influenced by the social and task-related characteristics of the paradigm (see also Kiss et al., submitted). These may reflect the individual’s response to social demand characteristics (Orne, 1962, 2009) represented by the experimenter (Support, Trust and Altruism - Affection subscales) and performance-related demand characteristics implied by the task itself (Responsibility and Discipline – Control subscales). That is, participants may start applying more force at a shorter delay (i.e. at lower levels of uncertainty during the progression of the experiment), or apply more force despite the presence of reliable feedback provided by the immediately elicited sound.

To sum up, our results show that action-effect related motor adaptation occurs for simple pinching force exertions only within a short time frame with an upper estimate of about 290 ms – with consistent action-effect delays within this window, participants exert less force when their voluntary actions result in a sound. This confirms and extends previous studies (Cao et al., 2020; Neszmélyi & Horváth, 2018). However, our hypothesis about a potential relationship between this temporal limit and the schizotypy trait was not confirmed.

## Electronic supplementary material

Below is the link to the electronic supplementary material.

Supplementary Material 1

Supplementary Material 2

## Data Availability

The dataset generated and analyzed in the current study is available at 10.6084/m9.figshare.25143959.

## References

[CR1] Abram, S. V., Hua, J. P. Y., & Ford, J. M. (2022). Consider the Pons: Bridging the gap on sensory prediction abnormalities in schizophrenia. *Trends in Neurosciences*, *45*(11), 798–808. 10.1016/j.tins.2022.08.008 36123224 10.1016/j.tins.2022.08.008 PMC9588719

[CR2] American Psychiatric Association (2013). *Diagnostic and statistical manual of mental disorders* (5th ed.). 10.1176/appi.books.9780890425596

[CR3] Arzy, S., Mohr, C., Molnar-Szakacs, I., & Blanke, O. (2011). Schizotypal perceptual aberrations of time: Correlation between score, behavior and brain activity. *Plos One*, *6*(1), e16154. 10.1371/journal.pone.0016154 21267456 10.1371/journal.pone.0016154 PMC3022658

[CR4] Asai, T., Sugimori, E., Bando, N., & Tanno, Y. (2011). The hierarchic structure in schizotypy and the five-factor model of personality. *Psychiatry Research*, *185*(1–2), 78–83. 10.1016/j.psychres.2009.07.018 20537405 10.1016/j.psychres.2009.07.018

[CR5] Asai, T., Sugimori, E., & Tanno, Y. (2008). Schizotypal personality traits and prediction of one’s own movements in motor control: What causes an abnormal sense of agency? *Consciousness and Cognition*, *17*(4), 1131–1142. 10.1016/j.concog.2008.04.004 18513994 10.1016/j.concog.2008.04.004

[CR6] Asai, T., & Tanno, Y. (2007). The relationship between the sense of self-agency and schizotypal personality traits. *Journal of Motor Behavior*, *39*(3), 162–168. 17550868 10.3200/JMBR.39.3.162-168

[CR7] Asai, T., & Tanno, Y. (2008). Highly schizotypal students have a weaker sense of self-agency. *Psychiatry and Clinical Neurosciences*, *62*, 115–119. 10.1111/j.1440-1819.2007.01768.x 18289150 10.1111/j.1440-1819.2007.01768.x

[CR8] Aschersleben, G., & Prinz, W. (1997). Delayed auditory feedback in synchronization. *Journal of Motor Behavior*, *29*(1), 35–46. 10.1080/00222899709603468 20037008 10.1080/00222899709603468

[CR9] Bansal, S., Ford, J. M., & Spering, M. (2018). The function and failure of sensory predictions: Sensory prediction function and failure. *Annals of the New York Academy of Sciences*, *1426*(1), 199–220. 10.1111/nyas.13686 10.1111/nyas.13686 29683518

[CR10] Buckley, N., & Williams, R. (2002). Testing on the web - Response patterns and image management. *Selection & Development Review*, *18*, 3–8.

[CR11] Cao, L., Kunde, W., & Haendel, B. (2020). Rapid and accumulated modulation of action-effects on action. *Journal of Cognitive Neuroscience*, *32*(12), 2333–2341. 10.1162/jocn_a_01633 32985944 10.1162/jocn_a_01633

[CR12] Champely, S. (2020). *pwr: Basic Functions for Power Analysis*. R package version 1.3-0. https://CRAN.R-project.org/package=pwr

[CR13] Chen, W. J., Hsiao, C. K., & Lin, C. C. H. (1997). Schizotypy in community samples: The three-factor structure and correlation with sustained attention. *Journal of Abnormal Psychology, 106*(4), 649–657. 10.1037//0021-843x.106.4.649. 10.1037//0021-843x.106.4.649 9358696

[CR14] Cicero, D. C., Jonas, K. G., Li, K., Perlman, G., & Kotov, R. (2019). Common taxonomy of traits and symptoms: Linking schizophrenia symptoms, schizotypy, and normal personality. *Schizophrenia Bulletin*, *45*(6), 1336–1348. 10.1093/schbul/sbz005 30753725 10.1093/schbul/sbz005 PMC6811822

[CR15] Couchman, J. J., Beasley, R., & Pfordresher, P. Q. (2012). The experience of agency in sequence production with altered auditory feedback. *Consciousness and Cognition*, *21*(1), 186–203. 10.1016/j.concog.2011.10.007 22056210 10.1016/j.concog.2011.10.007

[CR16] Cullen, K. E., Brooks, J. X., & Sadeghi, S. G. (2009). How actions alter sensory processing: Reafference in the vestibular system. *Annals of the New York Academy of Sciences*, *1164*, 29–36. 10.1111/j.1749-6632.2009.03866.x 19645877 10.1111/j.1749-6632.2009.03866.x PMC3311467

[CR17] Digman, J. M. (1990). Personality structure: Emergence of the Five-Factor model. *Annual Review of Psychology*, *41*(1), 417–440. 10.1146/annurev.ps.41.020190.002221

[CR18] Du, Y., Clark, J. E., & Whitall, J. (2017). Timing at peak force May be the hidden target controlled in continuation and synchronization tapping. *Experimental Brain Research*, *235*(5), 1541–1554. 10.1007/s00221-017-4918-3 28251338 10.1007/s00221-017-4918-3

[CR21] Elijah, R. B., Pelley, L., M. E., & Whitford, T. J. (2016). Modifying Temporal expectations: Changing cortical responsivity to delayed self-initiated sensations with training. *Biological Psychology*, *120*, 88–95. 10.1016/j.biopsycho.2016.09.001 27628506 10.1016/j.biopsycho.2016.09.001

[CR19] Elsner, B., & Hommel, B. (2001). Effect anticipation and action control. *Journal of Experimental Psychology: Human Perception and Performance*, *27*(1), 229–240. 10.1037/0096-1523.27.1.229 10.1037//0096-1523.27.1.229 11248937

[CR20] Elsner, B., & Hommel, B. (2004). Contiguity and contingency in action-effect learning. *Psychological Research Psychologische Forschung*, *68*(2–3), 138–154. 10.1007/s00426-003-0151-8 14685854 10.1007/s00426-003-0151-8

[CR22] Finney, S. A. (1997). Auditory feedback and musical keyboard performance. *Music Perception*, *15*(2), 153–174. 10.2307/40285747

[CR23] Ford, J. M., Gray, M., Faustman, W. O., Roach, B. J., & Mathalon, D. H. (2007). Dissecting corollary discharge dysfunction in schizophrenia. *Psychophysiology*, *44*(4), 522–529. 10.1111/j.1469-8986.2007.00533.x 17565658 10.1111/j.1469-8986.2007.00533.x

[CR24] Ford, J. M., Palzes, V. A., Roach, B. J., & Mathalon, D. H. (2014). Did i do that? Abnormal predictive processes in schizophrenia when button pressing to deliver a tone. *Schizophrenia Bulletin*, *40*(4), 804–812. 10.1093/schbul/sbt072 23754836 10.1093/schbul/sbt072 PMC4059422

[CR25] Frith, C. D. (2014). Action, agency and responsibility. *Neuropsychologia*, *55*, 137–142. 10.1016/j.neuropsychologia.2013.09.007 24036357 10.1016/j.neuropsychologia.2013.09.007

[CR26] Frith, C. D., & Done, D. J. (1988). Towards a neuropsychology of schizophrenia. *British Journal of Psychiatry*, *153*(4), 437–443. 10.1192/bjp.153.4.437 10.1192/bjp.153.4.437 3074851

[CR27] Fritz, C. O., Morris, P. E., & Richler, J. J. (2012). Effect size estimates: Current use, calculations, and interpretation. *Journal of Experimental Psychology: General*, *141*(1), 2–18. 10.1037/a0024338 21823805 10.1037/a0024338

[CR28] Galang, C. M., Malik, R., Kinley, I., & Obhi, S. S. (2021). Studying sense of agency online: Can intentional binding be observed in uncontrolled online settings? *Consciousness and Cognition*, *95*, 103217. 10.1016/j.concog.2021.103217 34619425 10.1016/j.concog.2021.103217

[CR29] Gates, A., & Bradshaw, J. L. (1974). Effects of auditory feedback on a musical performance task. *Perception & Psychophysics*, *16*(1), 105–109. 10.3758/BF03203260

[CR30] Gates, A., Bradshaw, J. L., & Nettleton, N. C. (1974). Effect of different delayed auditory feedback intervals on a music performance task. *Perception & Psychophysics*, *15*(1), 21–25. 10.3758/BF03205822

[CR31] Haering, C., & Kiesel, A. (2015). Was it me when it happened too early? Experience of delayed effects shapes sense of agency. *Cognition*, *136*, 38–42. 10.1016/j.cognition.2014.11.012 25490127 10.1016/j.cognition.2014.11.012

[CR32] Haggard, P., & Chambon, V. (2012). Sense of agency. *Current Biology*, *22*(10), R390–R392. 10.1016/j.cub.2012.02.040 22625851 10.1016/j.cub.2012.02.040

[CR33] Haggard, P., & Eitam, B. (Eds.). (2015). *The sense of agency*. Oxford University Press. 10.1093/acprof:oso/9780190267278.001.0001

[CR34] Haggard, P., & Tsakiris, M. (2009). The experience of agency: Feelings, judgments, and responsibility. *Current Directions in Psychological Science*, *18*(4), 242–246. 10.1111/j.1467-8721.2009.01644.x

[CR35] Hirjak, D., Meyer-Lindenberg, A., Kubera, K. M., Thomann, P. A., & Wolf, R. C. (2018). Motor dysfunction as research domain in the period preceding manifest schizophrenia: A systematic review. *Neuroscience & Biobehavioral Reviews*, *87*, 87–105. 10.1016/j.neubiorev.2018.01.011 29410313 10.1016/j.neubiorev.2018.01.011

[CR39] Holm, S. (1979). A simple sequentially rejective multiple test procedure. *Scandinavian Journal of Statistics*, *6*(2), 65–70.

[CR36] Hommel, B. (2009). Action control according to TEC (theory of event coding). *Psychological Research Psychologische Forschung*, *73*(4), 512–526. 10.1007/s00426-009-0234-2 19337749 10.1007/s00426-009-0234-2 PMC2694931

[CR37] Hommel, B. (2019). Theory of event coding (TEC) V2.0: Representing and controlling perception and action. *Attention Perception & Psychophysics*, *81*(7), 2139–2154. 10.3758/s13414-019-01779-4 10.3758/s13414-019-01779-4 PMC6848055 31168699

[CR38] Hommel, B., Müsseler, J., Aschersleben, G., & Prinz, W. (2001). The theory of event coding (TEC): A framework for perception and action planning. *Behavioral and Brain Sciences*, *24*(5), 849–878. 10.1017/S0140525X01000103 12239891 10.1017/s0140525x01000103

[CR40] Horváth, J. (2015). Action-related auditory ERP Attenuation: Paradigms and hypotheses. *Brain Research*, *1626*, 54–65. 10.1016/j.brainres.2015.03.038 25843932 10.1016/j.brainres.2015.03.038

[CR41] Horváth, J. (2024). Force reflections of auditory and tactile action-effect weighting in motor planning. *Scientific Reports*, *14*(1), 18407. 10.1038/s41598-024-69444-x 39117734 10.1038/s41598-024-69444-x PMC11310450

[CR42] Horváth, J., Bíró, B., & Neszmélyi, B. (2018). Action-effect related motor adaptation in interactions with everyday devices. *Scientific Reports*, *8*(1), 6592. 10.1038/s41598-018-25161-w 29700369 10.1038/s41598-018-25161-w PMC5920059

[CR43] Hothorn, T., Hornik, K., Van De Wiel, M. A., & Zeileis, A. (2006). A Lego system for conditional inference. *The American Statistician*, *60*(3), 257–263. 10.1198/000313006X118430

[CR44] Hothorn, T., Hornik, K., Wiel, M. A. V. D., & Zeileis, A. (2008). Implementing a class of permutation tests: The coin package. *Journal of Statistical Software*, *28*(8). 10.18637/jss.v028.i08

[CR45] Hughes, G., Desantis, A., & Waszak, F. (2013). Mechanisms of intentional binding and sensory Attenuation: The role of Temporal prediction, Temporal control, identity prediction, and motor prediction. *Psychological Bulletin*, *139*(1), 133–151. 10.1037/a0028566 22612280 10.1037/a0028566

[CR46] Huntley, M. K., Nguyen, A., Albrecht, M. A., & Marinovic, W. (2024). Tactile cues are more intrinsically linked to motor timing than visual cues in visual-tactile sensorimotor synchronization. *Attention Perception & Psychophysics*, *86*(3), 1022–1037. 10.3758/s13414-023-02828-9 10.3758/s13414-023-02828-9 PMC11062975 38263510

[CR47] IBM Corp. (2021). *IBM SPSS statistics for windows, version 29.0*. IBM Corp.

[CR48] Itaguchi, Y., Sugimori, E., & Fukuzawa, K. (2018). Schizotypal traits and forearm motor control against self-other produced action in a bimanual unloading task. *Neuropsychologia*, *113*, 43–51. 10.1016/j.neuropsychologia.2018.03.034 29601887 10.1016/j.neuropsychologia.2018.03.034

[CR49] Kiss, O., Topál, J., Berkes, D., Török-Suri, K., & Horváth, J. (submitted) The impact of intranasal oxytocin on time perception and task execution when performing a simple motor task in different observational conditions. 10.1007/s00426-026-02290-w PMC13056776 41944895

[CR50] Kocsis-Bogár, K. (2015). *A szkizofrénia spektrum és a traumatikus életesemények összefüggései.* Semmelweis University (Doctoral dissertation). 10.14753/SE.2016.1912

[CR51] Kocsis-Bogár, K., Nemes, Z., & Perczel-Forintos, D. (2016). Factorial structure of the Hungarian version of Oxford-Liverpool inventory of feelings and experiences and its applicability on the schizophrenia-schizotypy continuum. *Personality and Individual Differences*, *90*, 130–136.

[CR52] Kunde, W., Koch, I., & Hoffmann, J. (2004). Anticipated action effects affect the selection, initiation, and execution of actions. *The Quarterly Journal of Experimental Psychology Section A*, *57*(1), 87–106. 10.1080/02724980343000143 10.1080/02724980343000143 14681005

[CR54] Lee, M. D., & Wagenmakers, E. J. (2013). *Bayesian cognitive modeling: A practical course*. Cambridge University Press.

[CR53] Lányi, O., Kéri, S., Pálffy, Z., & Polner, B. (2024). Can you believe your eyes? Positive schizotypy is associated with increased susceptibility to the Müller-Lyer illusion. *Schizophrenia Research*, *264*, 327–335. 10.1016/j.schres.2023.12.023 38215568 10.1016/j.schres.2023.12.023

[CR55] Luzi, N., Piani, M. C., Hubl, D., & Koenig, T. (2024). More than fulfilled expectations: An electrophysiological investigation of varying cause-effect relationships and schizotypal personality traits as related to the sense of agency. *Consciousness and Cognition*, *119*, 103667. 10.1016/j.concog.2024.103667 38428277 10.1016/j.concog.2024.103667

[CR56] Mason, O. J. (2015). The assessment of schizotypy and its clinical relevance. *Schizophrenia Bulletin*, *41*(suppl 2), S374–S385. 10.1093/schbul/sbu194 25810054 10.1093/schbul/sbu194 PMC4373636

[CR57] Mason, O. J., & Claridge, G. (2006). The Oxford-Liverpool inventory of feelings and experiences (O-LIFE): Further description and extended norms. *Schizophrenia Research*, *82*(2–3), 203–211. 16417985 10.1016/j.schres.2005.12.845

[CR58] Mason, O., Linney, Y., & Claridge, G. (2005). Short scales for measuring schizotypy. *Schizophrenia Research*, *78*(2–3), 293–296. 16054803 10.1016/j.schres.2005.06.020

[CR59] McDonald, A., & Yarker, J. (2016). *Test Review: Facet5.* The British Psychological Society, Psychological Testing Centre. http://www.facet5global.com/_literature_218922/BPS_Review_2015

[CR60] Meehl, P. E. (1962). Schizotaxia, schizotypy, schizophrenia. *American Psychologist*, *17*(12), 827–838. 10.1037/h0041029

[CR61] Meehl, P. E. (1990). Toward an integrated theory of schizotaxia, schizotypy, and schizophrenia. *Journal of Personality Disorders*, *4*(1), 1–99. 10.1521/pedi.1990.4.1.1

[CR62] Memelink, J., & Hommel, B. (2013). Intentional weighting: A basic principle in cognitive control. *Psychological Research Psychologische Forschung*, *77*(3), 249–259. 10.1007/s00426-012-0435-y 22526717 10.1007/s00426-012-0435-y PMC3627030

[CR63] Minohara, R., Wen, W., Hamasaki, S., Maeda, T., Kato, M., Yamakawa, H., Yamashita, A., & Asama, H. (2016). Strength of intentional effort enhances the sense of agency. *Frontiers in Psychology*, *7*. 10.3389/fpsyg.2016.01165 10.3389/fpsyg.2016.01165 PMC4971100 27536267

[CR64] Nagybányai Nagy, O. (2013). Online Személyiségmérés A Hazai big five struktúra Mentén: A Facet5 Teszt Magyar Adaptációja. *Pszichológia*, *33*(1), 37–59.

[CR65] Neszmélyi, B., & Horváth, J. (2017). Consequences matter: Self-induced tones are used as feedback to optimize tone-eliciting actions: Self-induced tones used as feedback for actions. *Psychophysiology*, *54*(6), 904–915. 10.1111/psyp.12845 28240775 10.1111/psyp.12845

[CR66] Neszmélyi, B., & Horváth, J. (2018). Temporal constraints in the use of auditory action effects for motor optimization. *Journal of Experimental Psychology: Human Perception and Performance*, *44*(11), 1815–1829. 10.1037/xhp0000571 30091635 10.1037/xhp0000571

[CR67] Oestreich, L. K. L., Mifsud, N. G., Ford, J. M., Roach, B. J., Mathalon, D. H., & Whitford, T. J. (2015). Subnormal sensory Attenuation to self-generated speech in schizotypy: Electrophysiological evidence for a ‘continuum of psychosis’. *International Journal of Psychophysiology*, *97*(2), 131–138. 10.1016/j.ijpsycho.2015.05.014 26027781 10.1016/j.ijpsycho.2015.05.014 PMC4607510

[CR68] Oestreich, L. K. L., Mifsud, N. G., Ford, J. M., Roach, B. J., Mathalon, D. H., & Whitford, T. J. (2016). Cortical suppression to delayed self-initiated auditory stimuli in schizotypy: Neurophysiological evidence for a continuum of psychosis. *Clinical EEG and Neuroscience*, *47*(1), 3–10. 10.1177/1550059415581708 25898988 10.1177/1550059415581708

[CR69] Orne, M. T. (1962). On the social psychology of the psychological experiment: With particular reference to demand characteristics and their implications. *American Psychologist*, *17*(11), 776–783. 10.1037/h0043424

[CR70] Orne, M. T. (2009). Demand characteristics and the concept of Quasi-Controls. In R. Rosenthal, R. L. Rosnow, & A. E. Kazdin (Eds.), *Artifacts in behavioral research* (1st ed., pp. 110–137). Oxford University Press. 10.1093/acprof:oso/9780195385540.003.0005

[CR71] Pan, C., Lu, H., Gong, J., Guo, Y., Li, Z., & Xie, P. (2021). High schizotypy conditionally have a weaker sense of agency. *Current Psychology*. 10.1007/s12144-021-01870-w

[CR72] Pfister, R., Janczyk, M., Wirth, R., Dignath, D., & Kunde, W. (2014). Thinking with portals: Revisiting kinematic cues to intention. *Cognition*, *133*(2), 464–473. 10.1016/j.cognition.2014.07.012 25156629 10.1016/j.cognition.2014.07.012

[CR73] Pfister, R., Neszmélyi, B., & Kunde, W. (2023). Response durations: A flexible, no-cost tool for psychological science. *Current Directions in Psychological Science*, *32*(2), 160–166. 10.1177/09637214221141692

[CR75] Pfordresher, P., & Palmer, C. (2002). Effects of delayed auditory feedback on timing of music performance. *Psychological Research Psychologische Forschung*, *66*(1), 71–79. 10.1007/s004260100075 11963280 10.1007/s004260100075

[CR74] Pfordresher, P. Q., & Dalla Bella, S. (2011). Delayed auditory feedback and movement. *Journal of Experimental Psychology: Human Perception and Performance*, *37*(2), 566–579. 10.1037/a0021487 21463087 10.1037/a0021487

[CR76] Pfordresher, P. Q., & Palmer, C. (2006). Effects of hearing the past, present, or future during music performance. *Perception & Psychophysics*, *68*(3), 362–376. 10.3758/BF03193683 16900830 10.3758/bf03193683

[CR77] R Core Team (2023). R: A language and environment for statistical computing. R Foundation for Statistical Computing, Vienna, Austria. https://www.R-project.org/

[CR78] Rohde, M., & Ernst, M. O. (2016). Time, agency, and sensory feedback delays during action. *Current Opinion in Behavioral Sciences*, *8*, 193–199. 10.1016/j.cobeha.2016.02.029

[CR79] Ross, S. R., Lutz, C. J., & Bailley, S. E. (2002). Positive and negative symptoms of schizotypy and the five-factor model: A domain and facet level analysis. *Journal of Personality Assessment*, *79*(1), 53–72. 10.1207/S15327752JPA7901_04 12227668 10.1207/S15327752JPA7901_04

[CR80] Rouder, J. N., Speckman, P. L., Sun, D., Morey, R. D., & Iverson, G. (2009). Bayesian t tests for accepting and rejecting the null hypothesis. *Psychonomic Bulletin & Review*, *16*(2), 225–237. 10.3758/PBR.16.2.225 19293088 10.3758/PBR.16.2.225

[CR81] Schwarz, K. A., Klaffehn, A. L., Hauke-Forman, N., Muth, F. V., & Pfister, R. (2022). Never run a changing system: Action-effect contingency shapes prospective agency. *Cognition*, *229*, 105250. 10.1016/j.cognition.2022.105250 35963118 10.1016/j.cognition.2022.105250

[CR82] Tomczak, M., & Tomczak, E. (2014). The need to report effect size estimates revisited. An overview of some recommended measures of effect size. *Trends in Sport Sciences*, *1*(21), 19–25.

[CR84] Van Doorn, J., Ly, A., Marsman, M., & Wagenmakers, E. J. (2018). Bayesian inference for Kendall’s rank correlation coefficient. *The American Statistician*, *72*(4), 303–308. 10.1080/00031305.2016.1264998

[CR85] Varga, S., Neszmélyi, B., Hajdú, N., & Horváth, J. (2022). The emergence of action-effect-related motor adaptation amidst outcome unpredictability. *Journal of Experimental Psychology: Human Perception and Performance*, *48*(7), 711–723. 10.1037/xhp0001021 35587439 10.1037/xhp0001021

[CR92] Varga,S., Pfister, R., Neszmélyi, B., Kunde, W., & Horváth, J. (2024). Task-relevance and change detection in action-effect binding. *ActaPsychologica, 243*, 104147. 10.1016/j.actpsy.2024.104147 38237474 10.1016/j.actpsy.2024.104147

[CR83] Virtanen, P., Gommers, R., Oliphant, T. E., Haberland, M., Reddy, T., Cournapeau, D., Burovski, E., Peterson, P., Weckesser, W., Bright, J., van der Walt, S. J., Brett, M., Wilson, J., Millman, K. J., Mayorov, N., Nelson, A. R. J., Jones, E., Kern, R., Larson, E., & Vázquez-Baeza, Y. (2020). SciPy 1.0: Fundamental algorithms for scientific computing in python. *Nature Methods*, *17*(3), 261–272. 10.1038/s41592-019-0686-2 32015543 10.1038/s41592-019-0686-2 PMC7056644

[CR86] Volosin, M., & Horváth, J. (2022). Force and electromyography reflections of sensory action-effect weighting during pinching. *Human Movement Science*, *84*, 102969. 10.1016/j.humov.2022.102969 35704968 10.1016/j.humov.2022.102969

[CR87] Weiskrantz, L., Elliott, J., & Darlington, C. (1971). Preliminary observations on tickling oneself. *Nature*, *230*(5296), 598–599. 10.1038/230598a0 4928671 10.1038/230598a0

[CR88] Wen, W. (2019). Does delay in feedback diminish sense of agency? A review. *Consciousness and Cognition*, *73*, 102759. 10.1016/j.concog.2019.05.007 31173998 10.1016/j.concog.2019.05.007

[CR89] Whitford, T. J. (2019). Speaking-induced suppression of the auditory cortex in humans and its relevance to schizophrenia. *Biological Psychiatry: Cognitive Neuroscience and Neuroimaging*, *4*(9), 791–804. 10.1016/j.bpsc.2019.05.011 31399393 10.1016/j.bpsc.2019.05.011

[CR90] Whitford, T. J., Mathalon, D. H., Shenton, M. E., Roach, B. J., Bammer, R., Adcock, R. A., Bouix, S., Kubicki, M., De Siebenthal, J., Rausch, A. C., Schneiderman, J. S., & Ford, J. M. (2011). Electrophysiological and diffusion tensor imaging evidence of delayed corollary discharges in patients with schizophrenia. *Psychological Medicine*, *41*(5), 959–969. 10.1017/S0033291710001376 20663254 10.1017/S0033291710001376 PMC3807011

