# Supplementary material for: An upper temporal limit of action-effect integration as reflected by motor adaptation
Source: Psychol Res. 2025 Apr 23;89(3):94. doi: 10.1007/s00426-025-02121-4 (PMC12014811; doi:10.1007/s00426-025-02121-4)
Supplement: Supplementary file 1 — Supplementary Material 1 [file 426_2025_2121_MOESM1_ESM.docx]

**Supplementary Material**

An upper temporal limit of action-effect integration
as reflected by motor adaptation

Márta Volosin, Olivér Nagybányai Nagy, Bence Neszmélyi, János Horváth

1. **Histogram of R^2^ values**

***Figure S1.*** *Histogram of the R^2^ values (N=116).*


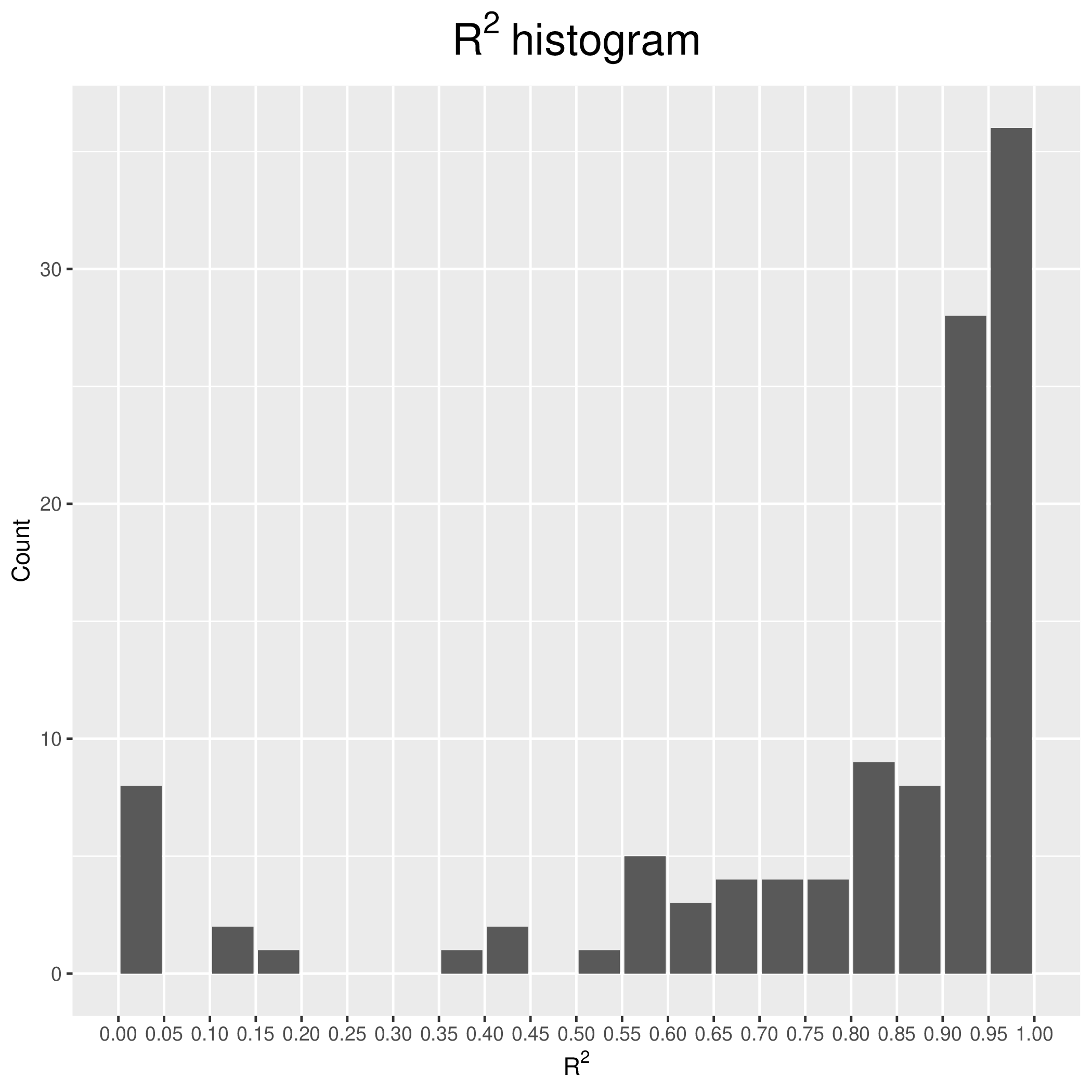


1. **Force peak latency analyses**

For the explorative analyses of the effect of condition on force peak latency, Friedman’s test showed a significant effect: *Χ^2^*(9) = 401.53, *p* < .001. Follow-up Wilcoxon signed-rank tests with Holm-correction (Table S1, and Figure S2) showed that force peak latency significantly increased with increasing action-effect delay up to 490 ms. Significant differences between the motor and tone conditions were found in the 0-280 ms range only.

***Table S1.***

*Z standardized test statistics of pairwise Wilcoxon signed-rank comparisons (with Holm-correction) of force peak latencies in the 9 action-effect delay and motor conditions for the whole sample (N=116). Positive Z statistics mean that the peak latency marked in the given row was longer than that in the respective column. The corresponding r effect sizes are presented in brackets.*

| Condition | 0 ms | 70 ms | 140 ms | 210 ms | 280 ms | 350 ms | 420 ms | 490 ms | 560 ms |
| --- | --- | --- | --- | --- | --- | --- | --- | --- | --- |
| 70 ms | 7.017 *** (.651) | - |  |  |  |  |  |  |  |
| 140 ms | 7.591 *** (.705) | 7.178 *** (.666) | - |  |  |  |  |  |  |
| 210 ms | 7.204 *** (.669) | 6.641 *** (.617) | 5.405 *** (.502) | - |  |  |  |  |  |
| 280 ms | 7.643 *** (.710) | 7.228 *** (.671) | 7.083 *** (.658) | 6.485 *** (.602) | - |  |  |  |  |
| 350 ms | 7.710 *** (.716) | 7.460 *** (.693) | 7.108 *** (.660) | 6.408 *** (.595) | 3.364 ** (.312) | - |  |  |  |
| 420 ms | 7.875 *** (.731) | 7.397 *** (.687) | 6.893 *** (.640) | 6.109 *** (.567) | 3.565 ** (.331) | 1.743 n.s. (.162) | - |  |  |
| 490 ms | 7.991 *** (.742) | 7.575 *** (.703) | 7.105 *** (.660) | 6.577 *** (.611) | 4.739 *** (.440) | 3.415 ** (.317) | 3.145 * (.292) | - |  |
| 560 ms | 8.126 *** (.754) | 7.524 *** (.699) | 6.668 *** (.619) | 6.393 *** (.594) | 4.153 ** (.386) | 2.985 * (.277) | 3.239 * (.301) | 0.439 n.s. (.041) | - |
| Motor | 7.945 *** (.738) | 7.401 *** (.687) | 6.789 *** (.630) | 5.929 *** (.550) | 3.535 ** (.328) | 2.278 n.s. (.212) | 0.787 n.s. (.073) | -0.986 n.s.  (-.092) | -0.606 n.s. (-.056) |

Note: Significance values: *p < .05, **p < .01, ***p < .001, n.s.: not significant

***Figure S2.***

*Tukey boxplots displaying the distribution of the individual median peak latencies for different action-effect delays and the motor condition (N=116).*


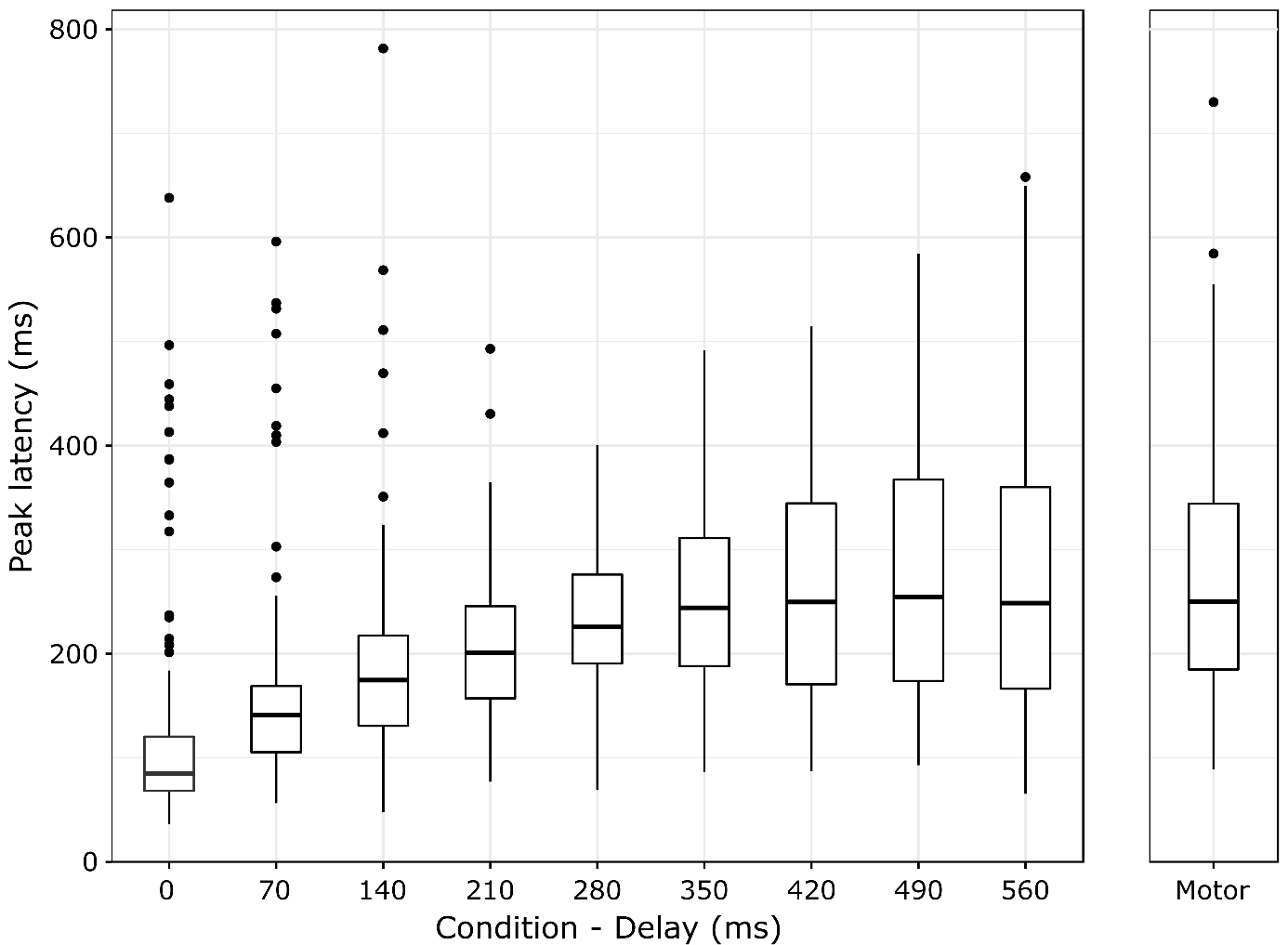


Note: Horizontal lines denote the median of the group, upper and lower strokes of the box indicate the 1^st^ and 3^rd^ quartiles of the data, and whiskers cover datapoints within the 1.5 interquartile range below and above the 1^st^ and 3^rd^ quartiles. Data points falling outside these bounds are shown individually.

1. **Pinch duration analyses**

For the explorative analyses of the effect of condition on pinch duration, Friedman’s test showed a significant effect: *Χ^2^*(9) = 401.53, *p* < .001. Follow-up Wilcoxon signed-rank tests with Holm-correction (Table S2, and Figure S3) showed that duration was significantly longer for longer action-effect delays in all comparisons up to 350 ms, with some comparisons yielding null effects above that. Significant differences between the motor and tone conditions were found in the 0-350 ms range only.

***Table S2.***

*Z standardized test statistics of pairwise Wilcoxon signed-rank comparisons (with Holm-correction) of pich durations in the 9 action-effect delay and motor conditions for the whole sample (N=116). Positive Z statistics mean that the pinch duration marked in the given row was longer than that in the respective column. The corresponding r effect sizes are presented in brackets.*

| Condition | 0 ms | 70 ms | 140 ms | 210 ms | 280 ms | 350 ms | 420 ms | 490 ms | 560 ms |
| --- | --- | --- | --- | --- | --- | --- | --- | --- | --- |
| 70 ms | 6.896  ***  (.640) | - |  |  |  |  |  |  |  |
| 140 ms | 7.821  *** (.726) | 7.768  *** (.721) | - |  |  |  |  |  |  |
| 210 ms | 7.493  *** (.696) | 7.290  *** (.677) | 6.043  *** (.561) |  |  |  |  |  |  |
| 280 ms | 8.046  *** (.747) | 7.544  *** (.700) | 7.161  *** (.665) | 6.902  *** (.641) | - |  |  |  |  |
| 350 ms | 7.868  *** (.731) | 7.733  *** (.718) | 7.210  *** (.669) | 6.500  *** (.604) | 3.621  **  (.336) | - |  |  |  |
| 420 ms | 7.952  *** (.738) | 7.569  *** (.703) | 6.843  *** (.635) | 5.761  *** (.535) | 3.207  *  (.298) | 1.518  n.s.  (.141) | - |  |  |
| 490 ms | 8.086  *** (.751) | 7.762  *** (.721) | 7.112  *** (.660) | 6.452  *** (.599) | 4.233  ***  (.393) | 2.931  *  (.272) | 3.252  * (.302) | - |  |
| 560 ms | 8.167  *** (.758) | 7.637  *** (.709) | 6.517  *** (.605) | 5.884  *** (.546) | 3.546  **  (.329) | 2.226  n.s.  (.207) | 2.875  *  (.267) | 0.101  n.s.  (.009) | - |
| Motor | 8.241  *** (.765) | 7.831  *** (.727) | 7.047  *** (.654) | 6.354  *** (.590) | 4.057  ***  (.377) | 2.800  *  (.260) | 1.562  n.s.  (.145) | 0.058  n.s.  (.005) | 0.388  n.s.  (.036) |

Note: Significance values: *p < .05, **p < .01, ***p < .001, n.s.: not significant

***Figure S3.***

*Tukey boxplots of the individual median pinch durations for different action-effect delays and the motor condition (N=116).*


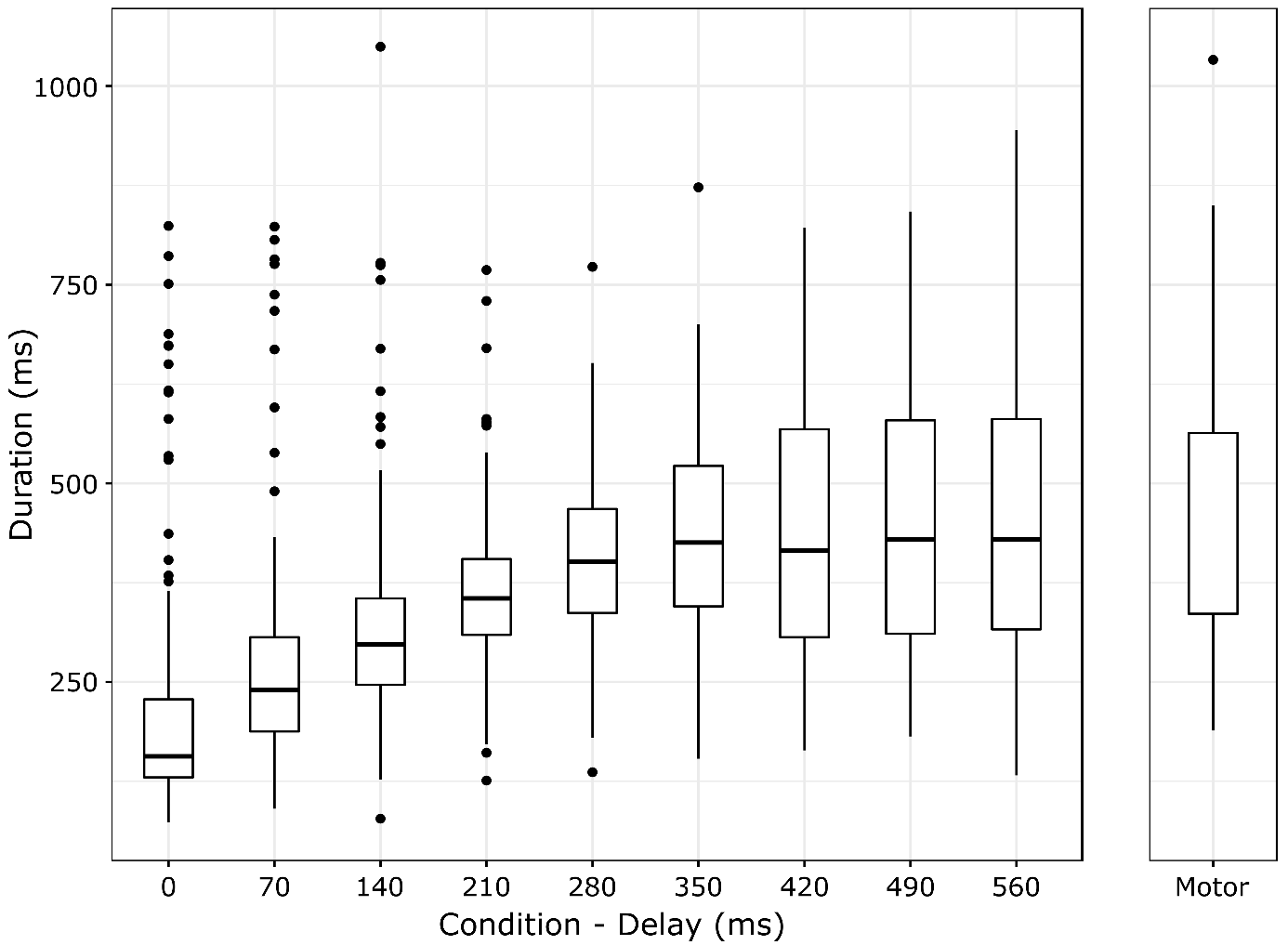


Note: Horizontal lines denote the median of the group, upper and lower strokes of the box indicate the 1^st^ and 3^rd^ quartiles of the data, and whiskers cover datapoints within the 1.5 interquartile range below and above the 1^st^ and 3^rd^ quartiles. Data points falling outside these bounds are shown individually.

1. **Between-action intervals (BAI)**

Participants were characterized by the median of BAIs in each condition. Friedman’s test showed a significant effect of condition: *Χ^2^*(9) = 53.749, *p* < .001. Follow-up, pairwise Wilcoxon signed-rank tests with Holm-correction (Table S3, and Figure S4) showed significantly shorter BAIs for the (first presented) 0-ms-delay condition in several comparisons, and the 140-ms-delay condition in some comparisons (vs. 490 ms delay, and the Motor condition). The relatively shorter BAIs in first (0-ms-delay) condition, and third (140-ms-delay) condition likely reflect the initial dynamics of the interval production task driven by the fixed presentation order and between-block feedback – that is, an initial undershoot which is compensated in the second block and then a slight undershoot again in the third (140-ms-delay) block.

***Table S3.***

*Z standardized test statistics of pairwise Wilcoxon signed-rank comparisons (with Holm-Bonferroni correction) of between action intervals (BAIs) in the 9 action-effect delay and motor conditions for the whole sample (N=116). Positive Z values mean that the BAI marked in the given row was longer than that in the respective column. The corresponding r effect sizes are presented in brackets.*

| Condition | 0 ms | 70 ms | 140 ms | 210 ms | 280 ms | 350 ms | 420 ms | 490 ms | 560 ms |
| --- | --- | --- | --- | --- | --- | --- | --- | --- | --- |
| 70 ms | **3.266**  *** (.303)** | - |  |  |  |  |  |  |  |
| 140 ms | 2.375  n.s.  (.220) | -0.616 (-.057) | - |  |  |  |  |  |  |
| 210 ms | 3.149  n.s.  (.292) | 0.185  n.s.  (.017) | 1.981  n.s.  (.184) | - |  |  |  |  |  |
| 280 ms | **4.422**  *******  **(.411)** | 1.068  n.s.  (.099) | 2.876  n.s.  (.267) | 1.528  n.s. (.142) | - |  |  |  |  |
| 350 ms | **3.508**  *****  **(.326)** | 0.674  n.s.  (.063) | 2.561  n.s.  (.238) | 1.562 n.s.  (.145) | -0.391  n.s.  (-.036) | - |  |  |  |
| 420 ms | **4.160**  ******  **(.386)** | 1.160  n.s.  (.108) | 3.106  n.s.  (.288) | 1.512  n.s.  (.140) | 0.044  n.s.  (.004) | 0.657  n.s.  (.061) | - |  |  |
| 490 ms | **4.372**  *******  **(.406)** | 1.581  n.s.  (.147) | **3.532**  *****  **(.328)** | 3.068  n.s.  (.285) | 1.315  n.s.  (.122) | 2.018  n.s.  (.187) | 2.123  n.s.  (.197) | - |  |
| 560 ms | **4.276**  *******  **(.397)** | 1.594  n.s.  (.148) | 3.145  n.s.  (.292) | 2.160  n.s.  (.201) | 0.891  n.s.  (.083) | 1.488  n.s.  (.138) | 1.459  n.s.  (.135) | -0.358  n.s.  (-.033) | - |
| Motor | **4.081**  ******  **(.379)** | 1.860  n.s.  (.173) | **3.953**  ******  **(.367)** | 2.824  n.s.  (.262) | 1.879  n.s.  (.174) | 2.171  n.s.  (.202) | 1.782  n.s.  (.165) | 0.501  n.s.  (.047) | 0.846  n.s.  (.079) |

Note: Significance values: *p < .05, **p < .01, ***p < .001, n.s.: not significant

***Figure S4.***

*Tukey boxplots of the individual median between action intervals (BAIs) for different action-effect delays and the motor condition (N=116).*


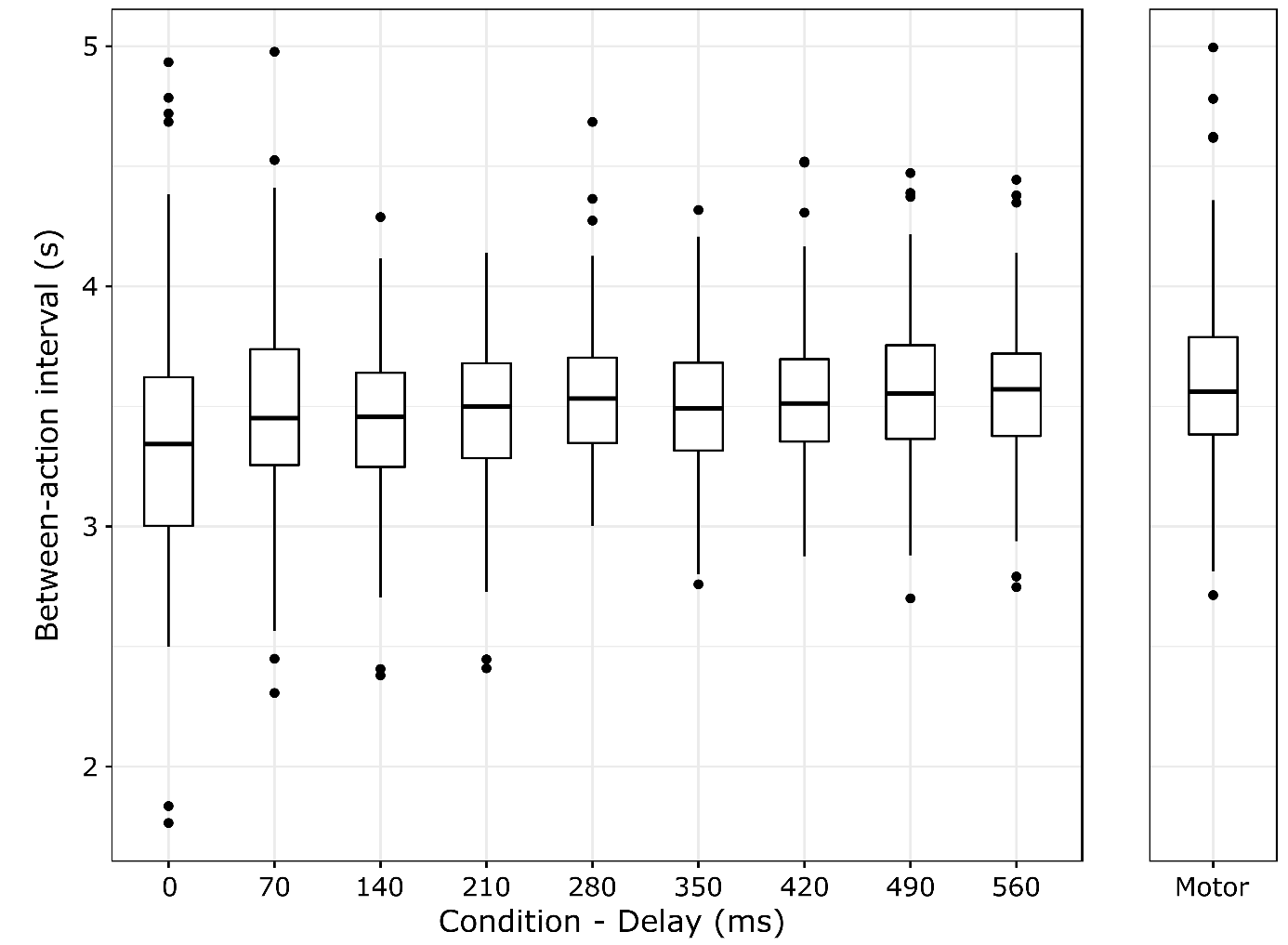


Note: Horizontal lines denote the median of the group, upper and lower strokes of the box indicate the 1^st^ and 3^rd^ quartiles of the data, and whiskers cover datapoints within the 1.5 interquartile range below and above the 1^st^ and 3^rd^ quartiles. Data points falling outside these bounds are shown individually.

1. **The Big Five-based Facet5**

The Big Five-based Facet5 test (Buckley & Williams, 2002; McDonald & Yarker, 2016; Nagybányai Nagy, 2013) measured 13 subscales in addition to the five basic factors. By assessing the Big Five personality traits, we exploratively searched for associations with the dynamics of action-affect-related motor optimization.

The Facet5 questionnaire consists of 106 pairs of statements. The items contain pairs of statements formulated as opposite endpoints, between which the respondent must choose on a five-point response scale. The middle choice in the response, i.e., a response of 3, can be interpreted as a lack of choice between the two opposing sides. This response style is equivalent to avoiding a choice, indicating a kind of omission, not a choice, in the test. Therefore, in order to reduce this type of low-informative response, the Facet5 questionnaire instructions warn the respondent to avoid these meaningless 3s when answering.

The naming of the dimensions measured by Facet5 (Buckley & Williams, 2002) is somewhat different from the traditional Big Five factor names. However, these differences do not imply differences in content. In our own research, the use of the Facet5 for personality measurement seems adequate compared to other Big Five-based measures because this personality test is well aligned with the results of previous Hungarian personality taxonomy analyses (Nagybányai Nagy, 2013) and has a large sample of Hungarian standards. In the Facet5 test, Extraversion is measured by the scale named Energy, which covers the topics of sociability, vitality, communicativeness and the ability to create connections. Conscientiousness is measured by the Control scale, which describes the internal need to conform to rules and the urge to conform at item level. The Emotionality scale is the reverse of the Neuroticism factor (i.e., emotional stability instead of emotional instability, but with the same content as the original Big Five factor). The last two scales, however, are somewhat specialized compared to the terminology of most Anglo-Saxon Big Five tests. (However, there are also some well-known measures with similar logic, such as the HEXACO – Lee & Ashton, 2004) The content of the Will and Affection dimensions of the Facet5 test is in line with Hungarian factor analytic studies (Szirmák, & De Raad, [1994](#_ENREF_29)). In this personality taxonomy research, a decomposition of the original Agreeableness factor into a narrower Agreeableness factor and a new Integrity, Honesty factor was observed. In addition, the research results showed that the Openness factor, which is common in the Anglo-Saxon factor structure, did not manifest as a separate domain. In other words, in the Hungarian trait taxonomy an independent factor can be distinguished, which, based on the marker words of its endpoints, can be called "positive attitude towards others" – this is included in the Facet5 test as the Affection scale, covering the themes of altruism, support, trust. In addition, a separate dimension combining the sub-themes of determination (vs uncertainty), confrontation (vs willingness to compromise) and independence (vs cooperation) appears as a separate factor, which is called Will in the Facet5 test.

1. **Descriptive statistics of questionnaire data**

The internal consistency of the Facet5 according to large-scale English and Hungarian psychometric analyses (McDonald & Yarker, 2016; Nagybányai Nagy, 2013), for all five basic dimensions is high, with Cronbach-alpha values around 0.7-0.8. For O-LIFE so far, however, reliability and norm average results are somewhat diverging, therefore we highlight the results of our own sample among other Hungarian studies. For example, in the study of Kocsis-Bogár (2015), 7 items were dropped from the questionnaire to better fit the confirmatory factor analysis, but we used the original shortened O-LIFE 43-item structure in the present study (Table S4).

***Table S4.***

*Cronbach-alpha and sample size (N) of O-LIFE scales in previous studies and the current study*

| Scale | Mason et al. (2005) | Kocsis-Bogár  (2015) | Kocsis-Bogár et al. (2016) | Fejes et al.  (2018) | Current study |
| --- | --- | --- | --- | --- | --- |
| Unusual Experiences | 0.80 | 0.72 | 0.72 | 0.58 | 0.67 |
| Cognitive Disorganisation | 0.77 | 0.83 | 0.83 | 0.73 | 0.79 |
| Introvertive Anhedonia | 0.62 | 0.69 | 0.69 | 0.54 | 0.55 |
| Impulsive Nonconformity | 0.63 | 0.54 | 0.54 | 0.62 | 0.53 |
| N | 928 | 131 | 380 | 57 | 146 |

The means and variances of the O-LIFE-scales for our own sample (N = 146) are shown in Table S5. and Figure S5., compared to similar results from previous studies. This shows that the O-LIFE averages from our own sample show significant differences for several dimensions compared to the results of the available national small-sample, shortened test version (Fejes et al., 2018; Kocsis-Bogár, 2015; Kocsis-Bogár et al., 2016).

***Table S5.***

*O-LIFE means and standard deviations as well as sample sizes in different studies*

|  | Kocsis-Bogár  (2015) | Kocsis-Bogár et al. (2016) | Fejes et al. (2018) | Current study |
| --- | --- | --- | --- | --- |
| Unusual Experiences (UE) | 2.57 (2.48) | 3.74 (2.29) | 2.93 (1.86) | 4.44 (2.55) |
| Cognitive Disorganisation (CD) | 4.33 (3.47) | 4.22 (2.96) | 5.23 (2.75) | 5.84 (3.03) |
| Introvertive Anhedonia (IA) | 2.43 (1.82) | 2.31 (2.40) | 1.04 (1.43) | 2.77 (1.70) |
| Impulsive Nonconformity (IN) | 1.18 (1.32) | 2.62 (1.70) | 2.05 (181) | 2.75 (1.82) |
| N | 131 | 380 | 57 | 146 |

***Figure S5.***

*O-LIFE means (and 95% confidence intervals indicated by whiskers) in different studies*


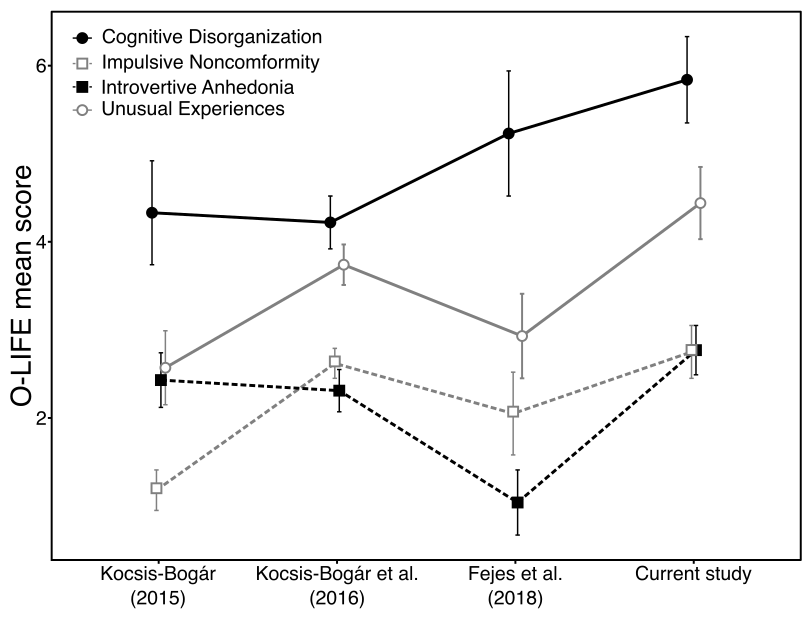


The Facet5 test was completed by all subjects – with the exception of 2 persons, (N = 144), but in the subsequent instrumental laboratory situation, not all subjects' test data proved to be usable. Facet5 has English and Hungarian psychometric analyses and standards based on large sample sizes (with standardized ten-point (Sten: 1-10) scoring, Standard-ten = 5.5; McDonald & Yarker, 2016; Nagybányai Nagy, 2013).

Significant differences in the Facet5 scores of all individuals were detected in our sample by one-sample t-tests (Table S6; Figure S6) compared to the available large-sample national standards.

***Table S6.***

*Facet5 mean differences (N=144) from the Hungarian normative Sten-point (5.5)*

| One-Sample T-Test | | | | |
| --- | --- | --- | --- | --- |
|  | Test Value = 5.5 | | | |
|  | t | Df | p | Mean difference |
| **Will (W)** | -3.043 | 143 | .003 | -0.5556 |
| W-Determination | 0.473 | 143 | .637 | 0.0910 |
| W-Confrontation | -6.780 | 143 | <.001 | -1.2389 |
| W-Independence | -2.515 | 143 | .013 | -0.4868 |
| **Energy (E)** | -5.581 | 143 | <.001 | -0.9569 |
| E-Vitality | -7.144 | 143 | <.001 | -1.3250 |
| E-Sociability | -8.943 | 143 | <.001 | -1.5035 |
| E-Adaptability | 0.069 | 143 | .945 | 0.0125 |
| **Affection (A)** | -0.895 | 143 | .372 | -0.1729 |
| A-Altruism | 0.785 | 143 | .434 | 0.1583 |
| A-Support | -3.209 | 143 | .002 | -0.6556 |
| A-Trust | 0.070 | 143 | .944 | 0.0132 |
| **Control (C)** | -1.436 | 143 | .153 | -0.2625 |
| C-Discipline | 0.682 | 143 | .497 | 0.1278 |
| C-Responsibility | -3.380 | 143 | <.001 | -0.6319 |
| **Emotionality (Em)** | 10.876 | 143 | <.001 | 1.7944 |
| Em-Tension | 8.148 | 143 | <.001 | 1.4931 |
| Em-Apprehension | 12.779 | 143 | <.001 | 2.0618 |

***Figure S6.***

*Distribution (means, minimum-maximum values) of Facet5 scales in the current sample (N=144)*


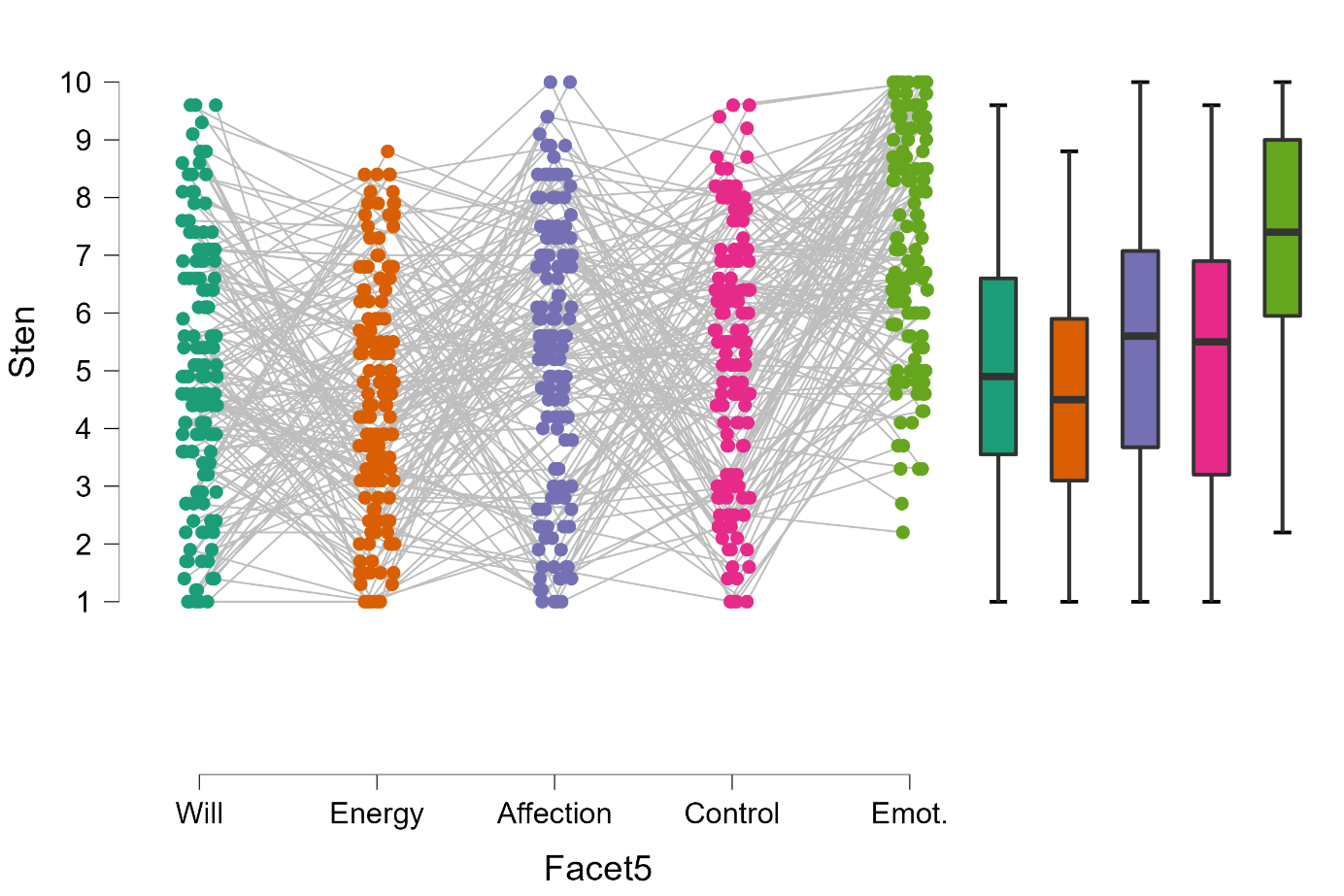


Note: Horizontal lines denote the mean of the group, upper and lower strokes of the box indicate the 1^st^ and 3^rd^ quartiles of the data, and whiskers indicate minimum and maximum values of the data range. Colored data points correspond to individual values. Dark green denotes Will, orange denotes Energy, purple denotes Affection, magenta denotes Control, and Emot. and light green denotes Emotionality, respectively.

1. **Scatter plots of individual behavioral and questionnaire data**

***Figure S7.***

*Histograms (left) and scatterplots (middle and right columns) of the O-LIFE Cognitive Disorganization and Unusual Experiences scores with the inflection point latencies (top), as well as force ratios between 0-ms-delay and motor condition (bottom) for participants with sigmoid fits of sufficient quality (N=102).*
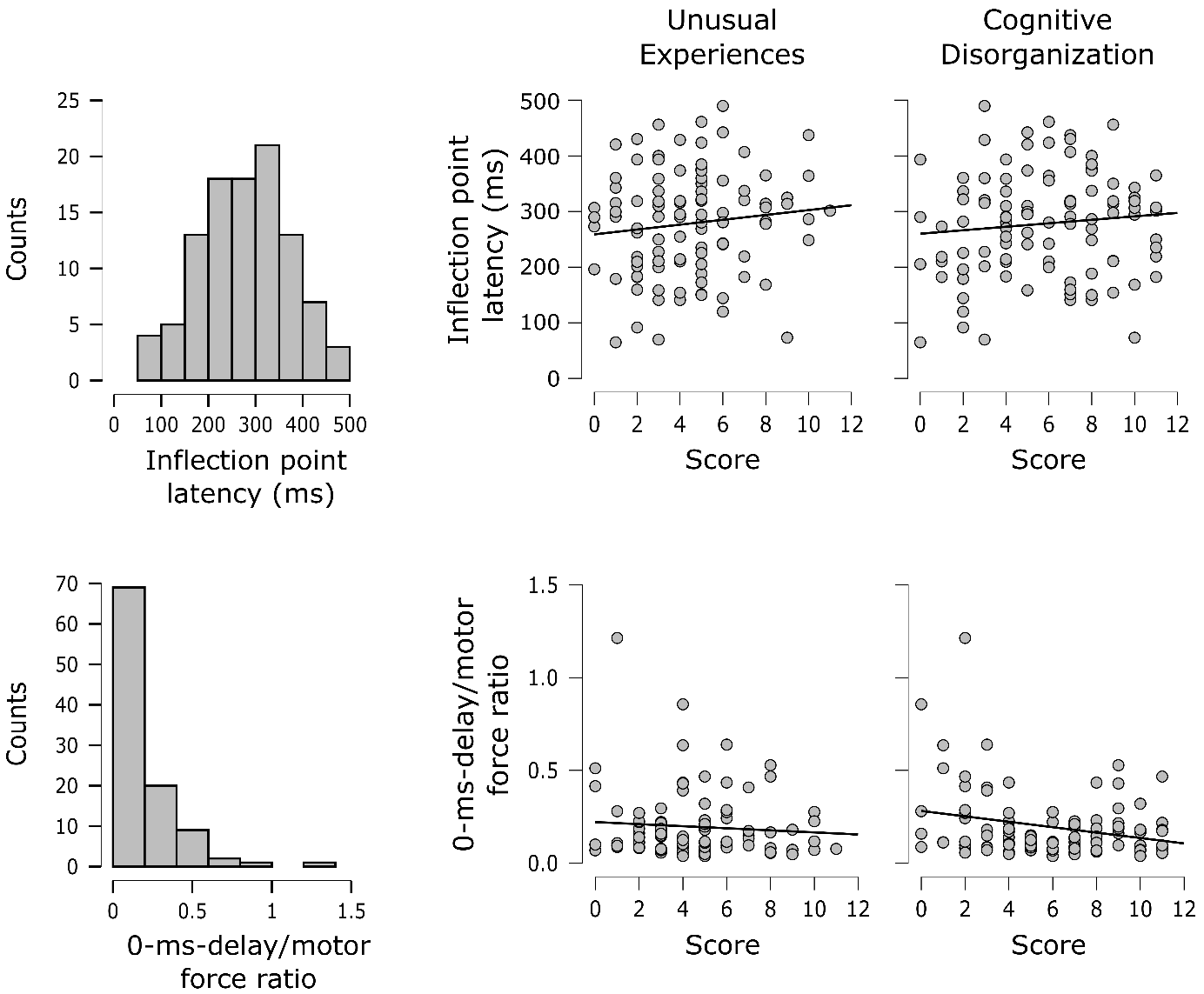


For Facet5, no significant correlations were present (Table S7 and Figure S6) with the inflection point latency, and only one significant correlation was found for the ratio of forces in the 0-ms-delay and motor conditions Note that the reported p-values are uncorrected for multiple testing, thus the single significant correlation with the Energy scale may well reflect a chance finding. Indeed, the low Bayes Factors provide “moderate” evidence (i.e., B_10_ <1/3, Lee and Wagenmakers, 2013) for the null-effect in most cases.

**Table S7**

*Kendall rank correlations (τb) between Facet5 factors and inflection point latency as well as the ratio of forces in the 0-ms-delay and motor conditions. Uncorrected p-values and Bayes-factors in favor of the alternative hypothesis (BF_10_) are shown.*

| Facet5 factor | Inflection point latency | | | 0-ms-delay and motor Peak force ratio | | |
| --- | --- | --- | --- | --- | --- | --- |
|  | τb | p | BF_10_ | τb | p | BF_10_ |
| Will | -.002 | .975 | 0.129 | .040 | .557 | 0.154 |
| Energy | -.007 | .919 | 0.130 | .140* | .039 | 1.116 |
| Affection | -.054 | .424 | 0.179 | .016 | .817 | 0.133 |
| Control | -.004 | .954 | 0.129 | .094 | .166 | 0.342 |
| Emotionality | .004 | .958 | 0.129 | .023 | .737 | 0.137 |

Note: Significance values: *p < .05

***Figure S8.***

*Histograms and scatterplots of the Facet5 scores with the inflection point latencies (top), as well as force ratios between 0-ms-delay and motor condition (bottom) for participants with sigmoid fits of sufficient quality (N=102).*


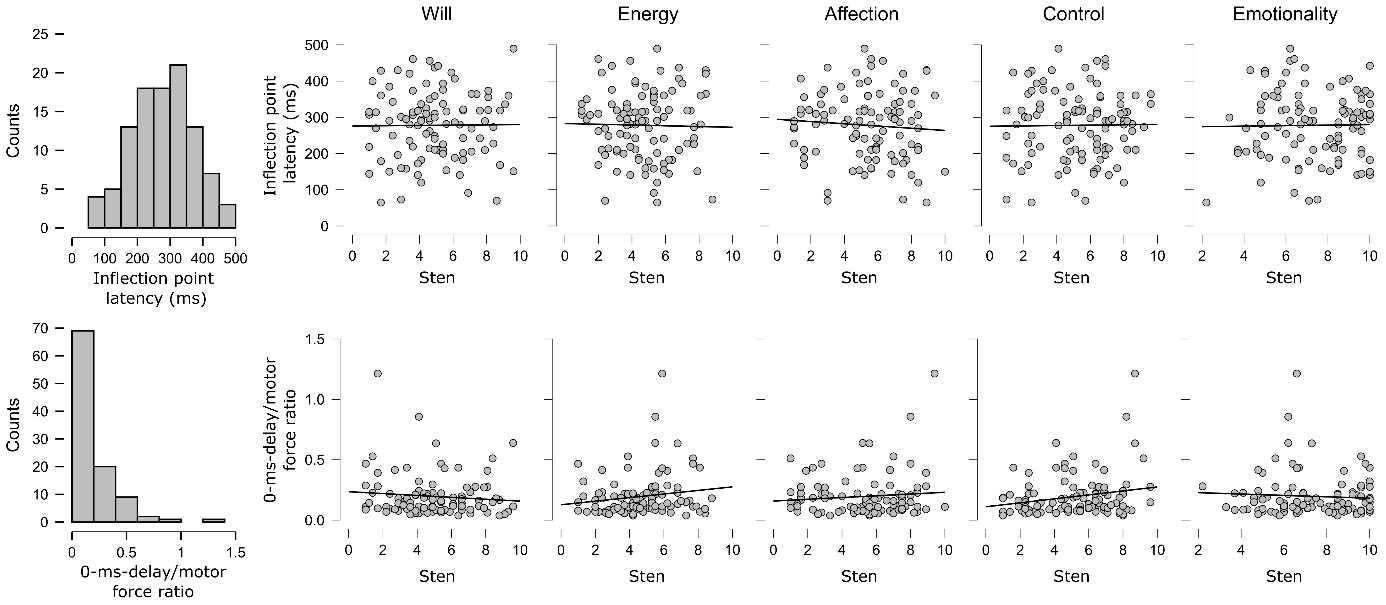


1. **Common factor analysis on behavioral and Facet5 data**

A common factor analysis is essentially a principal component analysis (PCA). It is called common because here we include variables from different sources in a common analysis, hoping that the otherwise negligible correlations will together produce a more salient picture. The linear combinations of these variables result in a small number of uncorrelated principal components, which can explain most of the total variance of the original variables, and also provides a better overview of the organization between variables.

The common factor analysis with the Facet5 scales was applied as an exploratory step. Correlations between variables and principal components are shown in the Component Matrix table (coefficients below 0.3 absolute values are not shown; Table S7). The two experimental variables were found to be mostly nested within the subscales of the Affection dimension (Agreeableness) and Control dimension (Conscientiousness), forming two common factors (Components 3 and 4) with them, although some linkage with the other four personality dimensions remains, but to a negligible extent.

The total variance explained by the five generated factors was 81.9% (17.3% for Component 3, and 13.3% for Component 4). The use of the Varimax rotation with Kaiser normalization is dictated by the Big Five model framework, which requires the formation of independent personality factors.

***Table S7.***

*Common factor analysis of all Facet5 scales and two experimental variables. Coefficients below 0.3 absolute values are not shown.*

|  | Component | | | | |
| --- | --- | --- | --- | --- | --- |
|  | 1 | 2 | 3 | 4 | 5 |
| Will-Determination | .942 |  |  |  |  |
| Will-Confrontation | .936 |  |  |  |  |
| Will-Independence | .876 |  |  |  |  |
| Energy-Vitality |  | .945 |  |  |  |
| Energy-Sociability |  | .922 |  |  |  |
| Energy-Adaptability |  | .892 |  |  |  |
| Affection-Support | -.343 |  | **.889** |  |  |
| Affection-Trust | -.375 |  | **.873** |  |  |
| Affection-Altruism | -.362 |  | **.865** |  |  |
| ***inflection point latency*** |  |  | ***-.328*** |  |  |
| Control -Responsibility |  |  |  | **.941** |  |
| Control -Discipline |  |  |  | **.913** |  |
| ***0-ms-delay/motor force ratio*** |  |  |  | ***.497*** |  |
| Emotionality-Apprehension |  |  |  |  | .949 |
| Emotionality-Tension |  |  |  |  | .918 |

Note: Experimental variables are highlighted in italics and bold. Loadings to the 3^rd^ and 4^th^ Factor are highlighted in bold.

**References**

Buckley, N., & Williams, R. (2002). Testing on the web – Response patterns and image management. *Selection & Development Review, 18*, 3-8.

Fejes, N. É., Rózsa, S., & Must, A. (2018). Szkizotíp személyiségvonások és a nyelvi kreativitás összefüggéseinek vizsgálata [Schizotypal traits and verbal creativity]. *Ideggyógyászati Szemle, 71*(3-4), 113-125.

Kocsis-Bogár, K. (2015). *A szkizofrénia spektrum és a traumatikus életesemények összefüggései*. Semmelweis University (Doctoral dissertation). <https://doi.org/10.14753/SE.2016.1912>

Kocsis-Bogár, K., Nemes, Z., & Perczel-Forintos, D. (2016). Factorial structure of the Hungarian version of Oxford-Liverpool Inventory of Feelings and Experiences and its applicability on the schizophrenia-schizotypy continuum. *Personality and Individual Differences*, *90*, 130-136.

Lee, K., & Ashton, M. C. (2004). Psychometric properties of the HEXACO personality inventory. *Multivariate Behavioral Research, 39*(2), 329-358.

Lee, M. D., & Wagenmakers, E.-J. (2013). *Bayesian cognitive modeling: A practical course.* Cambridge University Press.

McDonald, A., & Yarker, J. (2016). *Test Review: Facet5.* The British Psychological Society, Psychological Testing Centre. http://www.facet5global.com/_literature_218922/BPS_Review_2015

Nagybányai Nagy, O. (2013). Online személyiségmérés a hazai Big Five struktúra mentén: a Facet5 teszt magyar adaptációja. *Pszichológia, 33*(1), 37-59.

Szirmák, Z., & De Raad, B. (1994). Személyiségtaxonómia a magyar nyelv személyleíró szókincse. *Magyar Pszichológiai Szemle, 50(1-2)*, 39-65.
