# Supplementary material for: An upper temporal limit of action-effect integration as reflected by motor adaptation
Source: Psychol Res. 2025 Apr 23;89(3):94. doi: 10.1007/s00426-025-02121-4 (PMC12014811; doi:10.1007/s00426-025-02121-4)
Supplement: Supplementary file 2 — Supplementary Material 2 [file 426_2025_2121_MOESM2_ESM.pdf]

Participant code: 003

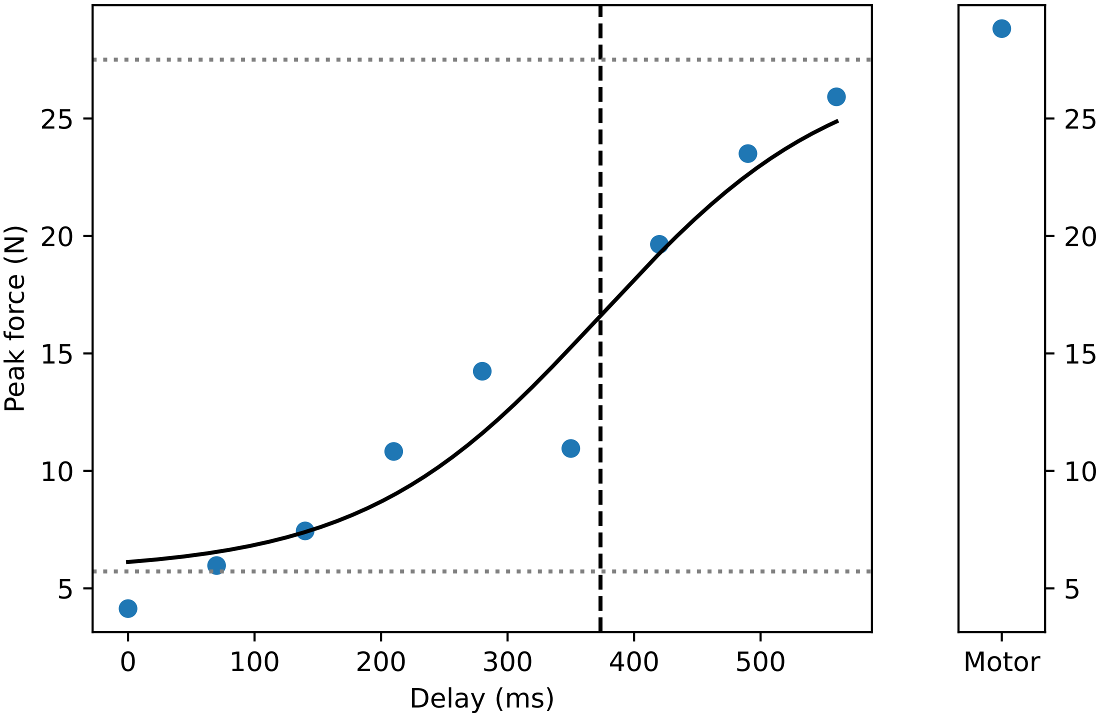

R^2: 0.93 Inflection point: 373.46 ms

Lower asymptote: 5.72 N; Upper asymptote: 27.5 N

Participant code: 008

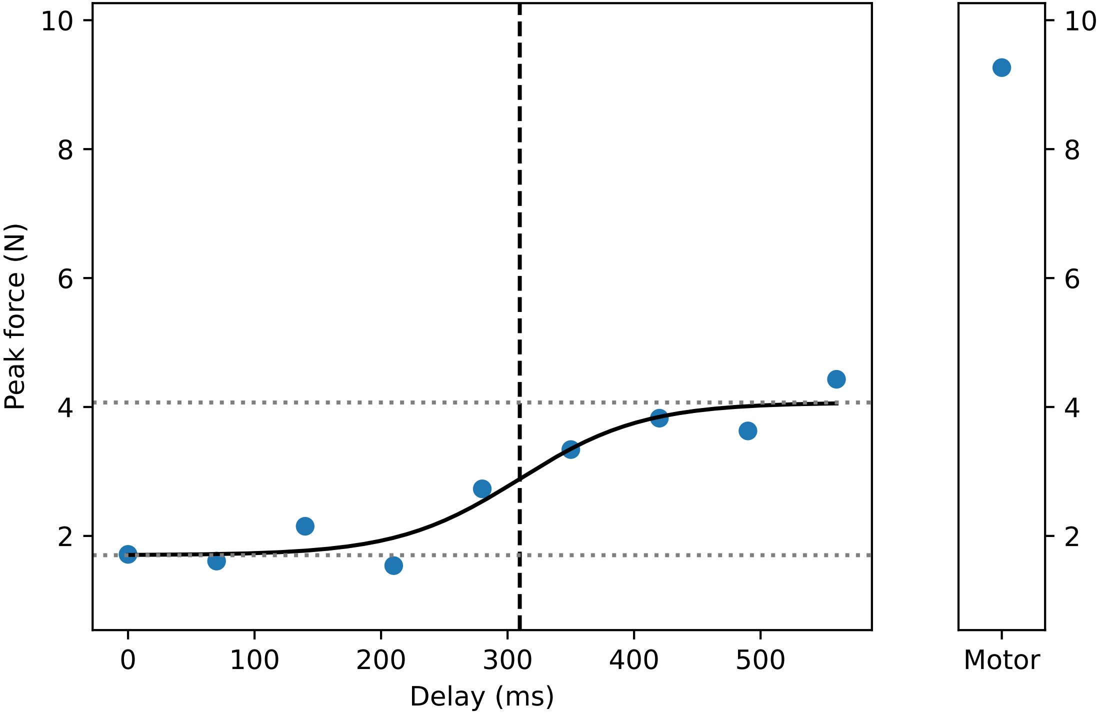

R^2: 0.93 Inflection point: 309.64 ms

Lower asymptote: 1.7 N; Upper asymptote: 4.07 N

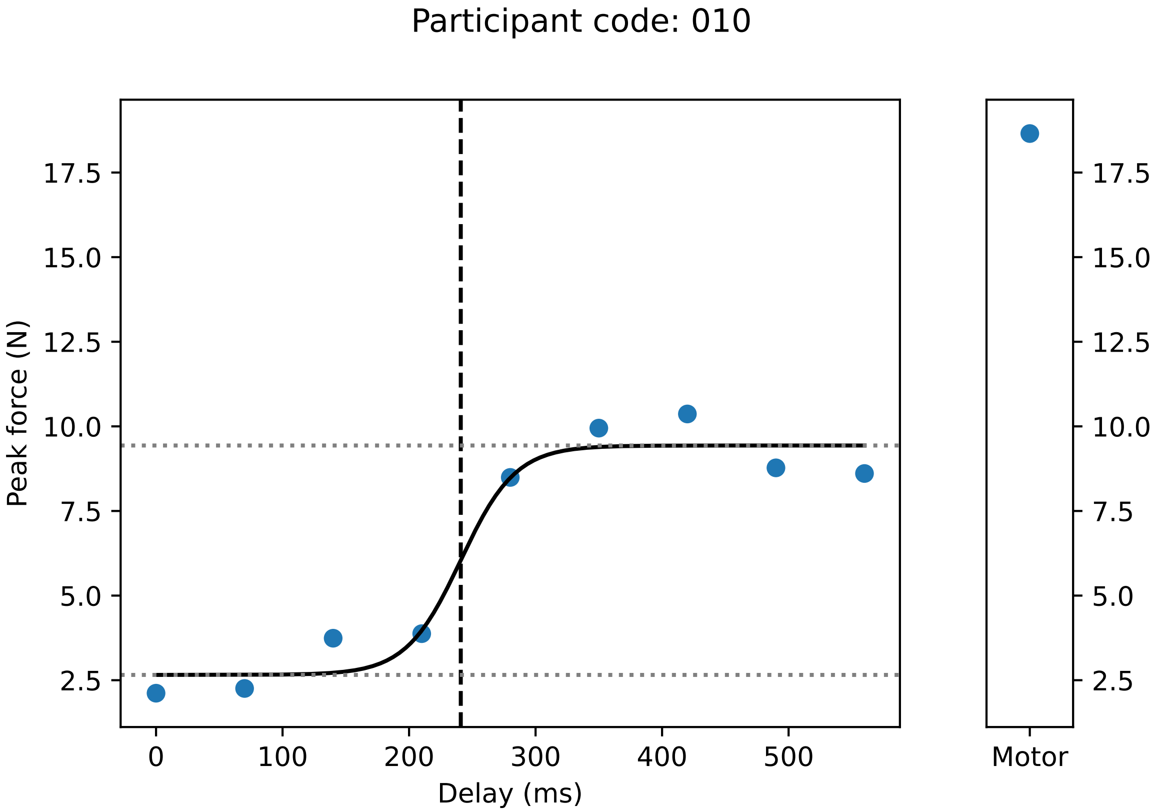

R^2: 0.96 Inflection point: 240.88 ms
Lower asymptote: 2.66 N; Upper asymptote: 9.43 N

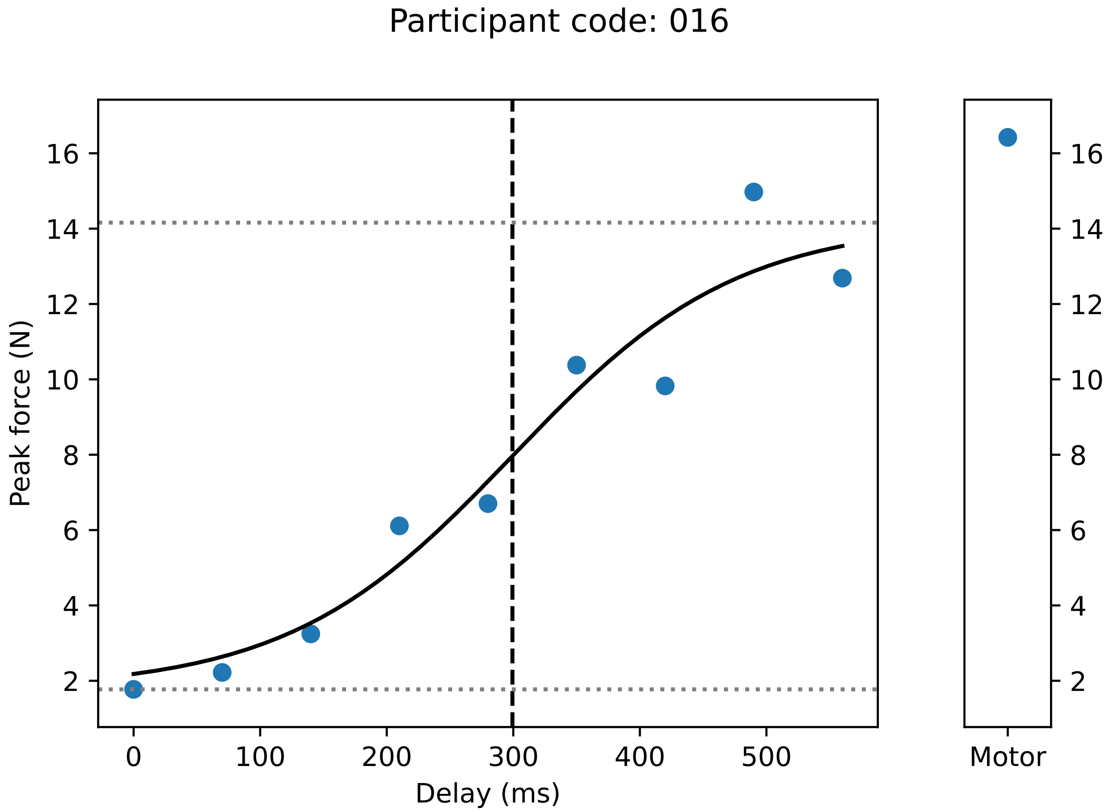

R^2: 0.94 Inflection point: 299.3 ms

Lower asymptote: 1.77 N; Upper asymptote: 14.16 N

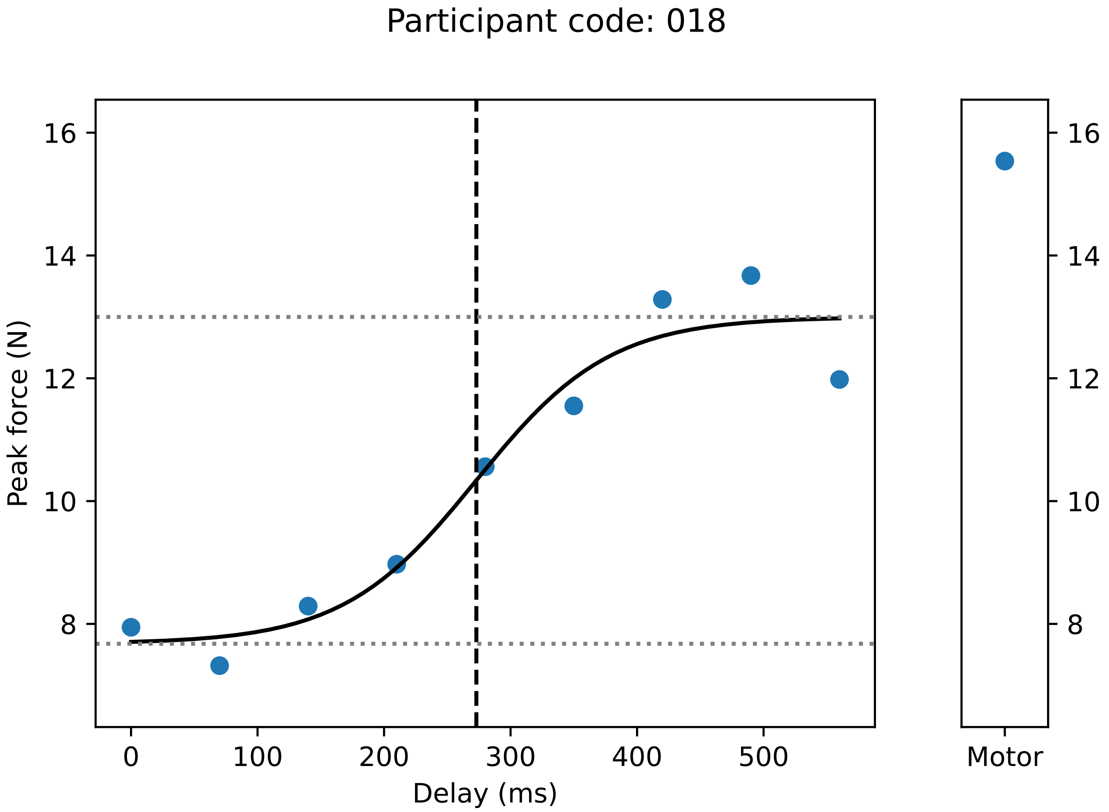

R^2: 0.95 Inflection point: 272.95 ms

Lower asymptote: 7.68 N; Upper asymptote: 13.0 N

Participant code: 026

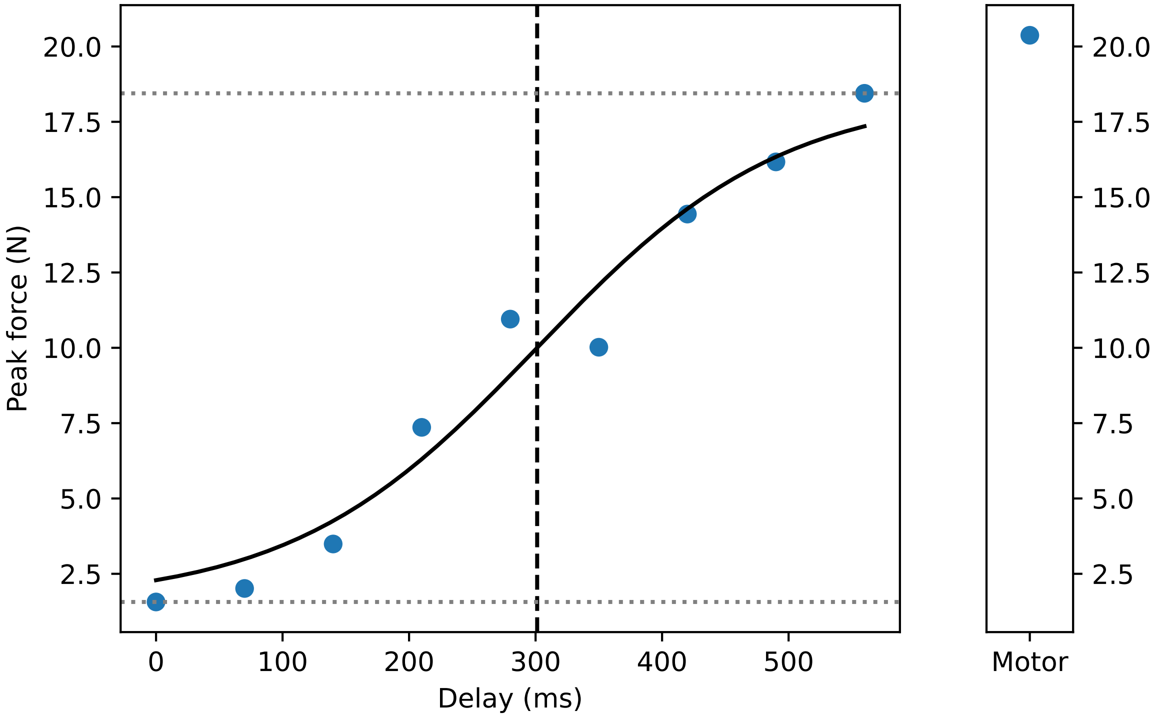

R^2: 0.96 Inflection point: 301.25 ms

Lower asymptote: 1.57 N; Upper asymptote: 18.45 N

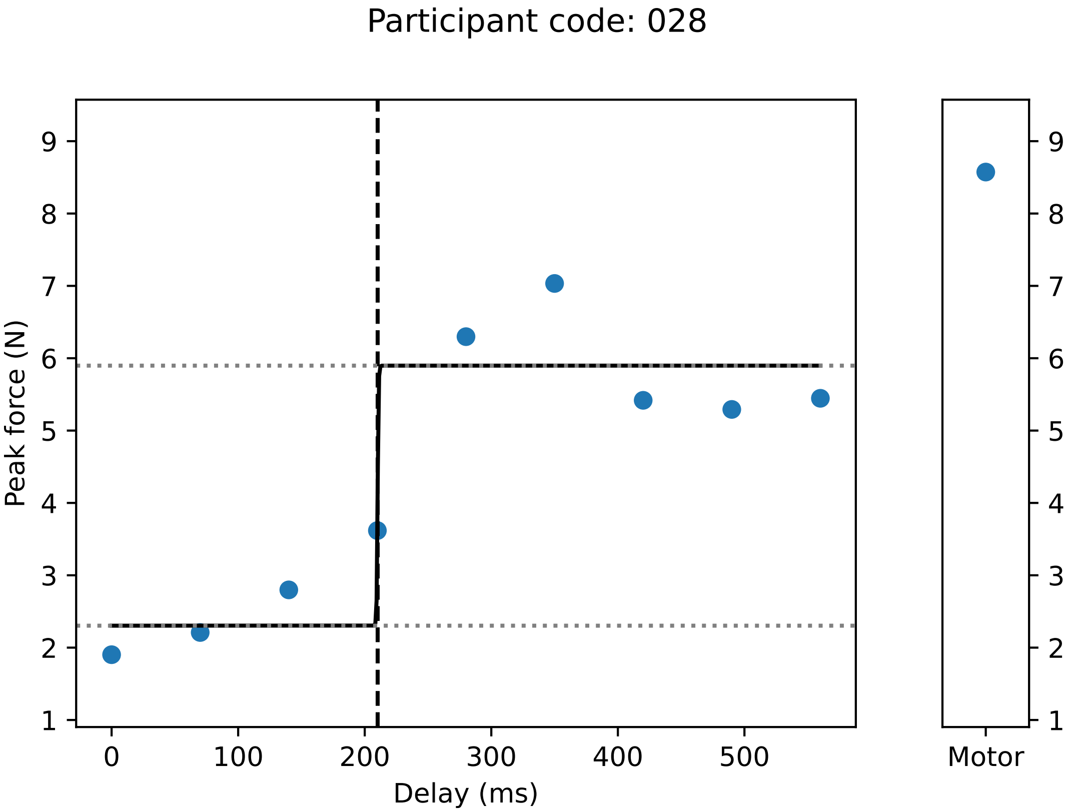

R^2: 0.9   Inflection point: 210.2 ms

Lower asymptote: 2.3 N; Upper asymptote: 5.9 N

Participant code: 029

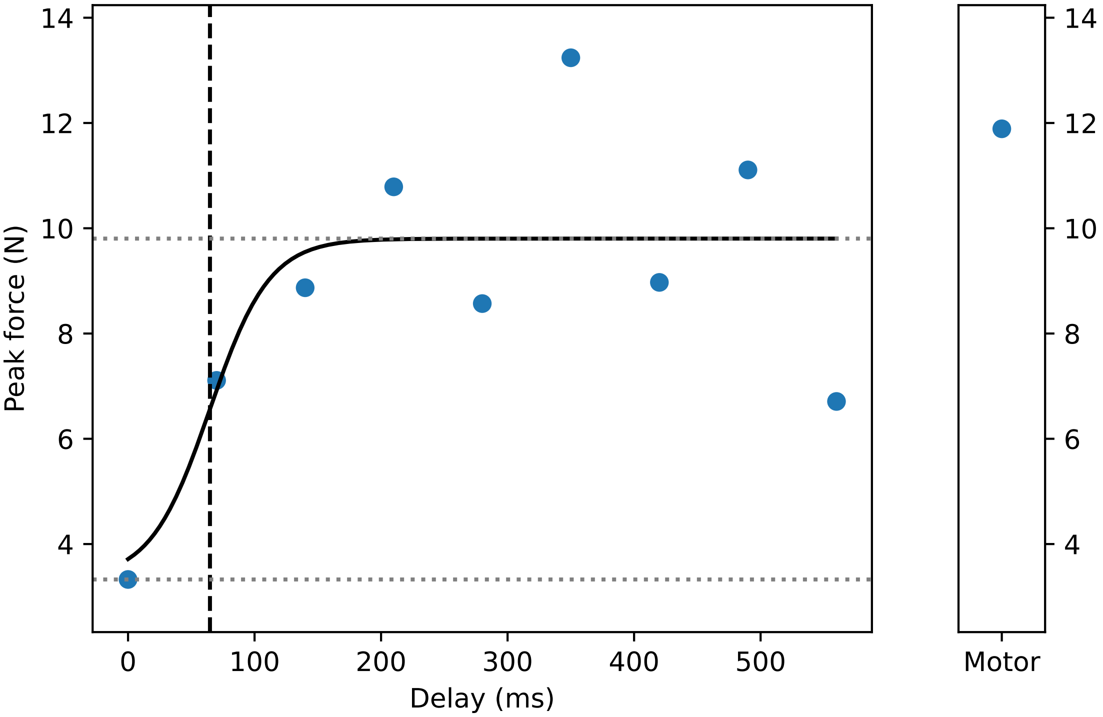

R^2: 0.59 Inflection point: 64.75 ms
Lower asymptote: 3.33 N; Upper asymptote: 9.8 N

Participant code: 030

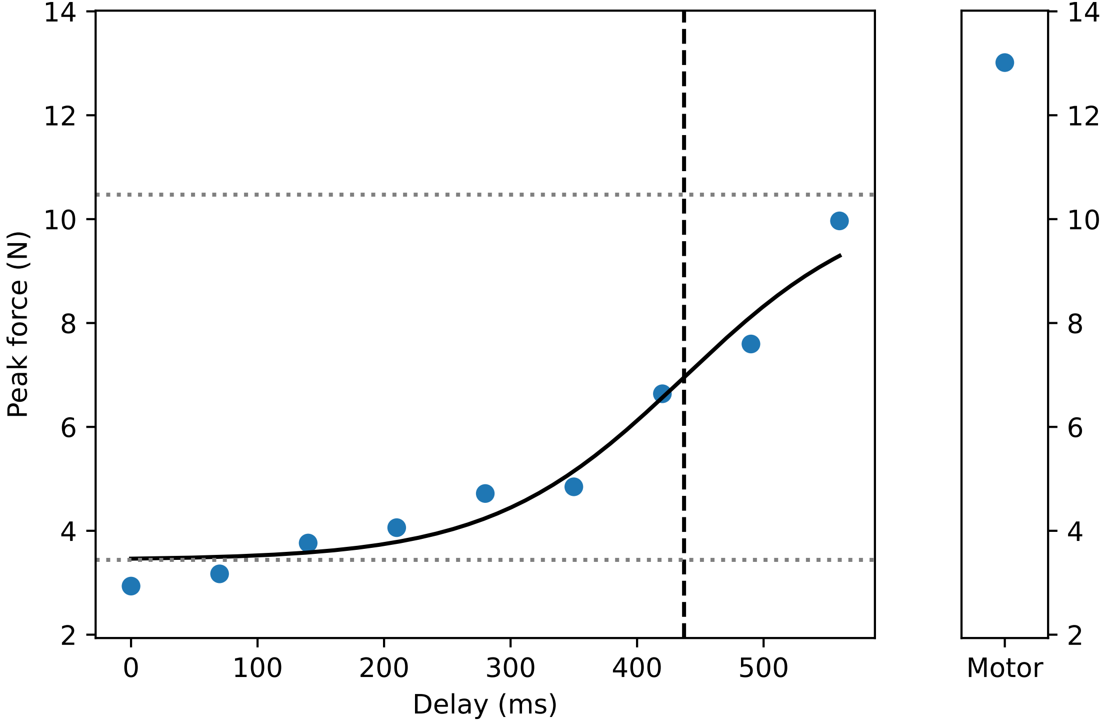

R^2: 0.96 Inflection point: 437.14 ms

Lower asymptote: 3.44 N; Upper asymptote: 10.47 N

Participant code: 037

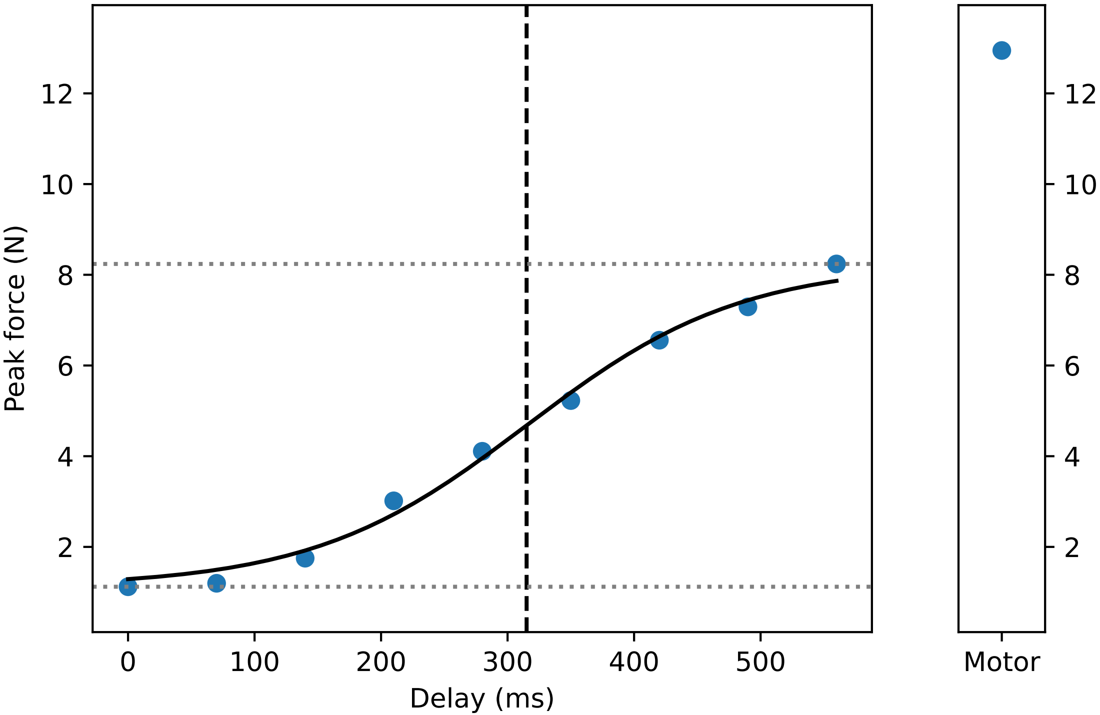

R^2: 0.99 Inflection point: 315.05 ms

Lower asymptote: 1.12 N; Upper asymptote: 8.24 N

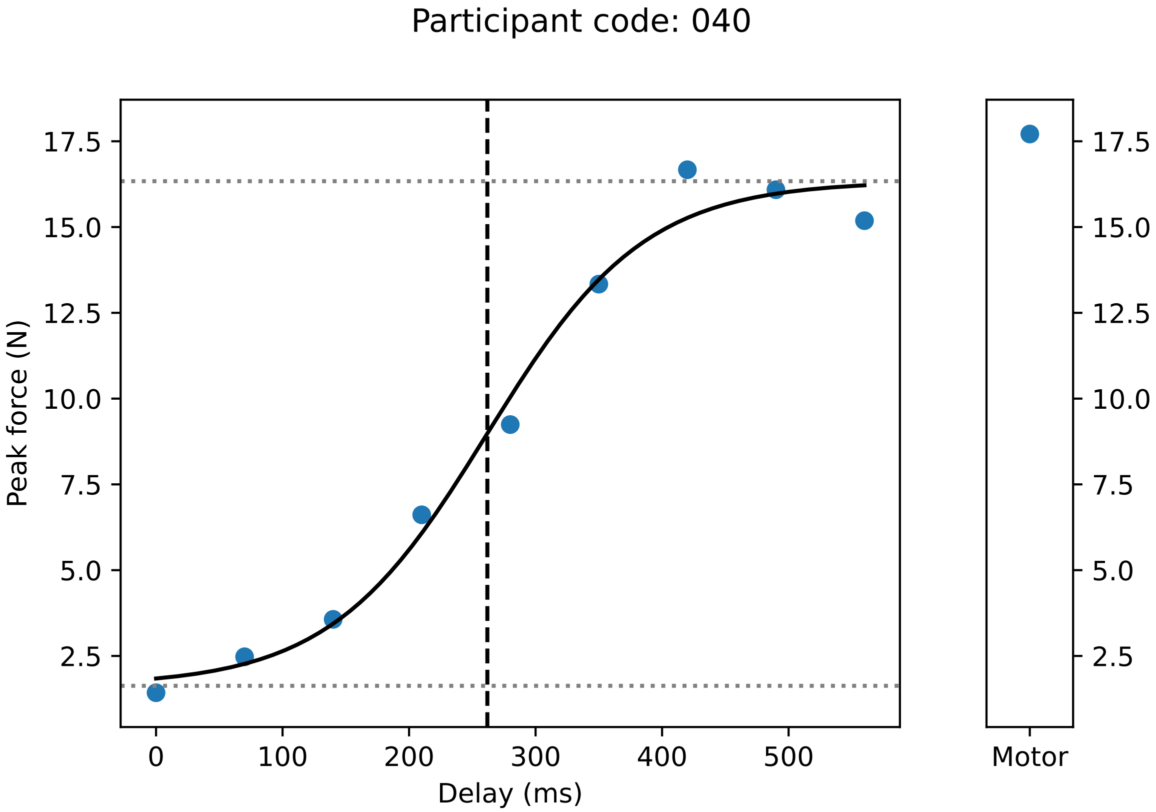

R^2: 0.99 Inflection point: 261.88 ms

Lower asymptote: 1.63 N; Upper asymptote: 16.34 N

Participant code: 041

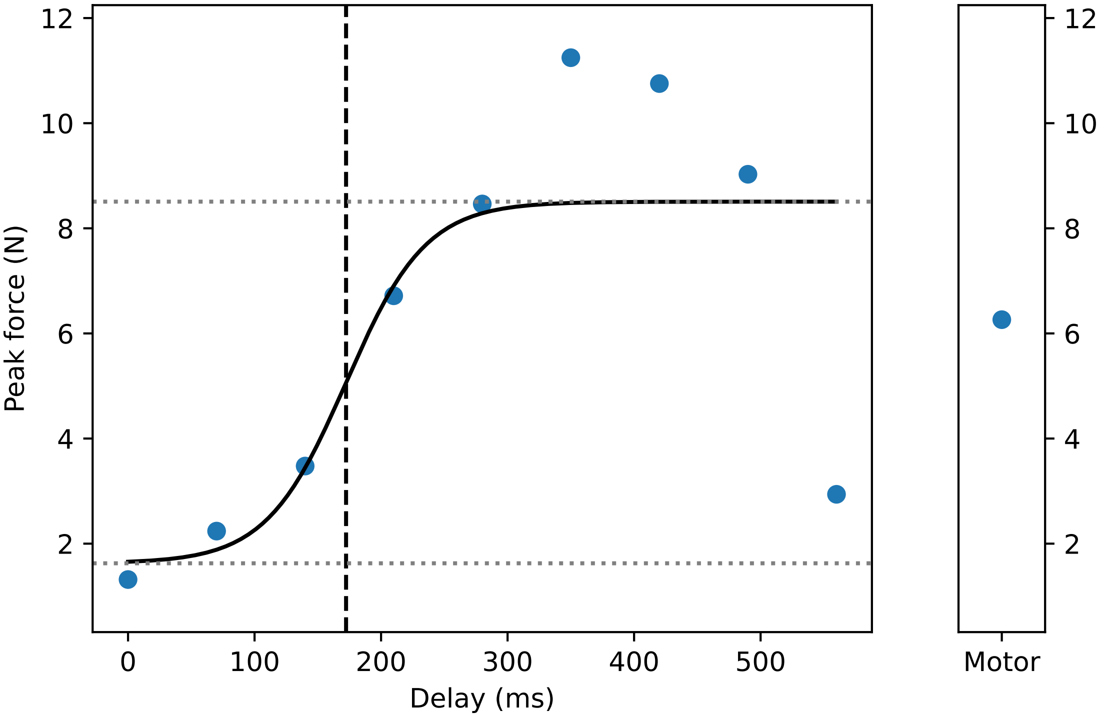

R^2: 0.62 Inflection point: 172.34 ms

Lower asymptote: 1.63 N; Upper asymptote: 8.51 N

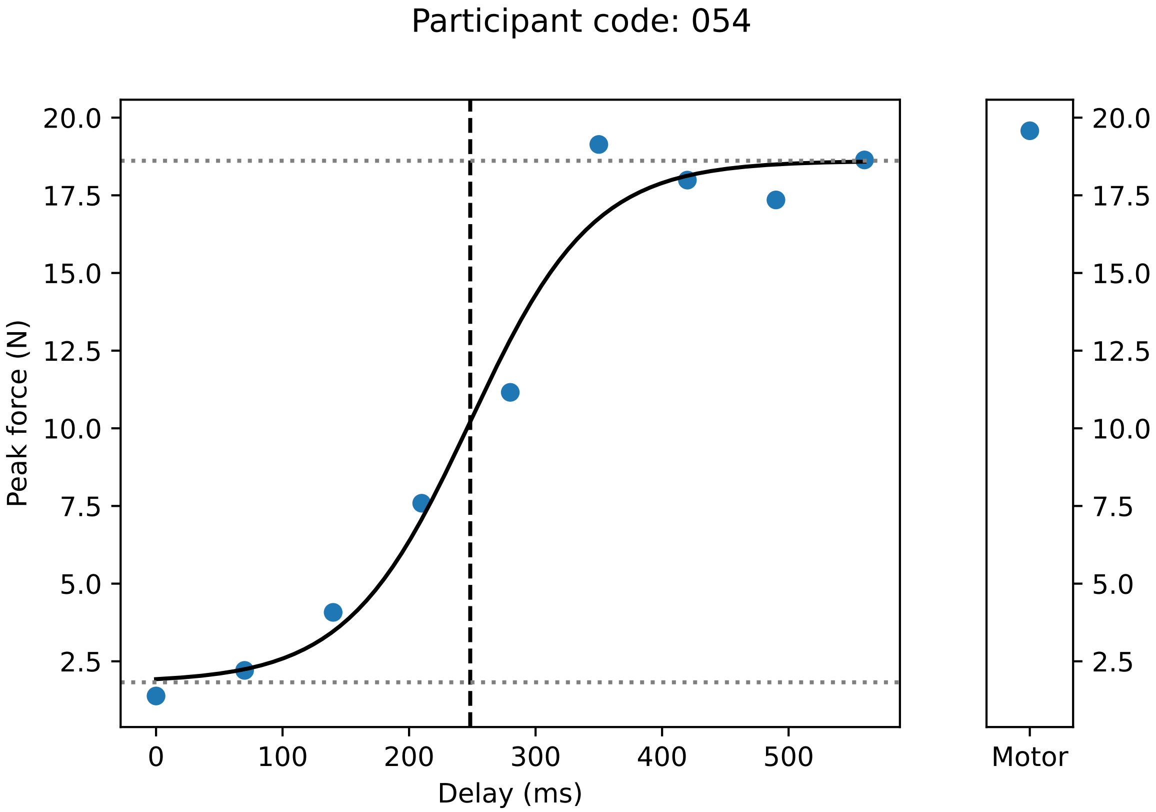

R^2: 0.98 Inflection point: 248.35 ms

Lower asymptote: 1.82 N; Upper asymptote: 18.61 N

Participant code: 055

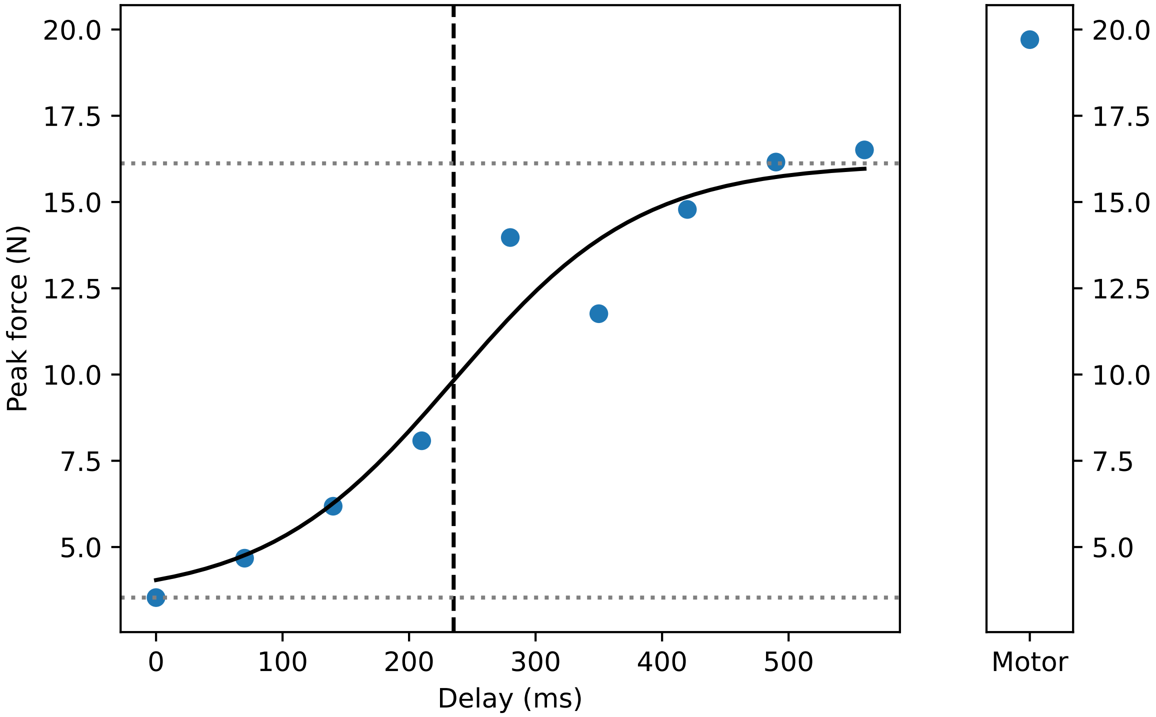

R^2: 0.95 Inflection point: 235.23 ms

Lower asymptote: 3.53 N; Upper asymptote: 16.12 N

Participant code: 062 !

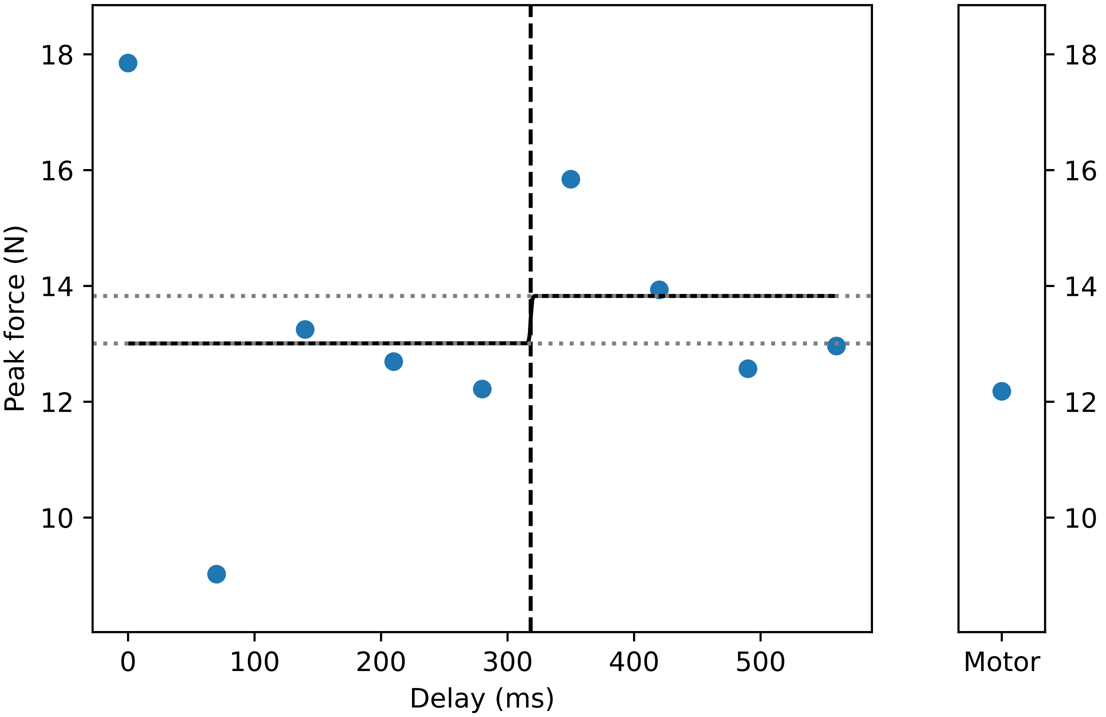

R^2: 0.03 ! Inflection point: 318.27 ms

Lower asymptote: 13.01 N; Upper asymptote: 13.83 N

Participant code: 067

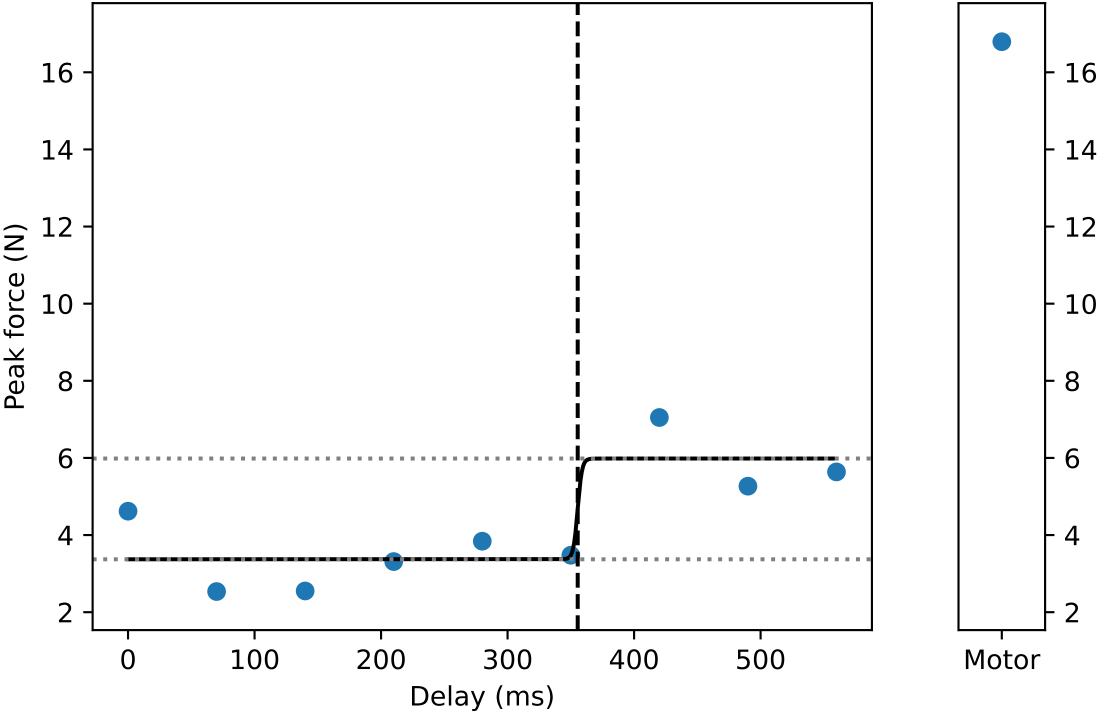

R^2: 0.73 Inflection point: 355.44 ms
Lower asymptote: 3.37 N; Upper asymptote: 5.98 N

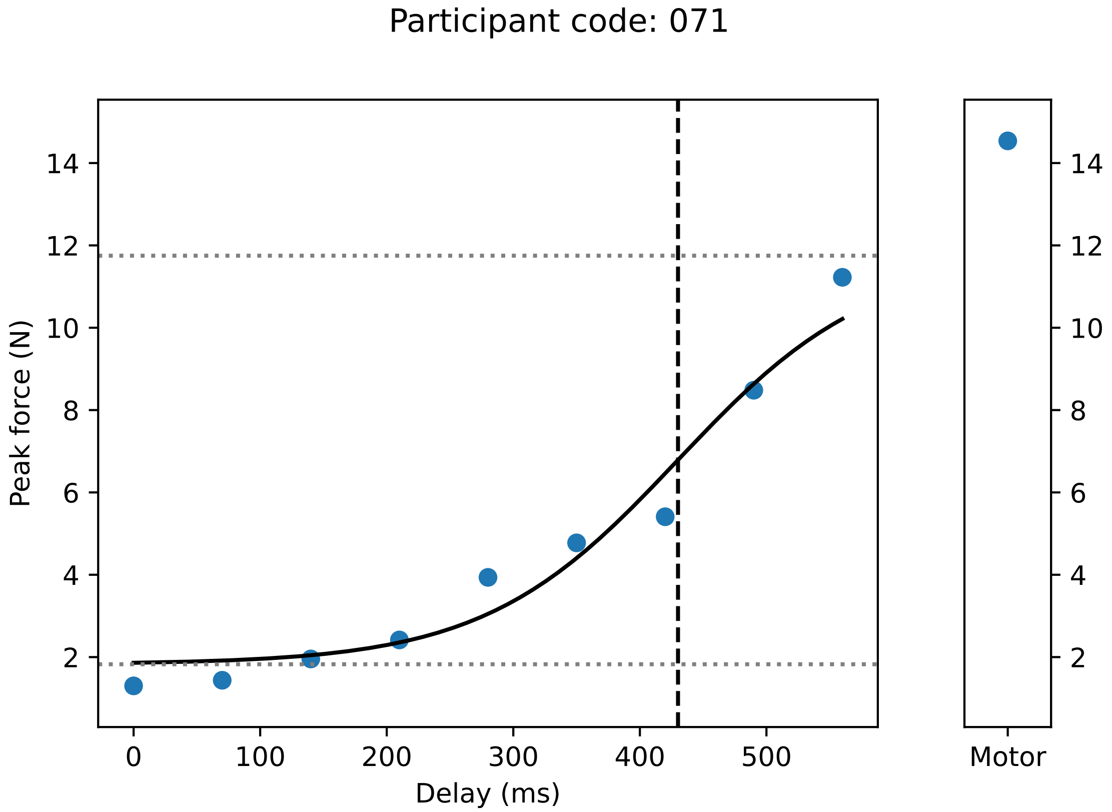

R^2: 0.96 Inflection point: 430.14 ms

Lower asymptote: 1.83 N; Upper asymptote: 11.75 N

Participant code: 075

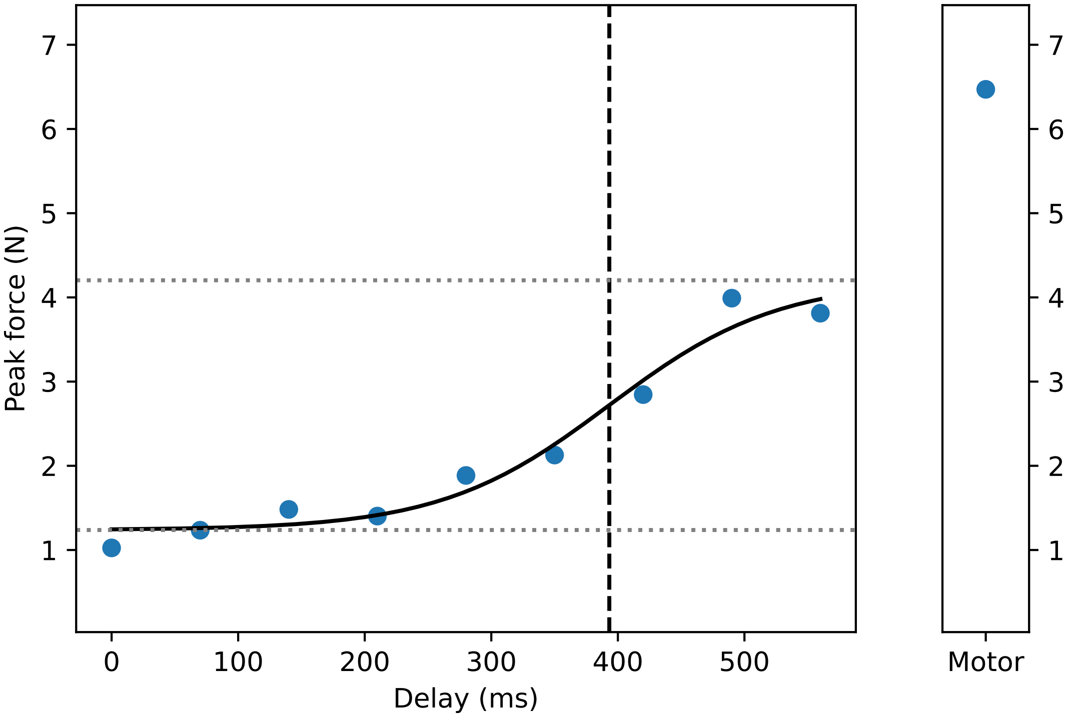

R^2: 0.97 Inflection point: 393.24 ms

Lower asymptote: 1.24 N; Upper asymptote: 4.2 N

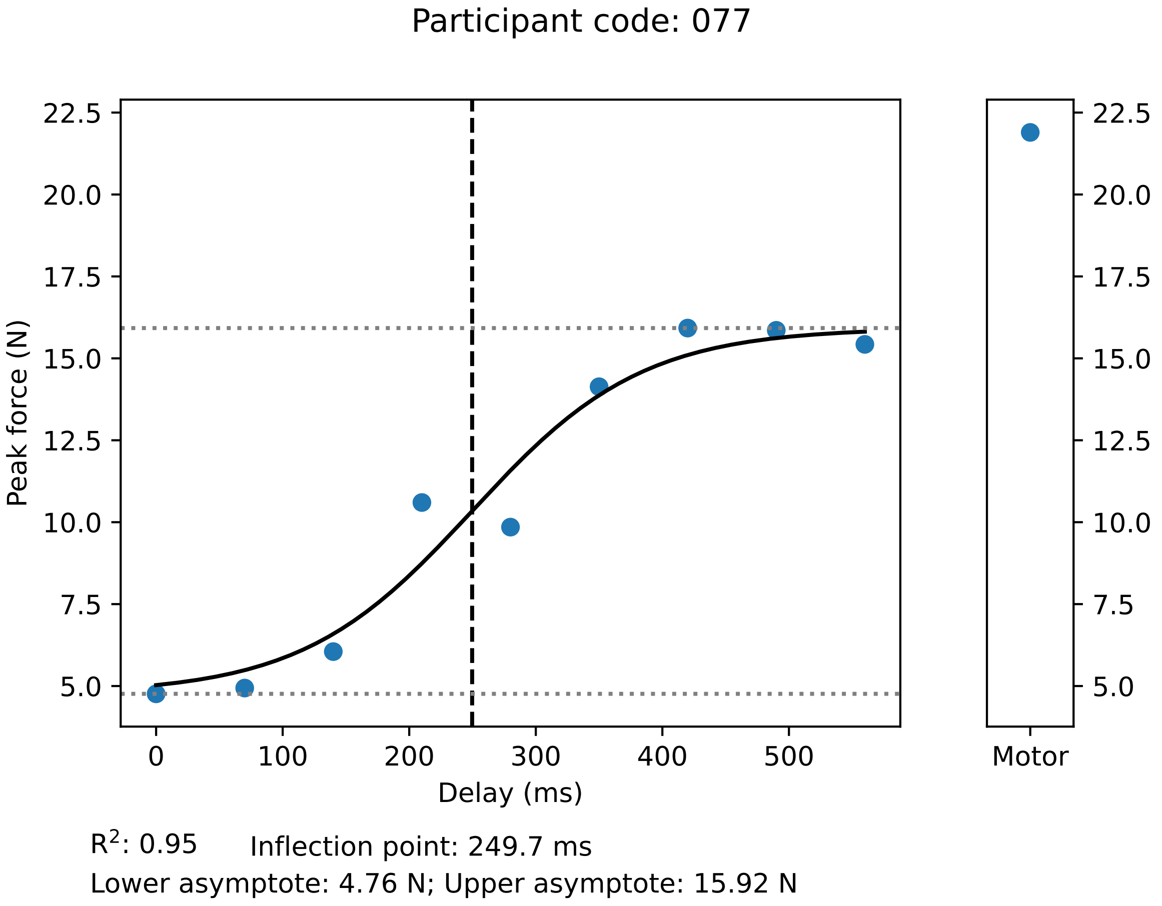

Participant code: 077
Peak force (N)
22.5
20.0
17.5
15.0
12.5
10.0
7.5
5.0
0
100
200
300
400
500
Delay (ms)
Motor
R²: 0.95
Inflection point: 249.7 ms
Lower asymptote: 4.76 N; Upper asymptote: 15.92 N

Participant code: 078

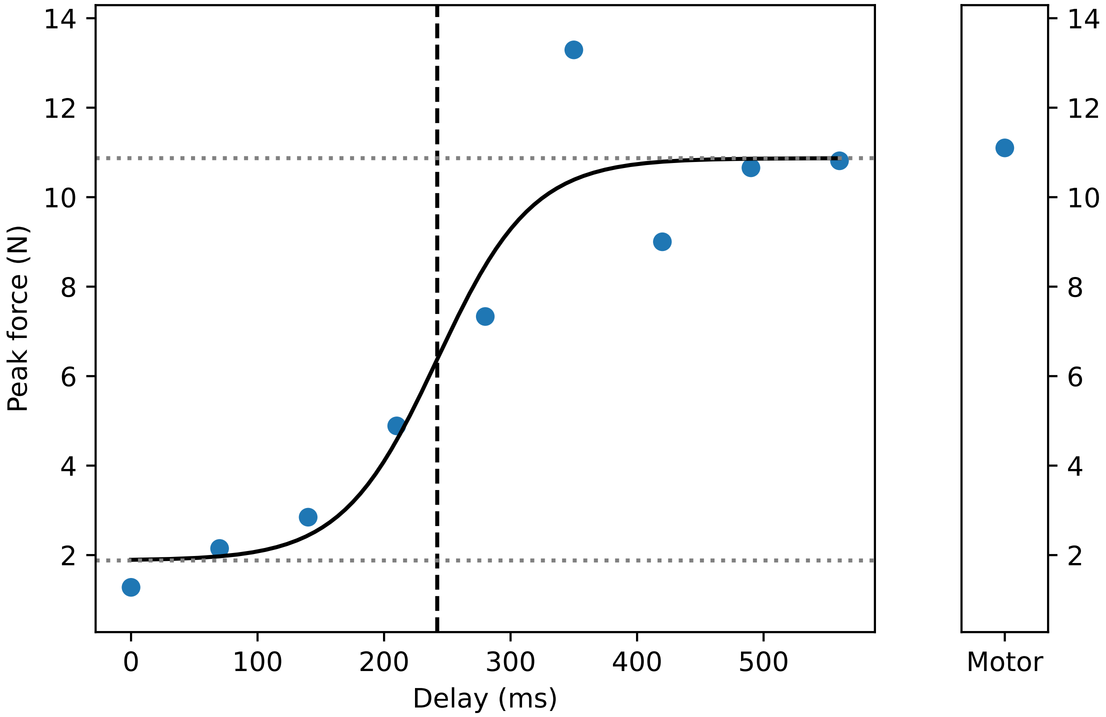

R^2: 0.91 Inflection point: 242.02 ms

Lower asymptote: 1.88 N; Upper asymptote: 10.87 N

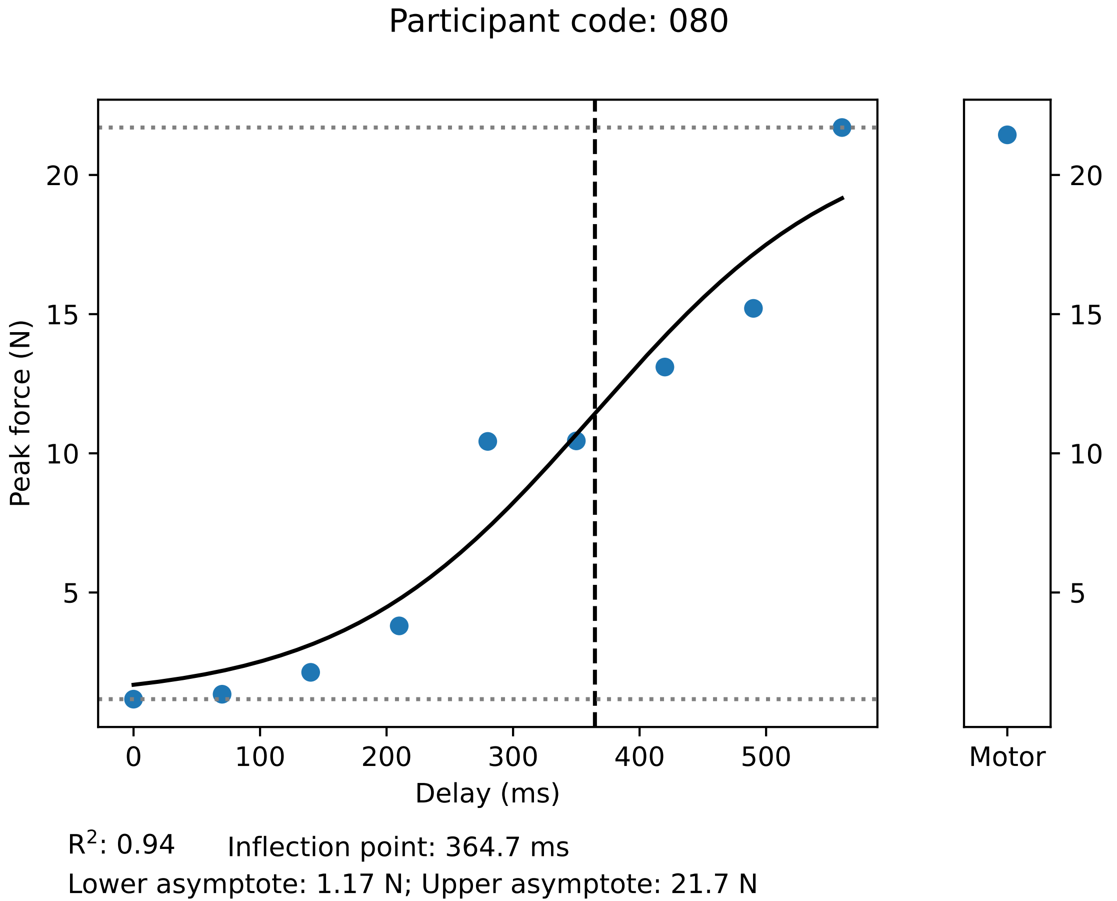
Participant code: 080
Peak force (N)
20
15
10
5
0
100
200
300
400
500
Delay (ms)
Motor
R²: 0.94
Inflection point: 364.7 ms
Lower asymptote: 1.17 N; Upper asymptote: 21.7 N

Participant code: 085

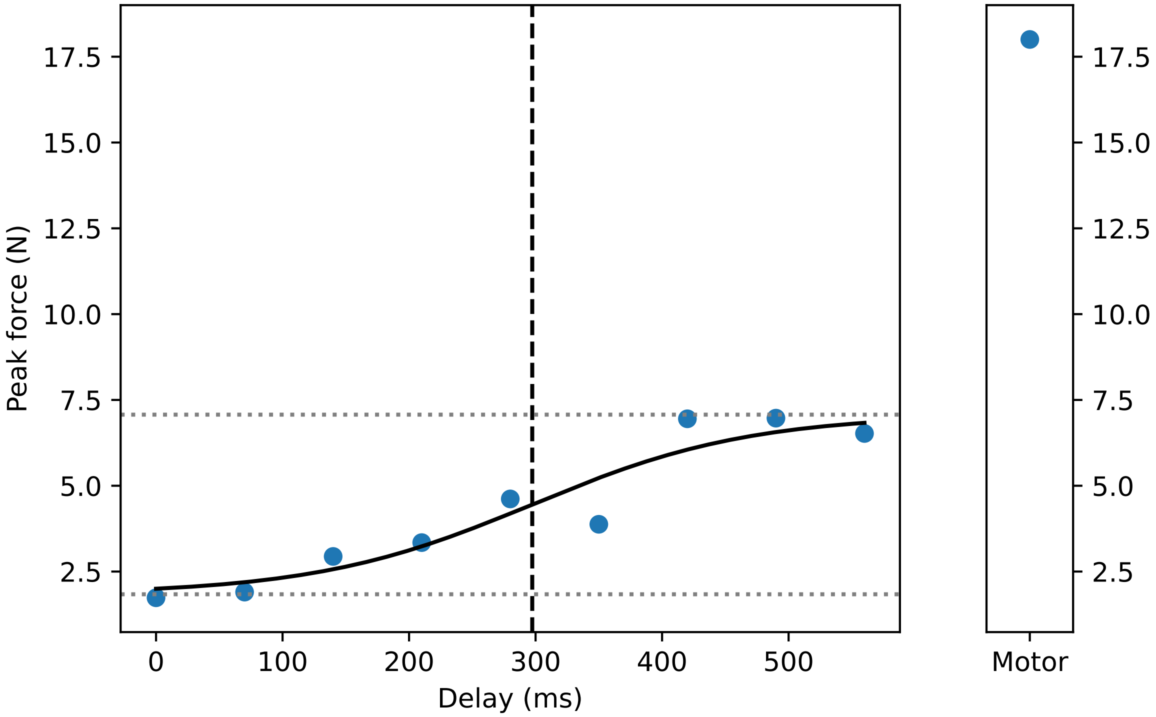

R^2: 0.9 Inflection point: 297.37 ms

Lower asymptote: 1.84 N; Upper asymptote: 7.07 N

Participant code: 086

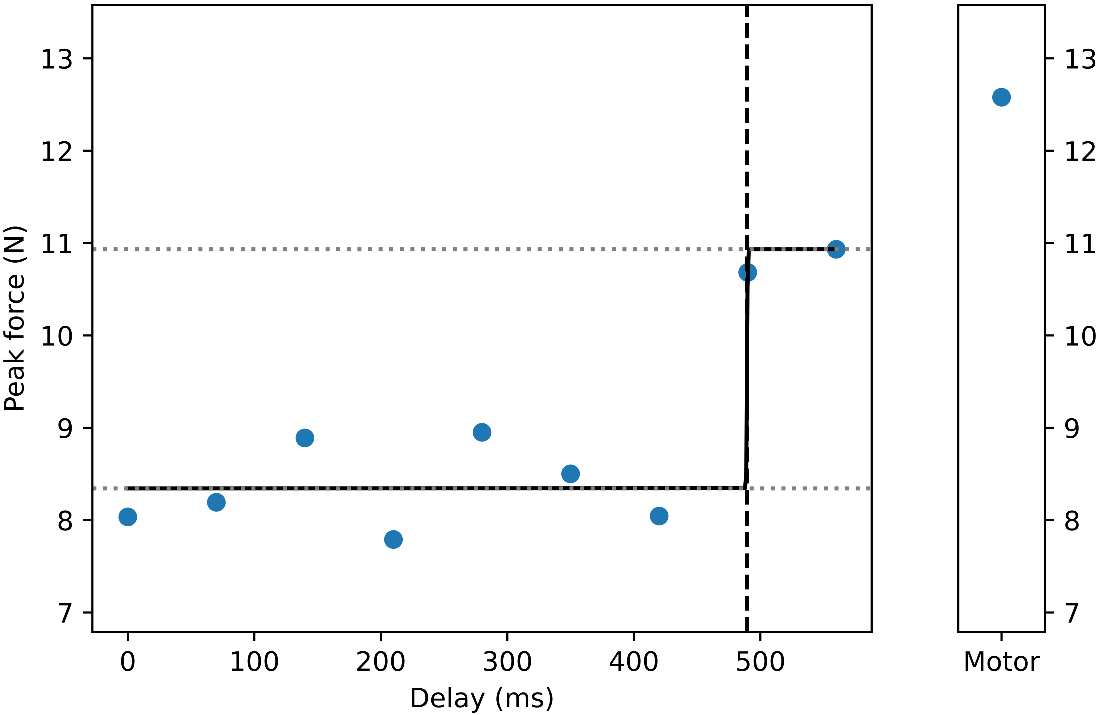

R^2: 0.89 Inflection point: 489.5 ms

Lower asymptote: 8.34 N; Upper asymptote: 10.93 N

Participant code: 090 !

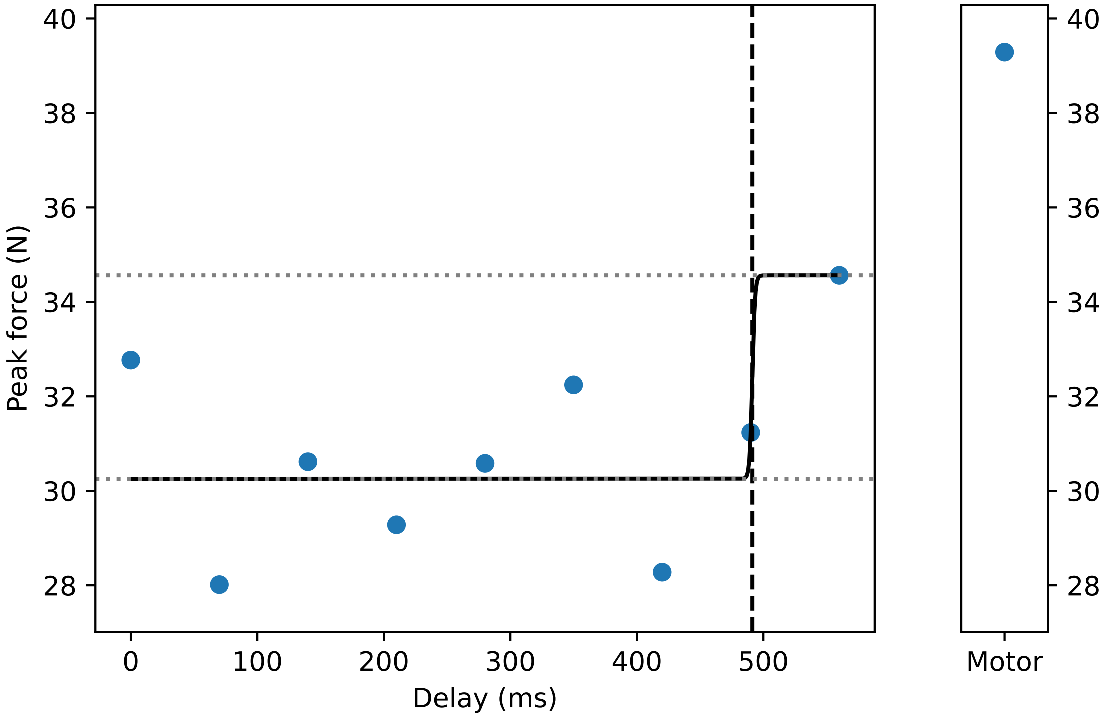

R^2: 0.45 ! Inflection point: 491.29 ms

Lower asymptote: 30.25 N; Upper asymptote: 34.56 N

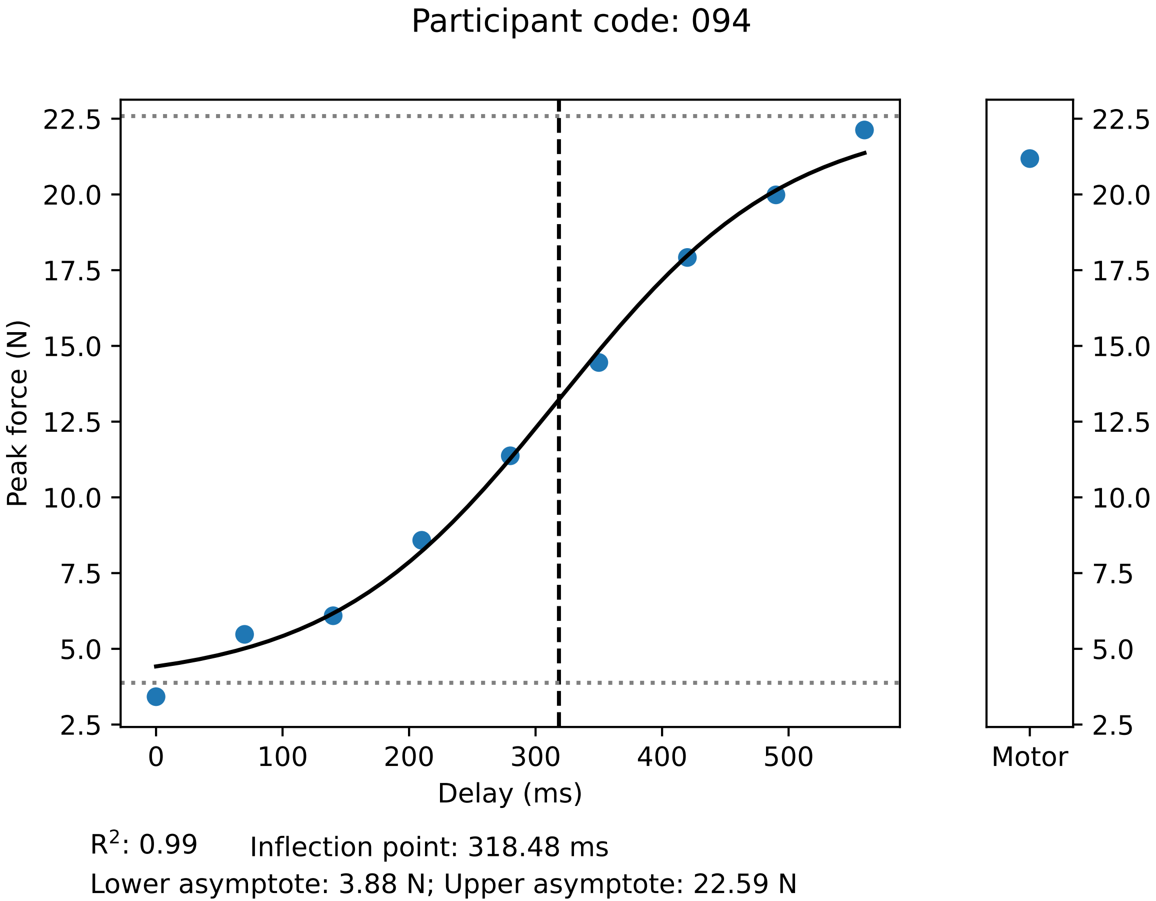

Participant code: 094
Peak force (N)
22.5
20.0
17.5
15.0
12.5
10.0
7.5
5.0
2.5
0
100
200
300
400
500
Delay (ms)
Motor
R^2: 0.99
Inflection point: 318.48 ms
Lower asymptote: 3.88 N; Upper asymptote: 22.59 N

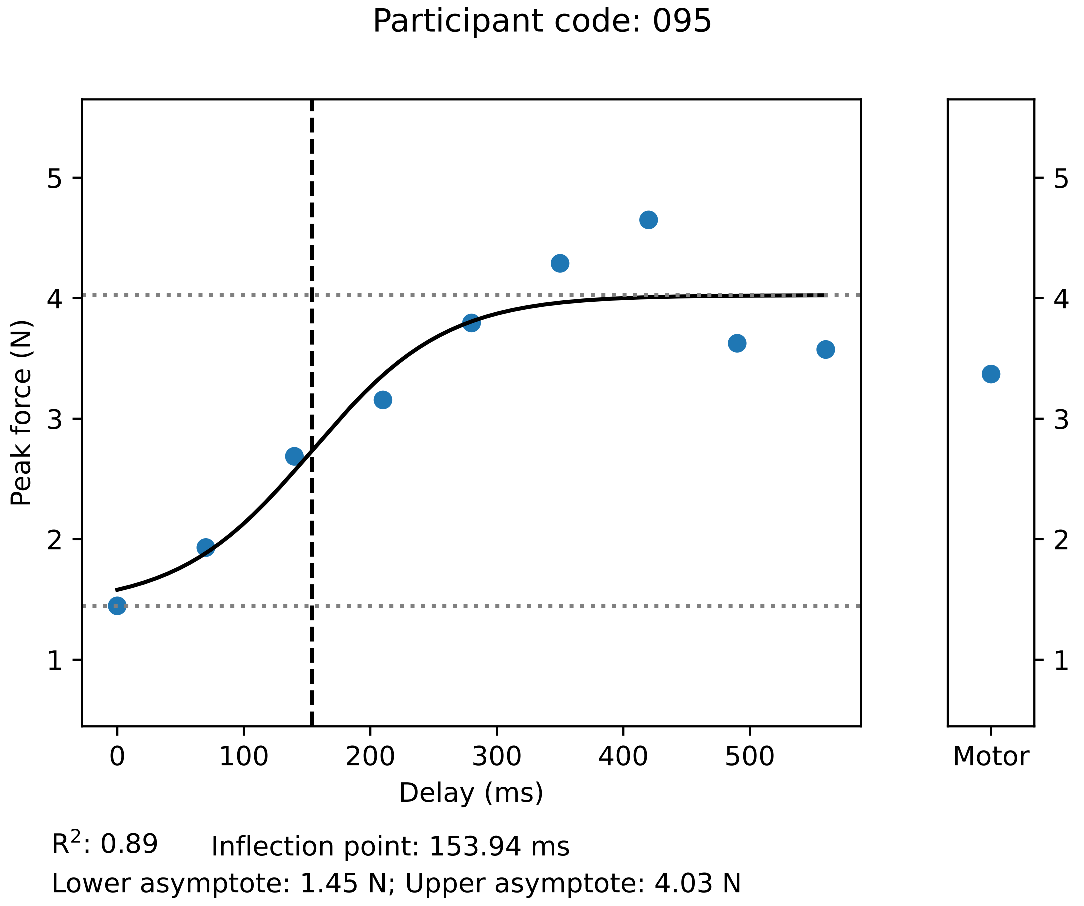
Participant code: 095
Peak force (N)
1
2
3
4
5
0
100
200
300
400
500
Delay (ms)
Motor
R2: 0.89
Inflection point: 153.94 ms
Lower asymptote: 1.45 N; Upper asymptote: 4.03 N

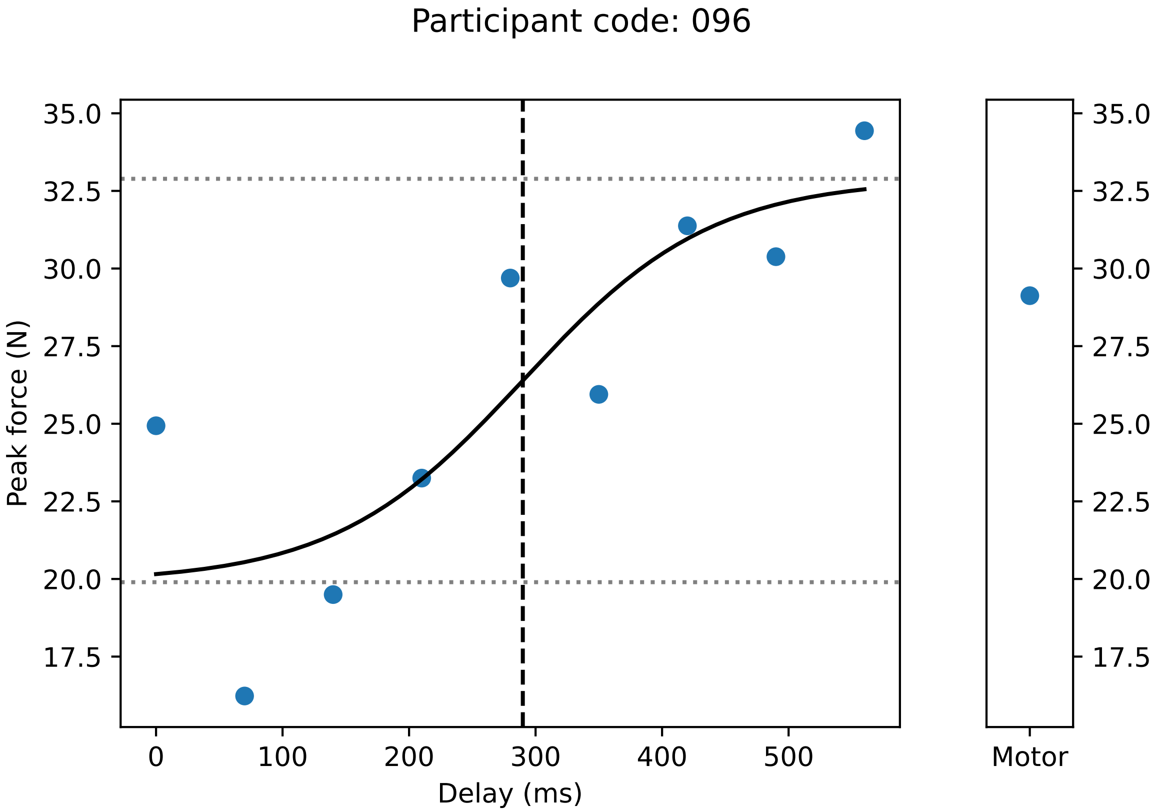

R^2: 0.73 Inflection point: 289.92 ms

Lower asymptote: 19.9 N; Upper asymptote: 32.89 N

Participant code: 103

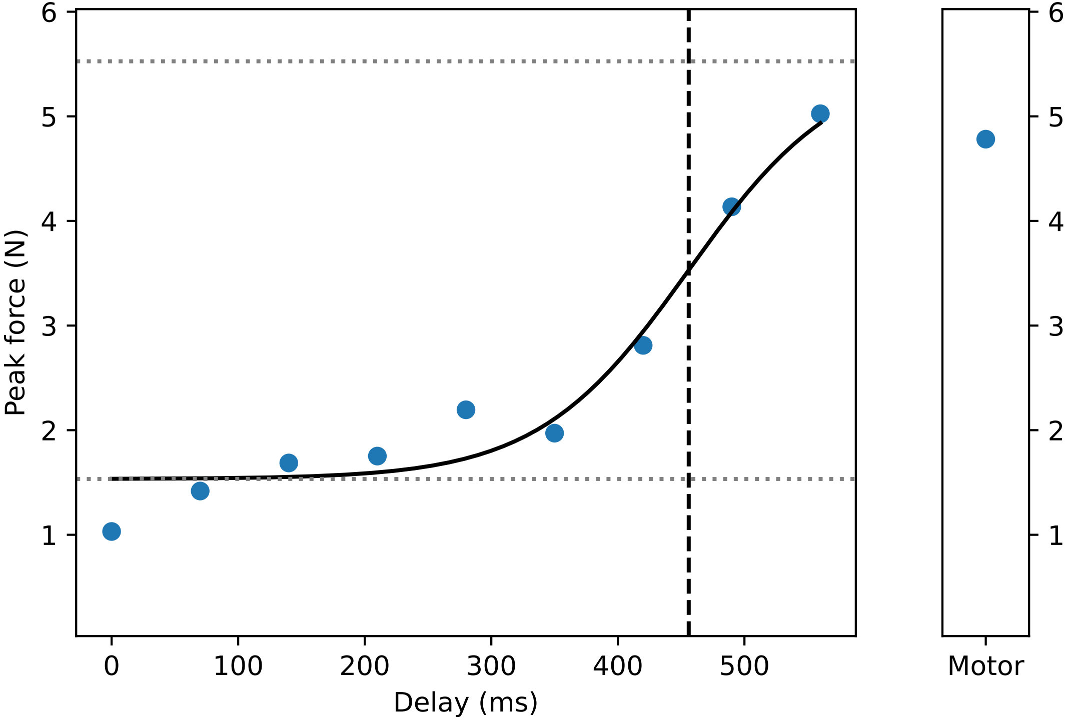

R^2: 0.96 Inflection point: 455.98 ms

Lower asymptote: 1.53 N; Upper asymptote: 5.53 N

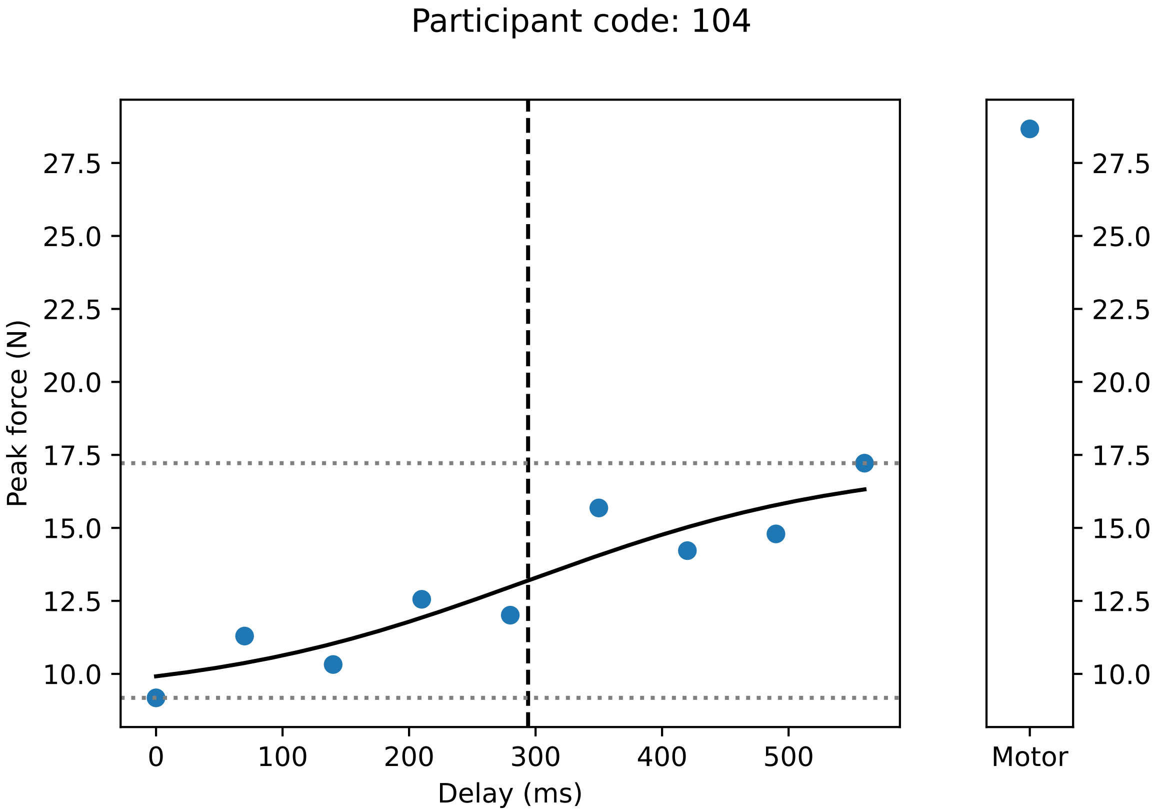

R^2: 0.85 Inflection point: 294.09 ms
Lower asymptote: 9.18 N; Upper asymptote: 17.22 N

Participant code: 110

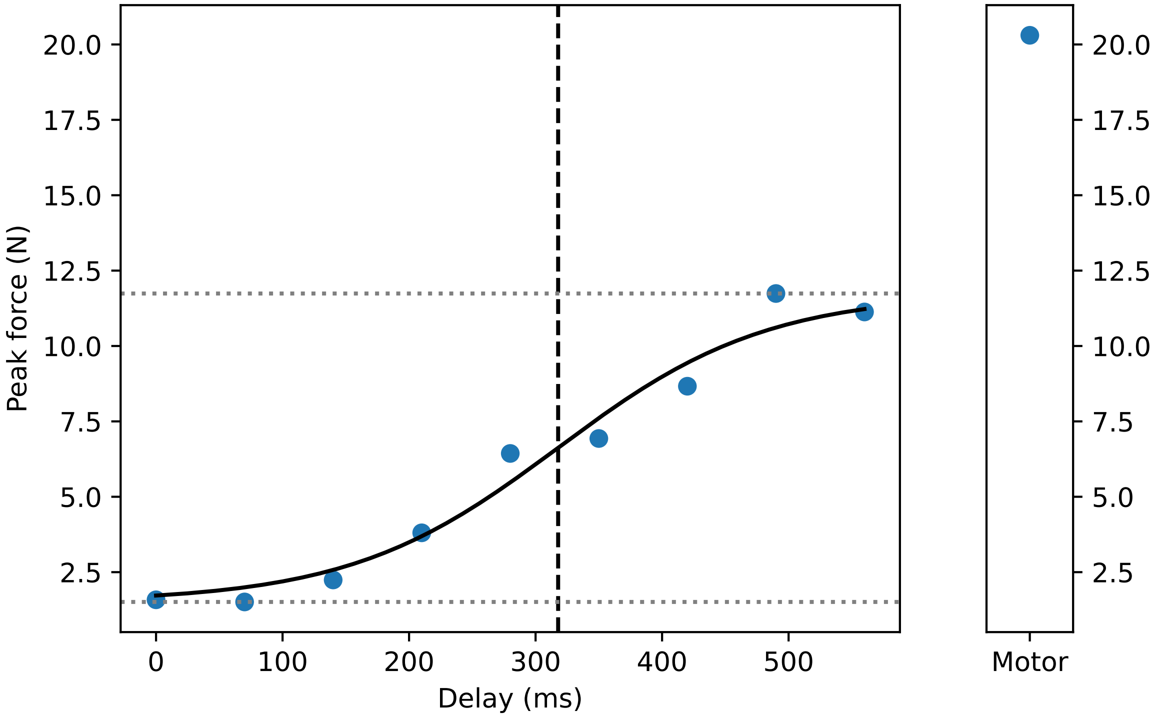

R^2: 0.97 Inflection point: 317.84 ms

Lower asymptote: 1.51 N; Upper asymptote: 11.74 N

Participant code: 111 !

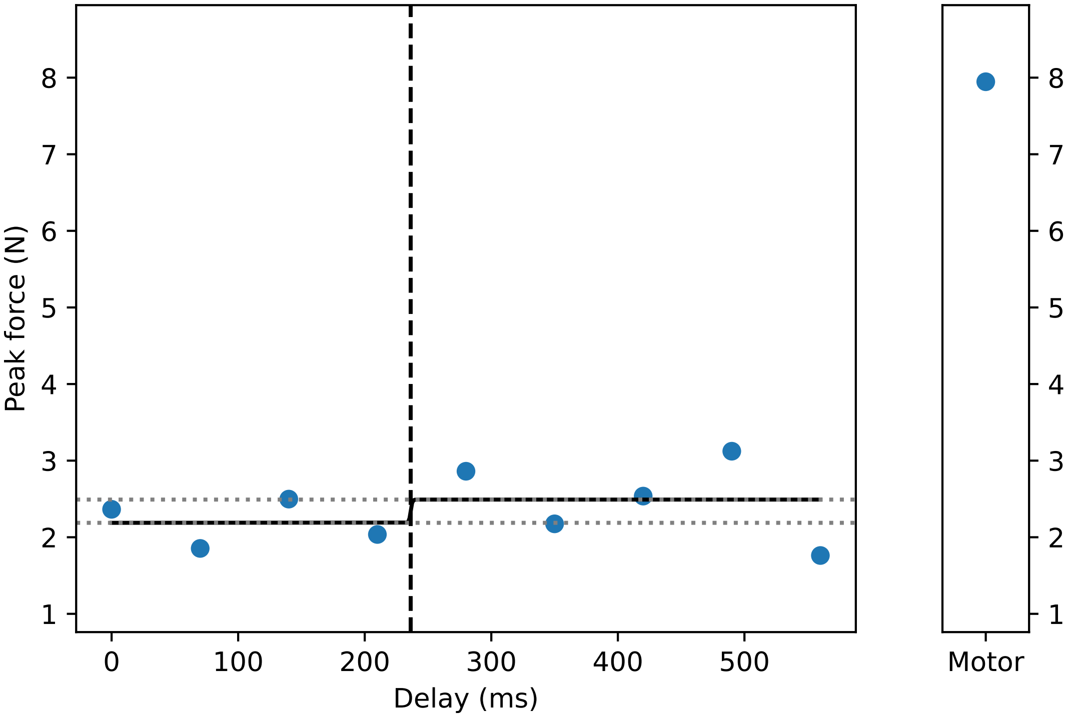

R^2: 0.13 ! Inflection point: 236.3 ms

Lower asymptote: 2.19 N; Upper asymptote: 2.49 N

Participant code: 112

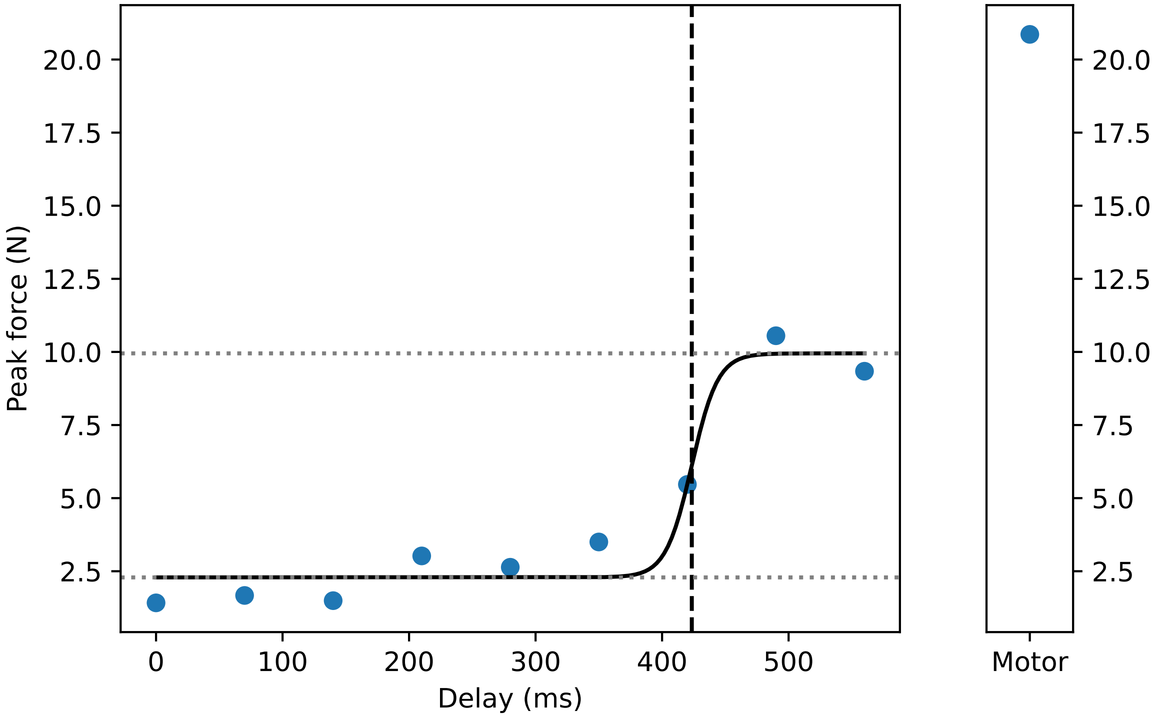

R^2: 0.95 Inflection point: 423.5 ms

Lower asymptote: 2.29 N; Upper asymptote: 9.95 N

Participant code: 113

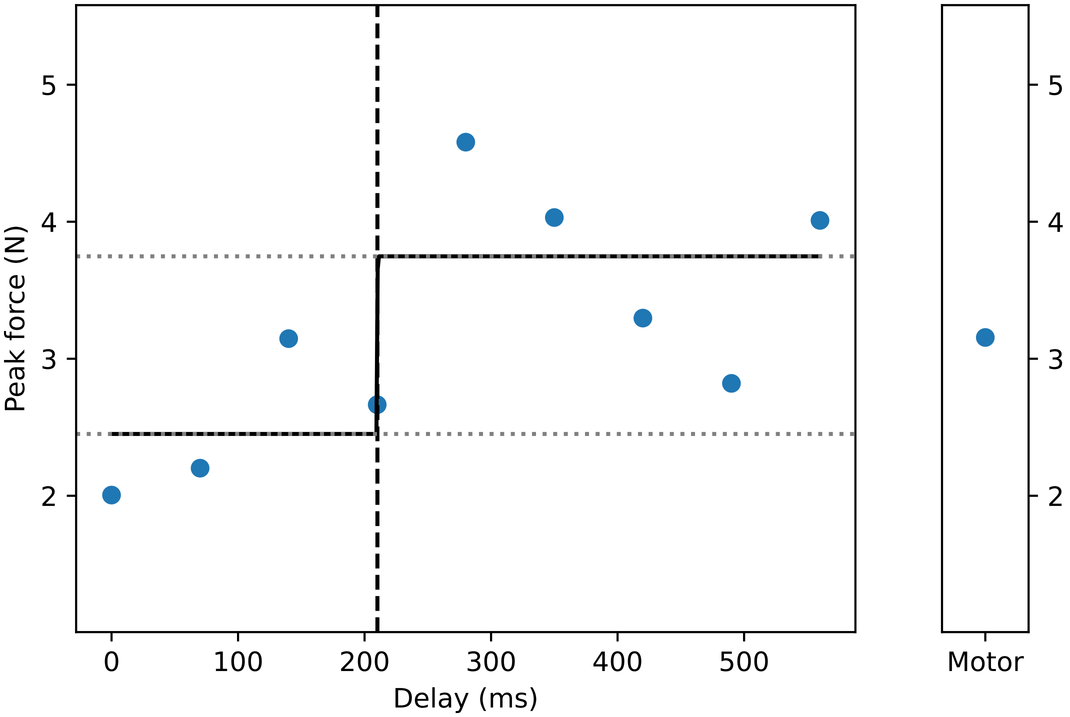

R^2: 0.57 Inflection point: 210.15 ms

Lower asymptote: 2.45 N; Upper asymptote: 3.75 N

Participant code: 116

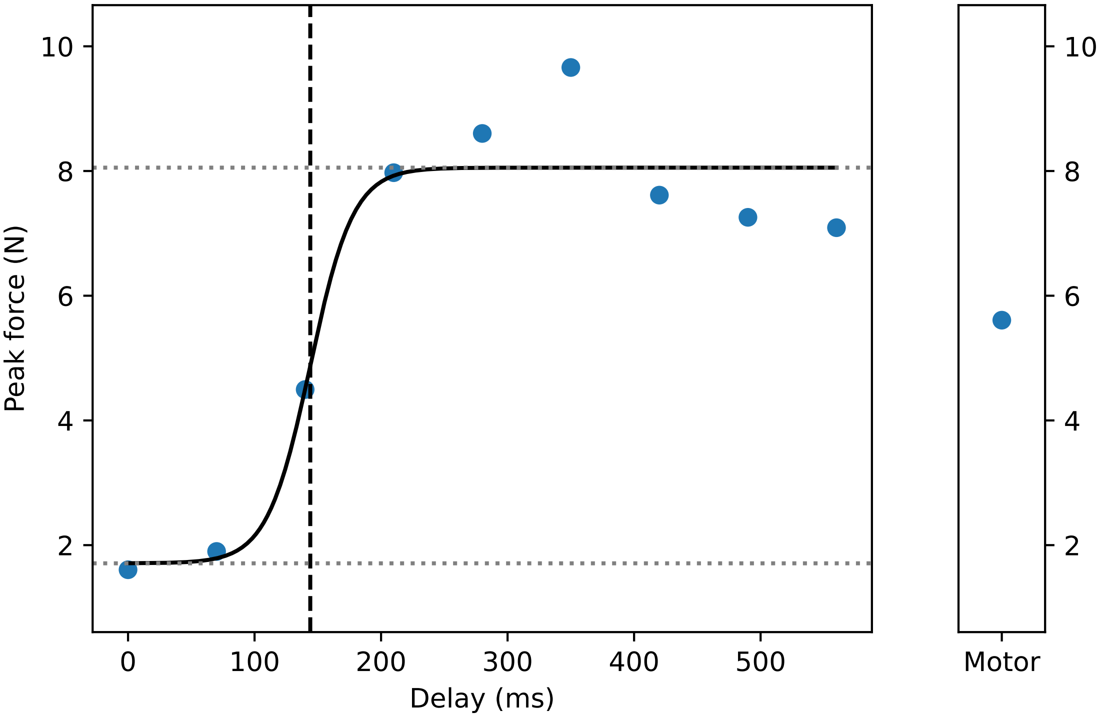

R^2: 0.93 Inflection point: 144.04 ms

Lower asymptote: 1.71 N; Upper asymptote: 8.05 N

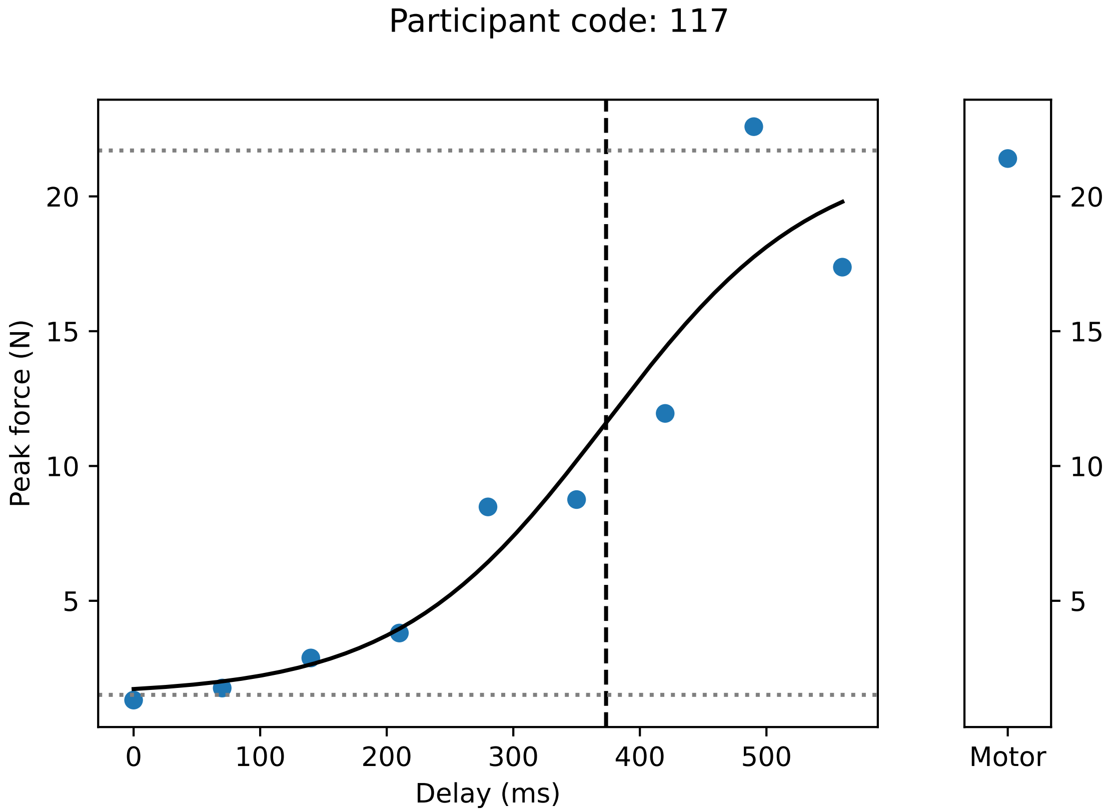

R^2: 0.9 Inflection point: 373.29 ms

Lower asymptote: 1.51 N; Upper asymptote: 21.7 N

Participant code: 122 !

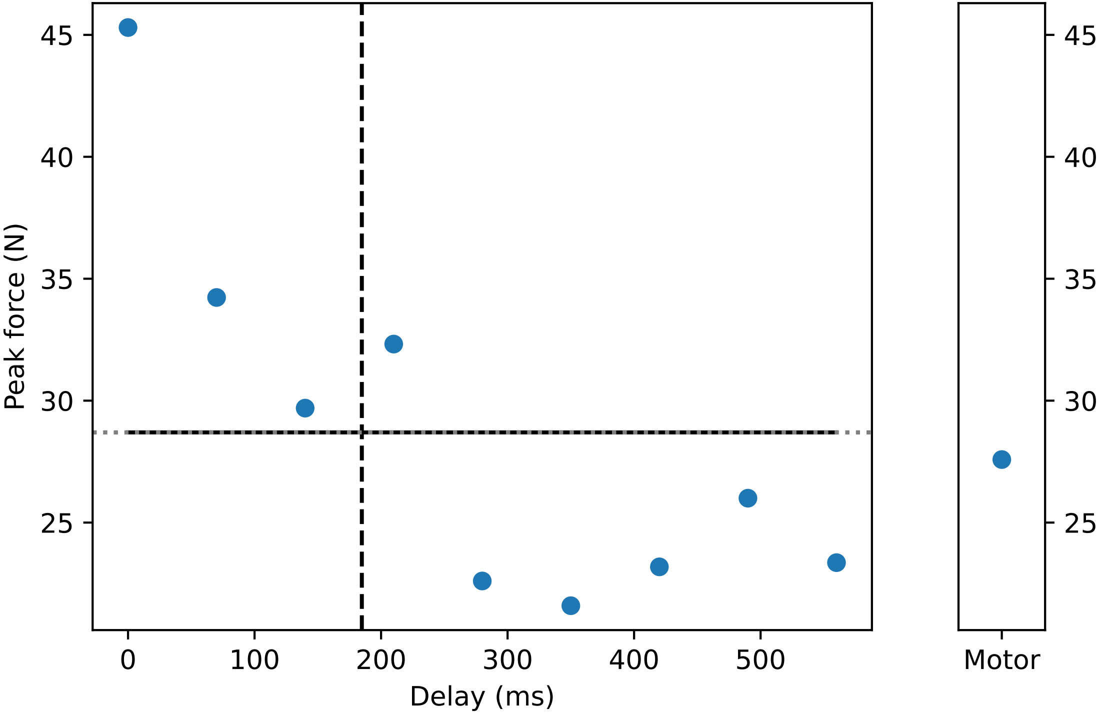

R^2: 0.0 ! Inflection point: 184.8 ms

Lower asymptote: 28.7 N; Upper asymptote: 28.7 N

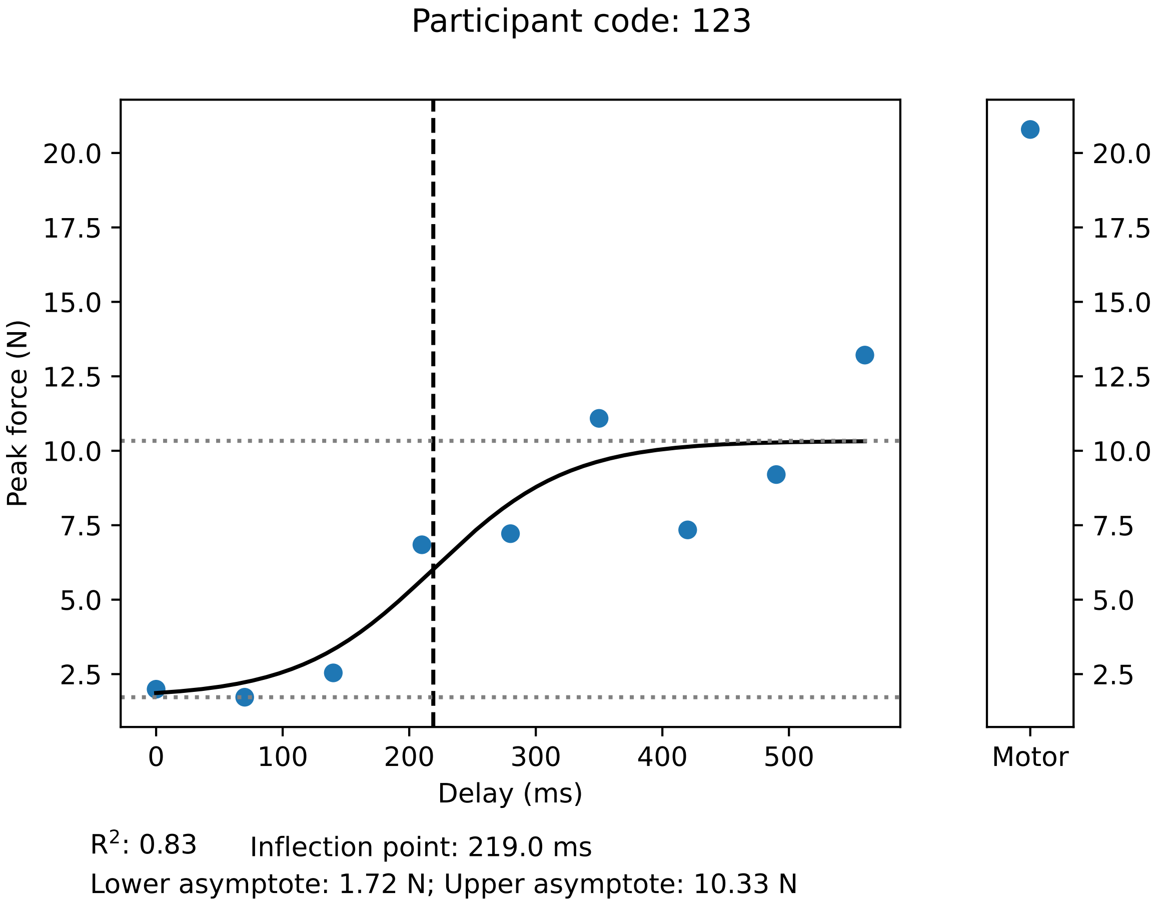

Participant code: 123
Peak force (N)
20.0
17.5
15.0
12.5
10.0
7.5
5.0
2.5
0
100
200
300
400
500
Delay (ms)
Motor
R^2: 0.83
Inflection point: 219.0 ms
Lower asymptote: 1.72 N; Upper asymptote: 10.33 N

Participant code: 128

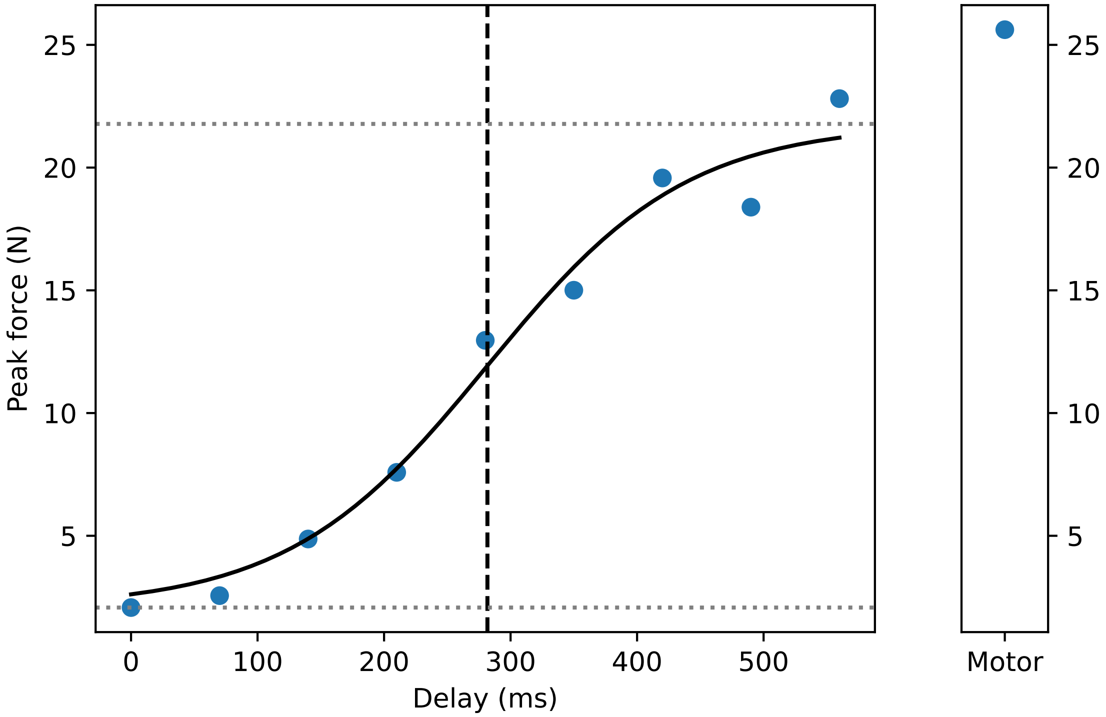

R^2: 0.98 Inflection point: 281.7 ms

Lower asymptote: 2.08 N; Upper asymptote: 21.78 N

Participant code: 129

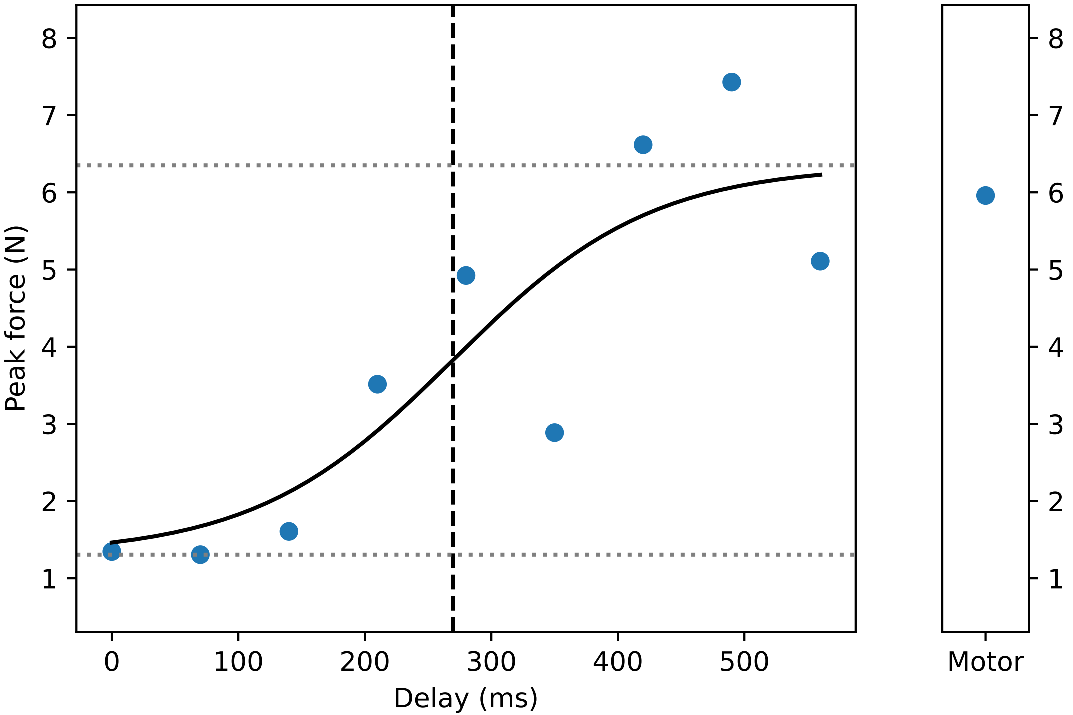

R^2: 0.76 Inflection point: 269.67 ms

Lower asymptote: 1.31 N; Upper asymptote: 6.35 N

Participant code: 134

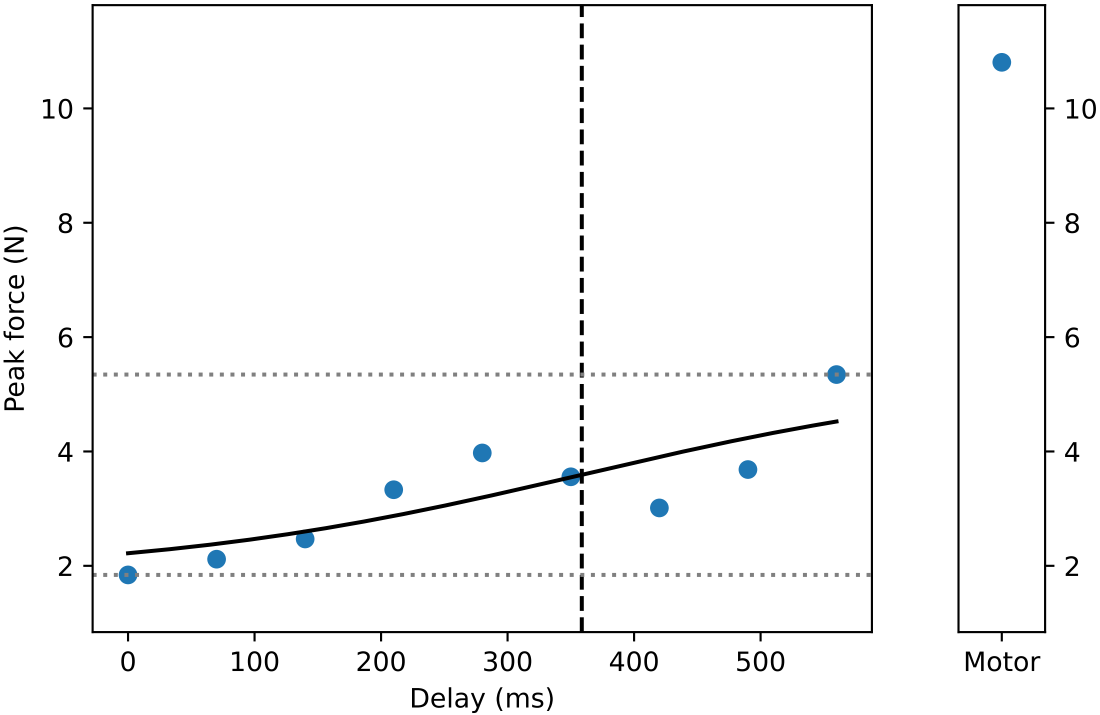

R^2: 0.69 Inflection point: 358.67 ms

Lower asymptote: 1.84 N; Upper asymptote: 5.35 N

Participant code: 141

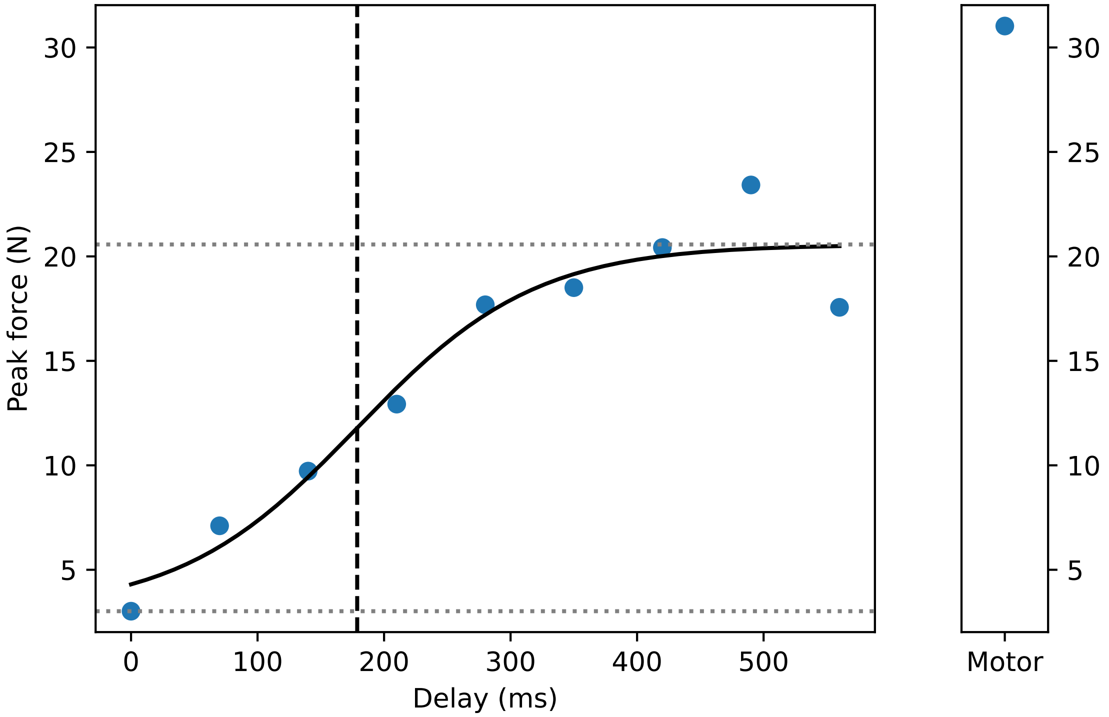

R^2: 0.94 Inflection point: 178.76 ms

Lower asymptote: 3.02 N; Upper asymptote: 20.57 N

Participant code: 143

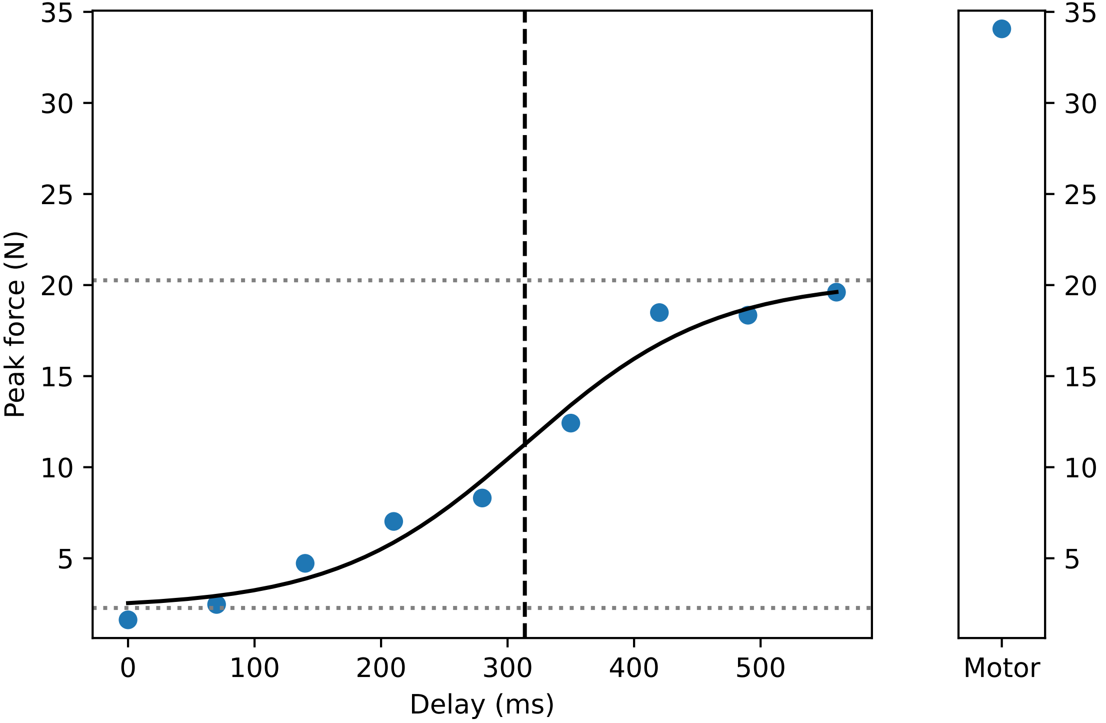

R^2: 0.98   Inflection point: 313.65 ms

Lower asymptote: 2.27 N; Upper asymptote: 20.26 N

Participant code: 147 !

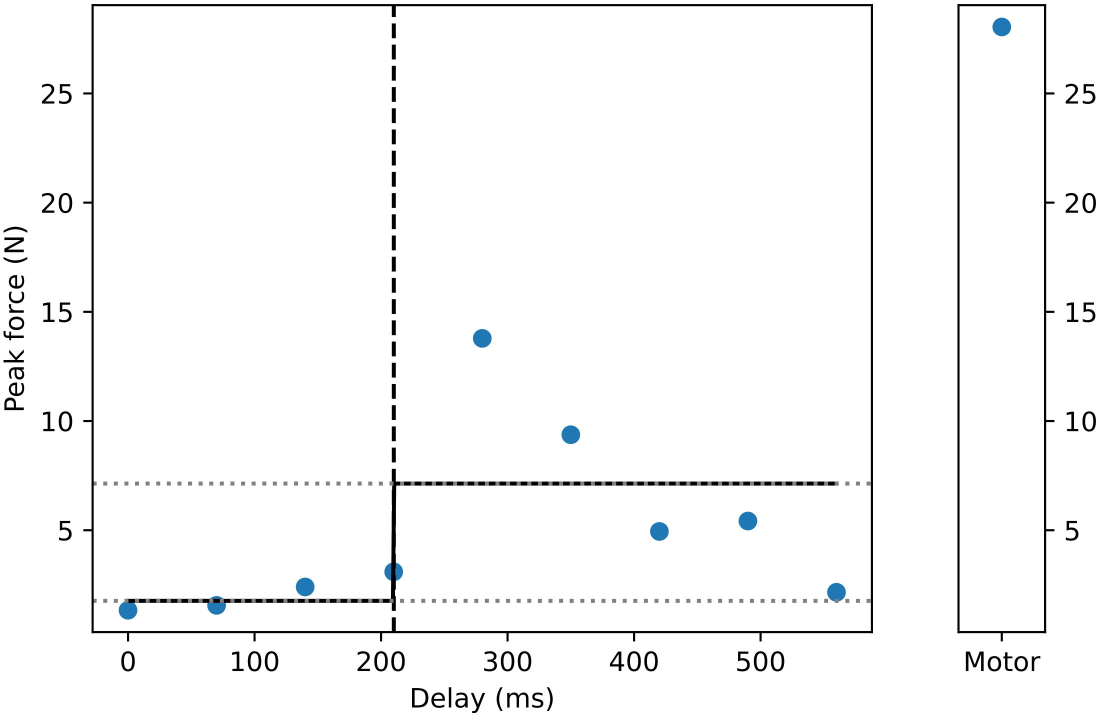

R^2: 0.41 !   Inflection point: 210.08 ms

Lower asymptote: 1.77 N; Upper asymptote: 7.14 N

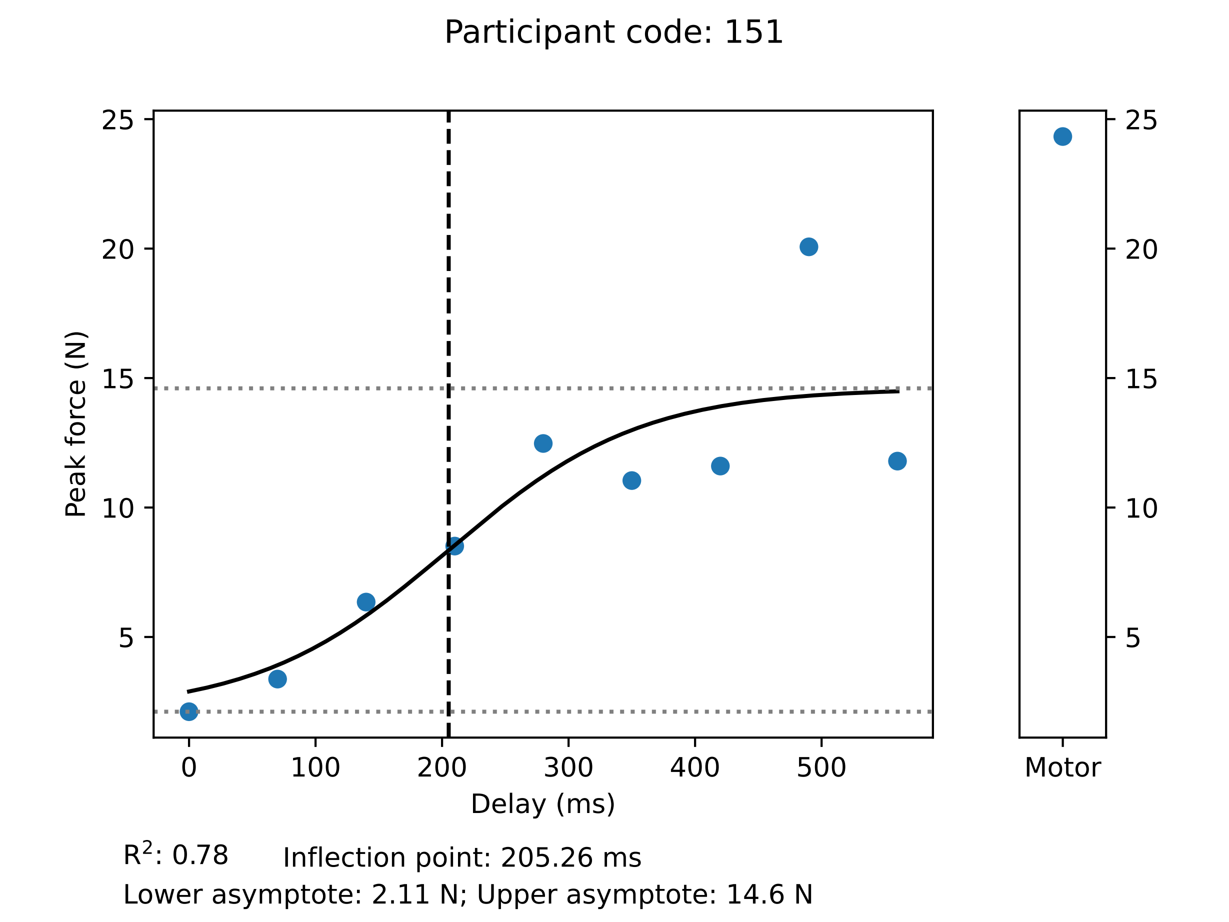
Participant code: 151
Peak force (N)
25
20
15
10
5
0
100
200
300
400
500
Delay (ms)
Motor
R²: 0.78
Inflection point: 205.26 ms
Lower asymptote: 2.11 N; Upper asymptote: 14.6 N

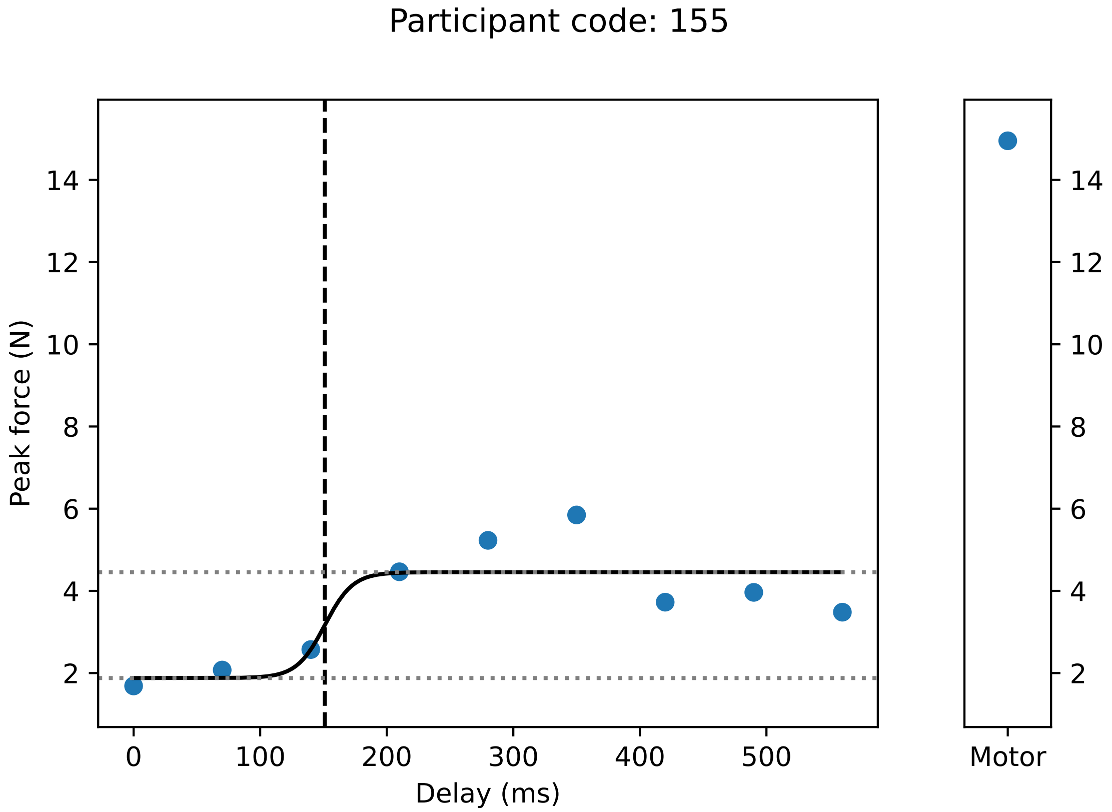

R^2: 0.72 Inflection point: 151.07 ms
Lower asymptote: 1.88 N; Upper asymptote: 4.45 N

Participant code: 159

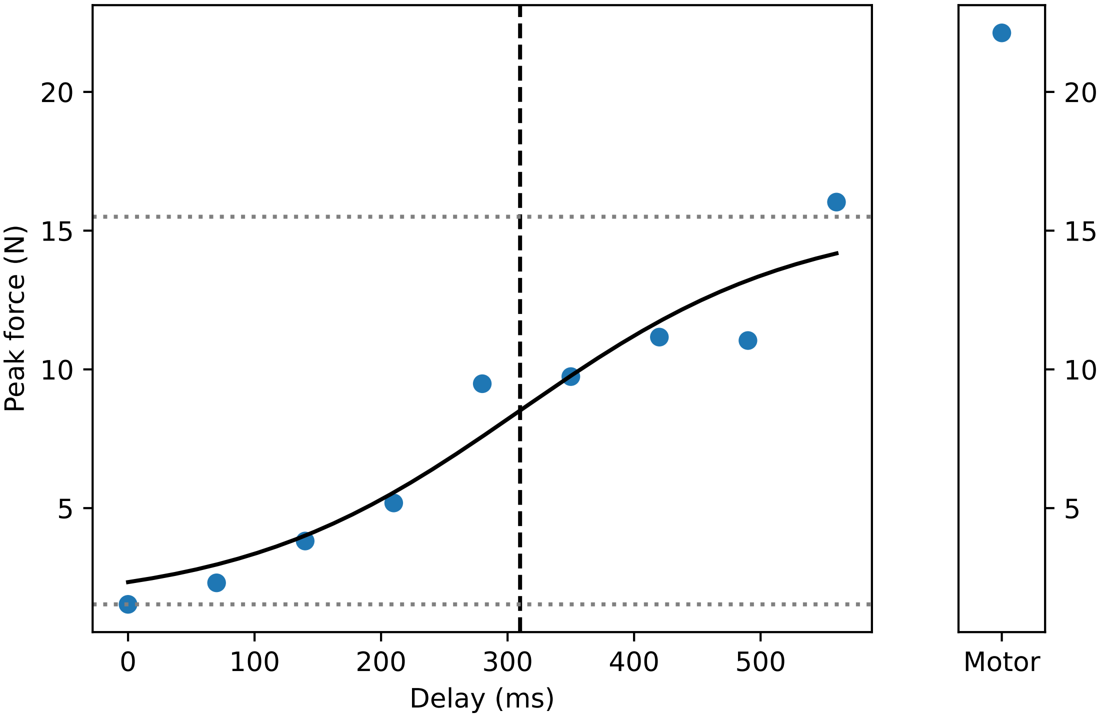

R^2: 0.93 Inflection point: 309.89 ms

Lower asymptote: 1.53 N; Upper asymptote: 15.5 N

Participant code: 163

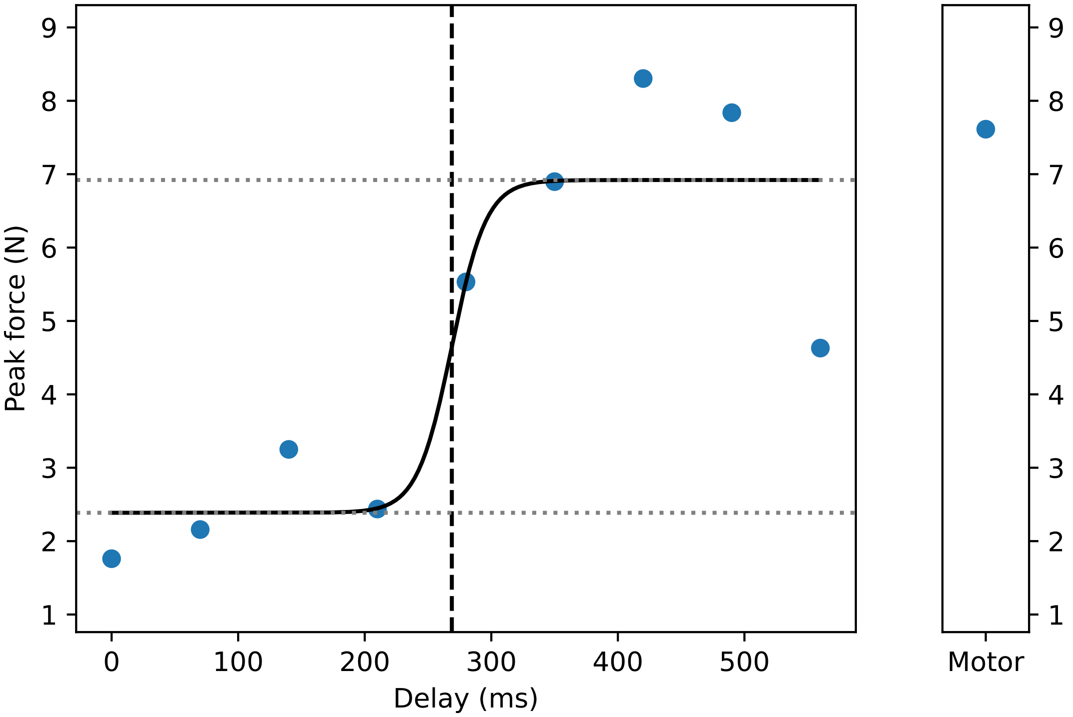

R^2: 0.82 Inflection point: 268.81 ms

Lower asymptote: 2.39 N; Upper asymptote: 6.92 N

Participant code: 167

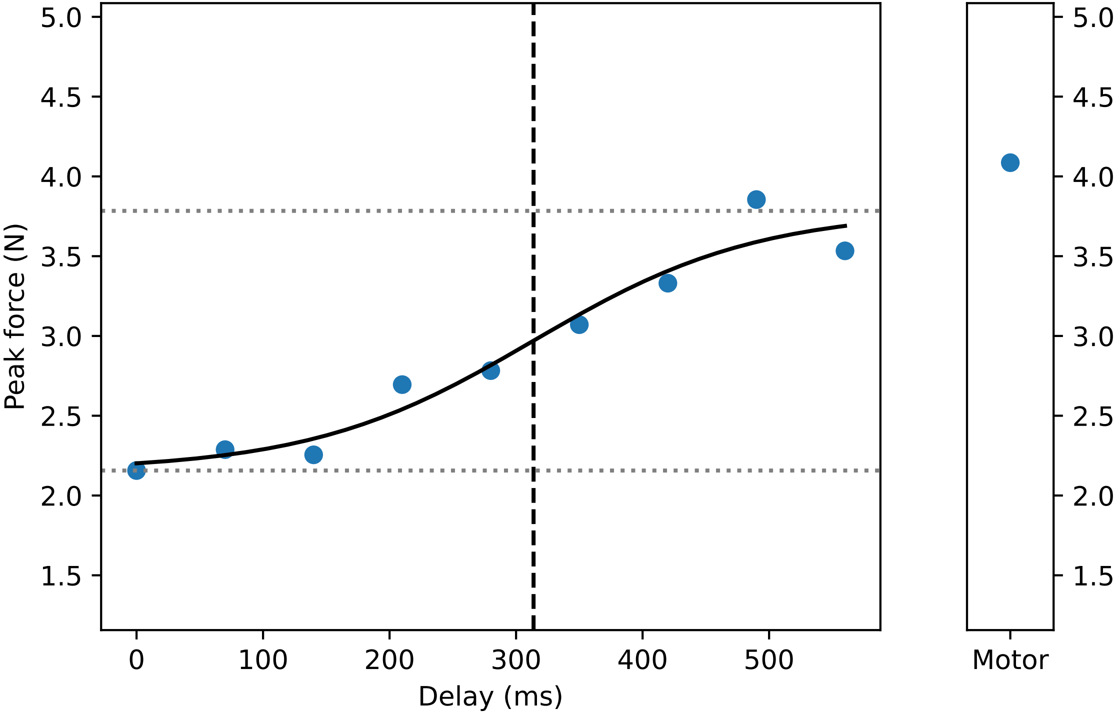

R^2: 0.95 Inflection point: 313.82 ms

Lower asymptote: 2.16 N; Upper asymptote: 3.78 N

Participant code: 174

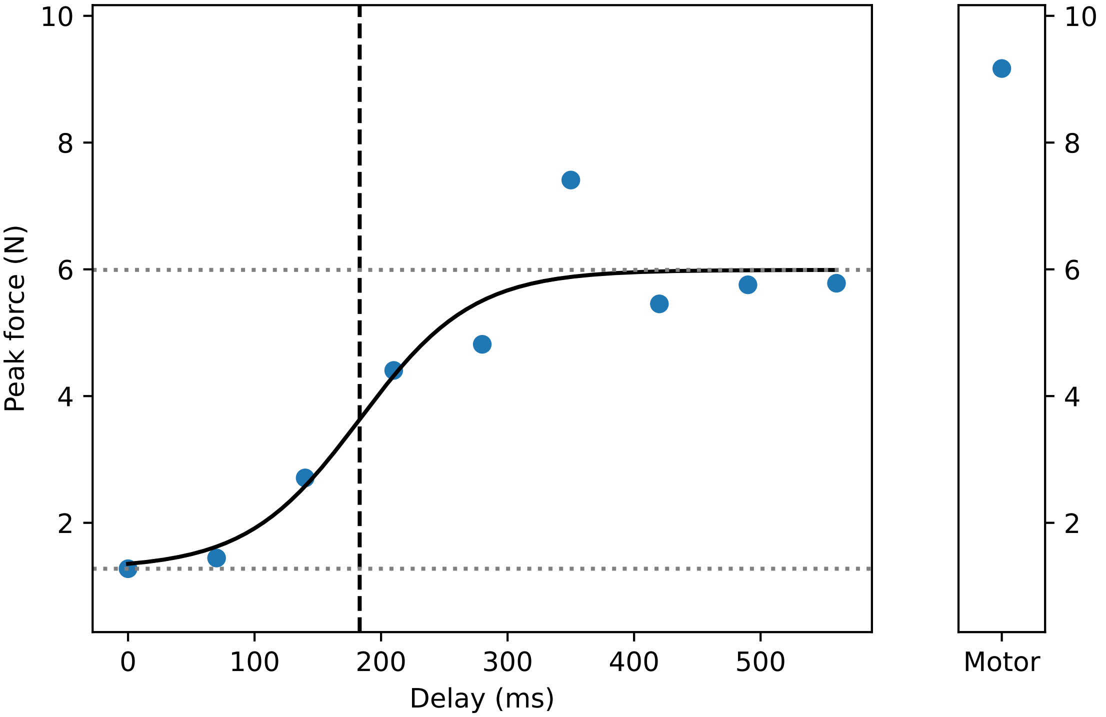

R^2: 0.91 Inflection point: 183.1 ms

Lower asymptote: 1.28 N; Upper asymptote: 5.99 N

Participant code: 177 !

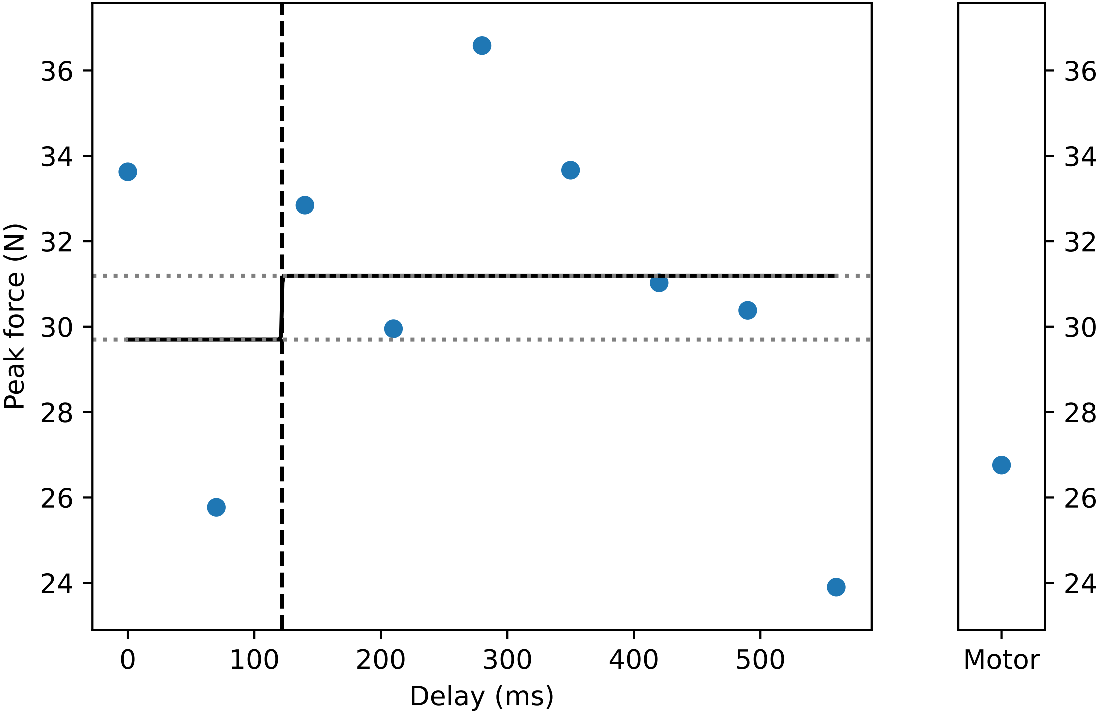

R^2: 0.03 ! Inflection point: 121.77 ms

Lower asymptote: 29.7 N; Upper asymptote: 31.19 N

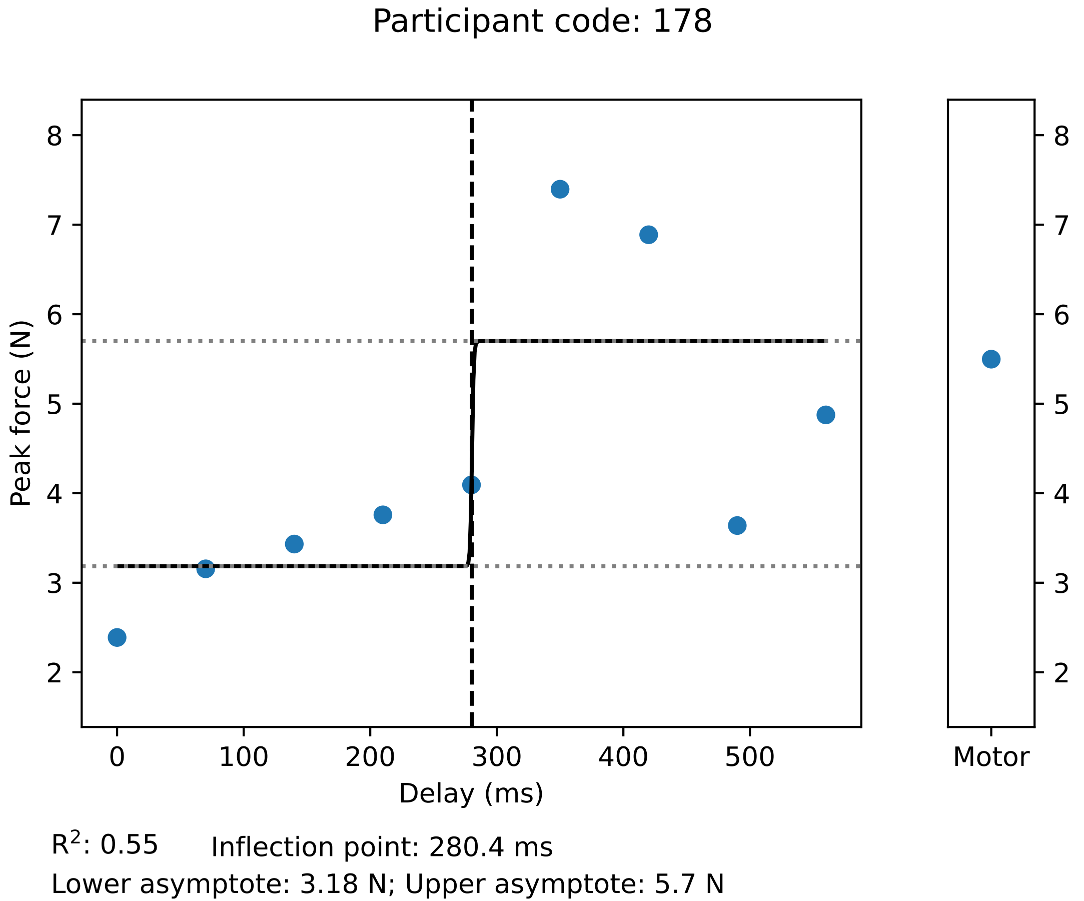
Participant code: 178
Peak force (N)
8
7
6
5
4
3
2
0
100
200
300
400
500
Delay (ms)
Motor
R²: 0.55
Inflection point: 280.4 ms
Lower asymptote: 3.18 N; Upper asymptote: 5.7 N

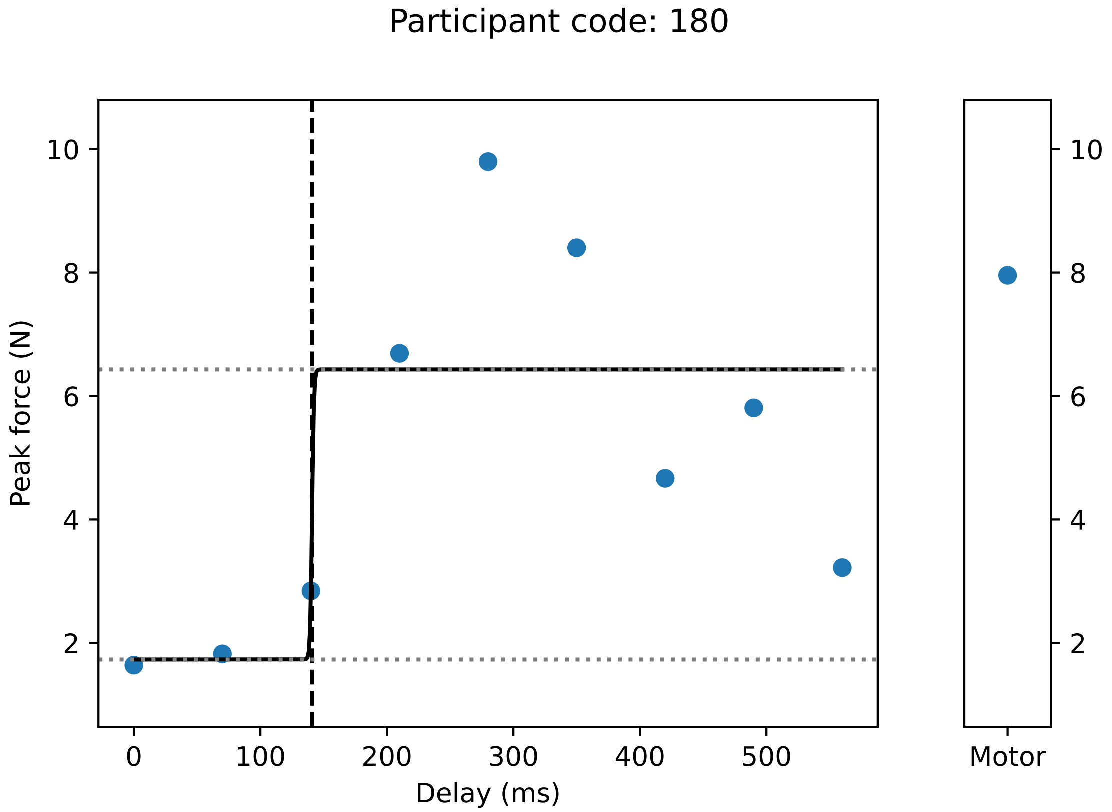

R^2: 0.57 Inflection point: 140.86 ms

Lower asymptote: 1.73 N; Upper asymptote: 6.43 N

Participant code: 183

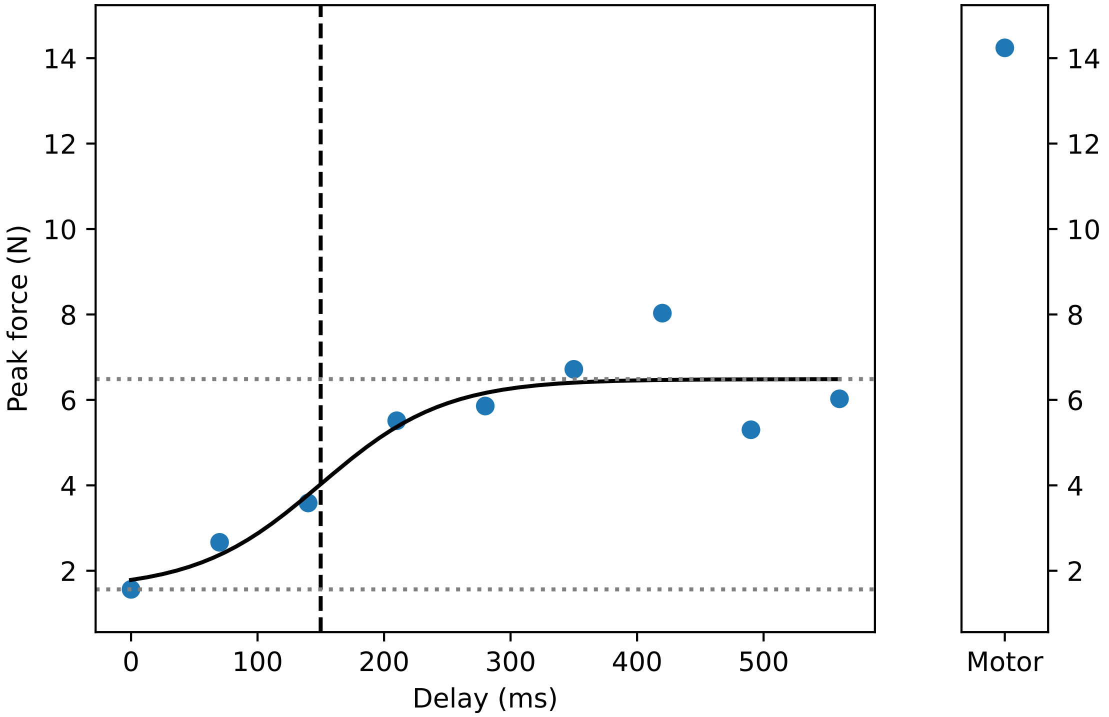

R^2: 0.87 Inflection point: 149.9 ms

Lower asymptote: 1.56 N; Upper asymptote: 6.49 N

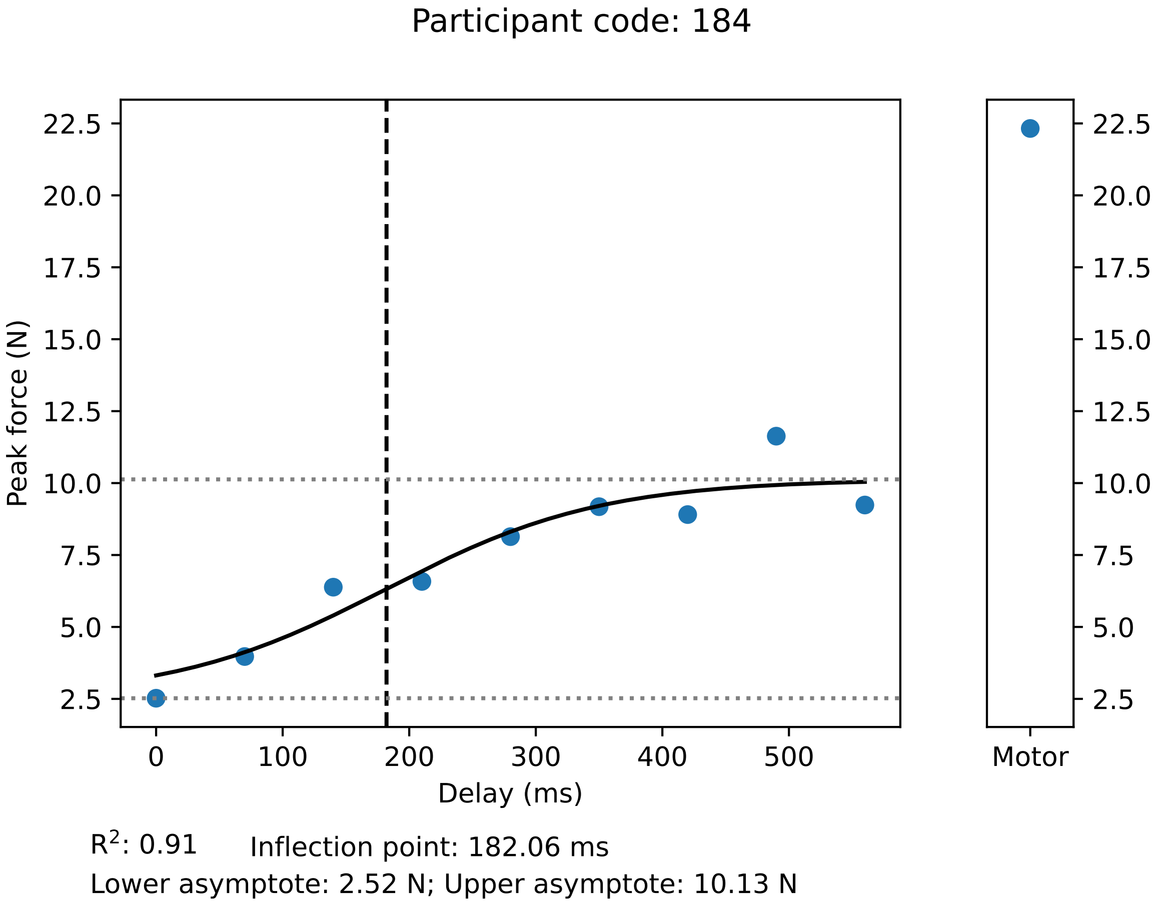
Participant code: 184
Peak force (N)
22.5
20.0
17.5
15.0
12.5
10.0
7.5
5.0
2.5
0
100
200
300
400
500
Delay (ms)
Motor
R^2: 0.91
Inflection point: 182.06 ms
Lower asymptote: 2.52 N; Upper asymptote: 10.13 N

Participant code: 185 !

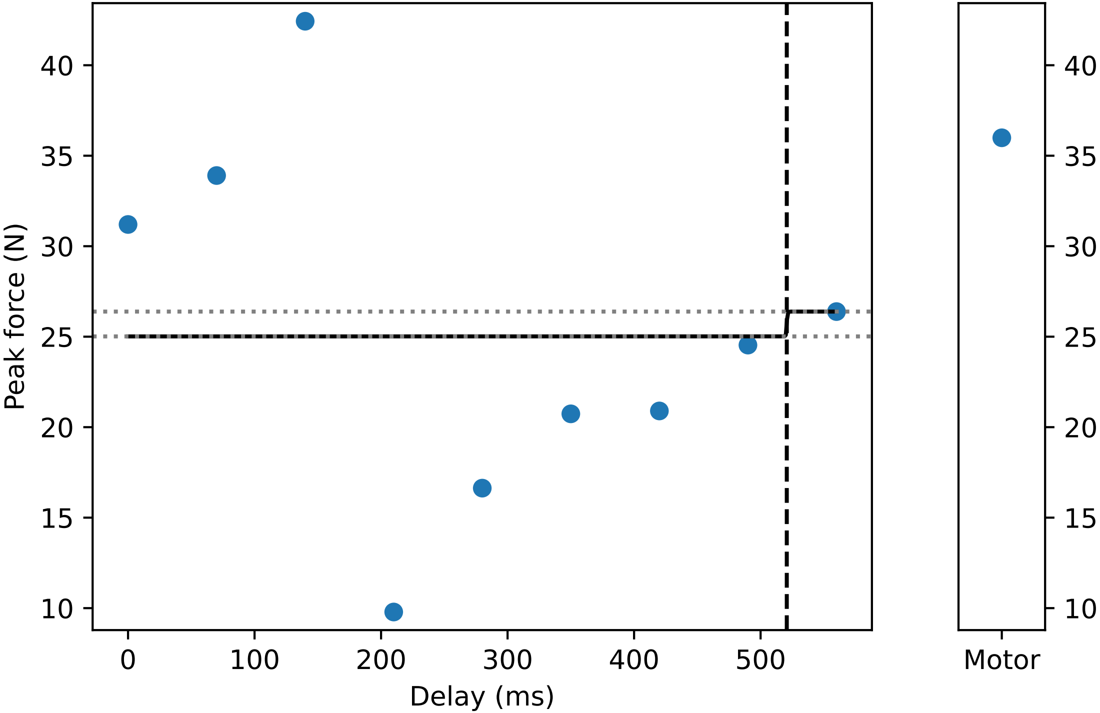

R^2: 0.0 !   Inflection point: 520.7 ms

Lower asymptote: 25.02 N; Upper asymptote: 26.39 N

Participant code: 190

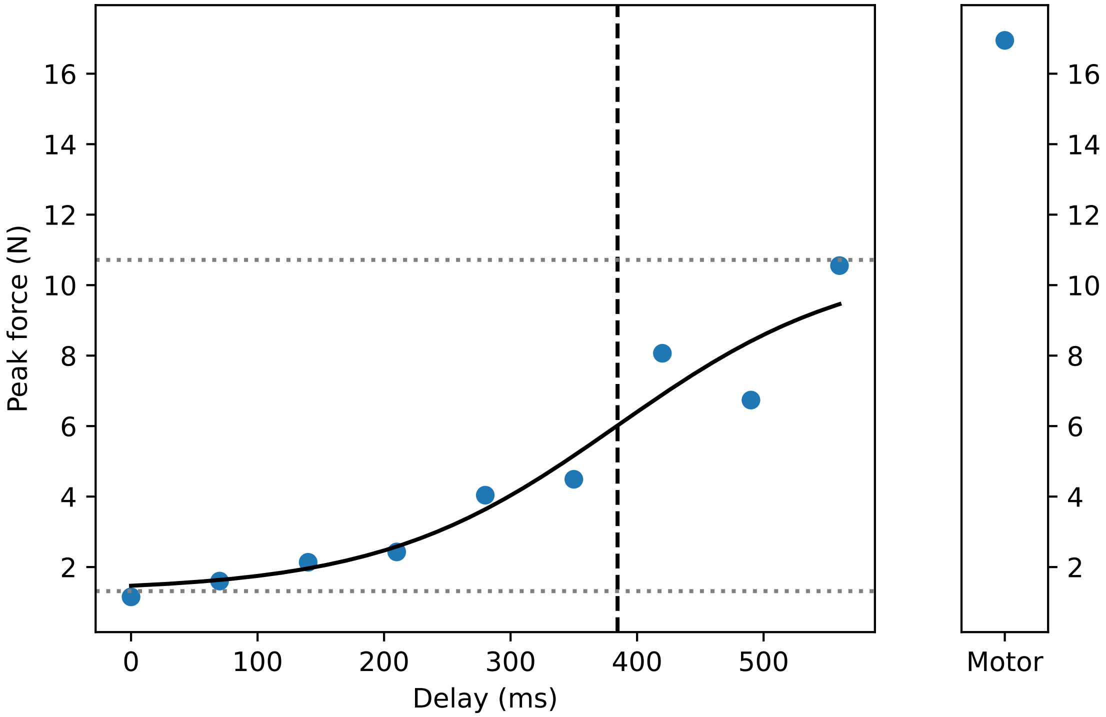

R^2: 0.93 Inflection point: 384.56 ms

Lower asymptote: 1.32 N; Upper asymptote: 10.72 N

Participant code: 200 !

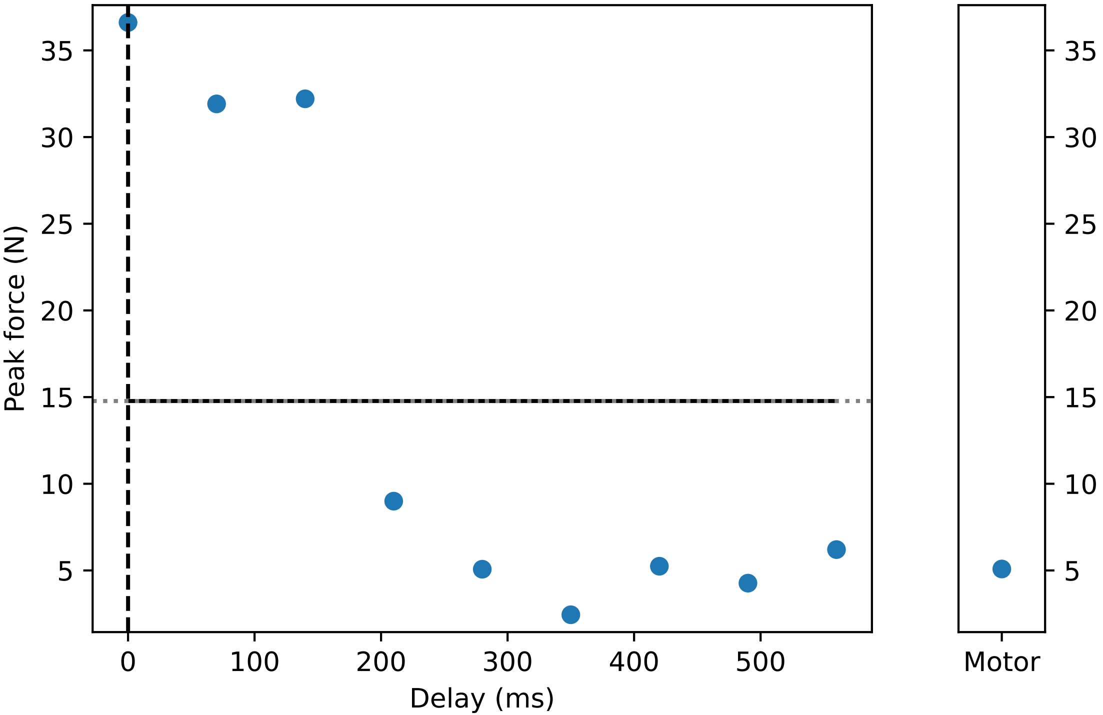

R^2: 0.0 ! Inflection point: 0.03 ms

Lower asymptote: 14.77 N; Upper asymptote: 14.77 N

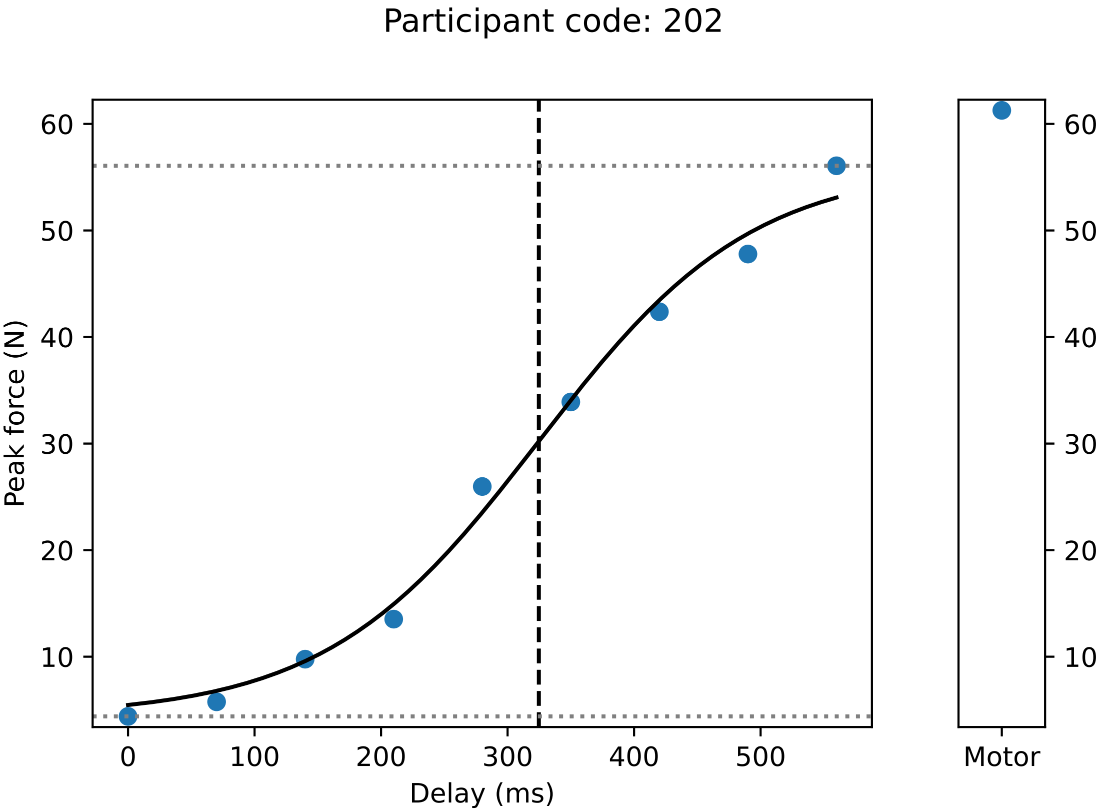

R^2: 0.99 Inflection point: 324.72 ms

Lower asymptote: 4.4 N; Upper asymptote: 56.07 N

Participant code: 203

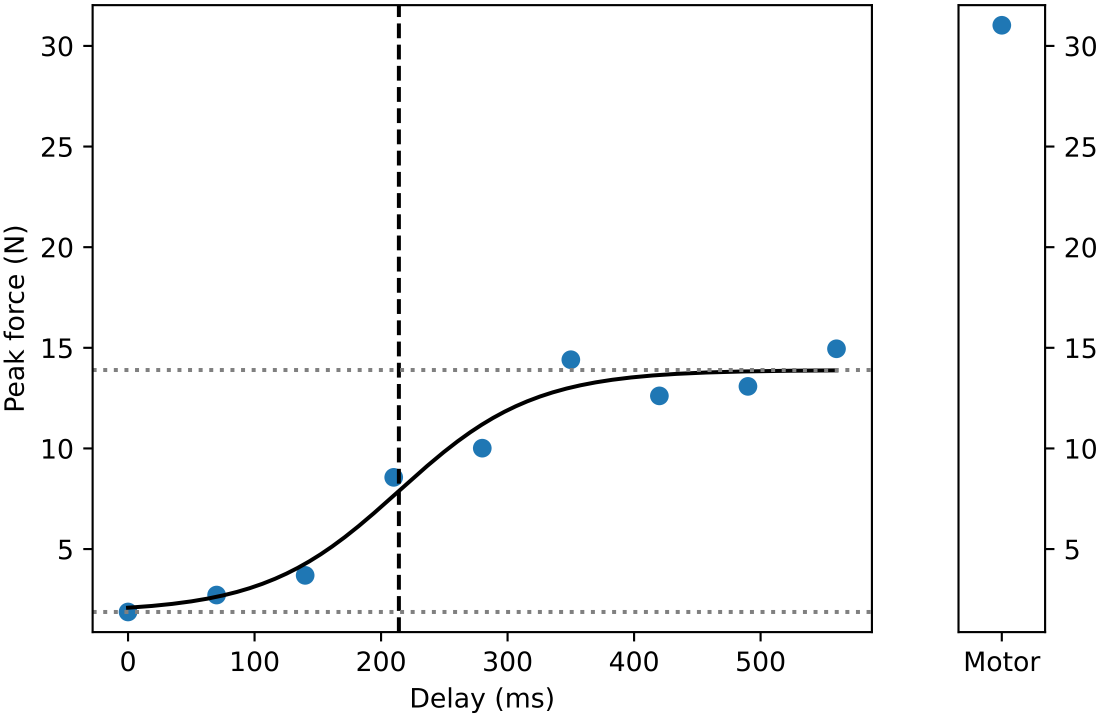

R^2: 0.97 Inflection point: 214.09 ms

Lower asymptote: 1.87 N; Upper asymptote: 13.9 N

Participant code: 211

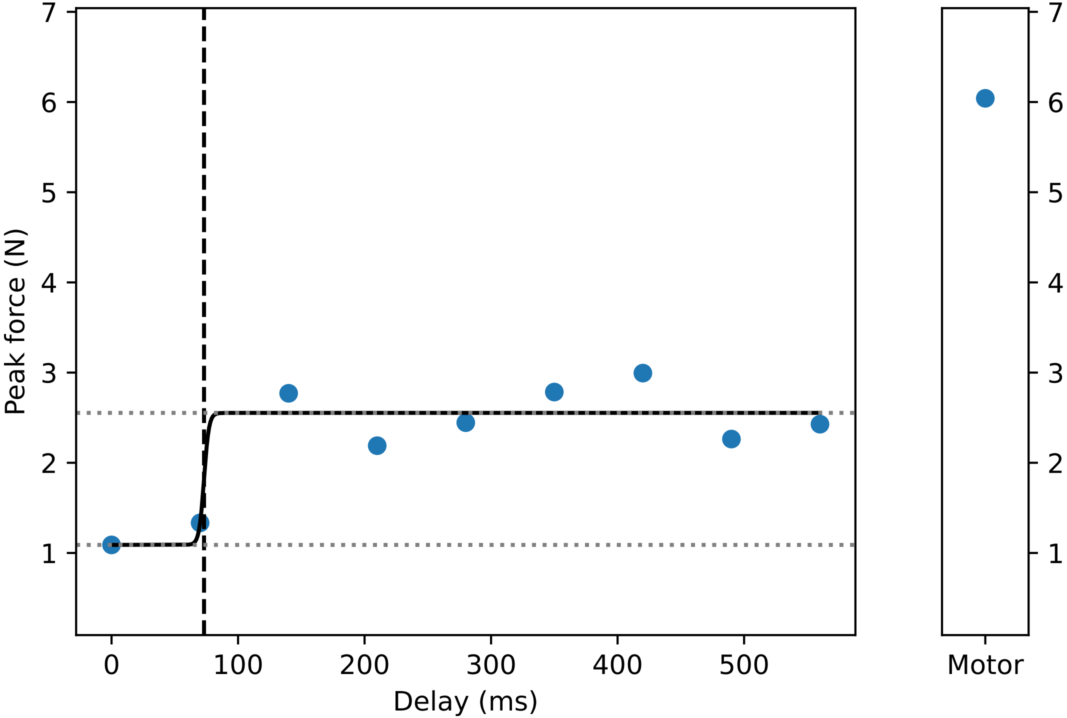

R^2: 0.84 Inflection point: 73.11 ms

Lower asymptote: 1.09 N; Upper asymptote: 2.55 N

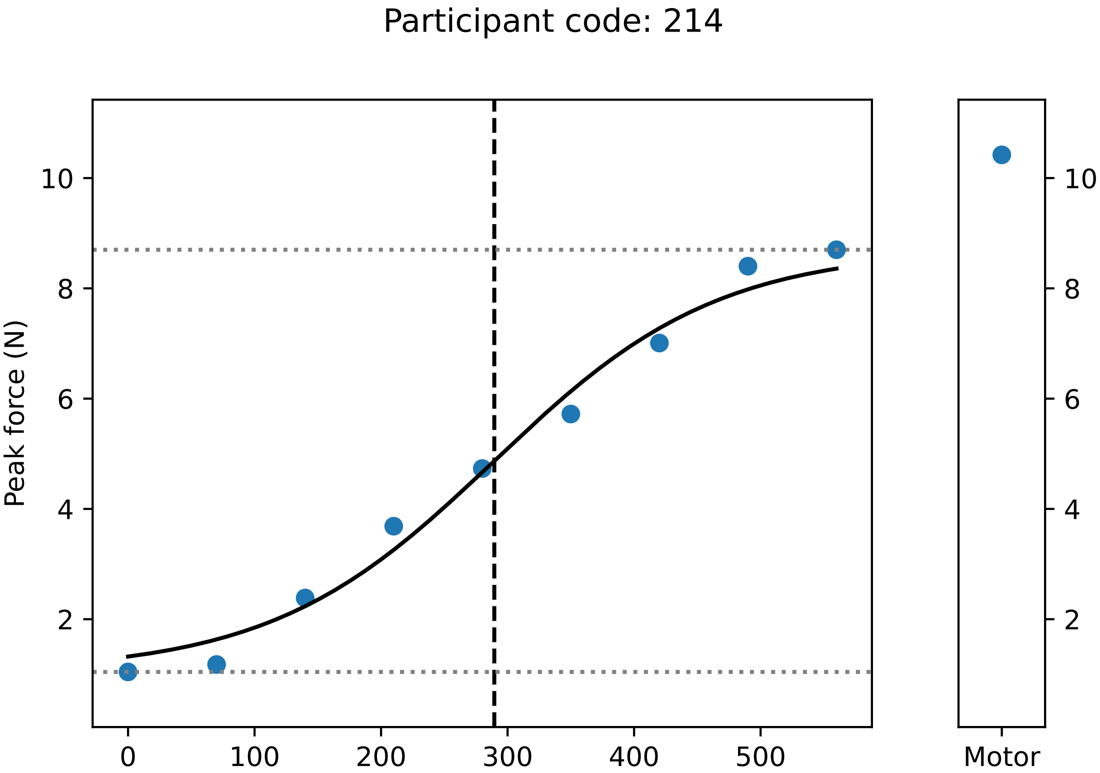

R^2: 0.98 Inflection point: 289.51 ms
Lower asymptote: 1.04 N; Upper asymptote: 8.7 N

Participant code: 217

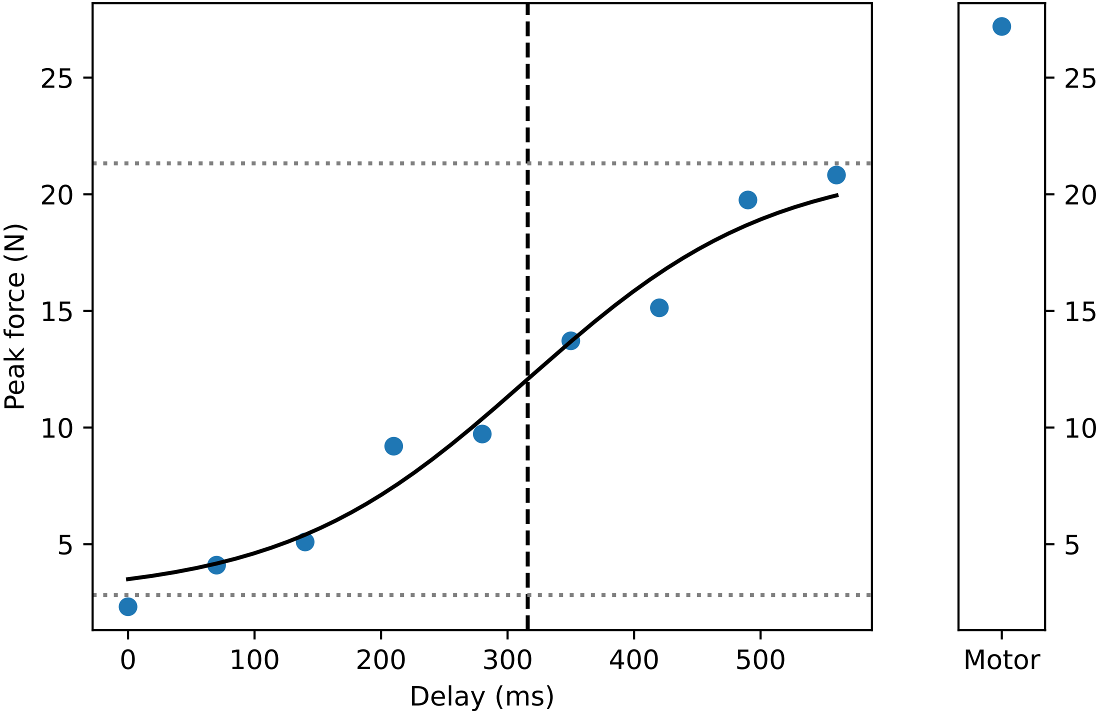

R^2: 0.97 Inflection point: 315.92 ms
Lower asymptote: 2.82 N; Upper asymptote: 21.33 N

Participant code: 218

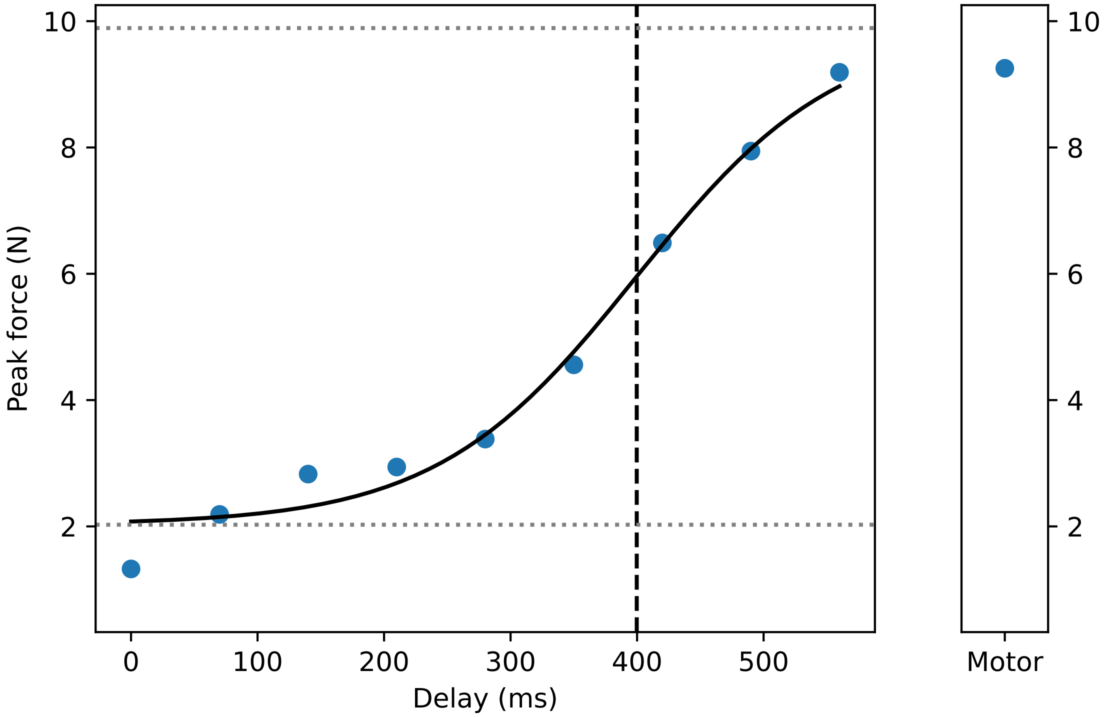

R^2: 0.98   Inflection point: 399.73 ms

Lower asymptote: 2.03 N; Upper asymptote: 9.89 N

Participant code: 219

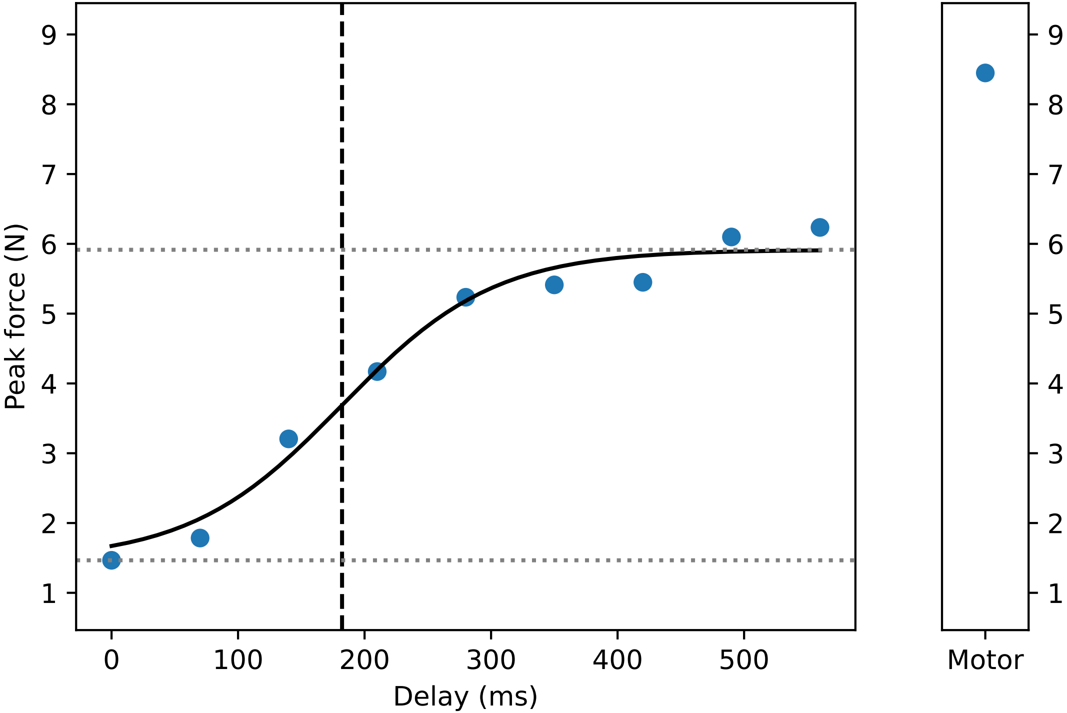

R^2: 0.98 Inflection point: 182.21 ms

Lower asymptote: 1.47 N; Upper asymptote: 5.91 N

Participant code: 220 !

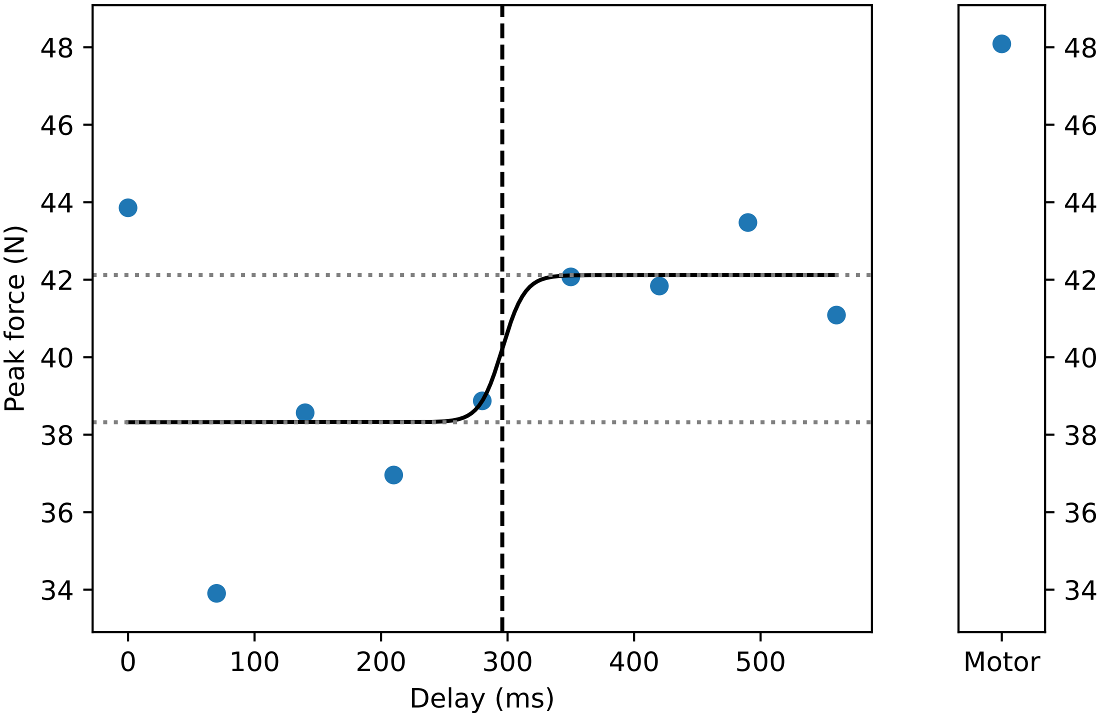

R^2: 0.36 ! Inflection point: 295.83 ms

Lower asymptote: 38.32 N; Upper asymptote: 42.12 N

Participant code: 225

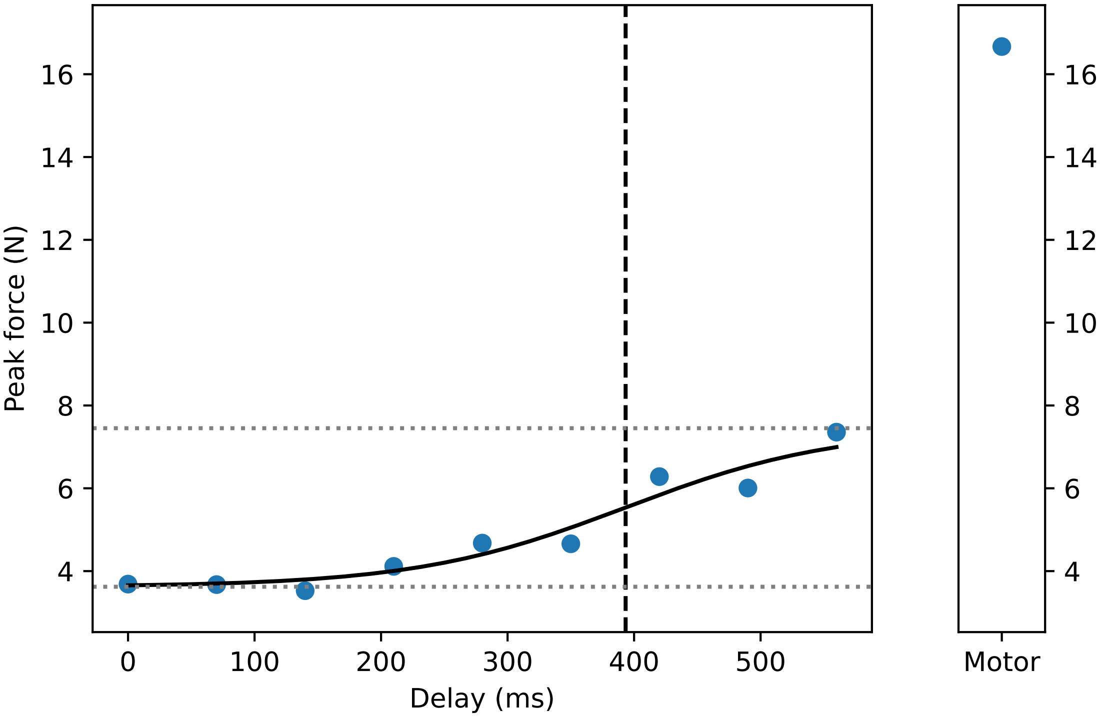

R^2: 0.94 Inflection point: 393.32 ms

Lower asymptote: 3.62 N; Upper asymptote: 7.45 N

Participant code: 231 !

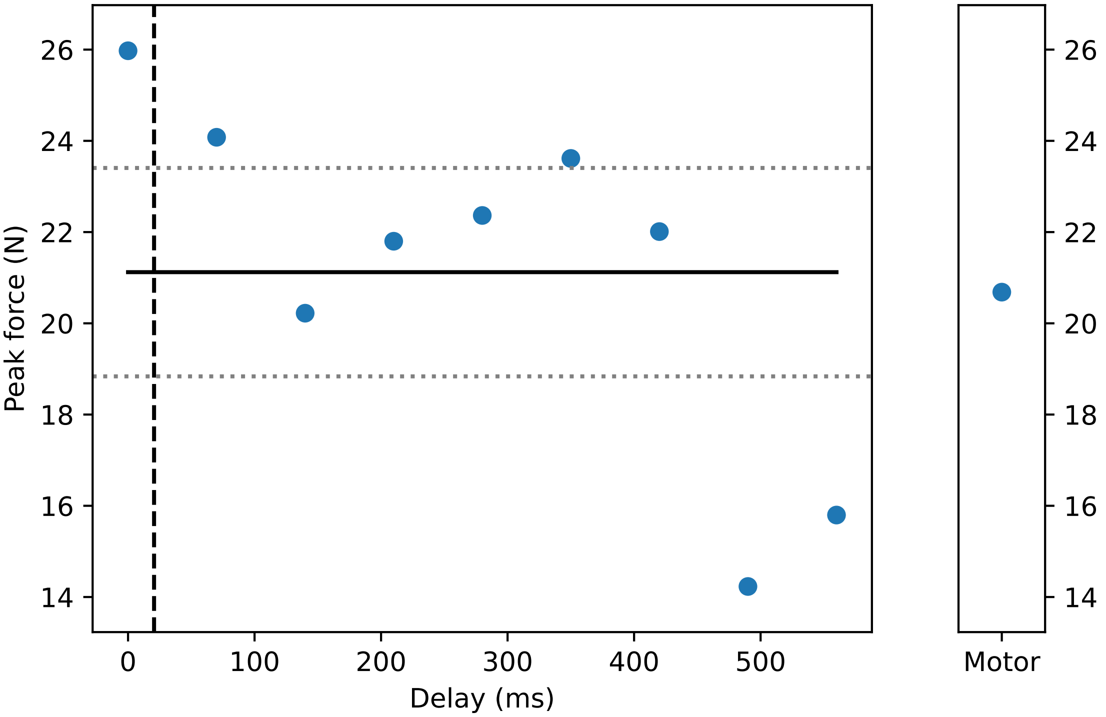

R^2: 0.0 !   Inflection point: 20.57 ms

Lower asymptote: 18.84 N; Upper asymptote: 23.41 N

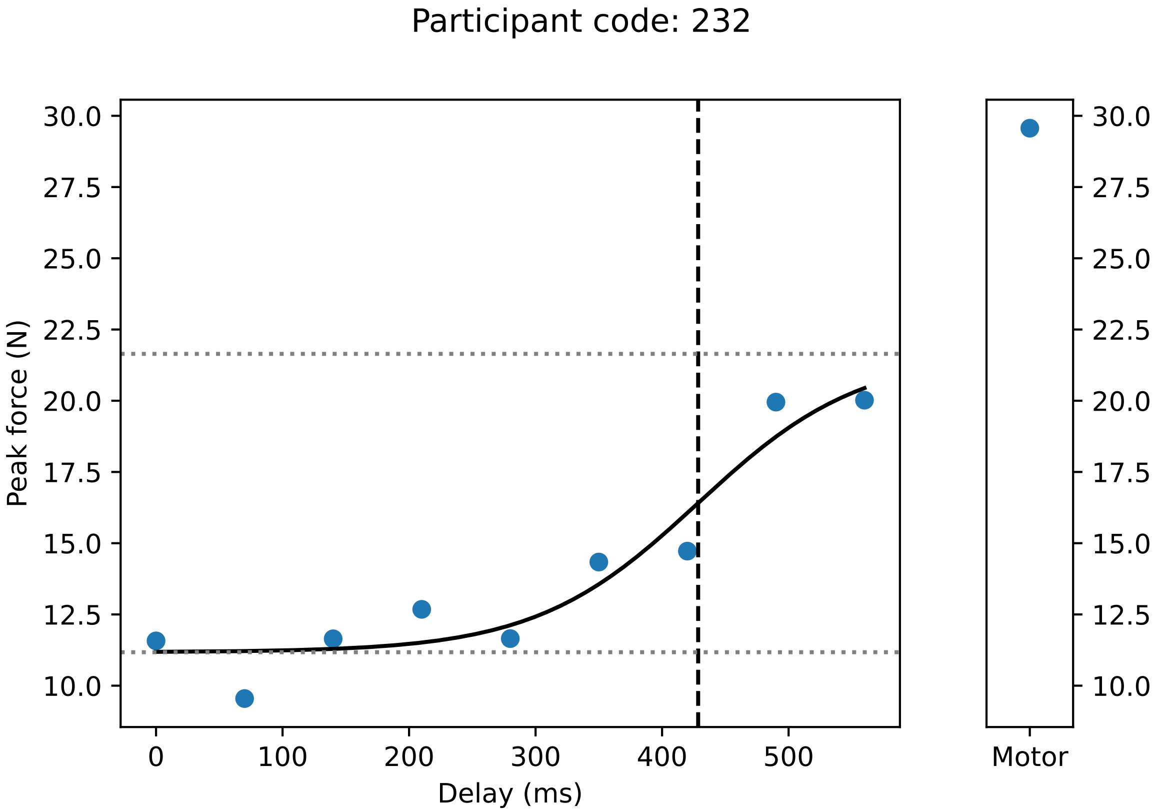

R^2: 0.92 Inflection point: 428.52 ms

Lower asymptote: 11.17 N; Upper asymptote: 21.65 N

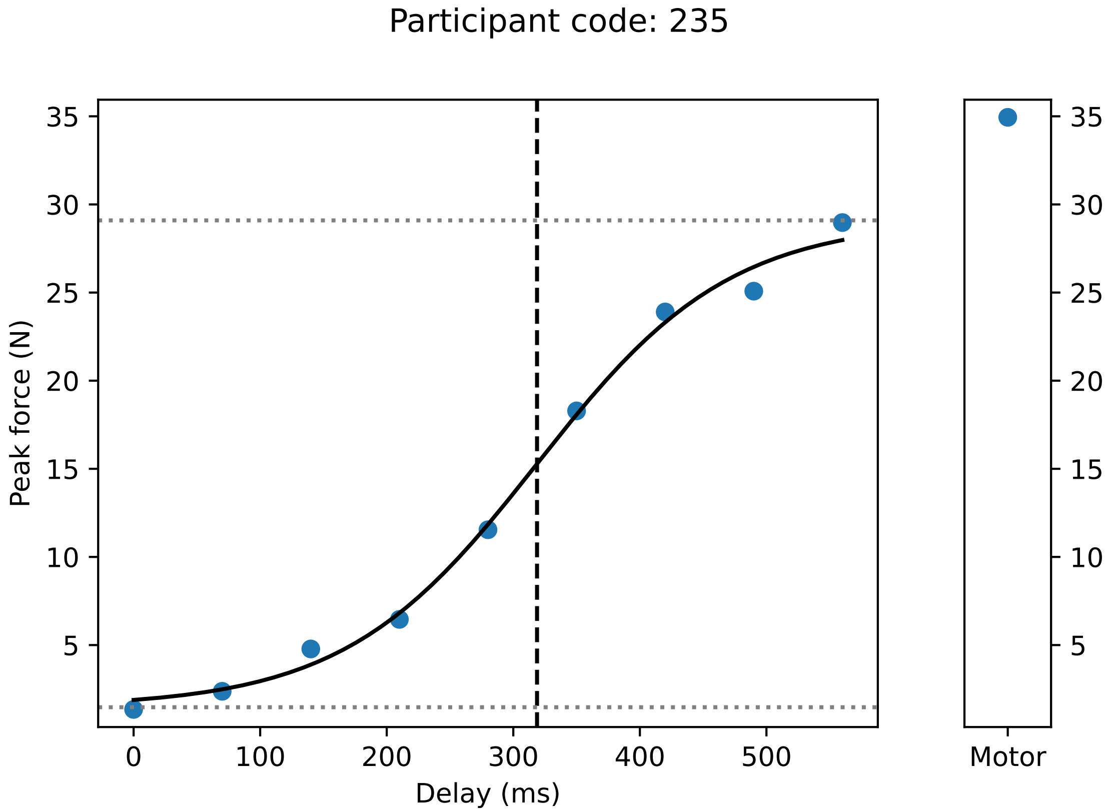

R^2: 0.99 Inflection point: 318.75 ms

Lower asymptote: 1.47 N; Upper asymptote: 29.09 N

Participant code: 236 !

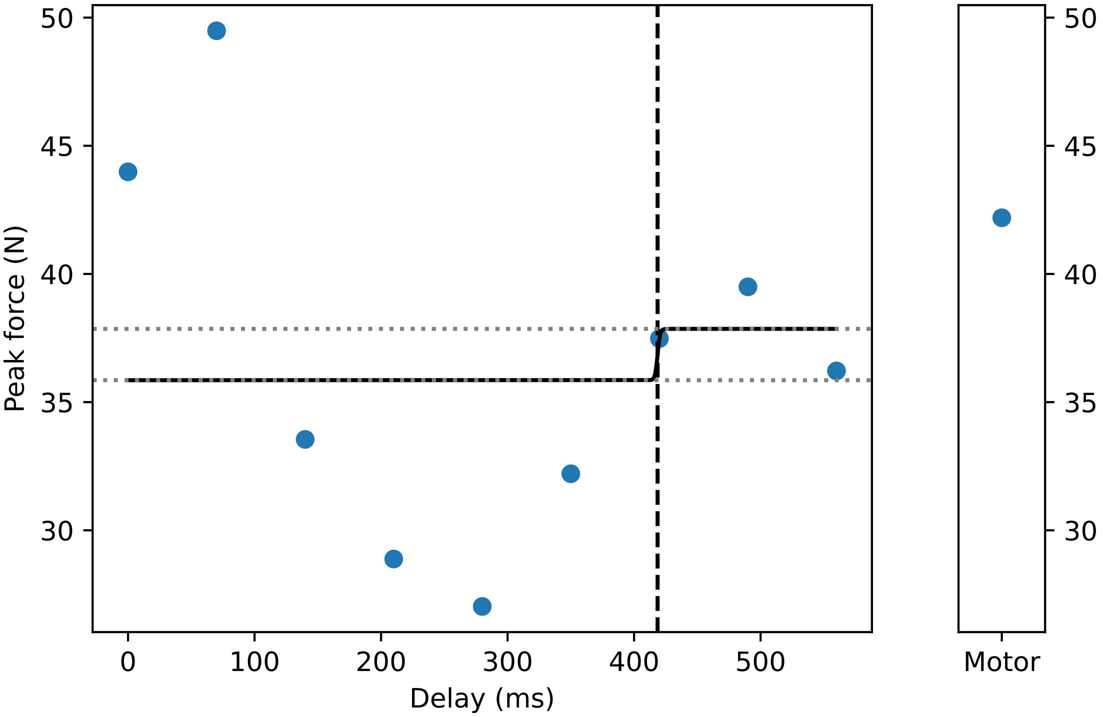

R^2: 0.02 ! Inflection point: 418.58 ms

Lower asymptote: 35.86 N; Upper asymptote: 37.86 N

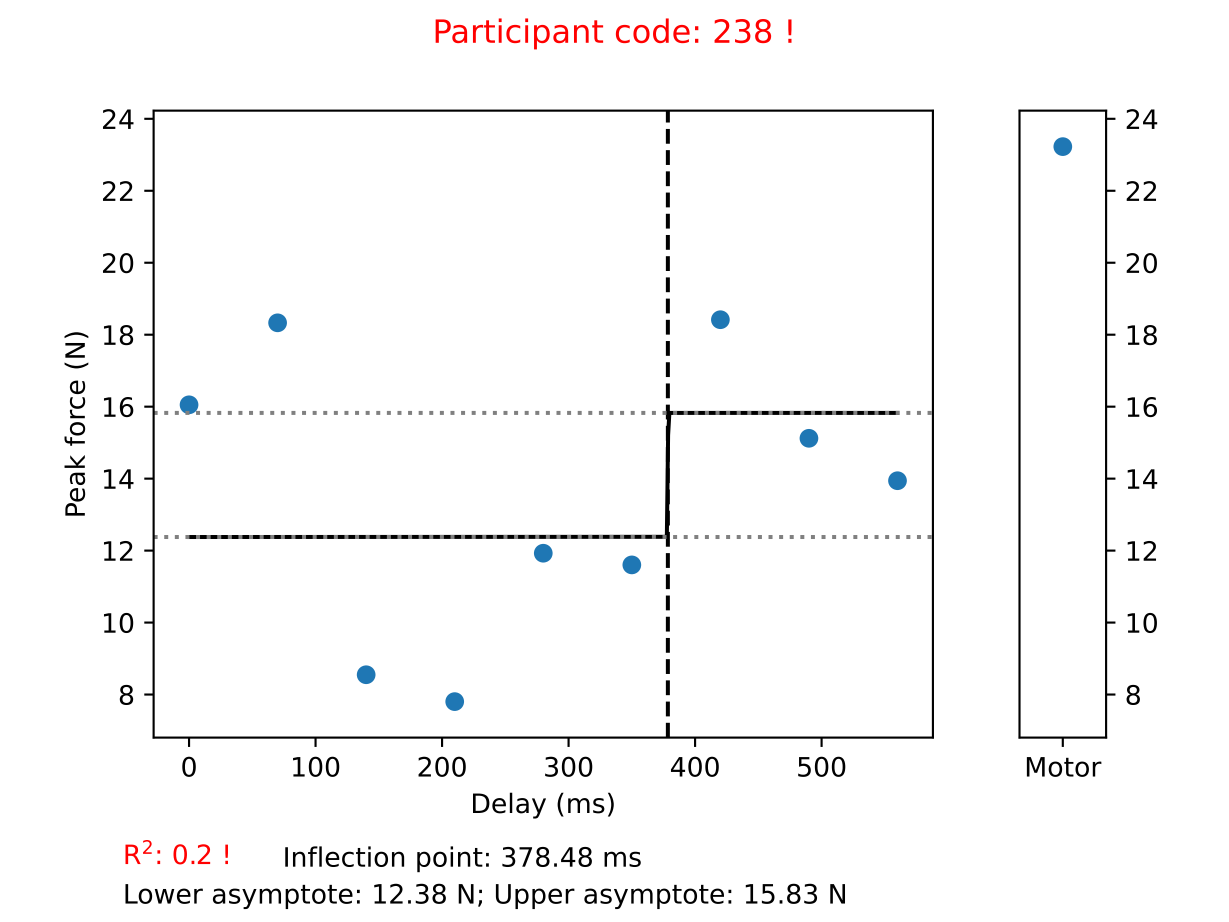

Participant code: 238 !
Peak force (N)
24
22
20
18
16
14
12
10
8
0
100
200
300
400
500
Delay (ms)
Motor
R²: 0.2 !
Inflection point: 378.48 ms
Lower asymptote: 12.38 N; Upper asymptote: 15.83 N

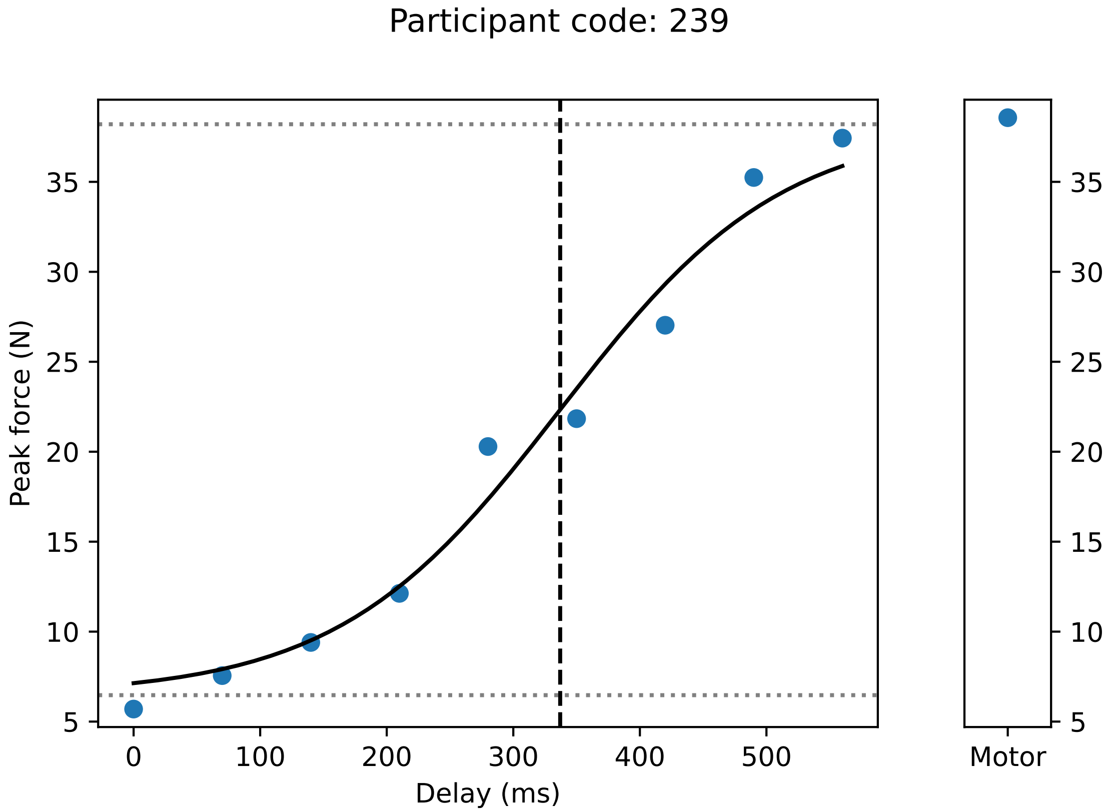

R^2: 0.98 Inflection point: 337.03 ms
Lower asymptote: 6.47 N; Upper asymptote: 38.2 N

Participant code: 247

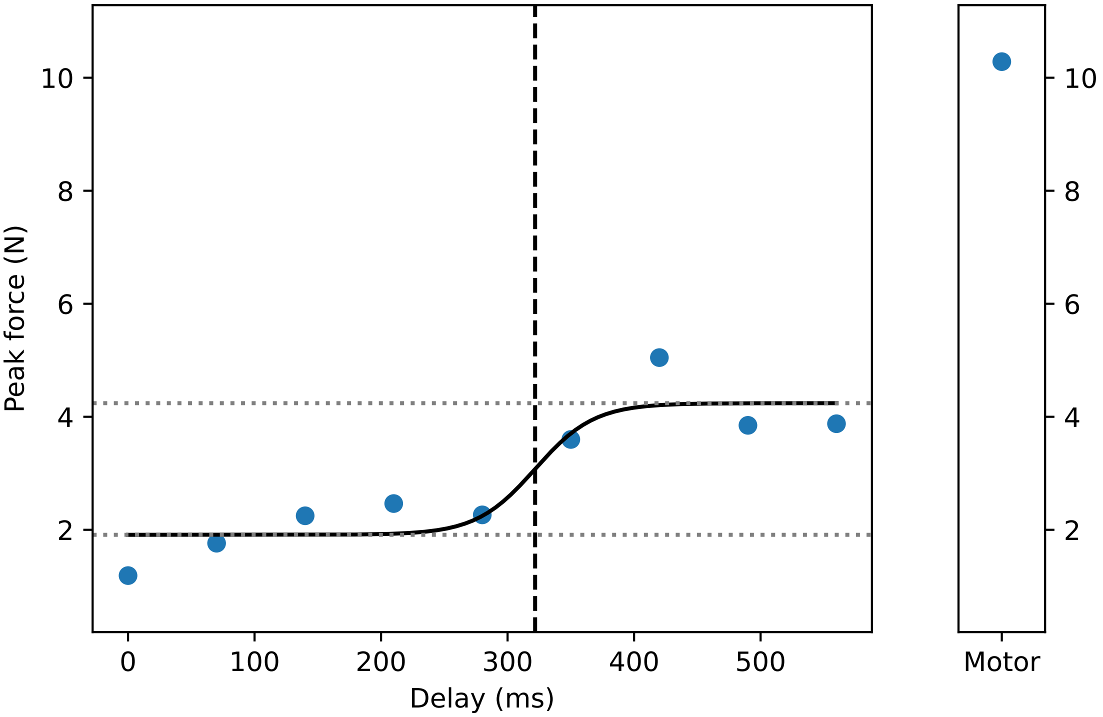

R^2: 0.84 Inflection point: 321.78 ms

Lower asymptote: 1.91 N; Upper asymptote: 4.24 N

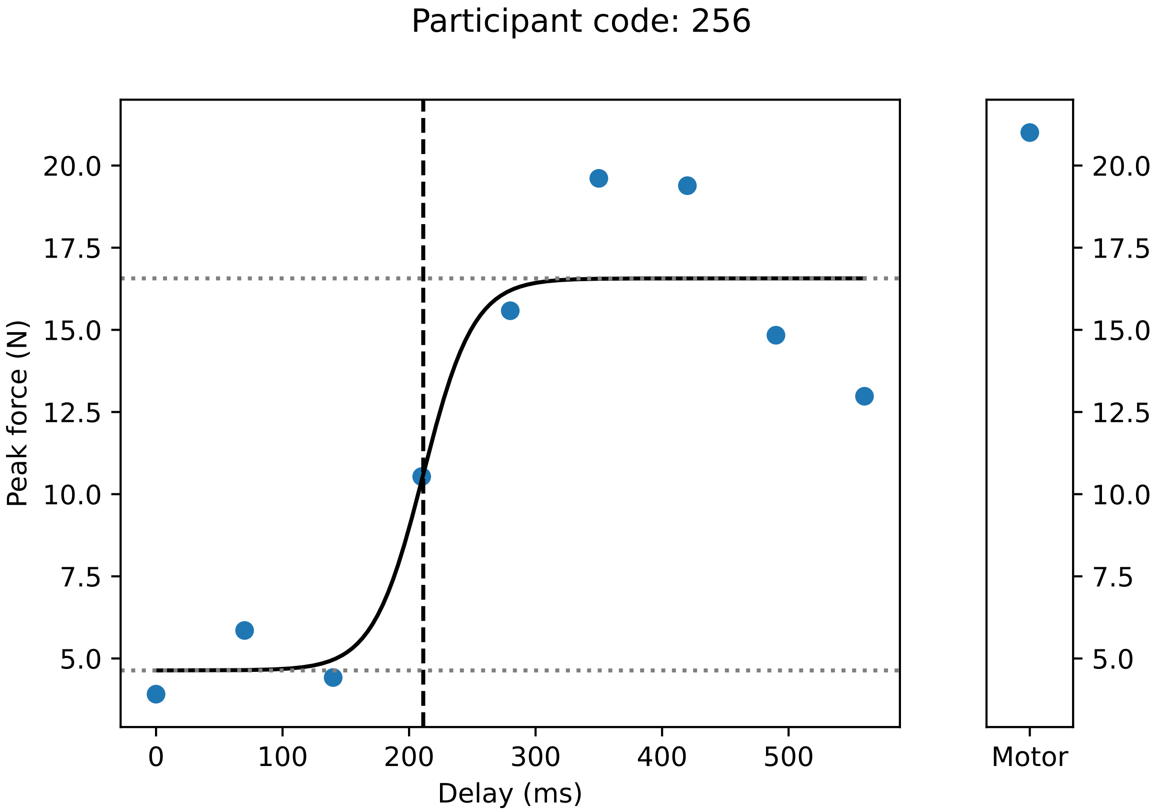

R^2: 0.88 Inflection point: 211.21 ms

Lower asymptote: 4.64 N; Upper asymptote: 16.57 N

Participant code: 259

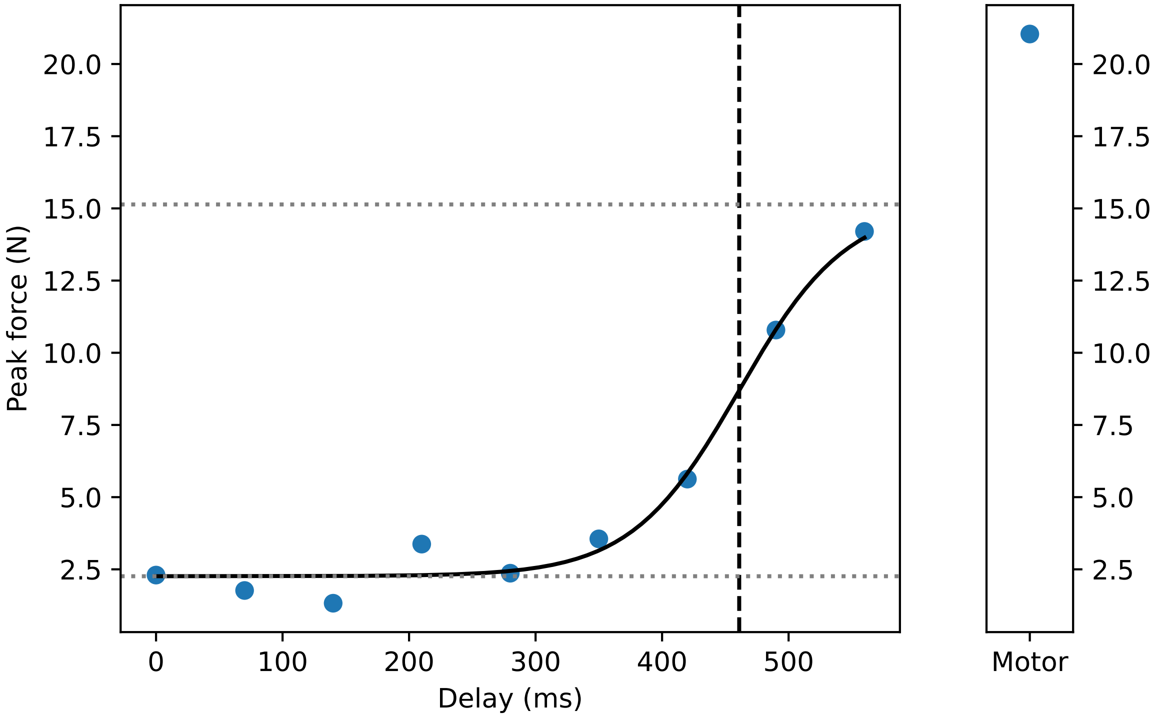

R^2: 0.98 Inflection point: 460.96 ms
Lower asymptote: 2.26 N; Upper asymptote: 15.14 N

Participant code: 260

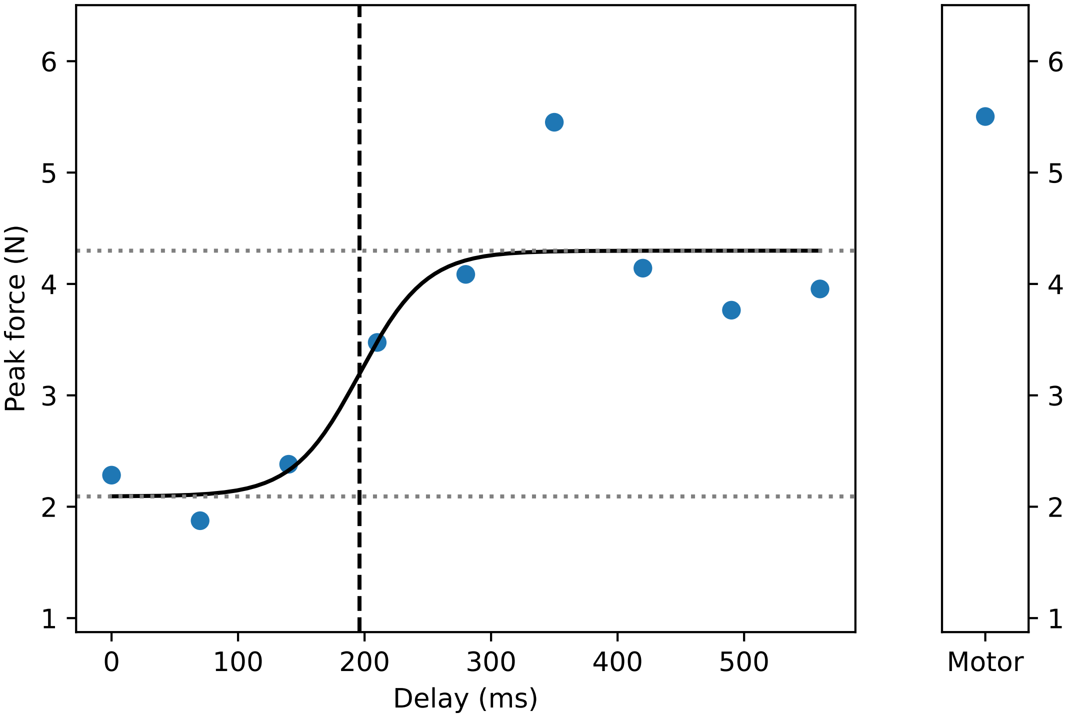

R^2: 0.82 Inflection point: 195.99 ms
Lower asymptote: 2.09 N; Upper asymptote: 4.3 N

Participant code: 261

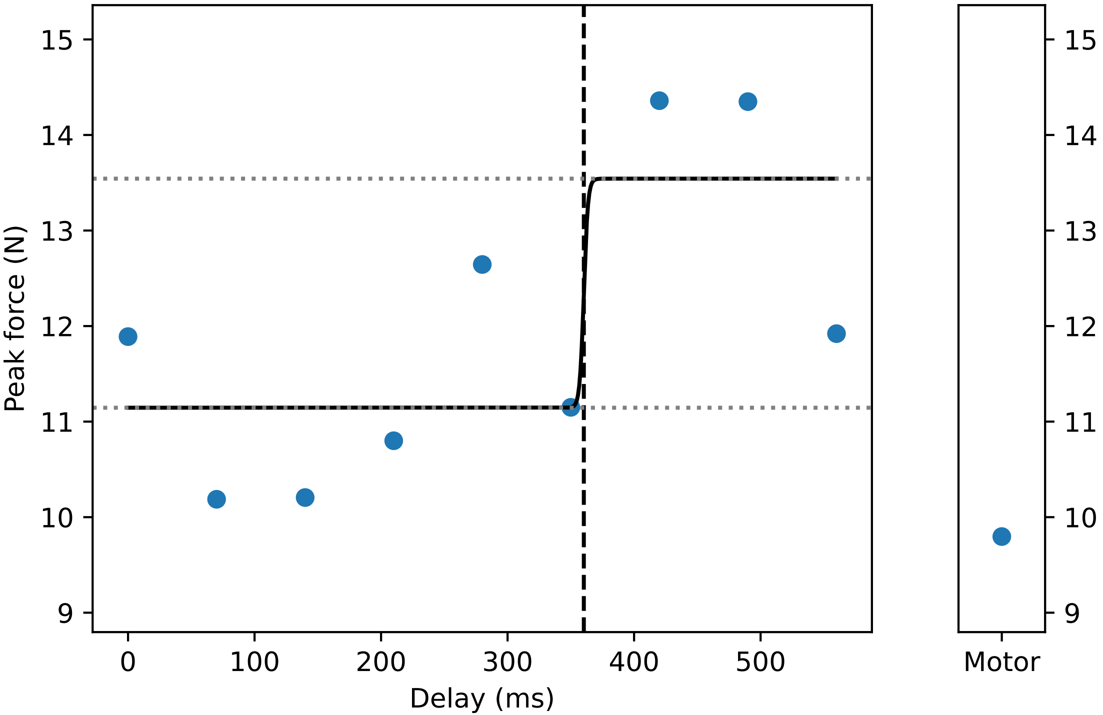

R^2: 0.57 Inflection point: 360.28 ms

Lower asymptote: 11.15 N; Upper asymptote: 13.54 N

Participant code: 264

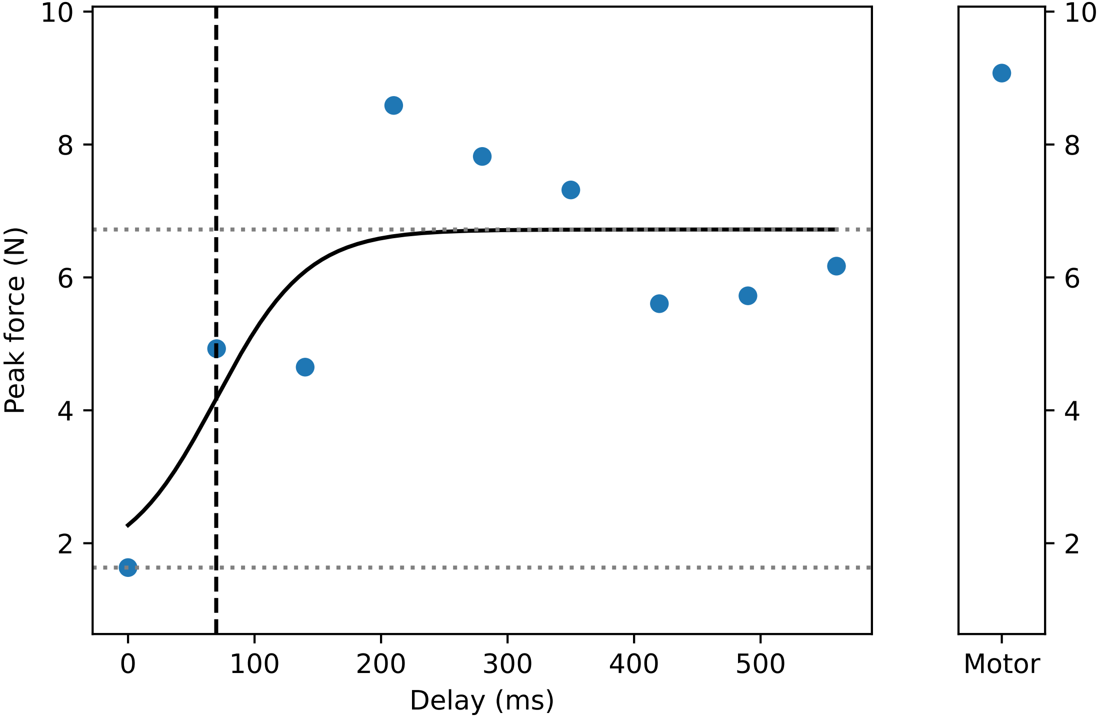

R^2: 0.67 Inflection point: 69.7 ms

Lower asymptote: 1.63 N; Upper asymptote: 6.72 N

Participant code: 265

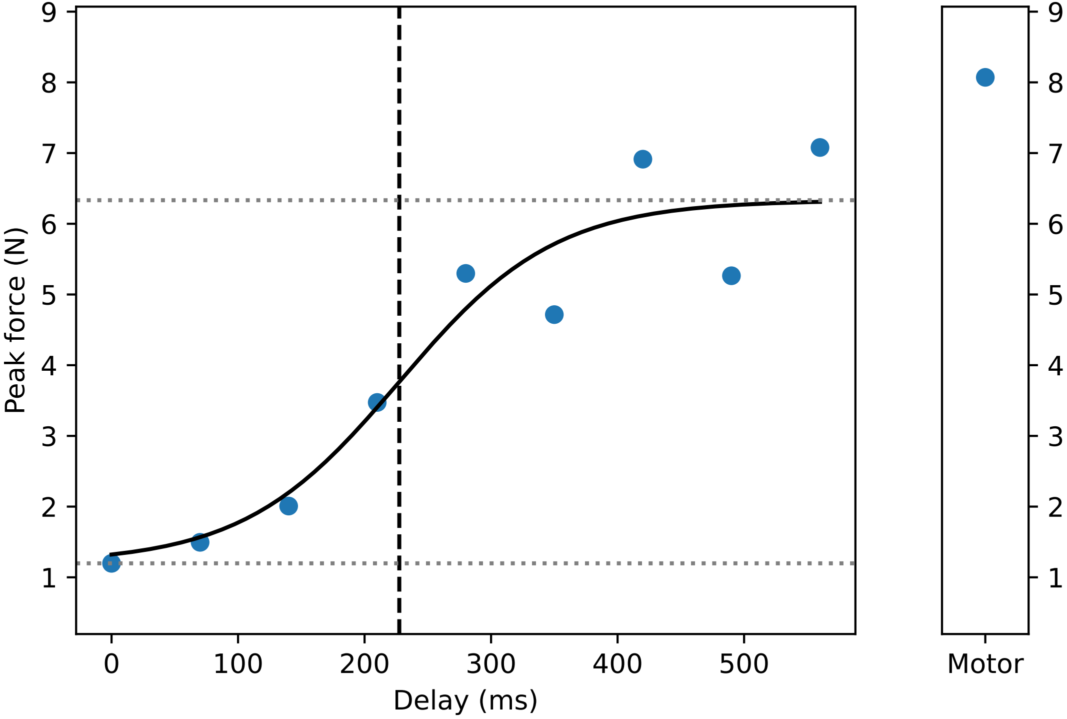

R^2: 0.91 Inflection point: 227.46 ms

Lower asymptote: 1.2 N; Upper asymptote: 6.33 N

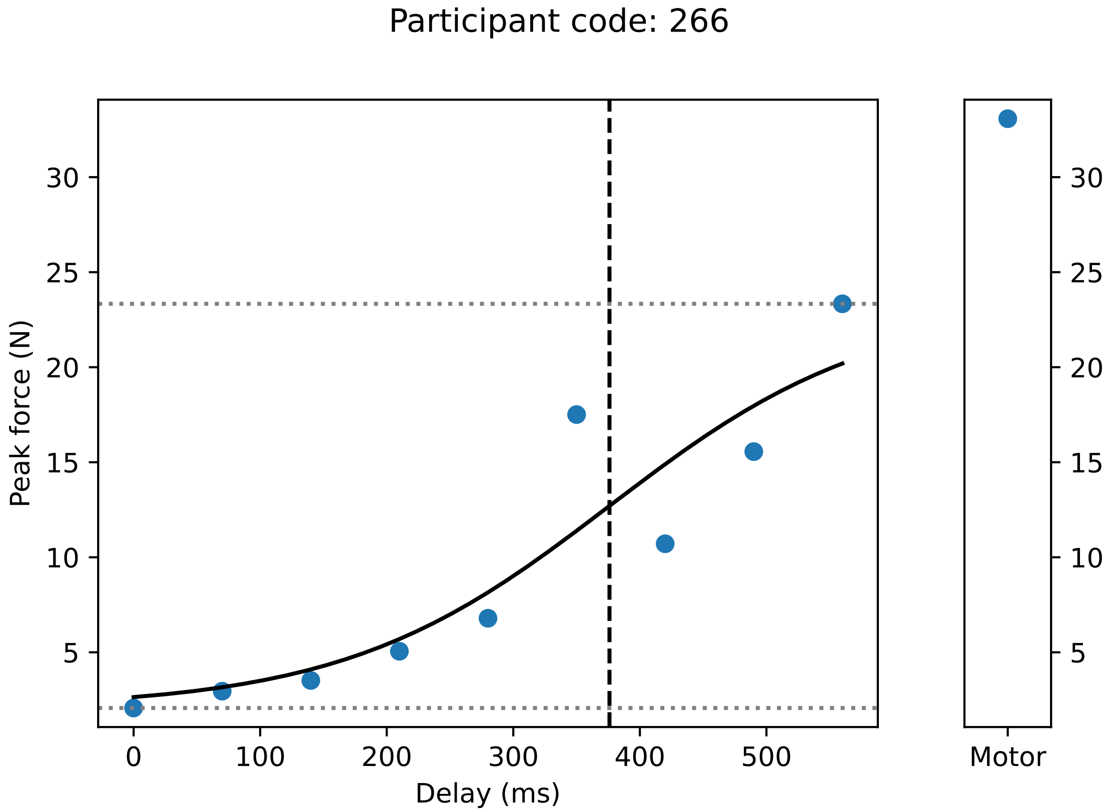

R^2: 0.84 Inflection point: 375.99 ms

Lower asymptote: 2.07 N; Upper asymptote: 23.34 N

Participant code: 269

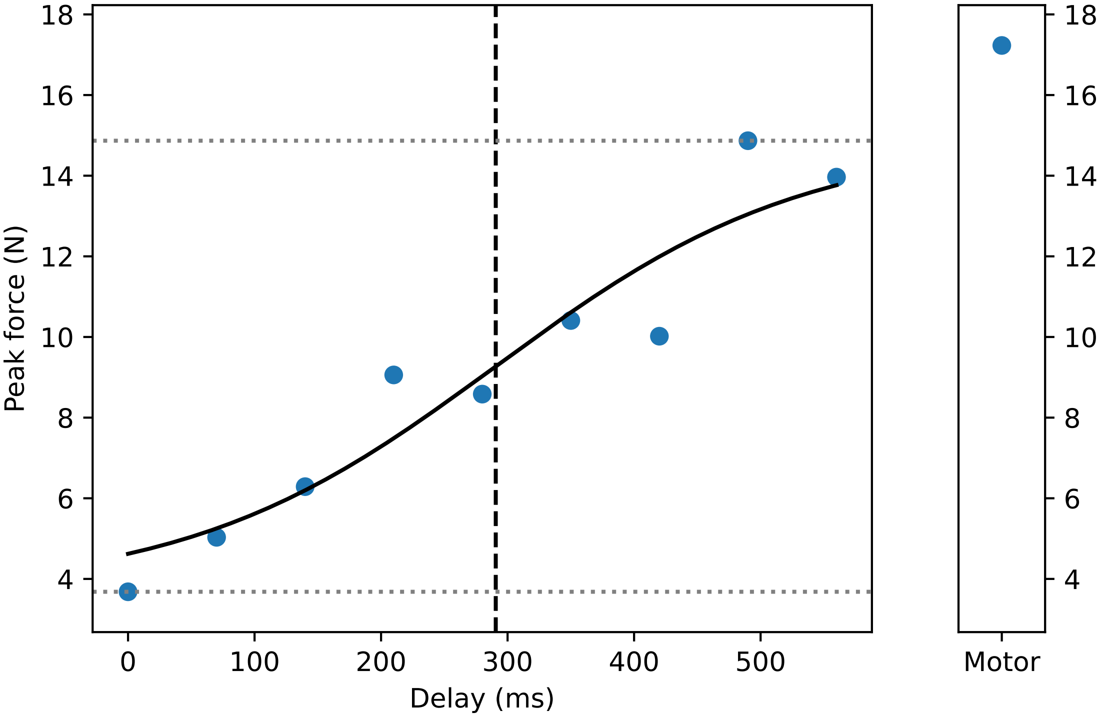

R^2: 0.9 Inflection point: 290.66 ms

Lower asymptote: 3.68 N; Upper asymptote: 14.87 N

Participant code: 271

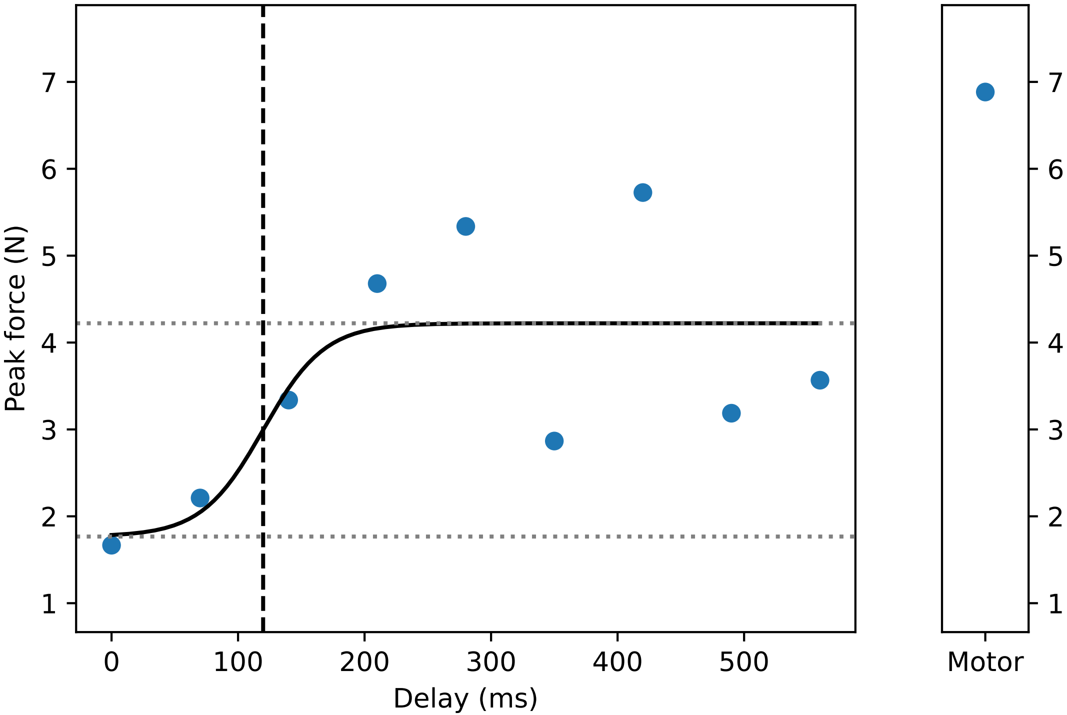

R^2: 0.53 Inflection point: 119.83 ms

Lower asymptote: 1.77 N; Upper asymptote: 4.22 N

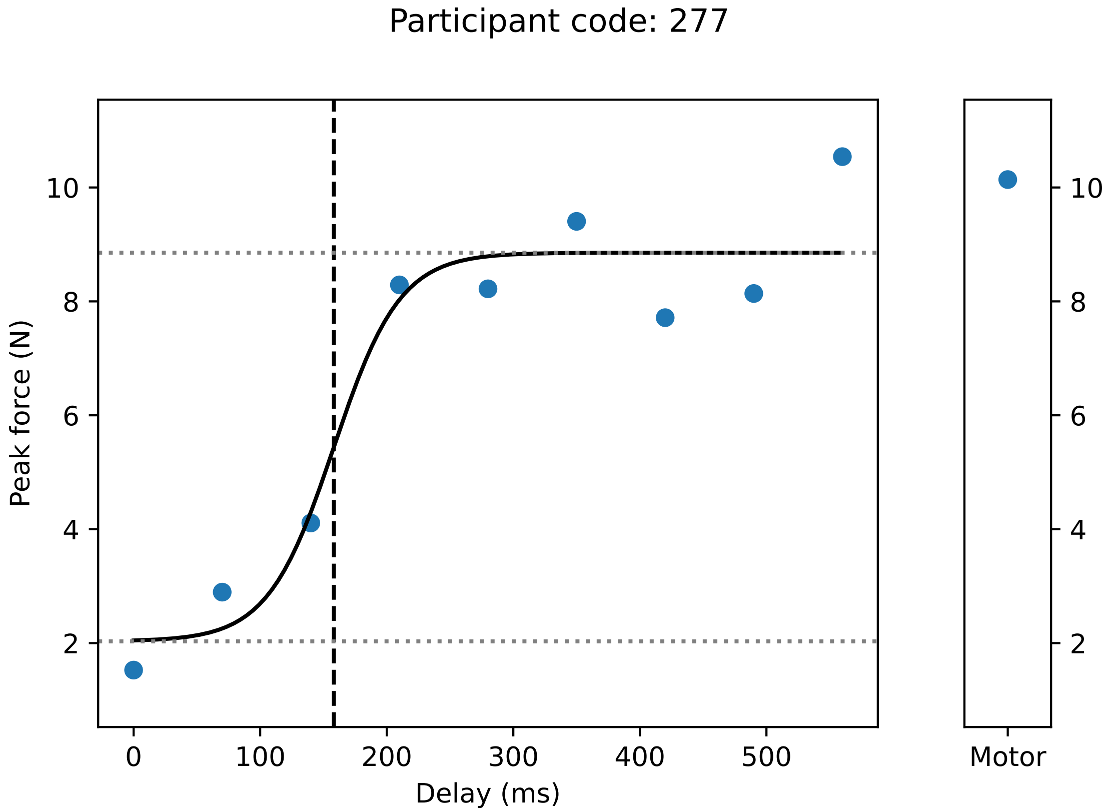

R^2: 0.92   Inflection point: 158.24 ms

Lower asymptote: 2.03 N; Upper asymptote: 8.86 N

Participant code: 278

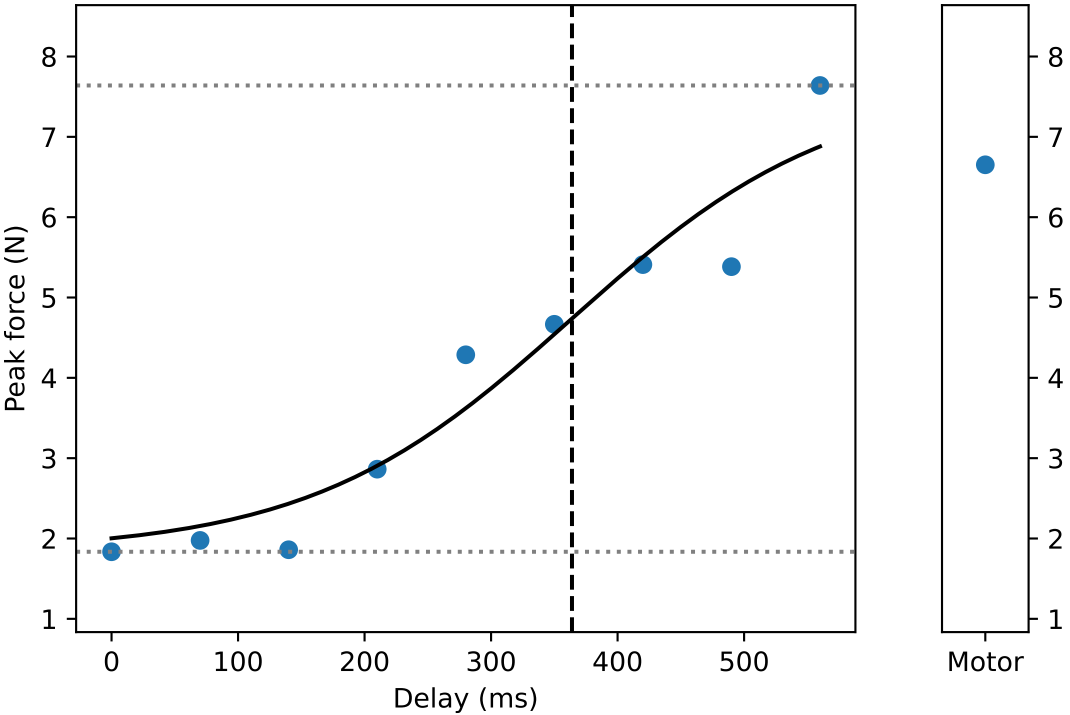

R^2: 0.93 Inflection point: 363.95 ms

Lower asymptote: 1.83 N; Upper asymptote: 7.64 N

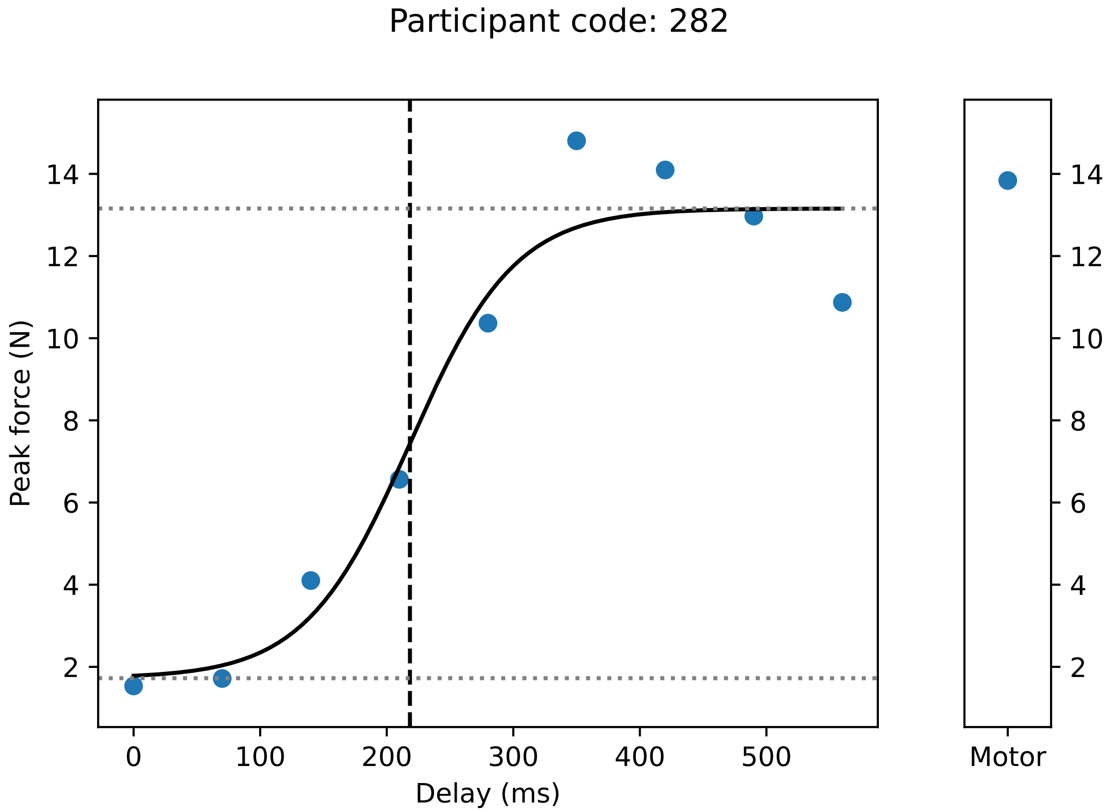

R^2: 0.94 Inflection point: 218.33 ms
Lower asymptote: 1.72 N; Upper asymptote: 13.16 N

Participant code: 283

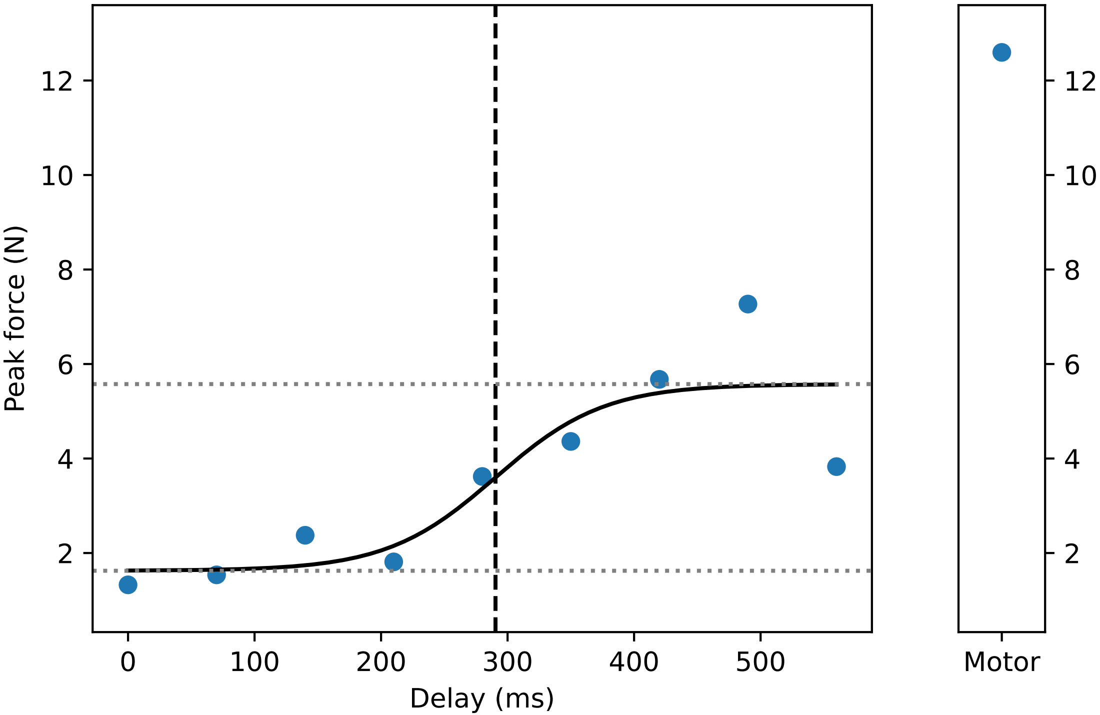

R^2: 0.79 Inflection point: 290.45 ms

Lower asymptote: 1.62 N; Upper asymptote: 5.57 N

Participant code: 287

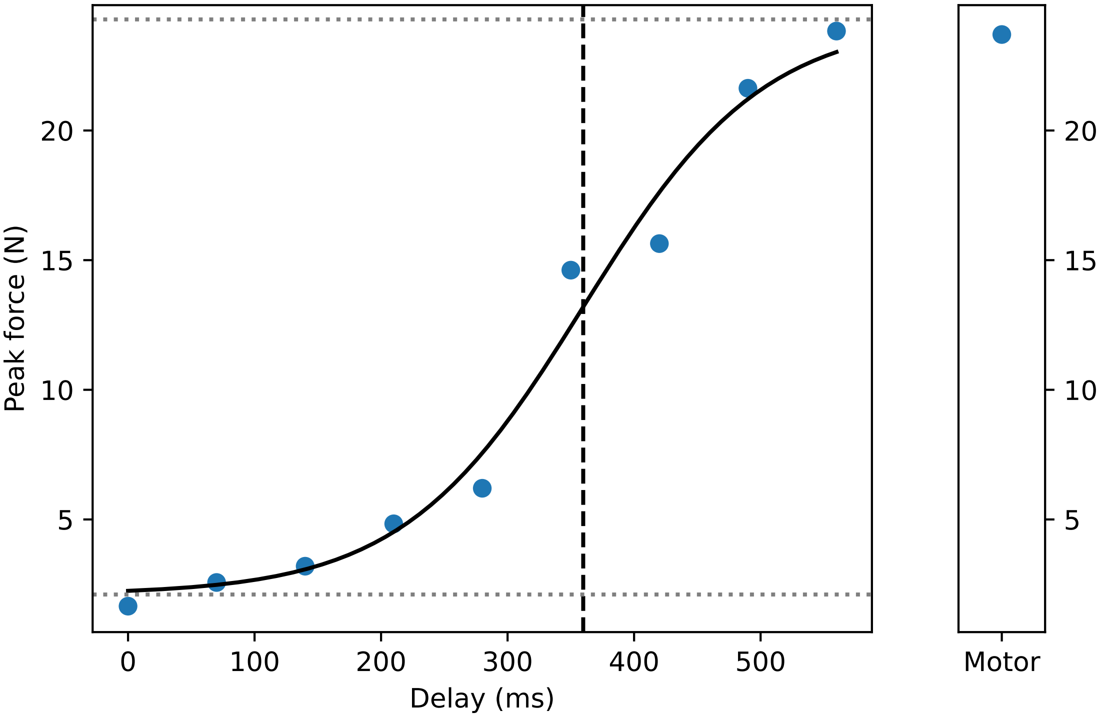

R^2: 0.98 Inflection point: 359.79 ms
Lower asymptote: 2.11 N; Upper asymptote: 24.28 N

Participant code: 289

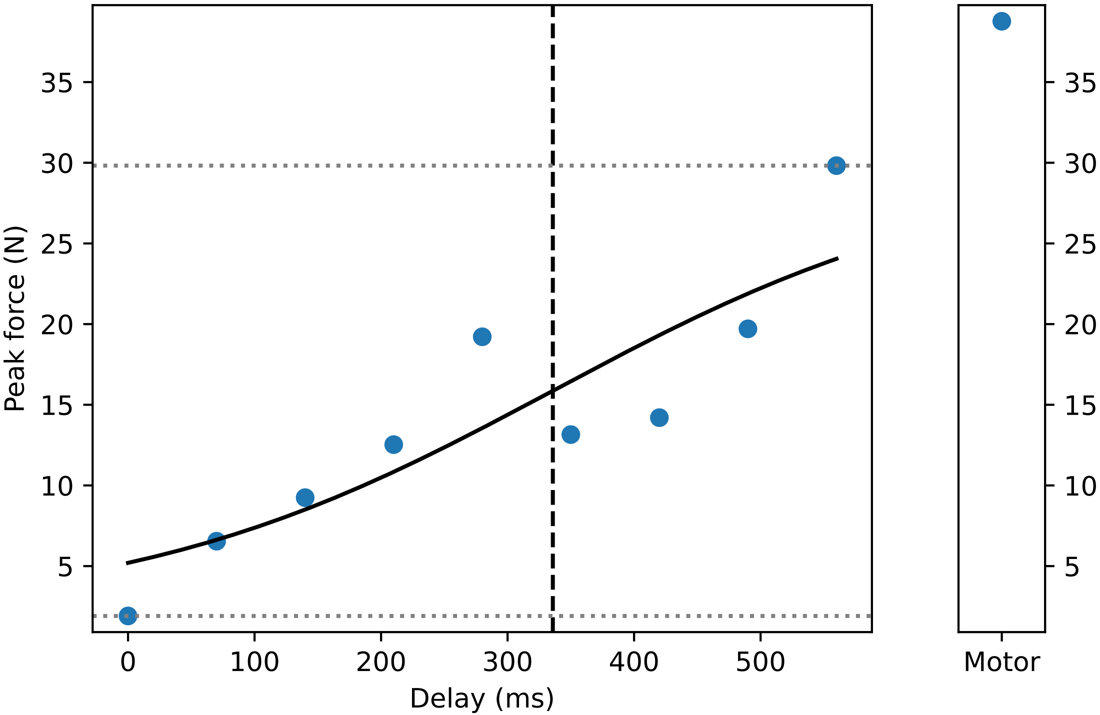

R^2: 0.77 Inflection point: 335.78 ms

Lower asymptote: 1.91 N; Upper asymptote: 29.82 N

Participant code: 292

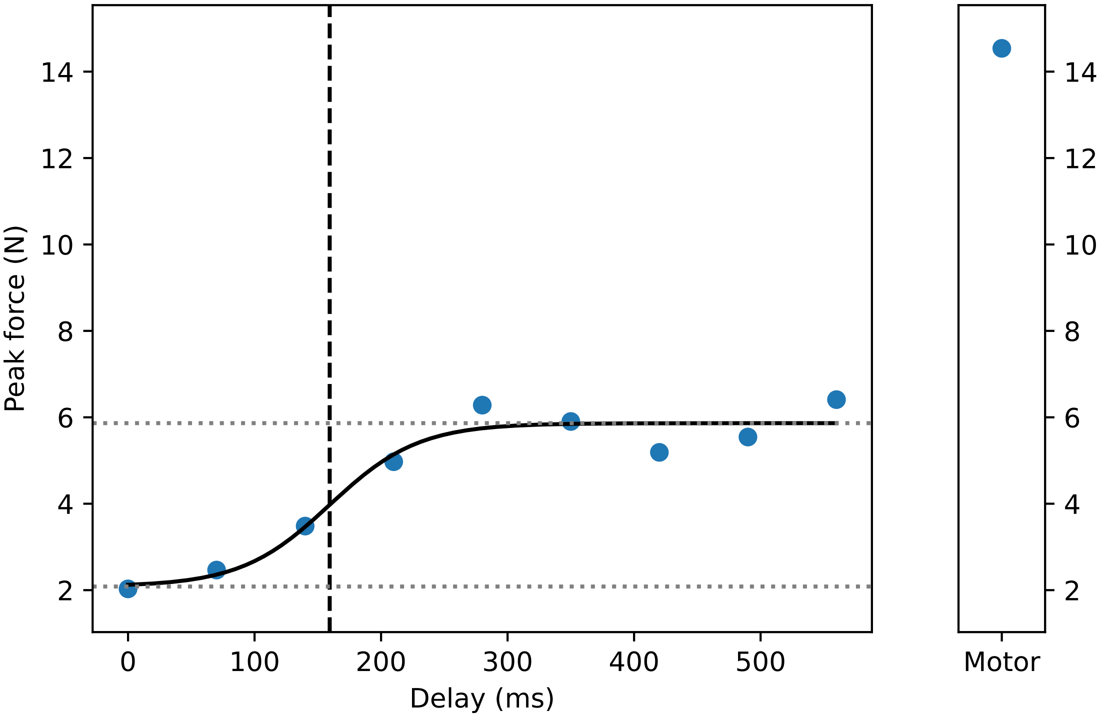

R^2: 0.94 Inflection point: 159.4 ms

Lower asymptote: 2.08 N; Upper asymptote: 5.86 N

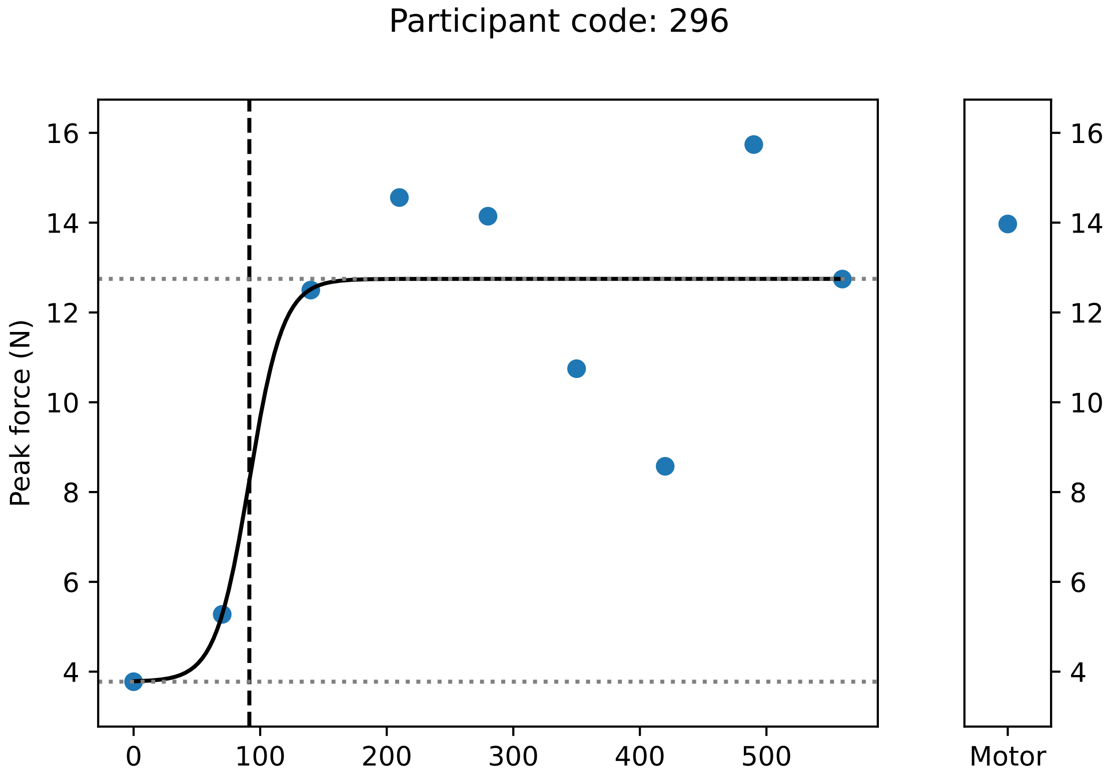

R^2: 0.75 Inflection point: 91.44 ms

Lower asymptote: 3.78 N; Upper asymptote: 12.75 N

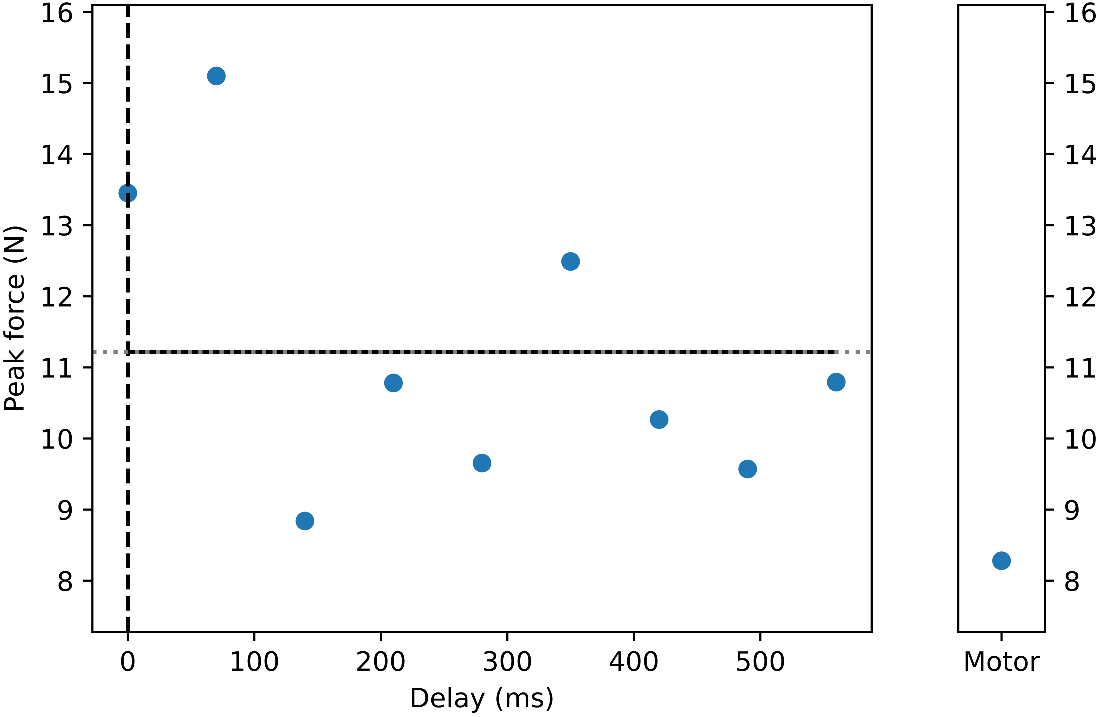

R^2: 0.0 !   Inflection point: 0.0 ms

Lower asymptote: 11.22 N; Upper asymptote: 11.22 N

Participant code: 300

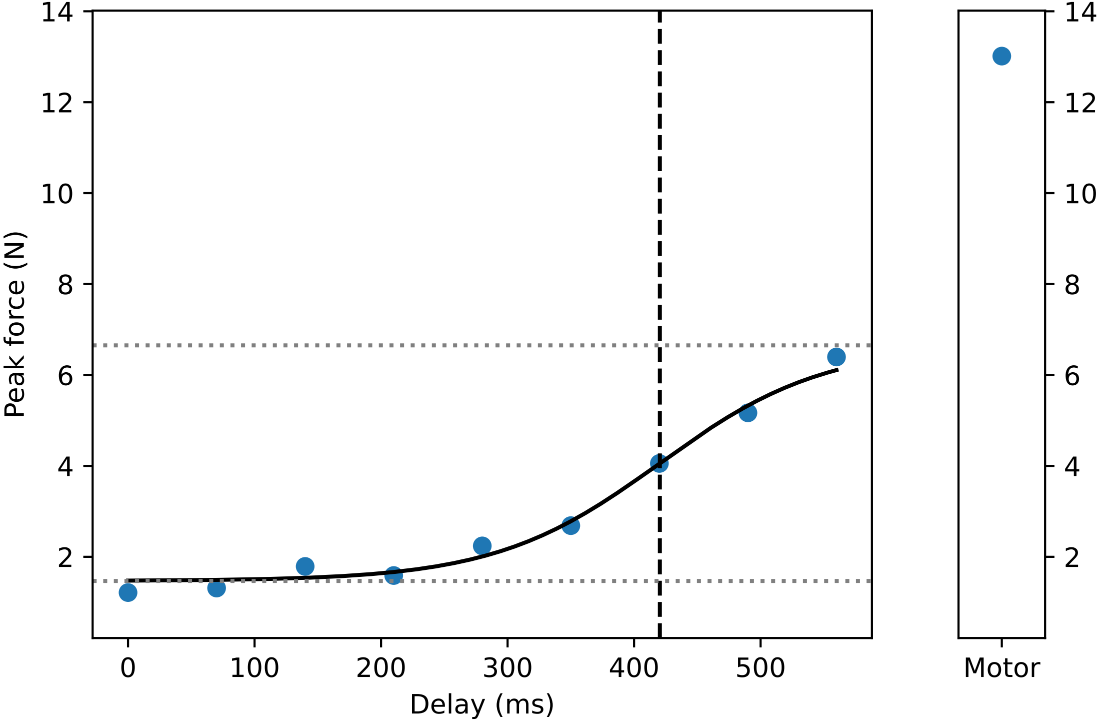

R^2: 0.99 Inflection point: 420.37 ms
Lower asymptote: 1.47 N; Upper asymptote: 6.65 N

Participant code: 305

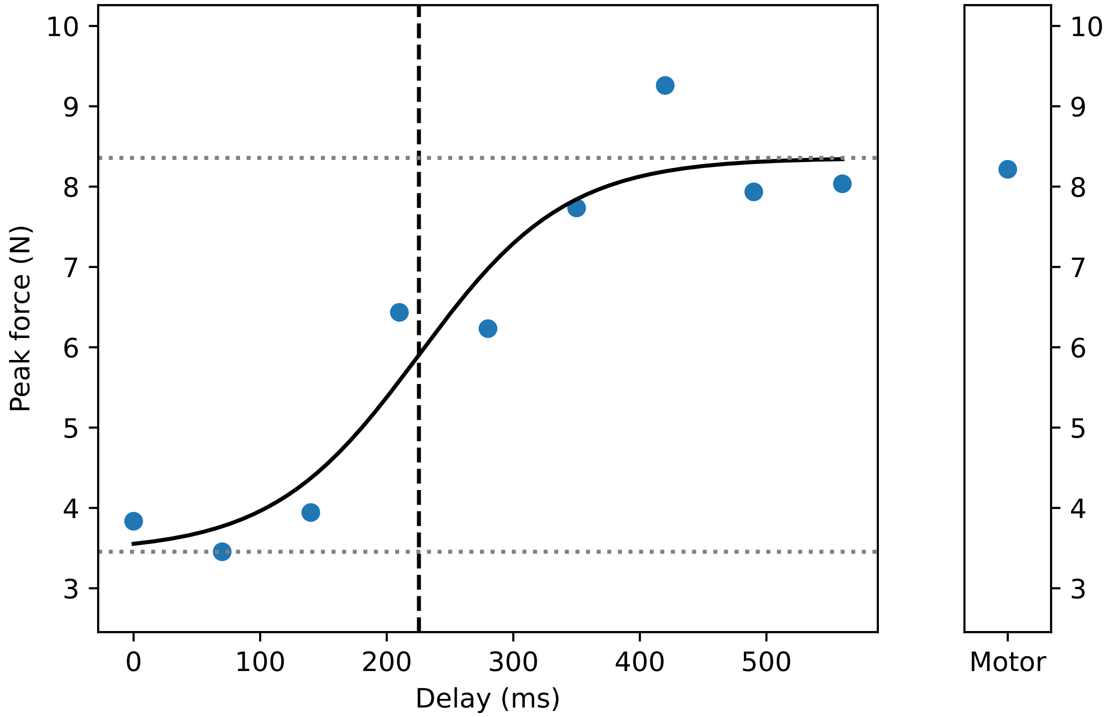

R^2: 0.92 Inflection point: 225.43 ms

Lower asymptote: 3.45 N; Upper asymptote: 8.36 N

Participant code: 307

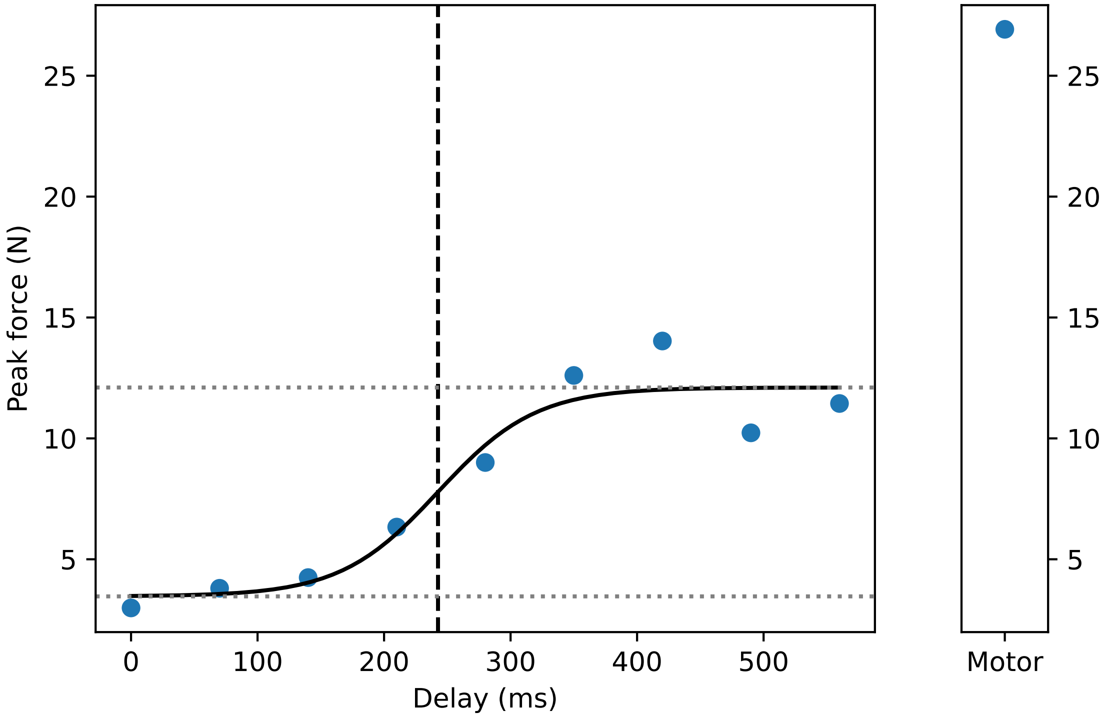

R^2: 0.93 Inflection point: 242.65 ms

Lower asymptote: 3.47 N; Upper asymptote: 12.1 N

Participant code: 319

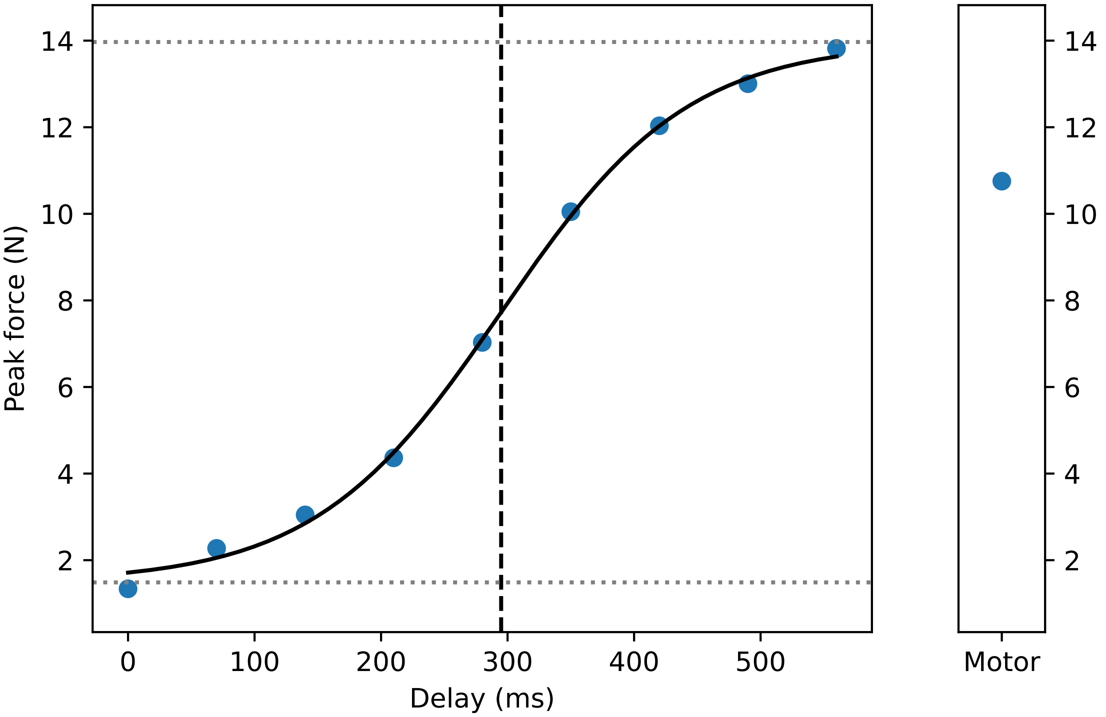

R^2: 1.0 Inflection point: 294.92 ms

Lower asymptote: 1.49 N; Upper asymptote: 13.97 N

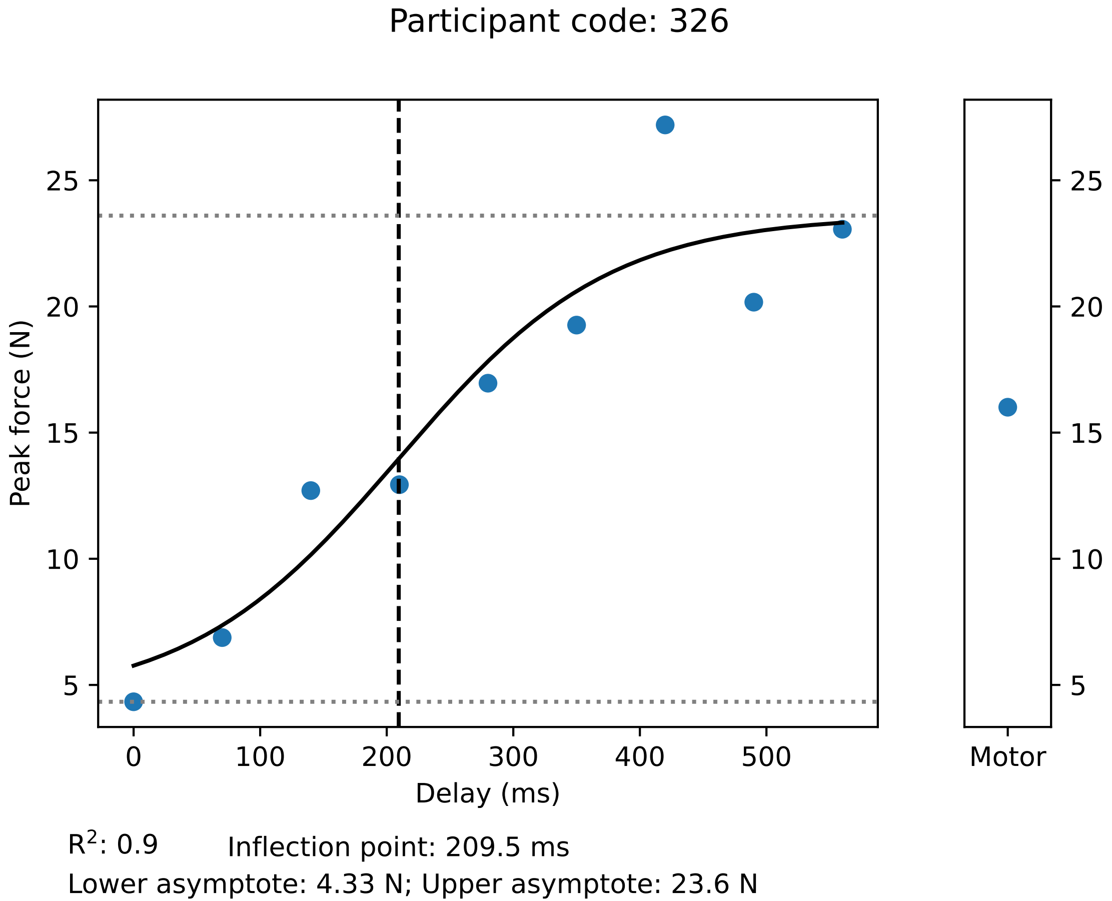
Participant code: 326
Peak force (N)
25
20
15
10
5
0
100
200
300
400
500
Delay (ms)
Motor
R²: 0.9
Inflection point: 209.5 ms
Lower asymptote: 4.33 N; Upper asymptote: 23.6 N

Participant code: 333

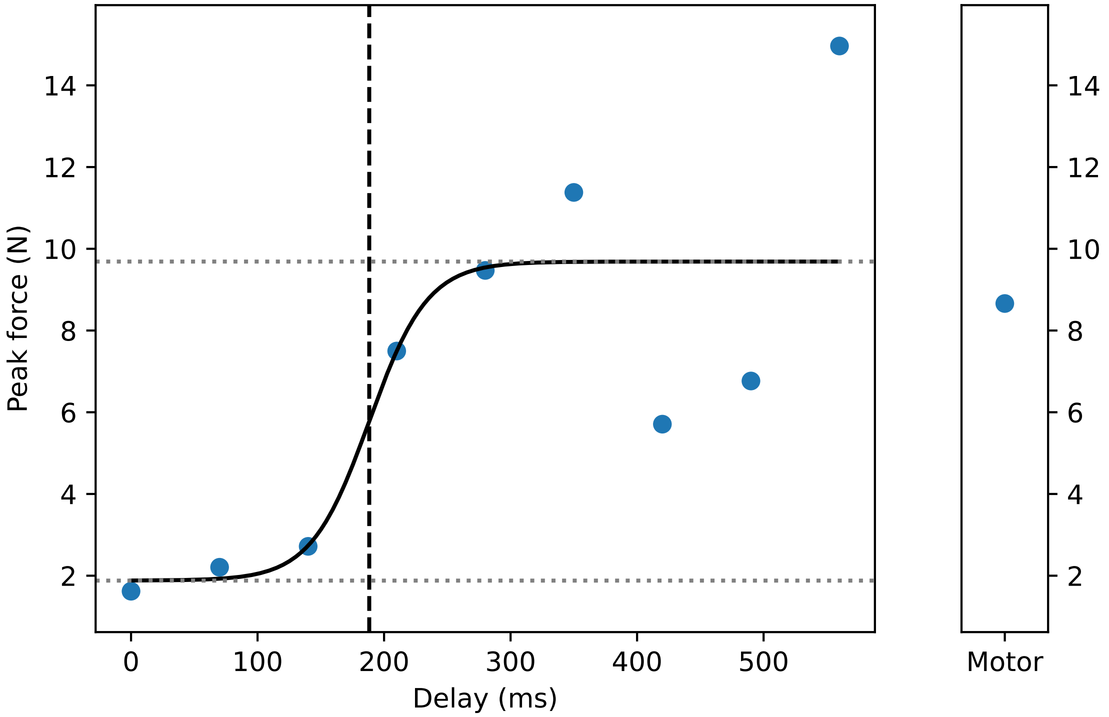

R^2: 0.66 Inflection point: 188.28 ms

Lower asymptote: 1.88 N; Upper asymptote: 9.69 N

Participant code: 335 !

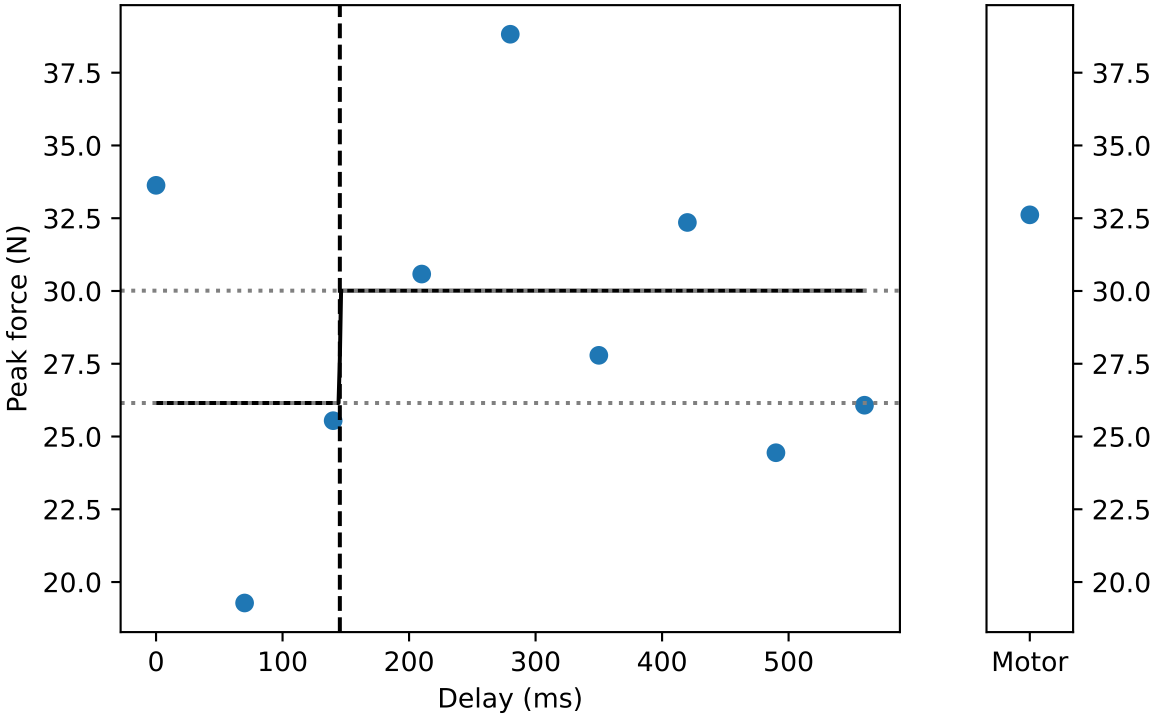

R^2: 0.11 !   Inflection point: 145.28 ms

Lower asymptote: 26.15 N; Upper asymptote: 30.01 N

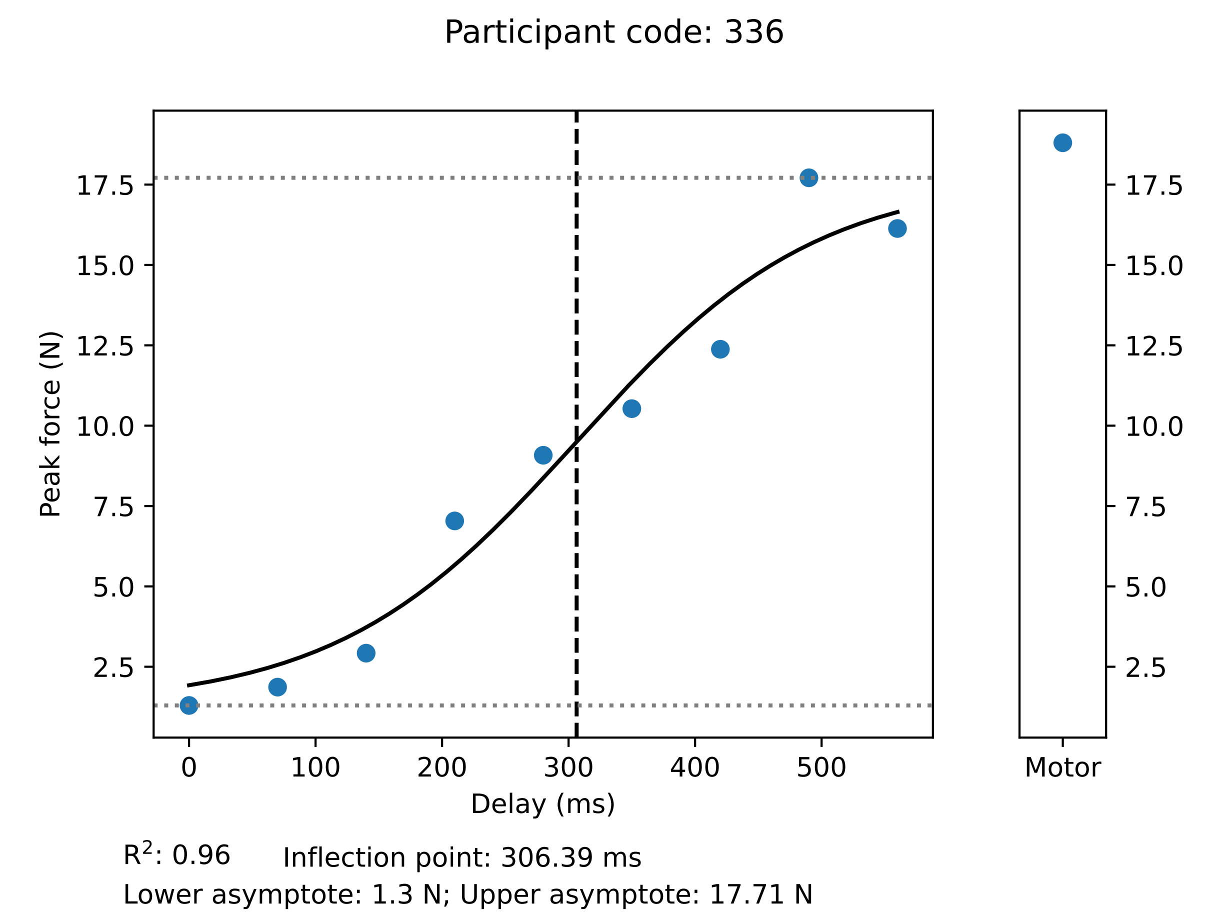
Participant code: 336
Peak force (N)
17.5
15.0
12.5
10.0
7.5
5.0
2.5
0
100
200
300
400
500
Delay (ms)
Motor
R2: 0.96
Inflection point: 306.39 ms
Lower asymptote: 1.3 N; Upper asymptote: 17.71 N

Participant code: 339

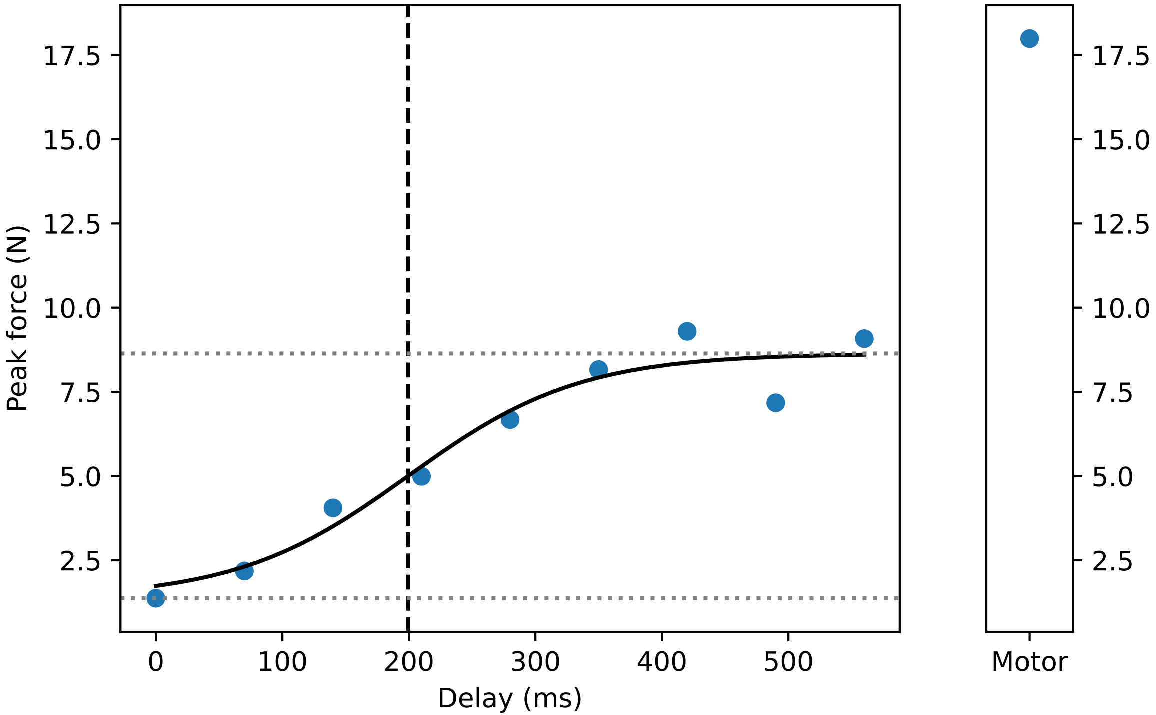

R^2: 0.95 Inflection point: 199.52 ms
Lower asymptote: 1.37 N; Upper asymptote: 8.64 N

Participant code: 343

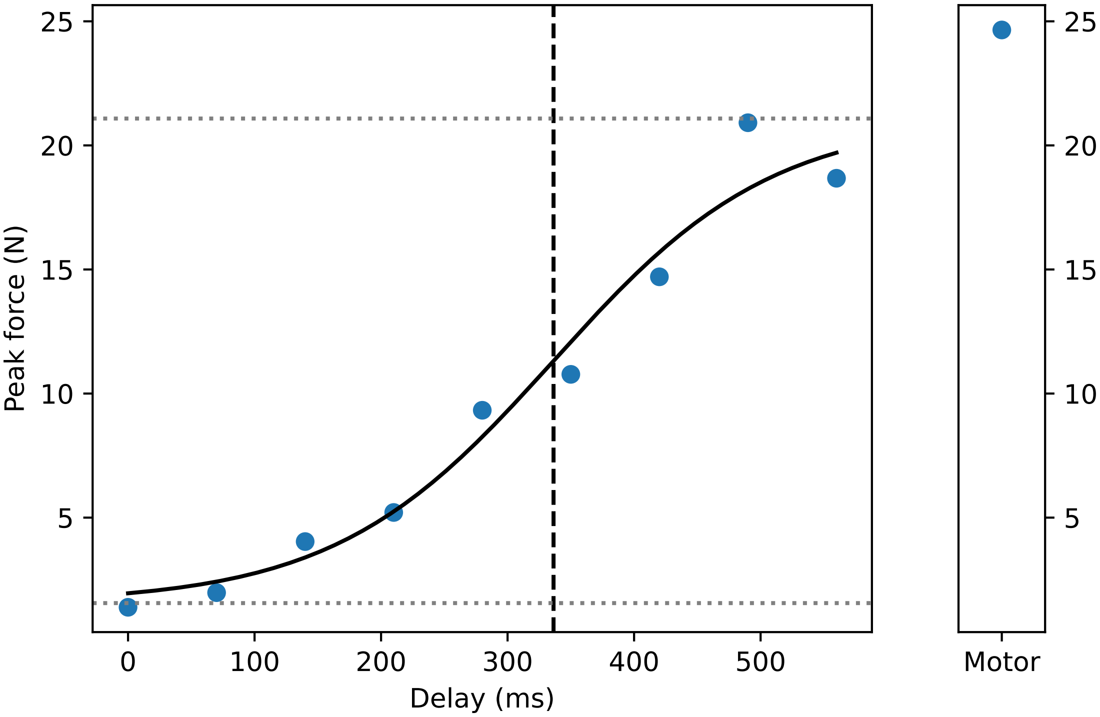

R^2: 0.97 Inflection point: 336.33 ms

Lower asymptote: 1.56 N; Upper asymptote: 21.08 N

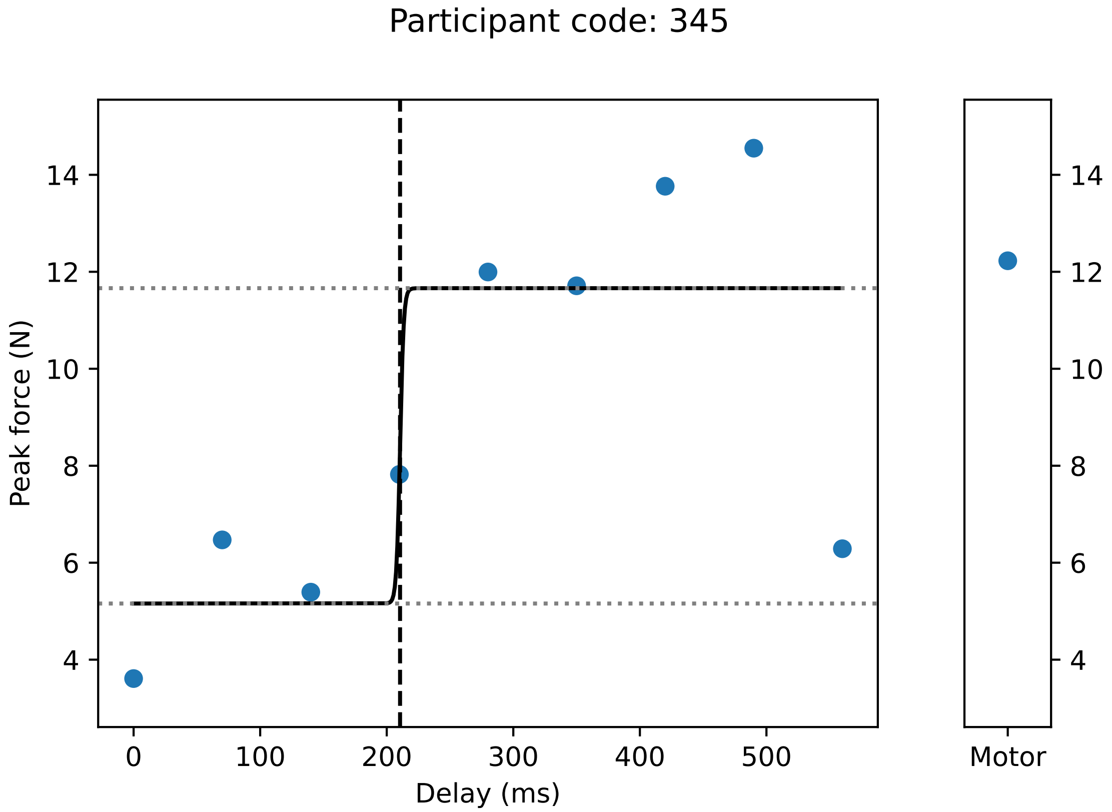

R^2: 0.64 Inflection point: 210.53 ms
Lower asymptote: 5.16 N; Upper asymptote: 11.66 N

Participant code: 348

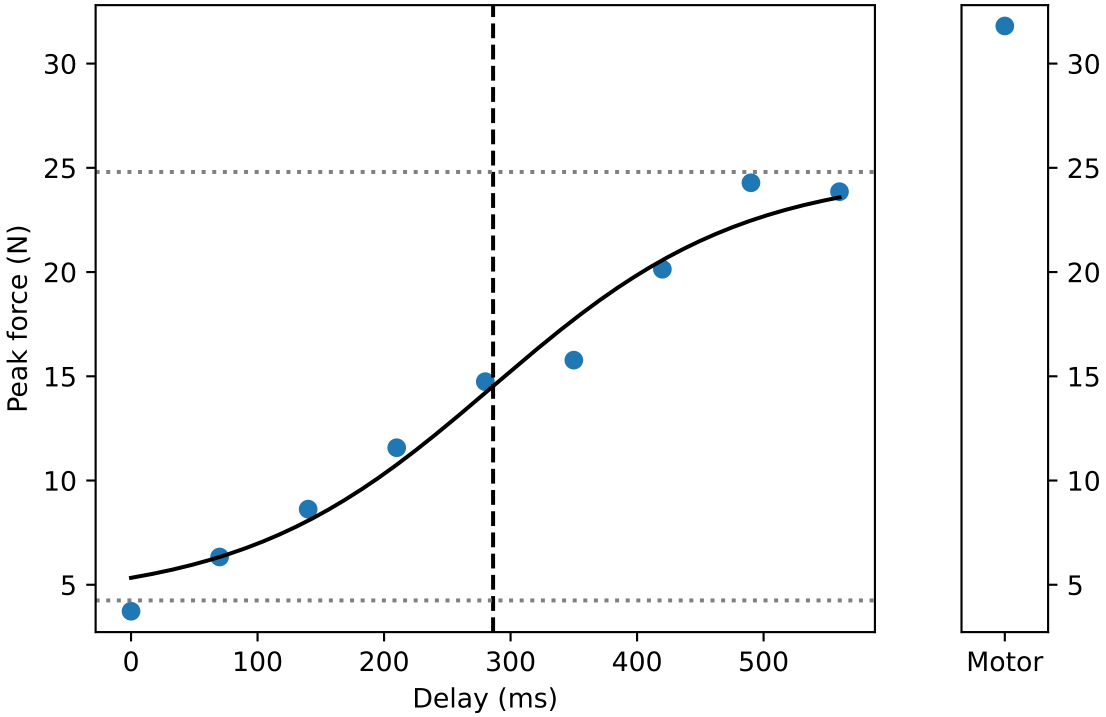

R^2: 0.97 Inflection point: 286.18 ms

Lower asymptote: 4.25 N; Upper asymptote: 24.8 N

Participant code: 349

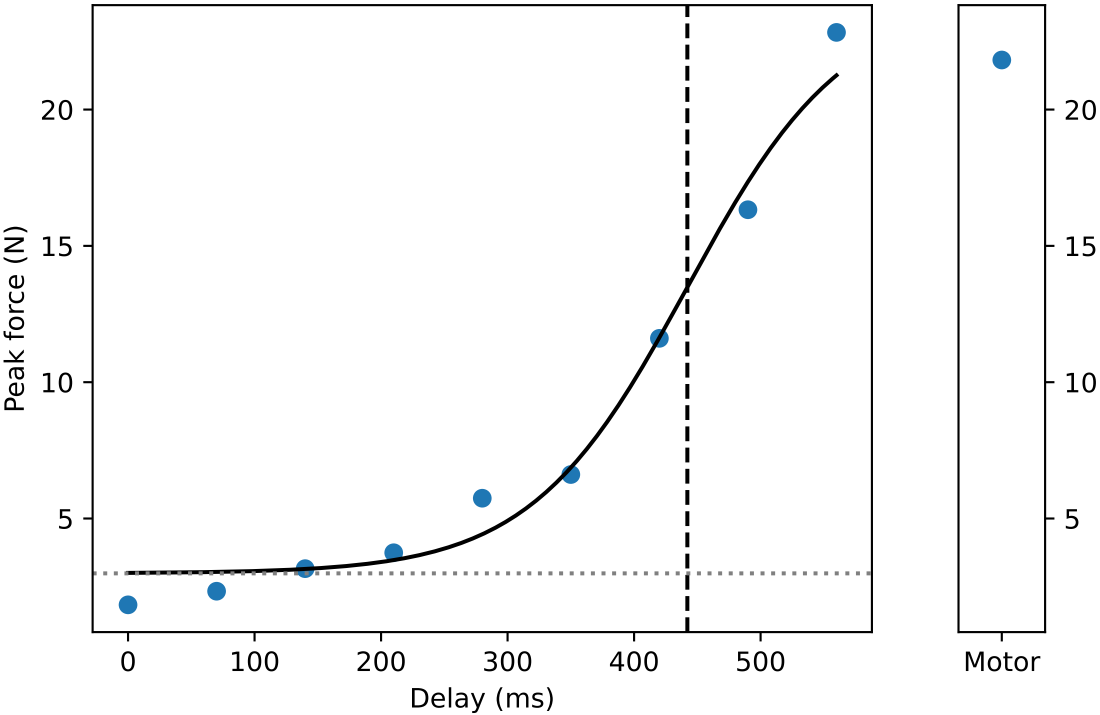

R^2: 0.98 Inflection point: 442.1 ms

Lower asymptote: 2.99 N; Upper asymptote: 23.98 N

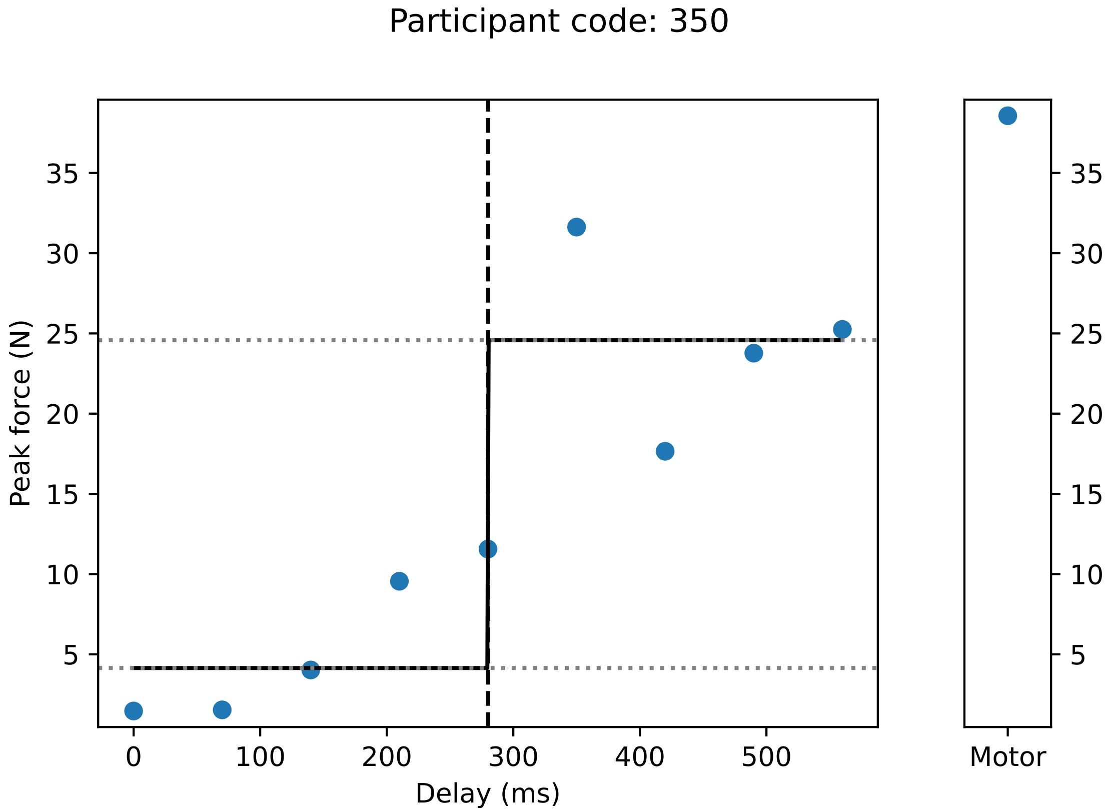

R^2: 0.86 Inflection point: 280.01 ms

Lower asymptote: 4.15 N; Upper asymptote: 24.57 N

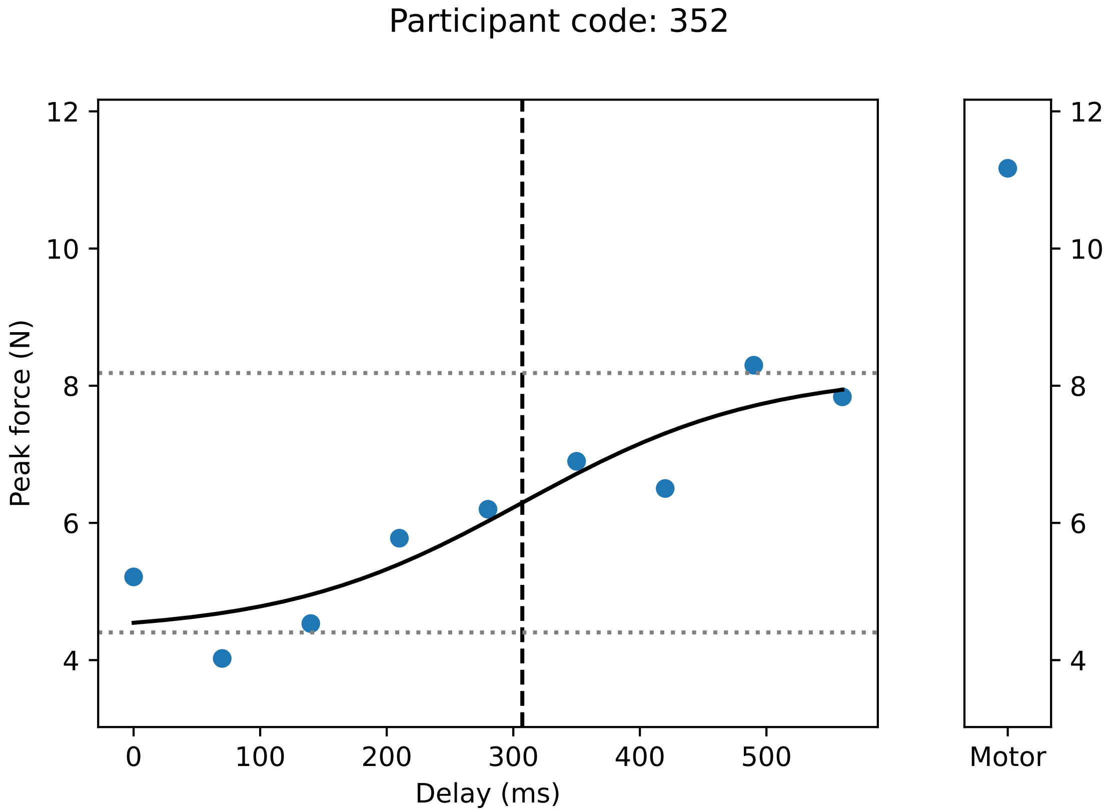

R^2: 0.86 Inflection point: 307.12 ms
Lower asymptote: 4.4 N; Upper asymptote: 8.19 N

Participant code: 355

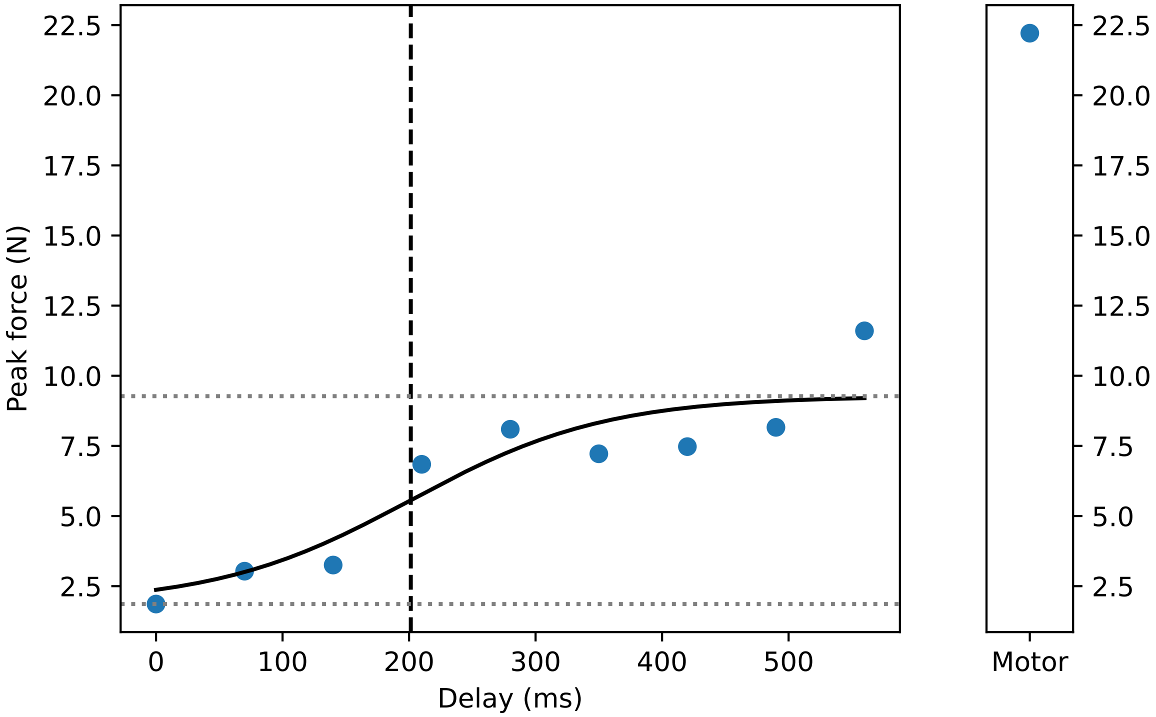

R^2: 0.84 Inflection point: 201.37 ms

Lower asymptote: 1.86 N; Upper asymptote: 9.27 N

Participant code: 357

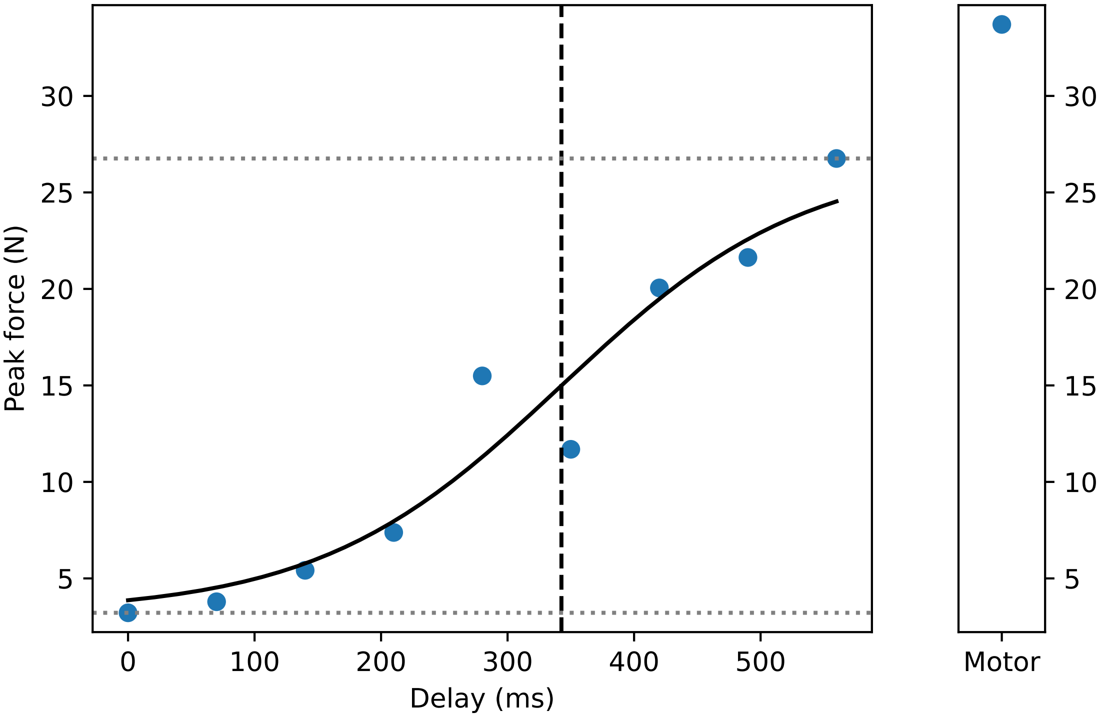

R^2: 0.93 Inflection point: 342.57 ms

Lower asymptote: 3.22 N; Upper asymptote: 26.76 N

Participant code: 358

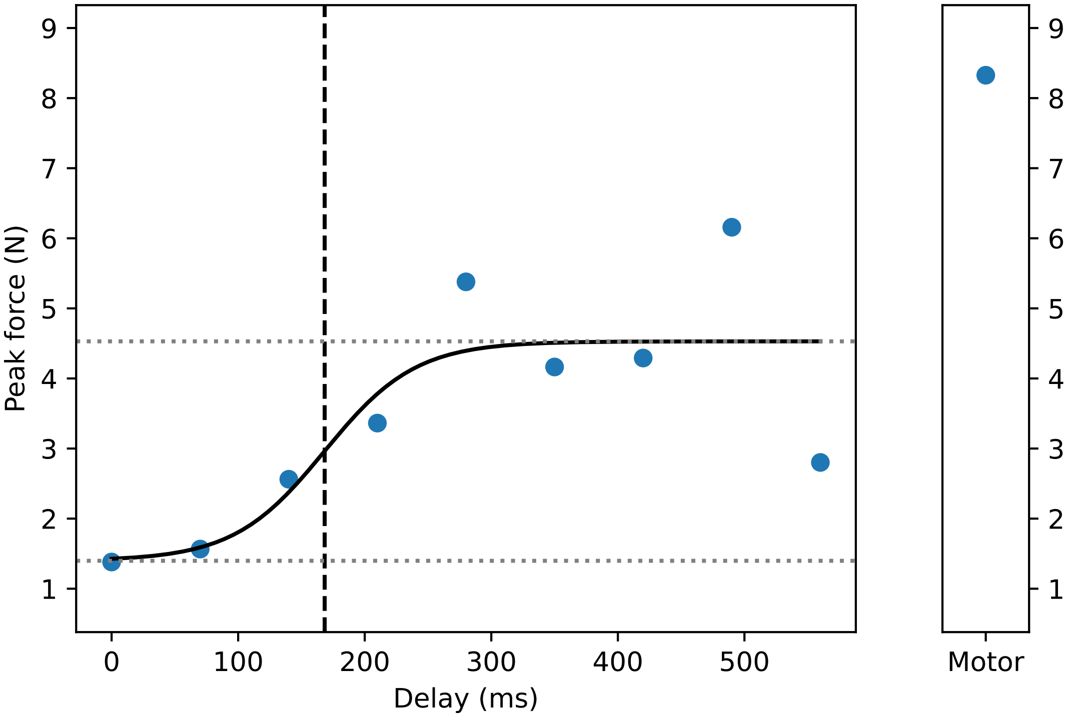

R^2: 0.67 Inflection point: 168.34 ms

Lower asymptote: 1.4 N; Upper asymptote: 4.53 N

Participant code: 369

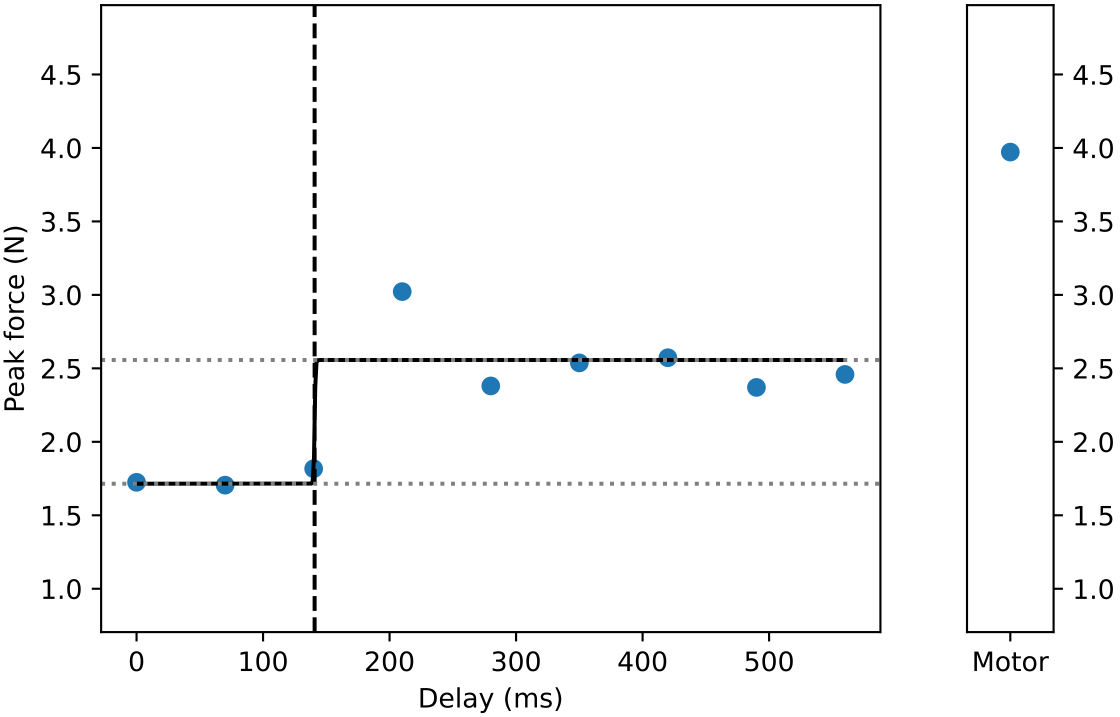

R^2: 0.82   Inflection point: 140.76 ms
Lower asymptote: 1.71 N; Upper asymptote: 2.56 N

Participant code: 371

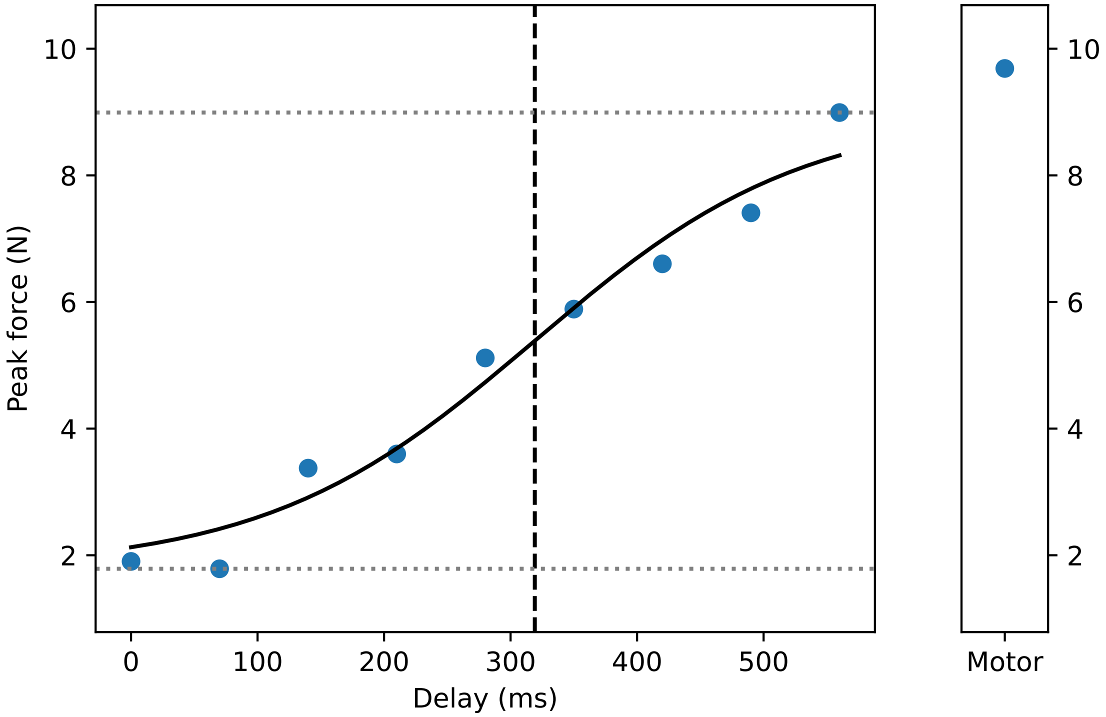

R^2: 0.97 Inflection point: 319.16 ms
Lower asymptote: 1.79 N; Upper asymptote: 8.99 N

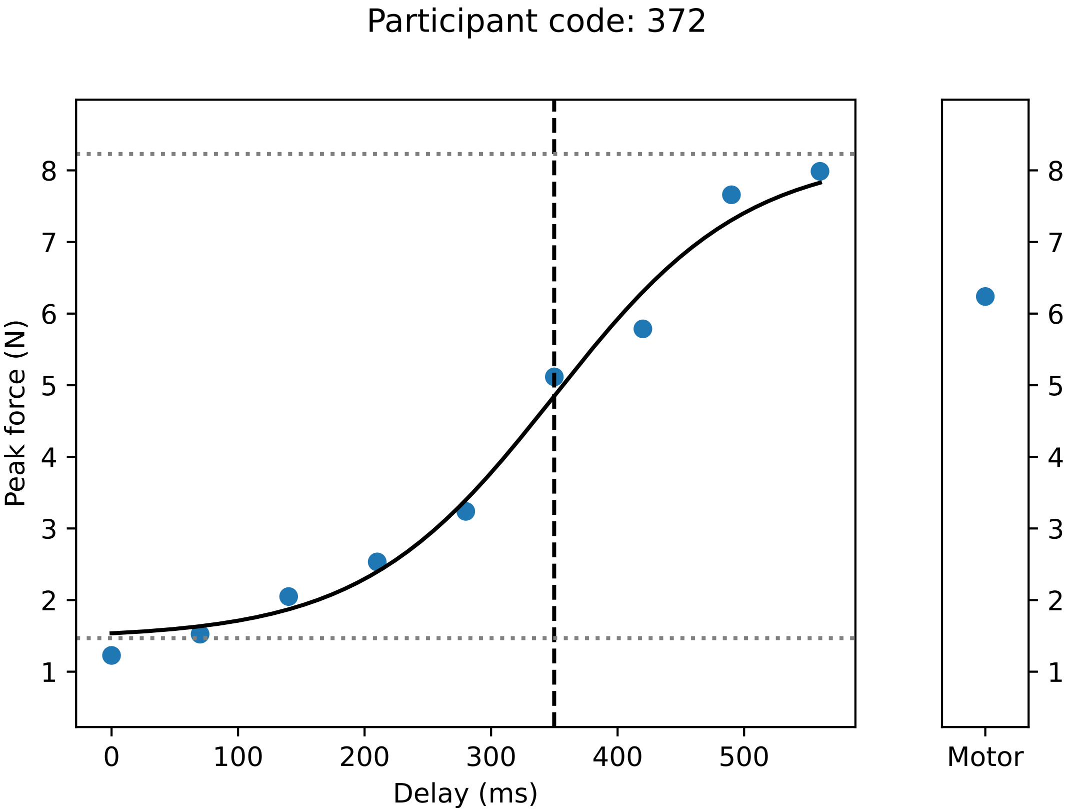

R^2: 0.99   Inflection point: 349.89 ms
Lower asymptote: 1.47 N; Upper asymptote: 8.23 N

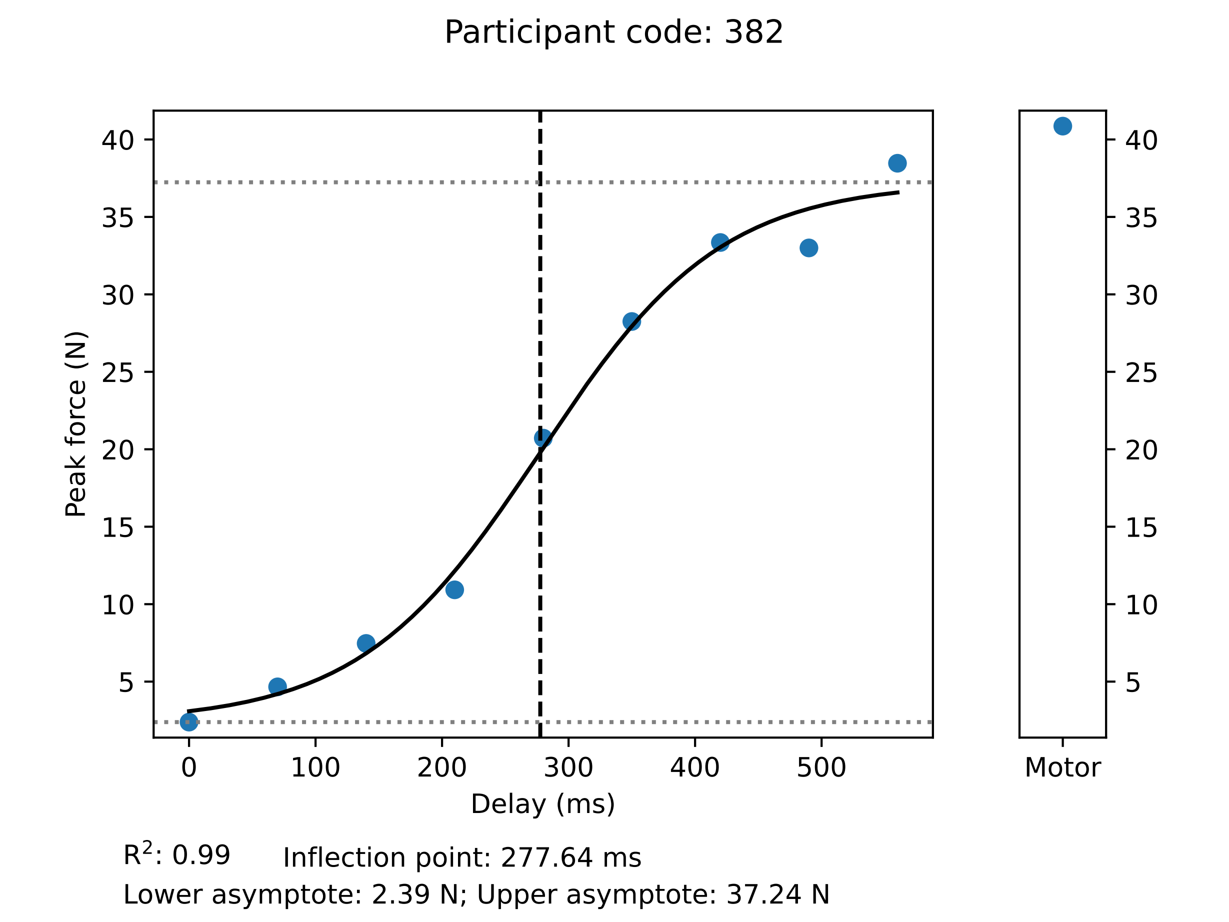

Participant code: 382
40
35
30
25
20
15
10
5
Peak force (N)
0
100
200
300
400
500
Delay (ms)
Motor
R²: 0.99
Inflection point: 277.64 ms
Lower asymptote: 2.39 N; Upper asymptote: 37.24 N

Participant code: 387

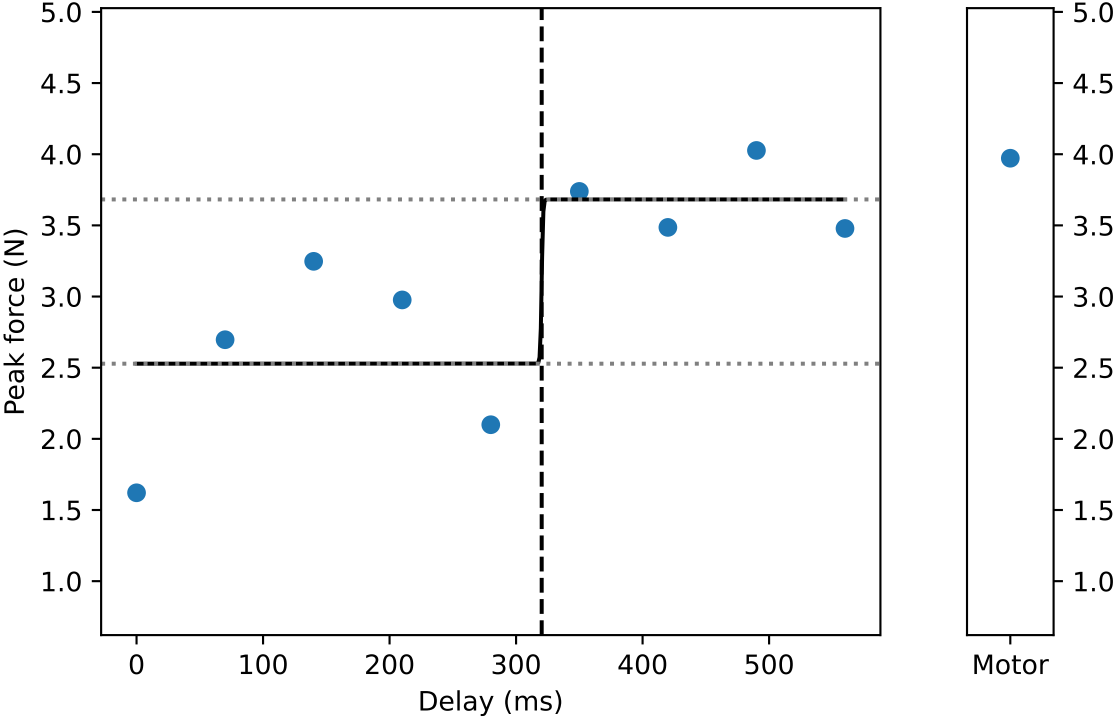

R^2: 0.6 Inflection point: 320.25 ms

Lower asymptote: 2.53 N; Upper asymptote: 3.68 N

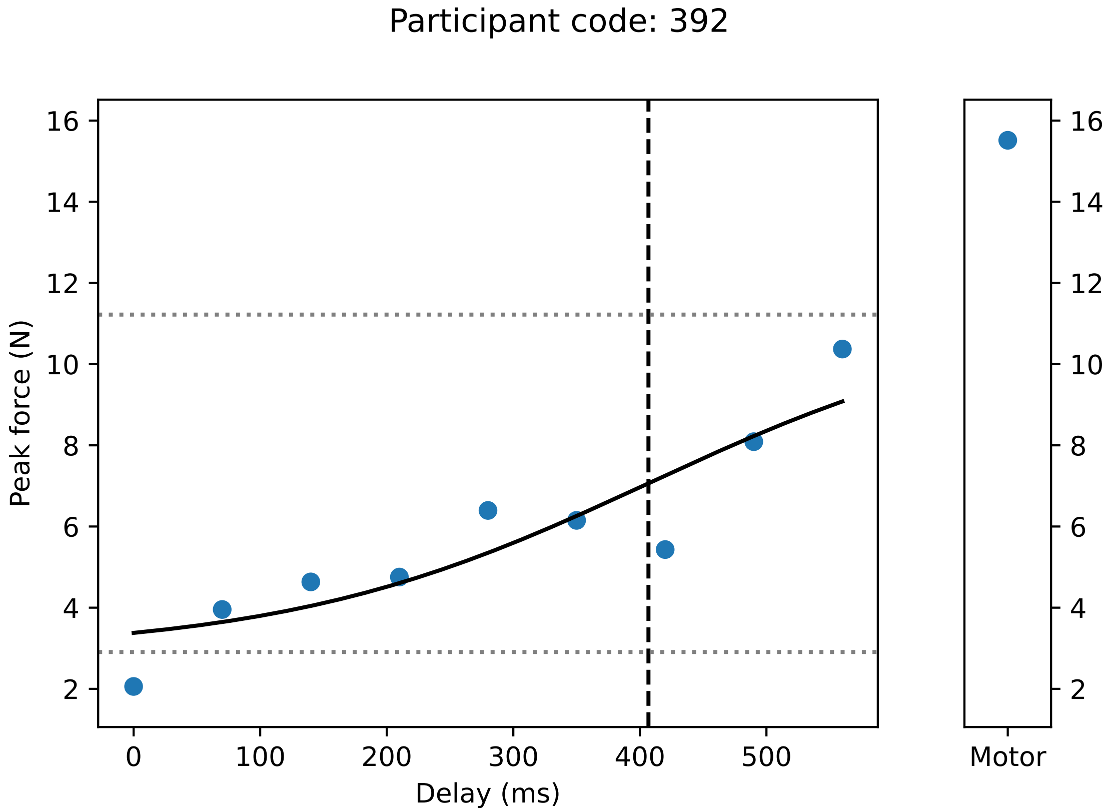

R^2: 0.82 Inflection point: 406.78 ms
Lower asymptote: 2.91 N; Upper asymptote: 11.22 N

Participant code: 396

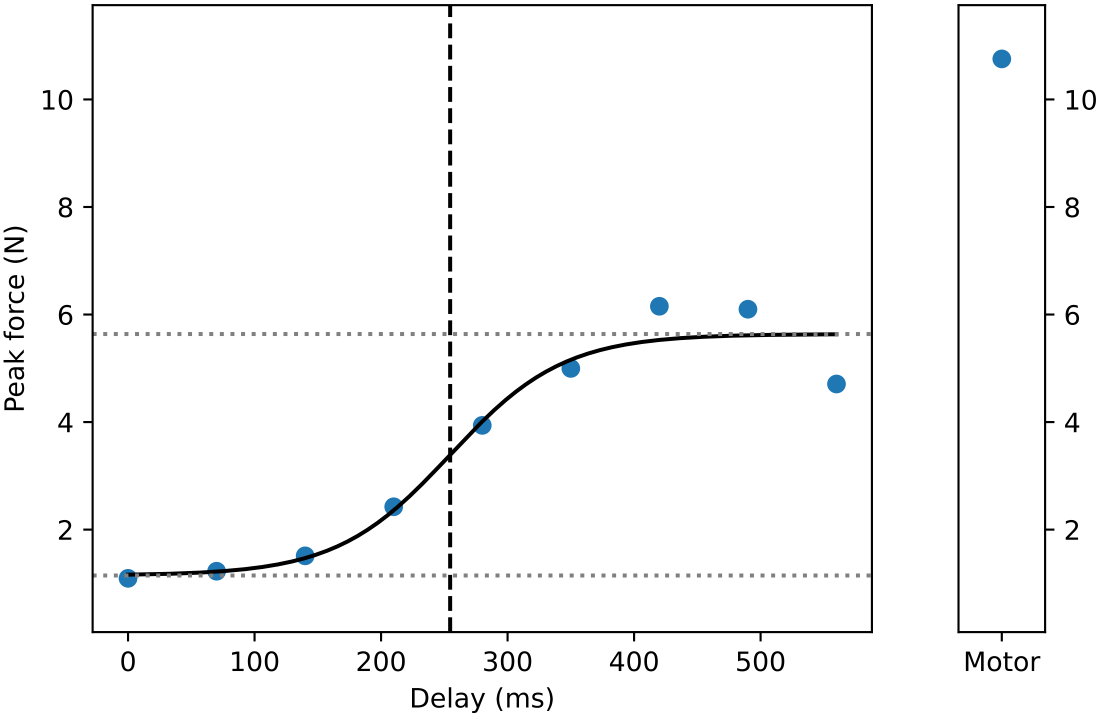

R^2: 0.95 Inflection point: 254.61 ms

Lower asymptote: 1.15 N; Upper asymptote: 5.64 N
